# Hypervalent chalcogenonium organocatalysis for the direct stereoselective synthesis of deoxyglycosides from hemiacetals

**DOI:** 10.1039/d5sc07018j

**Published:** 2026-01-07

**Authors:** Jennifer Johns, Mukul Mahanti, Thomas Hansen, M. Carmen Galan

**Affiliations:** a School of Chemistry, University of Bristol Cantock's Close Bristol BS8 1TS UK mukulmahanti@gmail.com m.c.galan@bristol.ac.uk; b Department of Chemistry and Pharmaceutical Sciences, Amsterdam Institute of Molecular and Life Sciences (AIMMS), Vrije Universiteit Amsterdam De Boelelaan 1108 1081 HZ Amsterdam The Netherlands t.hansen@vu.nl

## Abstract

Lewis acids are frequently used as catalysts in glycosylation reactions, however these reagents often suffer from significant limitations such as sensitivity to moisture and poor stereocontrol. Chalcogenonium catalysts have recently emerged as a new class of catalysts with improved Lewis acidity and stability. Here we describe a proof of concept study of the use of 1,2-oxaselenonium salts as effective organocatalysts for the direct and stereoselective dehydrative glycosylation with 1-hydroxy carbohydrates to give deoxyglycosides. The reaction is high yielding, stereoselective and amenable to a wide range of nucleophiles, including primary, secondary and tertiary alcohols and thiols. Experimental and computational mechanistic investigations suggest that the reaction proceeds through a cooperative mechanism involving the hemiacetal donor, acceptor, and catalyst. In this process, the Lewis acidic selenonium catalyst activates the donor, while the incoming alcohol nucleophile engages in a stabilizing hydrogen-bond interaction with the chalcogenonium triflate counterion. DFT calculations suggest a loose S_N_2-like transition state with a high degree of oxocarbenium ion character, reminiscent of the mechanism observed for glycosyl-modifying enzymes. The methodology is exemplified on the stereoselective synthesis of a tetrasaccharide in 52% yield.

## Abstract 2

The direct hemiacetal activation using chalcogenonium salts for the α-selective synthesis of deoxyglycosides is demonstrated. The reaction mechanism is studied using a combination of kinetic studies, computational and experimental techniques.

## Graphical abstract

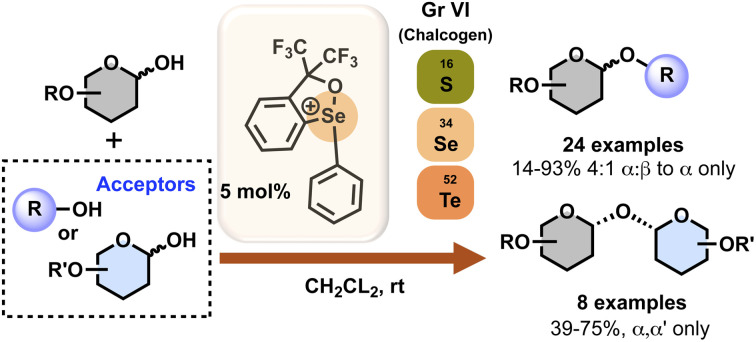

## Introduction

The controlled assembly of complex oligosaccharides and glycoconjugates from monosaccharide precursors, which play a myriad of biological roles in all life processes,^1,2^ is essential to advance the frontiers of glycobiology research.^3–6^ A crucial step in carbohydrate synthesis is the formation of the glycosidic bond. Most chemical approaches rely on the introduction of a latent leaving group at the anomeric position, which can be activated in the presence of a nucleophile acceptor to undergo the coupling step.^7,8^ A less explored strategy for glycosidic bond formation is the dehydrative coupling in which a 1-hydroxy carbohydrate or hemiacetal can be activated directly to generate a highly reactive species to undergo glycosylation *in situ*, thus potentially leading to a more efficient process.^9,10^ However, lack of control over the reversibility of the reaction, which in the absence of an excess of nucleophile can lead to incomplete reactions or hemiacetal donor dimerization *via* self-coupling,^11,12^ has limited its broad utility.

**Scheme 1:**
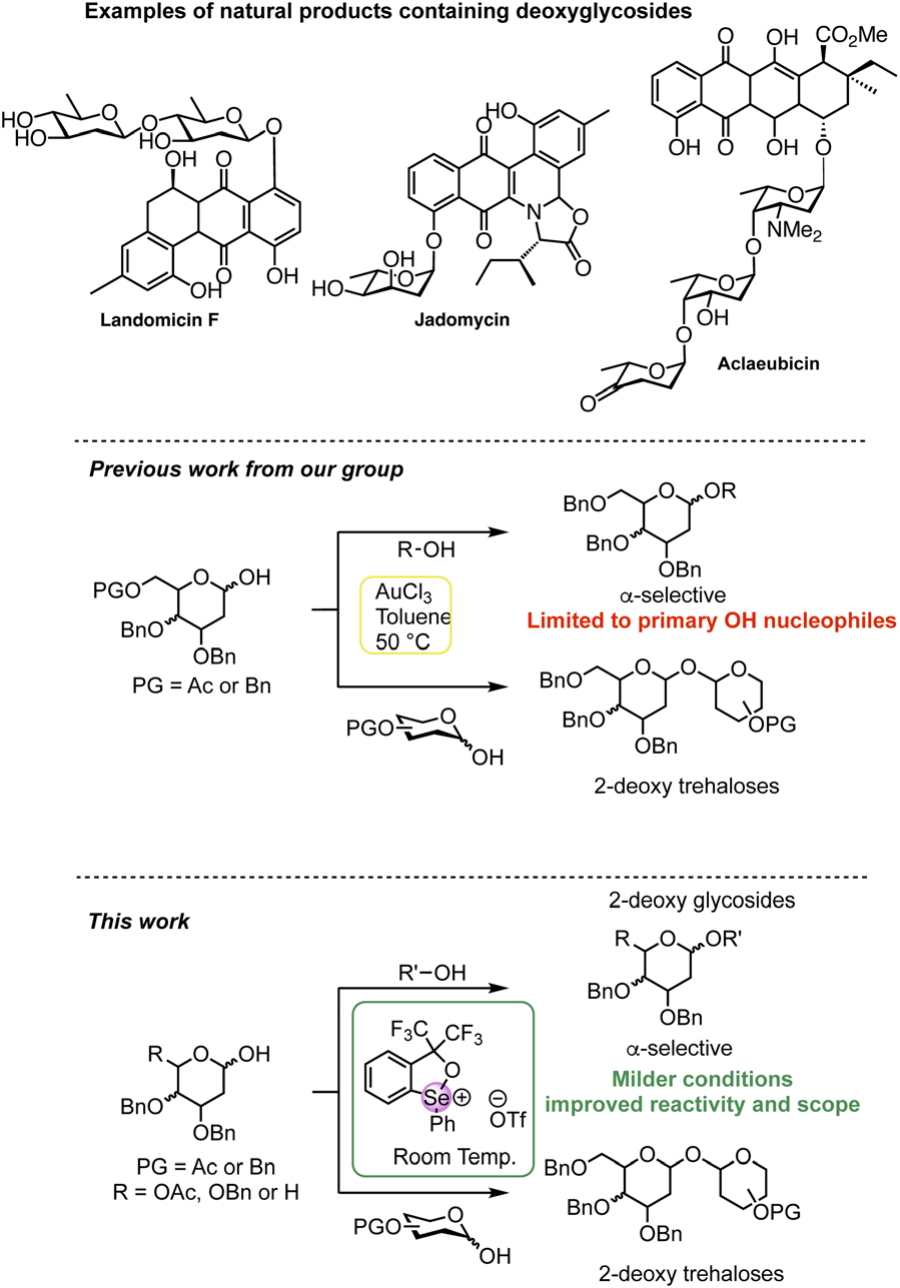
Examples of deoxyglycoside-containing natural products. Previous work: Au(iii) activation of hemiacetal donors; current work: chalcogenonium activation of hemiacetal donors. For a comprehensive review on hemiacetal activation see ref. 2 and 3.

Chiral acetals are ubiquitous in many natural products, ranging from spiroketal polyketides to complex oligosaccharides with a wide range of biological activities. 2-Deoxy-hexoses are important components of many active natural products such as antibiotics and anti-cancer agents (Scheme 1).^13^ The absence of substituents at C-2 poses significant synthetic challenges in directing the approach of the incoming nucleophile during the glycosylation reaction. This has spurred efforts to devise improved and stereoselective protocols for their assembly.^5,14–37^ The direct synthesis of deoxyglycosides from an activated electrophilic deoxy-sugar donor with a nucleophile (or acceptor) is the most straightforward strategy and a number of elegant approaches in recent years have been developed,^4,34^ including examples of dehydrative hemiacetal activation.^24,37–48^ We recently reported a catalytic AuCl_3_-catalysed dehydrative glycosylation using hemiacetal glycosyl donors and acceptors to access 1,1-α,α′-linked 2-deoxy trehalose analogues with high stereoselectivity (Scheme 1).^49^ Although glycosylation with primary OH nucleophiles was also possible, lower yields (10–20%) of the desired 2-deoxy glycoside products were observed with less reactive secondary alcohols due to competitive dimerization of the donor, even when an excess of the alcohol was used. These findings prompted us to investigate more efficient catalysts capable of modulating the activation of the hemiacetal donor and reactivity of the incoming nucleophile to yield 2-deoxyglycosides more efficiently.

Organoselenium chemistry has undergone rapid growth in the last few decades due to the increasing applications in organic synthesis.^50–53^ Chalcogen bonding (ChB)-catalysis has been applied in many intermolecular or intramolecular reactions,^54,55^ including recent elegant examples on carbohydrate chemistry using phosphonochalcogenide catalysts.^56–58^ A new class of chalcogen bond donors based on trisubstituted selenonium salts have emerged, exhibiting improved reactivity as Lewis acids in electrophilic halogenations and aldol-type reactions. These 1,2-oxaselenonium salts are thought to be more Lewis acidic than the more common divalent chalcogenides and able to catalyse reactions *via* unconventional seleniranium ion-like intermediates,^55^ due to the more positive charge at the chalcogen centre.^55^ These chalcogenonium salts can offer high directionality (interaction angle of *ca.* 180°), which allows for better control of the interaction between the catalyst and substrate and easy tunability of the chalcogenonium bond system. Their Lewis acidity and steric demand can be modulated by the choice the substituents, moreover coordinating counter anions occupying the σ-hole of Se can also sterically interfere with the catalytic interactions.^59,60^ Despite the growing interest in the catalytic properties of organo-chalcogenonium compounds based on group 16 elements in synthetic applications,^61^ there are very few examples in carbohydrate chemistry^62,63^ and no reports of electrophilic catalysts featuring a reactive cationic Se centre have been reported to date. We hypothesized that the unique reactivity of hypervalent chalcogenonium salts could help overcome some of the current challenges on the activation of hemiacetal donors in glycosylation reactions.

## Results and discussion

**Scheme 2:**
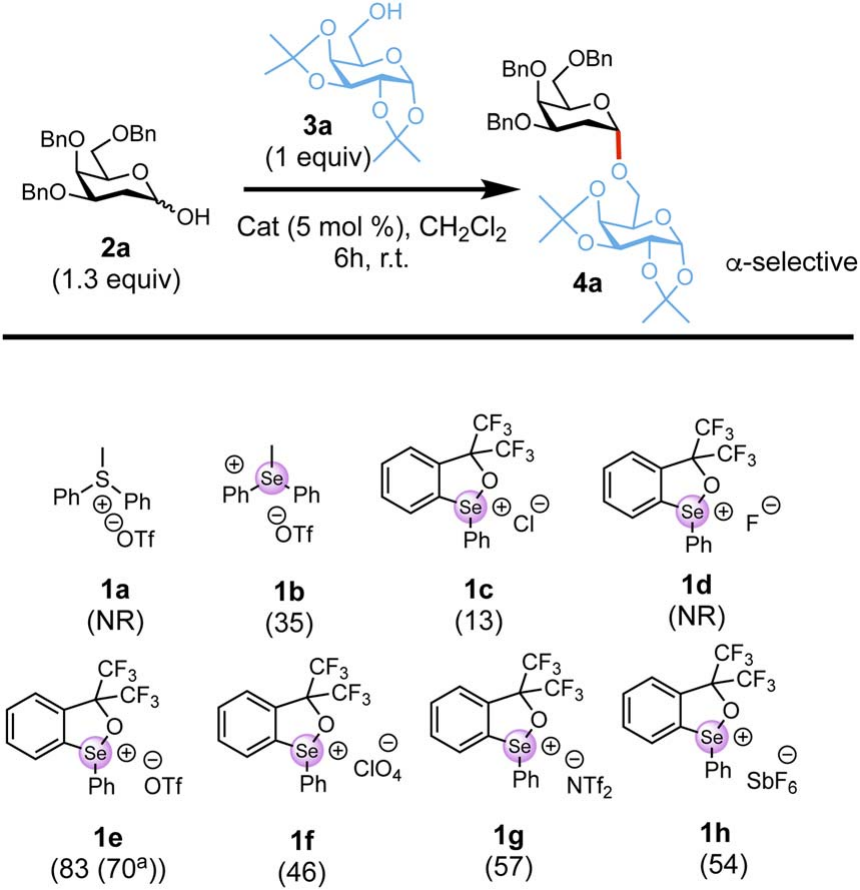
Catalyst screen for the glycosylation reaction with hemiacetal 2a with 3a. Conversion as determined by ^1^H-NMR shown in brackets; ^a^isolated yield; NR = No reaction.

The study began with the evaluation of a series of soft chalcogenonium salts (1a–1h) in a model glycosylation reaction of tri-benzylated 2-deoxy galactosyl hemiacetal 2a^64^ and galactoside acceptor 3a in CH_2_Cl_2_ at room temperature for 6 h. As summarized in (Scheme 2) chalcogenoniums featuring a common Se or S centre substituted with two aryl C–Se/S bonds and one Se/S–CH_3_ (1a–b) were screened. We also explored catalysts with a Se–O rigid ring core structure (1c–1h), which adopt a trigonal bipyramidal geometry with the cyclic selenide occupying the trigonal plane and are generally more reactive.^55,59^ Different counterions, exhibiting distinct metal coordination abilities and hydrogen-bond acceptor characters that can tune catalyst reactivity,^52,59^ were also screened. We found that rigid selenonium triflate 1e at 5 mol% catalyst loading at room temperature in CH_2_Cl_2_ afforded optimal conversion (83%) to the desired glycoside 4a with complete α-stereocontrol, thus suggesting both the choice of selenonium scaffold and counter anion have a significant effect on the catalysis. Changing the reaction temperature to 0 °C slowed the reaction, whilst no significant improvement was observed at 40 °C. Finally, running the reaction in solvents such as acetonitrile, ethyl acetate, dichloroethane, THF or toluene was detrimental to the overall yield (see Tables S1–S3 in SI).

**Scheme 3:**
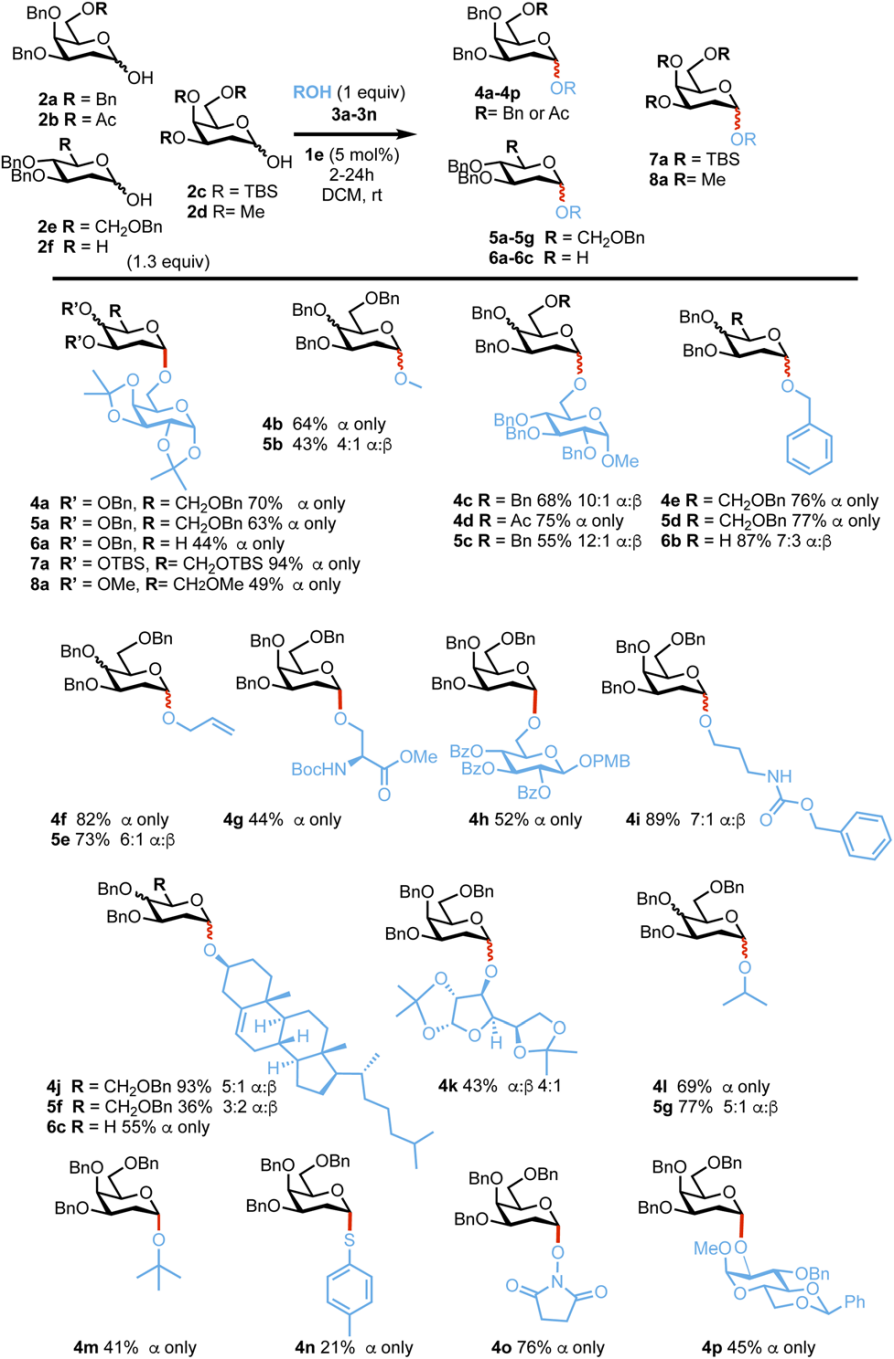
Substrate scope with donors 2a–f and ROH (3a–n). α : β selectivities were calculated from ^1^H-NMR on isolated products, yields provided are also from isolated products.

With optimized conditions in hand, we next examined the reaction substrate scope. 1-Hydroxy 2-deoxygalactoses 2a and 2-deoxyglucoses 2b^65^ and 2c^66^ were reacted with a range of primary and secondary OH nucleophiles 3a–3n under the optimized reaction conditions (Scheme 3). In most cases, reactions proceeded smoothly with high α-selectivity, demonstrating that the catalytic system is tolerant of common alcohol and amine protecting groups such as acetals, ethers, esters and carbamates. Glycosylation of 2-deoxygalactoses 2a, 2b, 2c or 2d with primary alcohols such as simple alcohols 3b, 3d, 3e, 3i or glycoside acceptors 3a, 3c and 3g and amino acid 3f afforded the corresponding glycoside products in 43–87% yield and with a 3 : 2 α : β to only α ratios, whilst reactions with secondary nucleophiles such as 3i–3k and 3p or tertiary alcohols (*e.g.*3l) also afforded the desired products in good yields (41–93%) and α-selectivity. Glycosylations with thiotoluene 3m prove to be more challenging affording lower yields (21%) but high α-stereocontrol. Pleasingly, reactions with 2,6 dideoxyglucoside 2f or the less reactive 2-deoxyglucoside 2e afforded the desired products in good yields and α-stereocontrol *e.g.*6a–6c (44–87% 7 : 3 α : β to α only) and 5a–5e, 5g–5i (36–77%, 3 : 2 α : β to only α), respectively.^67^ Reactions with fully oxygenated perbenzylated or peracetylated galactoside lactols were unsuccessful.

**Scheme 4:**
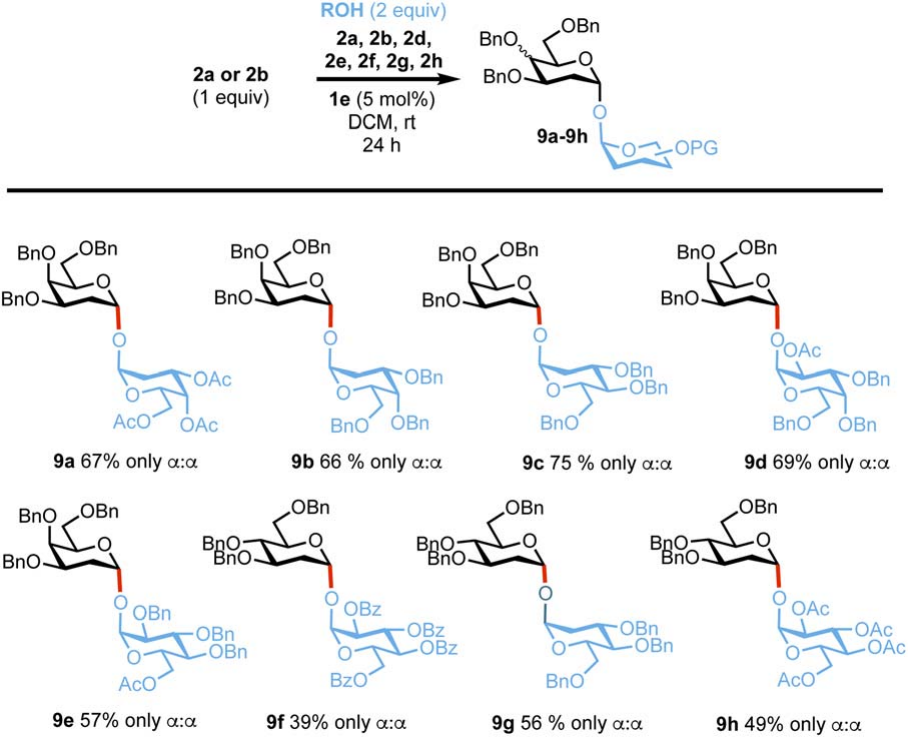
Synthesis of trehalose analogues 9a–9h. α : β selectivities were calculated from ^1^H-NMR on isolated products, yields provided are also from isolated products.

Next, we evaluated the reactivity of the catalytic system in the synthesis of a 1,1,-α,α′ linkage, which are often more challenging targets due to the necessary assembly of two anomeric centres in one step in the presence of other competing pathways.^49,68,69^ Reactions of either lactol 2a or 2c with a range of hemiacetal acceptors of differing reactivity (2a, 2c, 2d–2h) proceeded smoothly to give the desired products 9a–9e in yields of 39–75% and with complete α,α′-stereocontrol (Scheme 4).^70^

**Scheme 5:**
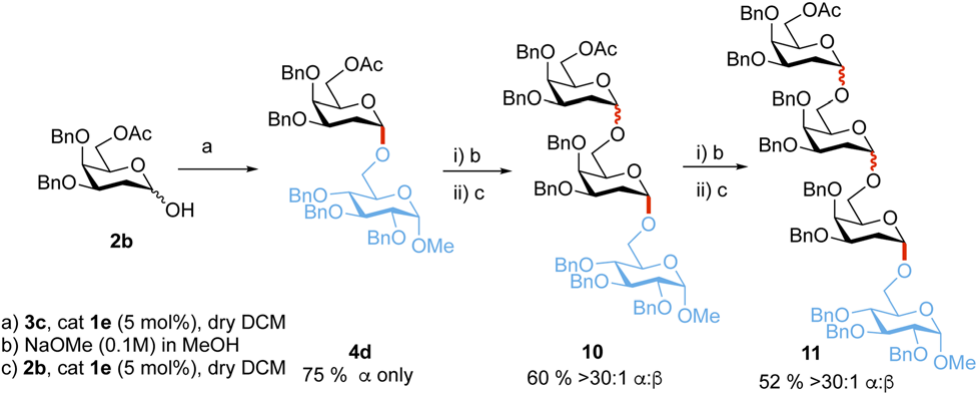
Iterative synthesis of tetrasaccharide 11. α : β selectivities were determined from ^1^H-NMR on isolated products, yields provided are also from isolated products.

Additionally, to showcase the utility of the methodology, the sequential synthesis of tetrasaccharide 11 was performed (Scheme 5). Glycosylation of 2b and 3c followed by three sequential deprotection and glycosylation steps afforded tetrasaccharide 11 in 23% overall yield and >30 : 1 α-stereoselectivity.

Preliminary ^1^H-NMR spectroscopy studies of mixtures of hemiacetal 2a and selenonium 1e in CD_2_Cl_2_ showed broadening of the catalyst protons in the aromatic region (*δ* 8.10–7.90 and 7.75–7.55 ppm) of the catalyst and formation of the 2a-α,α′-dimer 9b (*δ* 5.26 ppm) as the concentration of 2a increases (Fig. S1), suggest an interaction between the hemiacetal and the catalyst. ^1^H NMR titrations of OH acceptor 3a and 1e also show broadening of the catalyst's aromatic protons and a proton shift for the OH signals (Fig. S2a–c). The interaction between the catalyst and 3a is further supported by IR analysis of the mixture showing suppression of the IR alcohol stretch frequencies (3479–3413 cm^−1^, Fig. S3) and a small ^19^F chemical shift of the fluorine CF_3_ signals in the catalyst upon addition of the alcohol (Fig. S2d). Whilst it is difficult to disregard the possibility of a pi–aromatic–alcohol interactions with the OH moieties, as reported by Pederson,^71^ on account of the oxyphilic nature of chalcogenonium species^55,62^ and based on these initial results, we hypothesized that the catalyst could form a complex with the hemiacetal donor and incoming nucleophile to generate an activated catalytic species.

**Scheme 6:**
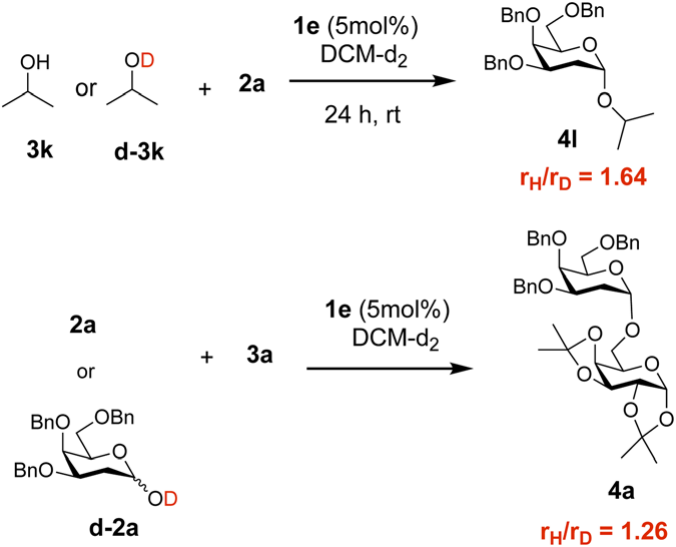
Kinetic isotope effect: reaction of 3k or d-3k with 2a and 2a or d-2a with 3a in the presence of 1e. Rate constants were calculated from initial rate measurements.

To further understand the process, control reactions were carried out between 2a and either isopropanol 3k or deuterated isopropanol d-3k as the nucleophile, in the presence of catalyst 1e (Scheme 6 and Fig. S15). In general, reaction rates with deuterated substrates were slower than those of non-deuterated acceptors (*r*_H_/*r*_D_ = 1.68), which suggest that breaking the O–H bond is potentially a key step in the reaction mechanism. Additionally, reaction rates for reactions between deuterated lactol 2a or d-2a and 3a also showed a reaction rate difference (*r*_H_/*r*_D_ = 1.26). It is worth noting that the hemiacetal H/D could readily exchange with the acceptor and thus any KIE observed is likely the result of both the partially deuterated donor and acceptor and should be taken as average values (Scheme S3 and Fig. S16–S29 for computational data).

Interestingly, we further identified in the ^1^H-NMR spectra of the model reaction between 2a and 3a (Scheme 6), a time-dependent shift of the anomeric protons of the hemiacetal 2a (*δ* 5.43 ppm), and acceptor 3a (*δ* 5.55 ppm), whereas product 4a (*δ* 5.50 ppm), exhibited only a minor shift. The anomeric signals of 2a were the most affected and particular the β-hemiacetal (α-anomer shift by 0.004 ppm, and β-anomer 0.02 ppm) (Fig. S11–S14). We also observed the formation of H_2_O over the course of the reaction.

Moreover, the reaction between 2a and 2e in the presence of 1e was monitored over time by ^1^H and HSQC NMR spectroscopy (Fig. S20–S25). Exclusive formation of the α,α-trehaloside 9c was observed, while the anomeric ratio of the unreacted 2a and 2e remained unchanged throughout the reaction, indicating that trehaloside product formation occurs without anomeric equilibration.

Additional experiments with 5 mol% of non-nucleophilic base 2,6-di-tertbutylpyridine (DTBP) added to the reaction of 2a and 3a in the presence of 1e, significantly slows down the reaction (only 28% conversion after 24 h *vs.* 83% at 6 h), whilst addition of a stoichiometric amount of the base completely inhibited the reaction, suggesting a H^+^ transfer mechanism that is disrupted by the DTBP acting as an scavenger (Scheme S4). To further evaluate the potential role of the triflic acid that could potentially be generated during the reaction due to the catalyst counterion, a control reaction between 2a and 3a in the presence of either 1 or 5 mol% TfOH as the sole catalyst was conducted. The reaction yielded an inseparable mixture of products in both instances, thus suggesting that although triflic acid can activate the hemiacetal donor, it is not directly responsible for the observed reactivity (Scheme S5 and Fig. S17). A much weaker acid, TFA (5 mol%), was also evaluated and gave no reaction. Next, an α/β-mixture of disaccharide 4k subjected to the reaction conditions in the absence and presence of a nucleophile MeOH and gave no change in anomeric ratio, indicating that the high α-selectivity is not the result of anomerisation (Scheme S6 and Fig. S18–S19).

**Fig. 1:**
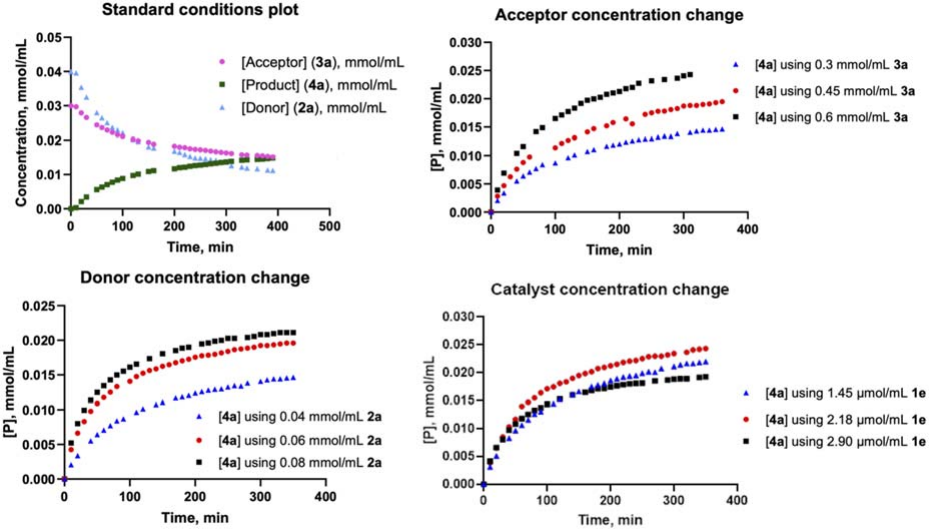
^1^H-NMR kinetics reaction profiles for: (A) glycosylation of 2a with 3a in the presence of 1e; (B) profile when modifying acceptor [3a]; (C) profile when modifying donor [2a]; (D) profile when modifying catalyst [1e].

In order to help elucidate the reaction mechanism, kinetic orders based on initial rates were determined by ^1^H-NMR by modifying the concentrations of donor 2a, acceptor 3a and catalyst 1e (Fig. 1, S5–S7 and Tables S5–S7). The reaction showed first-order kinetics with respect to the glycosyl donor and acceptor. Moreover, we also found that the reaction rate is dependent on the catalyst concentration, with an increase in rate at 1.5 × [1e], followed by a decrease at higher catalyst concentrations, likely due to catalyst aggregation^17^ or diversion into alternative reaction pathways (*e.g.* trehaloside formation (Fig. S1).

**Fig. 2:**
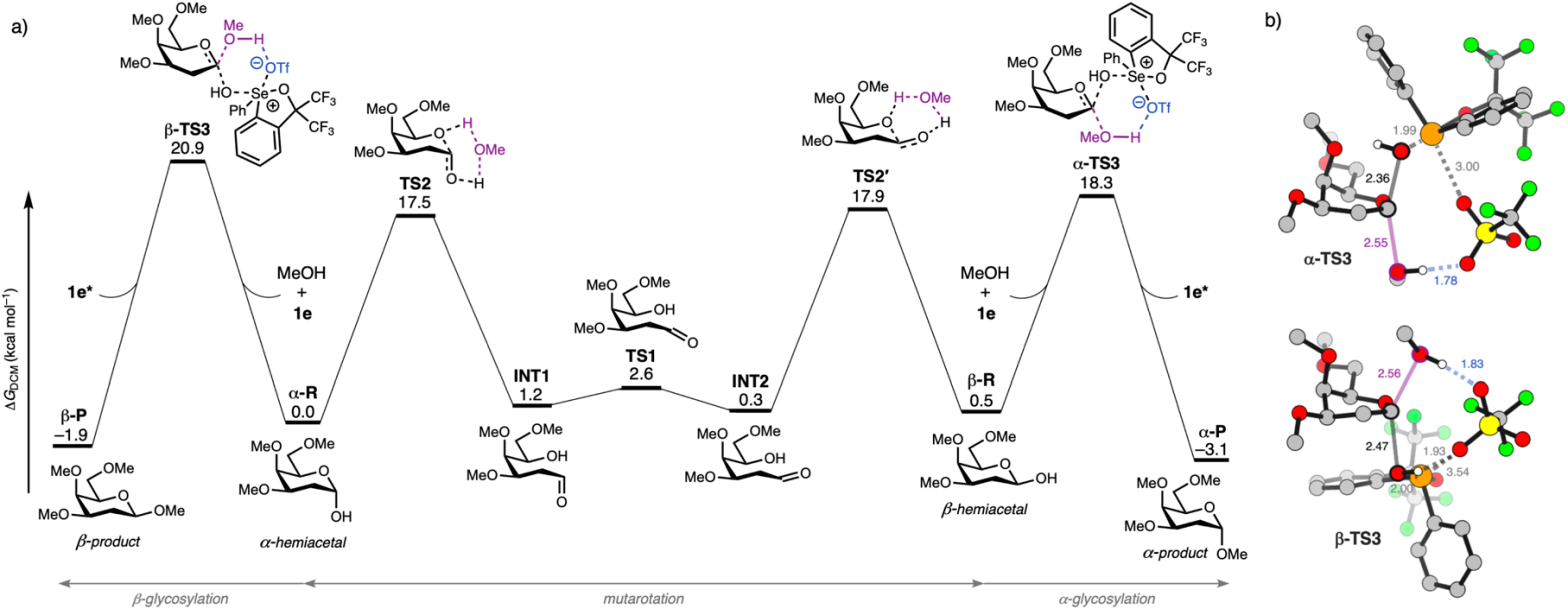
(a) Reaction profile of organocatalytic glycosylation reactions (Δ*G*_DCM_ in kcal mol^−1^) between 2-deoxy-galactosyl hemiacetal donor (α/β-R), methanol acceptor, and organocatalyst 1e in dichloromethane. (b) Key transition state structures for the organocatalytic glycosylation reactions (α/β-TS3) with key bond lengths (in Å). All non-polar hydrogens are omitted for clarity reasons. Atom colours: carbon (grey), fluorine (green), hydrogen (white), oxygen (red), selenium (orange), and sulfur (yellow). Computed at COSMO(DCM)-ZORA-BLYP-BJ(D3)/TZ2P.

Having established the synthetic utility of chalcogenonium salts as organocatalysts, we focused on understanding their catalytic mechanism using DFT computations. The overall lowest energy computed reaction profile for a model organocatalytic glycosylation reaction between a model 2-deoxy-galactosyl hemiacetal donor (α-R/β-R), methanol acceptor 3b, and organocatalyst 1e, with the energies relative to the separate reactants, is summarized in Fig. 2a (see SI Fig. S26–S30 for all computed pathways and data).^72,73^ We found that the two reactants, α-R and β-R can interconvert efficiently *via* mutarotation (highest barrier TS2′: Δ*G*^‡^_DCM_ = 17.9 kcal mol^−1^). In agreement with the experimentally observed α-selectivity, the hemiacetal β-R reacts with a lower barrier (α-TS3: ΔΔ*G*^‡^_DCM_ = −2.6 kcal mol^−1^) than α-R with MeOH catalysed by 1e to product P.^74^ In both transition states (Fig. 2b), the OH leaving group of the hemiacetal interacts with the selenium center (Se^+^) of catalyst 1e, thereby enhancing its leaving group capability. Upon addition of the nucleophile OH, the triflate counter ion (TfO^−^) stabilizes the proton of the incoming alcohol acceptor. Importantly, this interaction is not unique to the triflate counterion, as other computed anions (*e.g.* ClO_4_^−^, see SI Fig. S24) exhibit similar behaviour.

These S_N_2-like transition states are consistent with experimental kinetic data, showing concentration dependence on the catalyst, donor, and acceptor. Furthermore, the involvement of a key proton transfer step in the rate-determining transition state, as found by kinetic isotope effect (KIE) experiments, is consistent with the computed mechanism. Consistent with the kinetic preference of the reaction, α-P is also thermodynamically more stable than β-P by 1.2 kcal mol^−1^. Notably, an S_N_1 pathway proceeding *via* the 2-deoxygalactosyl oxonium ion (Δ*G*^‡^_DCM_ = 22.0 kcal mol^−1^) could represent a viable competing mechanism. It is worth noting that galactosyl oxonium ions have been associated with highly α-selective reactions.^75,76^ However, this route is higher in energy than both S_N_2 pathways and is not supported by our kinetic data. It is also plausible that in the absence of an available OH glycosyl acceptor, the activated 1-OH hemiacetal can also act as the nucleophile as evidenced by the NMR titration data (Fig. S1) to give the observed trehalosides, albeit this is a much slower process as per our calculations and experimental results.

## Conclusions

**Scheme 7:**
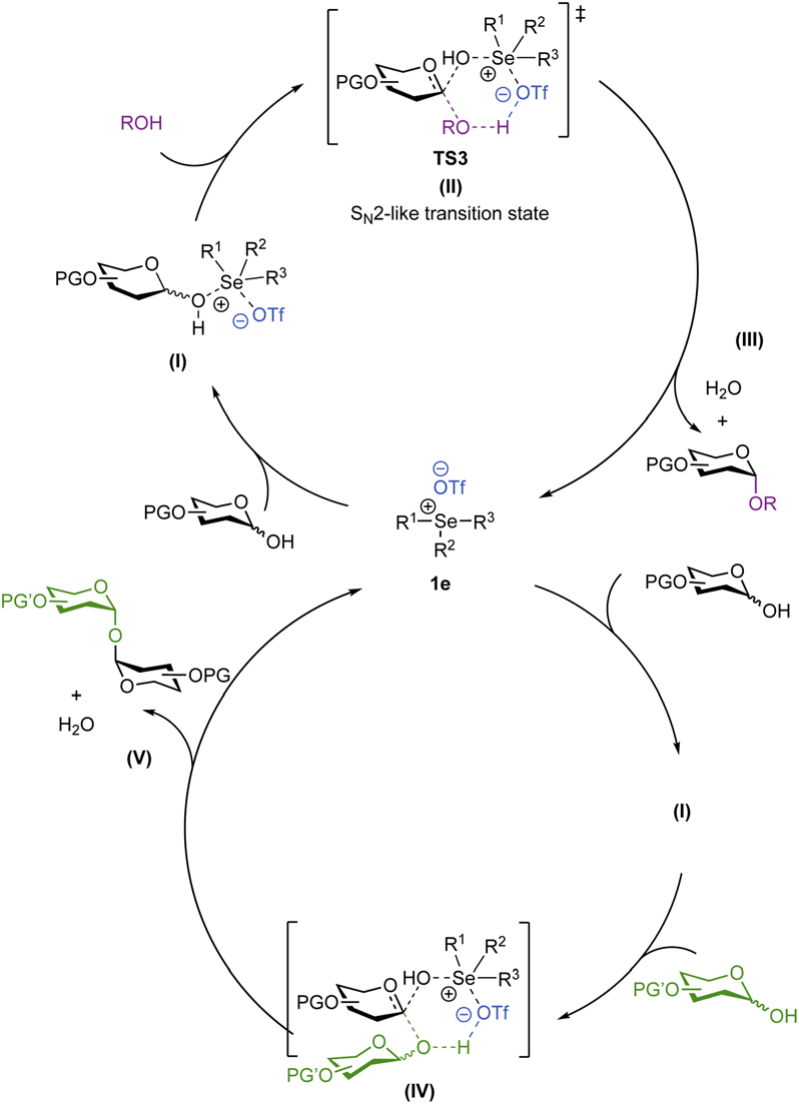
Proposed mechanism for the studied chalcogenonium catalyzed glycosylation reactions.

In conclusion, we have described the first application of trisubstituted selenonium salts for the direct stereoselective synthesis of deoxyglycosides directly from hemiacetals, obviating the need for anomeric functionalization. The reaction conditions are mild, compatible with most common protecting groups and are demonstrated to be effective in the iterative synthesis of a tetrasaccharide in 52% overall yield. Improving on previous methods, primary, secondary and tertiary alcohols can all be utilized to afford a range of α-glycoside products. Based on experimental and computational evidence, we invoke a stepwise mechanism (Scheme 7), in which the glycosyl donor, acceptor and the catalyst are participants in the rate-limiting step. We propose that upon the catalytic activation of the hemiacetal donor, a chalcogonenium complex, TS3 (II), is formed *in situ* and features a stabilizing hydrogen bond interaction between the incoming alcohol nucleophile and the chalcogenonium triflate,^77^ to help promote the key H^+^ transfer step to yield the product with high stereocontrol. DFT calculations suggest that both S_N_2 and S_N_1-type pathways are feasible suggesting a loose S_N_2-like transition state with a high degree of oxocarbenium ion character, reminiscent of the mechanism observed for glycosyl-modifying enzymes.^78,79^

This study opens new avenues for developing mild non-metallic catalysis for the stereoselective synthesis of complex glycosides and highlights the potential of chalcogenonium salts as a new class of catalysts for challenging glycosylation chemistry.

## Methods section

Hemiacetal donors 2a–2d (∼20–100 mg, 1.3 eq.) and acceptors 3a–3n (1.0 eq.) were added to a microwave tube or round bottom flask depending on scale and placed under N2 and anhydrous CH_2_Cl_2_ (1 mL solvent per 10 mg of donor) was added to dissolve the substrates. 5 mol% of catalyst 1e was then added, and the mixture was stirred until the reaction was deemed to be complete by TLC, after which the reaction mixture was concentrated under reduced pressure and the crude products were purified using silica gel flash column chromatography. For specific details for each substrate and full characterization, see SI.

## Author contributions

JJ, MM, and TH: investigation, methodology, formal analysis. MM and MCG: conceptualization. JJ, MM, TH and MCG: writing – review & editing, visualization. MCG and TH resources, project administration, funding acquisition, formal analysis, project administration, direct lab supervision and data validation.

## Conflicts of interest

There are no conflicts to declare.

## Supplementary Material

SC-OLF-D5SC07018J-s001

## Data Availability

The data supporting this article have been included as part of the supplementary information (SI). This includes synthetic and computational protocols and characterization data for all compounds, including NMR spectra. Supplementary information is available. See DOI: https://doi.org/10.1039/d5sc07018j.
