# Supplementary material for: Hypervalent chalcogenonium organocatalysis for the direct stereoselective synthesis of deoxyglycosides from hemiacetals
Source: Chem Sci. 2026 Jan 7;17(10):4991–7. doi: 10.1039/d5sc07018j (PMC12814205; doi:10.1039/d5sc07018j)
Supplement: SC-017-D5SC07018J-s001 [file SC-017-D5SC07018J-s001.pdf]

Supplementary Information (SI) for Chemical Science.
This journal is © The Royal Society of Chemistry 2026

Supporting Information

Hypervalent Chalcogenonium Organocatalysis for the Direct Stereoselective Synthesis of Deoxyglycosides from Hemiacetals

Jennifer Johns[a], Mukul Mahanti[a]*, Thomas Hansen[b]* and M. Carmen Galan[a]*

[a]School of Chemistry, University of Bristol, Cantock's Close, Bristol, BS8 1TS
[b]Department of Chemistry and Pharmaceutical Sciences, Vrije Universiteit Amsterdam, De Boelelaan 1108, 1081 HZ Amsterdam, The Netherlands

E-mail: mukulmahanti@gmail.com, t.hansen@vu.nl and m.c.galan@bristol.ac.uk

Contents

General Experimental 2
Synthesis of the Starting materials 3
  Synthesis of the catalysts 3
  Synthesis of the acceptors 5
  List of glycosyl acceptors used for the synthesis of 2-deoxy glycosides. 5
  Synthesis of 2-deoxy hemiacetals 2a- 2h 6
General procedure A: Se-catalysed glycosylation reactions 6
Reaction optimisation 6
Synthesis of 2-deoxy glycosides 9
General Glycosylation procedure B 29
General Glycosylation procedure C 29
Synthesis of trehalose derivatives 29
Mechanistic Study 38
^{1}H NMR titration study of the nucleophile 3a and catalyst 1e 39
Kinetics Study 43
Deuterium kinetic study 51
Control experiments with base 55

Control experiment with TfOH 57

NMR anomerization studies 58

Computational Details 68

NMR Spectra 112

General Experimental

All reactions, unless otherwise stated, were carried out at room temperature under an inert (N_2) atmosphere in standard glassware. Compositions of solvents are given as ratios of volumes unless otherwise stated. Anhydrous solvents were either purchased from Sigma Aldrich, or obtained from the University of Bristol's anhydrous Grubbs' type solvent stills. Reactions were monitored by TLC analysis, on aluminium-backed TLC plates, Silica Gel 60 F254 (Merck). TLCs were visualized with either UV light (λ = 254 nm) or charring with 5% H_2SO_4 in EtOH solution. Solutions were concentrated under reduced pressure using both a Büchi rotary evaporator at a pressure of either 15 mmHg (diaphragm pump) or 0.1 mmHg (oil pump), as appropriate, and a high vacuum line at room temperature. Reactions that required heating were carried out on a Drysyn heating block.

^{1}H NMR and ^{13}C NMR spectra were measured in the solvent stated at 400, 500 or 600 MHz. Chemical shifts are quoted in parts per million from residual solvent peak ($CDCl_3$: ^{1}H - 7.26 ppm and ^{13}C - 77.16 ppm) and coupling constants (*J*) given in Hertz. ^{1}H shifts are given to 2.d.p and ^{13}C shifts are given to 1 d.p. Multiplicities are abbreviated as br (broad), s (singlet), d (doublet), t (triplet), q (quartet), m (multiplet) or combinations thereof. Coupling constants (*J* values) are quoted to the nearest 0.1 Hz. Where a signal in the ^{1}H or ^{13}C NMR cannot be fully assigned as much information as possible is given for assignment. If signals overlap, both signal assignments are included in the same bracket (eg. C-3 and C-4) or (2C, CH_2Ph). Where ^{1}H NMR data for a mixture of anomers has been measured, separate 1H assignments are given. Where signals in the ^{13}C NMR spectrum are not assignable due to spectral overlap, the multiplicity of the carbon is given (e.g. CH_2). Structural assignments were made with additional information from 2D COSY, HSQC and HMBC experiments. Mass spectrometry was carried out by the University of Bristol Mass Spectrometry service on a micrOTOF II (ESI) spectrometer, with the HRMS mode incorporating a lock-in mass injected midway through the run (sodium formate). IR spectra were recorded on a Perkin Elmer Spectrum One FT-IR spectrometer fitted with a universal ATR accessory.

Synthesis of the Starting materials

Synthesis of the catalysts

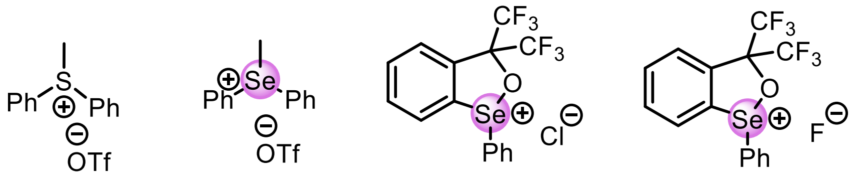

1a **1b** **1c** **1d**

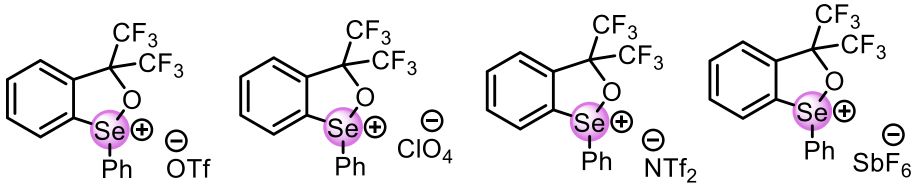

1e **1f** **1g** **1h**

1a is commercially available **1b**, **1c**, **1e**, **1f** and **1h** were prepared by reported literature procedure.

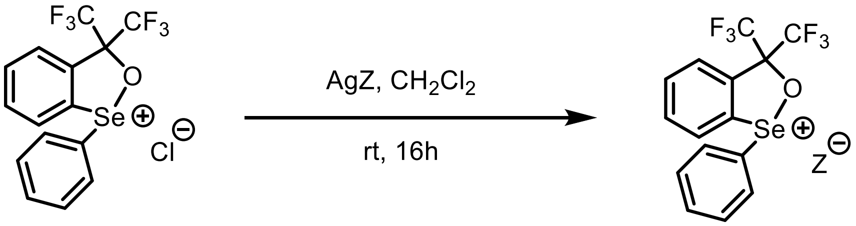

1c

1d-1h

Z= ClO_4, OTf, F, NTf_2, SbF_6

Scheme S1. General procedure for the synthesis **1d** and **1g**

Silver salt **AgZ** (0.2 mmol) was added to a solution of **1c** (0.2 mmol) in dry CH_2Cl_2 (2 mL). The reaction was stirred at room temperature overnight, after which silver chloride was removed by filtration, the filtrates concentrated under reduced pressure and the residues recrystallized from CH_2Cl_2-Hexane to afford catalysts **1d** -**1h.**

Phenyl-3,3-bis(trifluoromethyl)-1,3-dihydrobenzo[c][1,2]oxaselenol-1-ium fluoride **(1d)**

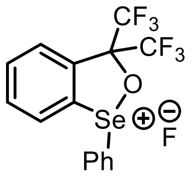

Yield: 74 % **^{1}H NMR (400 MHz, $CDCl_3$) δ** 8.98 (d, *J* = 8.2 Hz, 1H), 7.93 (t, *J* = 7.0 Hz, 1H), 7.87 (t, *J* = 7.5 Hz, 1H), 7.82 (d, *J* = 8.0 Hz, 1H), 7.53 (d, *J* = 7.8 Hz, 1H). **^{13}C NMR (101 MHz, $CDCl_3$) δ** 146.3, 134.9, 133.7, 133.4, 132.9, 131.8, 131.5, 129.7, 127.5, 126.9. **^{19}F NMR (471 MHz, $CDCl_3$) δ** -74.69 (d, *J* = 9.0 Hz), -74.88 (q, *J* = 9.0 Hz), -76.30 (q, *J* = 8.9 Hz), -76.48 (q, *J* = 9.1 Hz).

Phenyl-3,3-bis(trifluoromethyl)-1,3-dihydrobenzo[c][1,2]oxaselenol-1-ium bistriflimide **(1g)**

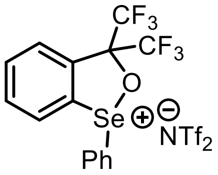

Yield: 85 % **^{1}H NMR (400 MHz, $CDCl_3$) δ** 8.55 (br s, 1H), 8.02 (d, J = 35.8 Hz, 2H), 7.95- 7.87 (m, 1H), 7.73- 7.63 (m, 2H), 7.61- 7.52 (m, 5H). **^{13}C NMR (101 MHz, $CDCl_3$) δ** 134.9, 133.9, 133.7, 133.7, 133.4, 133.3, 132.7, 131.7, 131.6, 130.7, 128.7, 127.6, 122.7, 120.5, 118.4, 116.3.**^{19}F NMR (471 MHz, $CDCl_3$) δ** -74.0, -75.7, -78.6.

Synthesis of the acceptors

List of glycosyl acceptors used for the synthesis of 2-deoxy glycosides.

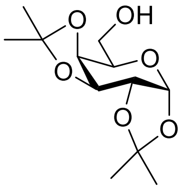

3a

CH_3OH

3b

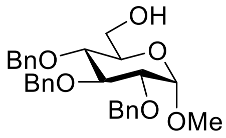

3c

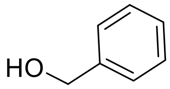

3d

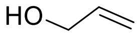

3e

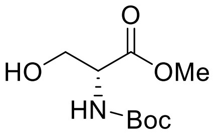

3f

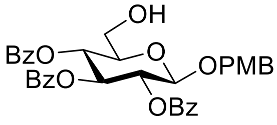

3g

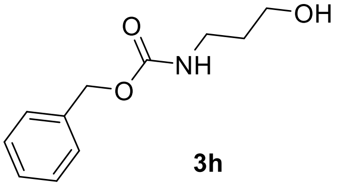

3h

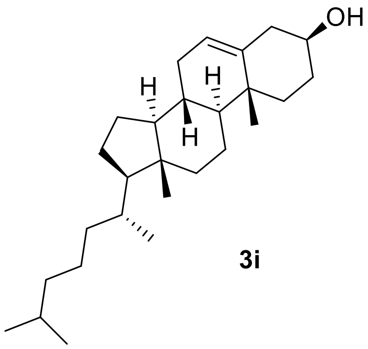

3i

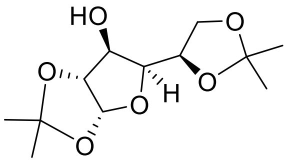

3j

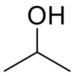

3k

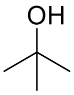

3l

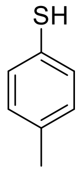

3m

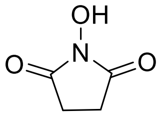

3n

CD_3OH

3o

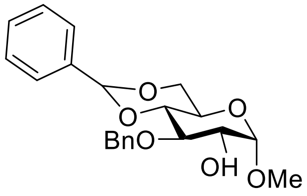

3p

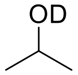

d- 3k

3a, **3b**, **3d**, **3e**, **3f**, **3h**, **3i**, **3j**, **3k**, **3l**, **3m**, **3n** , **3o** and **d-3k** were commercially available**. 3c** and **3g** were synthesized according to literature procedure.[2]

Synthesis of 2-deoxy hemiacetals 2a- 2h

List of hemiacetals used.

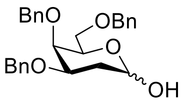

2a

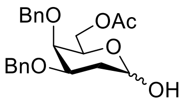

2b

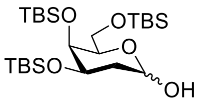

2c

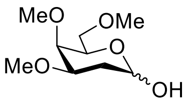

2d

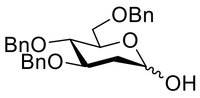

2e

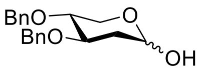

2f

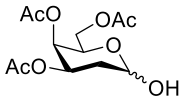

2g

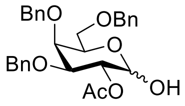

2h

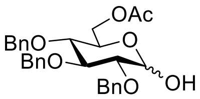

2i

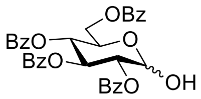

2j

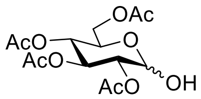

2k

Hemiacetals **2a-2k** were synthesised by a literature procedure.[2]

General procedure A: Se-catalysed glycosylation reactions

Hemiacetal donor and acceptor were added to microwave tubes/ RBF and placed under N_2. The vessels were sealed and anhydrous DCM was added to dissolve the substrates. **1e** was added, and the reactions were stirred until the reaction was deemed to be complete by TLC, after which the reaction mixtures were concentrated. The crude products were purified using silica gel flash column chromatography.

Reaction optimisation

Reactions to optimise catalyst, solvent and catalyst loading were performed according to general procedure A, with reactions to optimise temperature including an additional heating/cooling step to either 40 °C using a drysyn or 0 °C using an ice bath. Crude ^{1}H NMR spectra were used to calculate conversions and could be compared to previously reported data on compound **4a**.

Triflate catalyst **1e** was selected as the optimal catalyst for this work, producing the highest conversions (Table 1). Catalyst loading was investigated, (Table S3) and it was found that

there was not a significant difference between 5- and 10 mol %, so 5 mol % was selected. Dichloromethane was found to be the best performing solvent (Table S1), and the temperature dependence of itself as well as dichloroethane was assessed (Table S2). As a control experiment to probe counter anion effects, reactions using an increased amount of catalyst **1c** (Cl^-) and **1d** (F^-) (30 mol%) were also screened which afforded low or no yields, respectively, and were not investigated further.

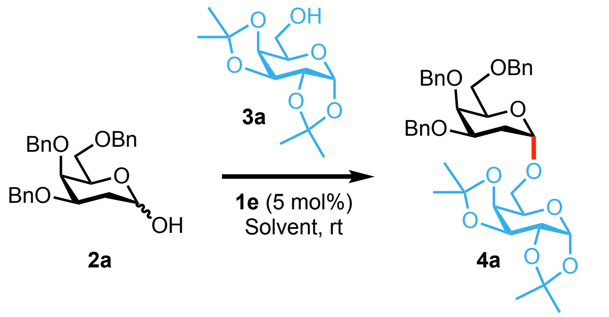

Table S1: Solvent screen with catalyst **1e**.

Entry	Solvent	Time, h	Yield[a],%
**1**	DCM	24	83
**2**	DMF	24	0
**3**	THF	24	69
**4**	EtOAc	24	67
**5**	DCE	24	69
**6**	MeCN	24	45
**7**	Toluene	24	60
**8**	DMSO	24	33
**9**	$CDCl_3$	24	4

[a]Determined by crude ^1H NMR.

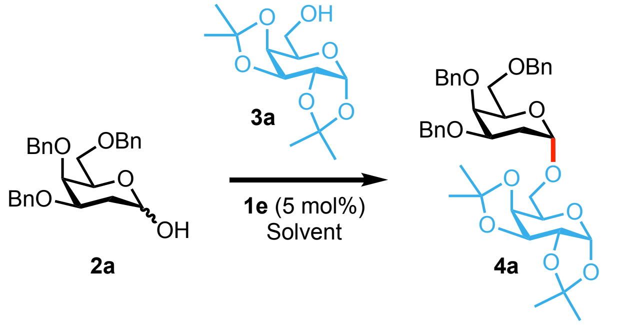

Table S2: Temperature screen using DCM and DCE

Entry	Solvent	Temperature, °C	Time, h	Yield[a], %
**1**	DCM	0 °C	3	9

			6	9
			24	25
**2**	DCM	40 °C	3	44
			6	50
			24	74
**3**	DCE	25 °C	3	33
			6	36
			24	63
**4**	DCE	40 °C	3	-
			6	58
			24	63

[a]Determined by crude ^{1}H NMR

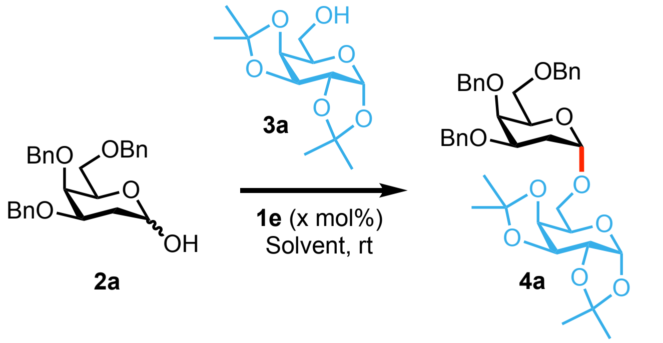

Table S3: Catalyst loading of **1e**.

Entry	Catalyst loading, mol %	Temperature, °C	Time	Yield[a], %
**1**	1	25	6	15
			24	25
**2**	1	40	6	40
			24	74
**3**	10	25	6	44
			24	61
**4**	10	40	6	58
			24	70

[a]Determined by crude ^{1}H NMR

Synthesis of 2-deoxy glycosides

6-*O*-(2-deoxy-3,4,6-tri-*O*-benzyl-α-D-galactopyranosyl)-1,2,3,4-di-O-isopropylidene-α-D-galactopyranoside (**4a**)

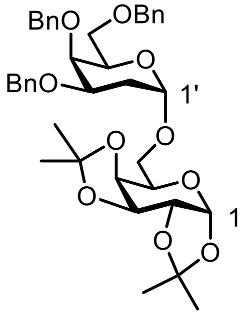

Prepared according to general glycosylation procedure A from hemiacetal donor **2a** (30 mg, 69 μmol), acceptor **3a** (14 mg, 53 μmol) and catalyst **1e** (1.5 mg, 2.7 μmol). After 6 hours and following purification using column chromatography (12:1 to 4:1 Hexane:EtOAc), **4a** was afforded as a colourless oil (25 mg, 70%, α only).

^{1}H NMR (400 MHz, $CDCl_3$) δ 7.39–7.20 (m, 15H, ArH), 5.54 (d, *J* = 5.1 Hz, 1H, H-1), 5.05 (d, *J* = 2.6 Hz, 1H, H-1'), 4.91 (d, *J* = 10.8 Hz, 1H, CH_2Ph), 4.72–4.61 (m, 4H, H-3, 3 x CH_2Ph), 4.59–4.52 (m, 2H, 2 x CH_2Ph), 4.34 (dd, *J* = 5.0, 2.4 Hz, 1H, H-2), 4.25 (dd, *J* = 7.9, 1.9 Hz, 1H, H-4), 4.06–3.96 (m, 2H),4.03 (ddd, *J* = 9.1, 6.7, 4.7 Hz, 1H, H-3'), 3.99–3.96 (m, 1H, H-5), 3.85–3.75 (m, 3 H, H-5', H-6a', H-6a), 3.72–3.66 (m, 3H, H-4', H-6b', H-6b), 2.36 (ddd, *J* = 12.6, 5.1, 1.2 Hz, 1H, H-2'), 1.76 (ddd, *J* = 13.0, 11.5, 3.7 Hz, 1H, H-2' ax), 1.55 (m, 3H, CH_3), 1.47 (m, 3H, CH_3), 1.37 (m, 3H, CH_3), 1.36 (m, 3H, CH_3). **^{13}C NMR (101 MHz, $CDCl_3$) δ** 138.8 (Ar C), 138.6 (Ar C), 138.3 (Ar C), 138.2 (Ar C), 128.4 (Ar CH), 128.33 (Ar CH), 128.31 (Ar CH), 127.95 (Ar CH), 127.91 (Ar CH), 127.60 (Ar CH), 127.59 (Ar CH), 127.56 (Ar CH), 127.49 (Ar CH), 109.3 ($C(CH_3)_2$), 108.5 ($C(CH_3)_2$), 97.3 (C-1'), 96.3 (C-1), 78.2 (C-4'), 77.6 (C-3'), 75.0 (CH_2Ph), 73.4 (CH_2Ph), 71.8 (CH_2Ph), 71.0 (C4 and C-5'), 70.7 (C-2 and C-3), 68.8 (C-6), 65.7 (C-5), 65.4 (C-6'), 35.4 (C-2'), 26.2 ($C(CH_3)$), 26.0 ($C(CH_3)$), 24,9 ($C(CH_3)$), 24,6 ($C(CH_3)$).
Spectroscopic data was in agreement with previously reported literature.[3]

Methyl 3,4,6-tri-*O*-benzyl-2-deoxy-a-D-galactopyranoside (**4b**)

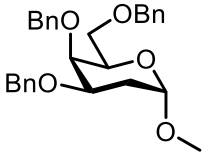

Prepared according to general glycosylation procedure A from hemiacetal **2a** (15 mg, 34.5 μmol), acceptor **3b** (1.1 μL, 26.6 μmol), catalyst **1e** (728 μg, 1.3 μmol). After 2 hours and following purification by column chromatography (8:2 Hexane:EtOAc), **4b** was obtained as a colourless oil (7.6 mg, 64%, α only). R_f = 0.58 (8:2 Hexane:EtOAc).

^{1}H NMR (500 MHz, CDCl$_3$) δ 7.35- 7.25 (m, 15H, Ar-H), 4.93 (d, *J* = 11.6, 1H, OCH_2Ar), 4.88 (d, $J_{1,2}$ = 2.8 Hz, 1H, H-1), 4.62 (d, *J* = 11.6 Hz, 1H, OCH_2Ar), 4.61 (s, 2H, OCH_2Ar), 4.52 (d, *J* = 11.8 Hz, 1H, OCH_2Ar), 4.43 (d, *J* = 11.8 Hz, 1H, OCH_2Ar), 3.95-3.85 (m, 3H, H-3, H-4, H-5), 3.59 (dd, $J_{6a,6b}$ = 6.4, $J_{6,5}$ = 3.0 Hz, 2H, H-6a, H-6b), 3.32 (s, 3H, OCH_3), 2.23 (ddd, $J_{2a,2b}$ = 12.6, $J_{2a,3}$ = 8.3, $J_{2a,1}$ = 2.5 Hz, 1H, H-2a), 1.99 (ddt, $J_{2b,2a}$ = 12.6, $J_{2b,3}$ = 4.4, $J_{2b,1}$ = 1.4 Hz, 1H, H-2b).**^{13}C NMR (126 MHz, CDCl$_3$) δ** 139.0 (4° C), 138.7 (4° C), 128.3 (4° C), 128.6 (Ar-CH), 128.5 (Ar-CH), 128.4 (Ar-CH), 128.3 (Ar-CH), 127.9 (Ar-CH), 127.8 (Ar-CH), 127.7 (Ar-CH), 127.4 (Ar-CH), 99.1 (C-1), 74.9 (C-3), 74.4 (CH_2Ph), 73.6 (CH_2Ph),73.2 (C-4), 70.6 (CH_2Ph), 69.9 (C-5), 69.8 (C-6), 55.0 (OCH_3), 31.3 (C-2).

HRMS: Calculated for $C_{28}H_{32}O_5$ $[M+Na]^+$ 471.2142 observed 471.2164.

Methyl 2,3,4-tri-*O*-benzyl-6-O-(2-deoxy-3,4,6-tri-O-benzyl-α-D-galactopyranosyl)-α-D-galactopyranoside (**4c**)

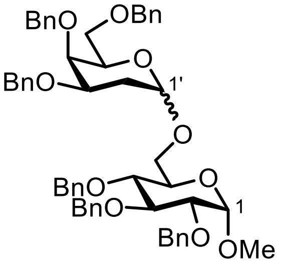

Prepared according to general glycosylation procedure A from hemiacetal donor **2a** (30 mg, 69 μmol), acceptor **3c** (25 mg, 53 μmol) and catalyst **1e** (1.5 mg, 2.7 μmol). After 6 hours and following purification using column chromatography (12:1 to 4:1 Hexane:EtOAc), **4c** was afforded as a colourless oil (31 mg, 68%, 10:1 α:β).

^{1}H NMR (400 MHz, Chloroform-d) δ 7.44–7.20 (m, 30 H, ArH), 5.06 (br s, 1H, H-1’), 5.05 (d, *J* = 10.9 Hz, 1H, CH_2Ph), 4.98 (d, *J* = 11.2 Hz, 1H, CH_2Ph), 4.94 (dd, *J* = 11.0 Hz, 1H, CH_2Ph), 4.88–4.84 (m, 2H, 2 x CH_2Ph), 4.76–4.60 (m, 7H, H-1, 6 x CH_2Ph), 4.55 (dd, *J* = 11.0 Hz, 1H, CH_2Ph), 4.47 (dd, *J* = 12.1 Hz, 1H, CH_2Ph), 4.06 (t, *J* = 9.3 Hz, 1H, H-3), 4.02–3.97 (m, 1H, H-3’), 3.88 (dd, *J* = 11.3, 4.4 Hz, 1H, H-6a’), 3.80 (ddd, *J* = 10.0, 4.3, 1.5 Hz, 1H, H-5’), 3.74 (ddd, *J* = 9.9, 3.3, 1.8 Hz, 1H, H-5), 3.69–3.54 (m, 3H, H-4, H6b’, H-6a), 3.60–3.54 (m, 3H, H-2, H-4’, H-6b), 3.41 (s, 3H, CH_3), 2.36 (ddd, *J* = 13.0, 5.0, 1.1 Hz, 1H, H-2’b),2.01 (ddd, *J* = 12.9, 11.6 Hz, 3.5 Hz, 1H, H-2’a).**^{13}C NMR (101 MHz, CDCl$_3$) δ** 128.48 (Ar CH), 128.42 (Ar CH),

128.37 (Ar CH), 128.34 (Ar CH), 128.30 (Ar CH), 128.2 (Ar CH), 128.1 (Ar CH), 128.0 (Ar CH), 127.92 (Ar CH), 127.87 (Ar CH), 127.78 (Ar CH), 127.74 (Ar CH), 127.65 (Ar CH), 127.57 (Ar CH), 127.54 (Ar CH), 127.4 (Ar CH), 97.9 (C-1), 97.8 (C-1'), 82.2 (C-3), 80.0 (C-4'), 78.1 (C-4), 77.8 (C-2), 77.2 (C-3'), 75.8 (CH_2Ph), 74.9 (CH_2Ph), 74.8 (CH_2Ph), 73.4 (CH_2Ph), 73.3 (CH_2Ph), 71.7 (CH_2Ph), 70.9 (C-5), 69.8 (C-5'), 68.7 (C-6), 65.7 (C-6'), 55.1 (OCH_3), 35.3 (C-2').
Spectroscopic data was in agreement with previously reported literature.[3]

Methyl 2,3,4-tri-*O*-benzyl-6-O-(3,4-di-*O*-benzyl-6-*O*-acetyl-2-deoxy-α-D-lyxo-hexapyranosyl)-α-D glucopyranoside (**4d**)

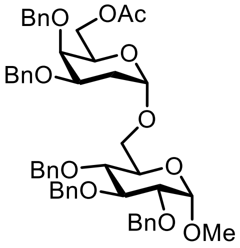

Prepared according to general glycosylation procedure A from hemiacetal donor **2b** (100 mg, 259 μmol), acceptor **3c** (100 mg, 215 μmol) and catalyst **1e** (8 mg, 13.5 μmol). After 8 hours and following purification using column chromatography (12:1 to 4:1 Hexane:EtOAc), **4d** was afforded as a white solid (147 mg, 68% only α).
^{1}H NMR (400 MHz, $CDCl_3$) δ 7.41 – 7.18 (m, 25H, Ar*H*, 5.03 (d, *J* = 3.4 Hz, 1H, H-1'), 4.99 (d, *J* = 10.8 Hz, 1H, OCH*H*Ph), 4.94 (d, *J* = 11.7 Hz, 1H, OCH*H*Ph), 4.86 (d, *J* = 11.0 Hz, 1H, OCH*H*Ph), 4.83 – 4.76 (m, 1H, OCH*H*Ph), 4.69 (d, *J* = 12.2 Hz, 1H, OCH*H*Ph), 4.66 – 4.55 (m, 3H, H-1, OCH_2Ph), 4.48 (d, *J* = 11.1 Hz, 1H, OCH*H*Ph), 4.11 – 3.93 (m, 3H, H-6a', H-6b', H-3), 3.89 – 3.81 (m, 1H, H-3'), 3.80 – 3.68 (m, 3H, H-4', H-6a, H-2), 3.61 (t, *J* = 5.8 Hz, 1H, H-5'), 3.51 (dd, *J* = 9.6, 3.6 Hz, 1H, H-2), 3.47 – 3.39 (m, 1H, H-4), 3.36 – 3.27 S, 3H, OCH_3), 2.25 – 2.14 (m, 1H, H-2a'), 2.03 (dd, *J* = 12.6, 4.5 Hz, 1H, H-2b'), 1.85 (s, 3H. $COCH_3$). **^{13}C NMR (101 MHz, $CDCl_3$) δ** 170.6 (C=O), 138.8, 138.6, 138.4,138.3, 128.6, 128.6, 128.5, 128.5, 128.5, 128.2, 128.1, 127.9, 127.8, 127.6, 127.6 (Ar *C*), 98.2 (C-1'), 97.9 (C-1), 82.3 (C-3), 80.1 (C-2), 78.1, 77.4, 76.0, 75.1, 74.1 (C-3'), 73.4, 72.7, 70.5, 69.3 (C-4'), 69.1 (C-5), 65.7 (C-6), 63.8 (C-6'), 55.2 (OCH_3), 30.8 (C-2'), 20.9 ($COCH_3$).
Spectroscopic data was in agreement with previously reported literature.[3]

Benzyl 2-deoxy-3,4,6-tri-*O*-benzyl-α-D-galactopyranoside (**4e**)

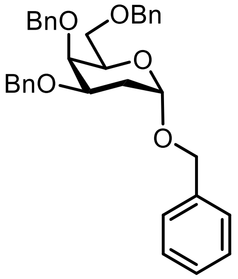

Prepared according to general glycosylation procedure A from hemiacetal donor **2a** (30 mg, 69 µmol), acceptor **3d** (5.5 µL, 53 µmol) and catalyst **1e** (1.5 mg, 2.7 µmol). After 2 hours and following purification by column chromatography (8.5:1.5 Hexane:EtOAc), **4e** was obtained as a pale yellow oil (21.2 mg, 76%, α only).

^{1}H NMR (500 MHz, $CDCl_3$) δ 7.36-7.23 (m, 20H, Ar-H), 5.07 (d, 1H, $J_{1,2b}$= 3.5 Hz, H-1), 4.94 (d, *J* = 11.6 Hz, 1H, OCH_2Ar), 4.68 (d, *J* = 11.9 Hz, 1H, OCH_2Ar), 4.63 (d, *J* = 11.7 Hz, 1H, OCH_2Ar), 4.60 (s, 2H, OCH_2Ar), 4.52- 4.46 (m, 3H, OCH_2Ar), 4.01- 3.94 (m, 3H, H-3, H-4, H-5), 3.63-3.54 (m, 2H, H-6), 2.25 (td, $J_{2b,2a}$ = 12.4, $J_{2b,3}$ = 3.7 Hz, 1H, H-2b), 2.05 (dd, $J_{2a,2b}$ = 12.4 Hz, $J_{2a,3}$ = 4.6 Hz, 1H, H-2a).**^{13}C NMR (101 MHz, $CDCl_3$) δ** 138.9 (4 °C, Ar-C), 138.5 (4 °C, Ar-C), 138.1 (4 °C, Ar-C), 137.8 (4 °C, Ar-C), 128.4 (CH, Ar-C), 128.3 (CH, Ar-C), 128.3 (CH, Ar-C), 128.2 (CH, Ar-C), 128.1 (CH, Ar-C), 127.9 (CH, Ar-C), 127.8 (CH, Ar-C), 127.7 (CH, Ar-C), 127.6 (CH, Ar-C), 127.5 (CH, Ar-C), 127.5 (CH, Ar-C), 127.3 (CH, Ar-C), 97.1 (C-1), 74.9 (C-3), 74.3 (OCH_2Ar), 73.5 (OCH_2Ar), 73.0 (C-4), 70.5 (OCH_2Ar), 70.1 (C-5), 69.6 (C-6), 68.9 (OCH_2Ar), 31.1 (C-2).

Spectroscopic data was in agreement with previously reported literature.[4]

Allyl 2-deoxy-3,4,6-tri-*O*-benzyl-α-D-galactopyranoside (**4f**)

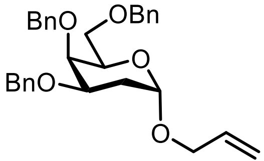

Prepared according to general glycosylation procedure A from hemiacetal donor **2a** (30 mg, 69 µmol), acceptor **3e** (3.6 µL, 53.1 µmol) and catalyst **1e** (1.5 mg, 2.7 µmol). After 3 hours and following purification using column chromatography (8.5:1.5 Hexane:EtOAc), **4f** was afforded as a colourless oil (20.5 mg, 82%, α only).

^{1}H NMR (500 MHz, $CDCl_3$) δ 7.37- 7.27 (m, 15H, Ar-H), 5.93- 5.87 (m, 1H, H-8), 5.25 (dd, $J_{9a,8}$= 17.2, $J_{9a,9b}$ = 1.7 Hz, 1H, H-9a), 5.16 (dd, $J_{9b,8}$= 10.4, $J_{9b,9a}$ = 1.7 Hz, 1H, H-9b), 5.03 (d, $J_{1,2b}$ = 3.7 Hz, 1H, H-1), 4.94 (d, *J* = 11.6 Hz, 1H, OCH_2Ar), 4.67 (d, *J*= 11.9 Hz, 1H, OCH_2Ar), 4.63 (d, *J* =

2.6 Hz, 1H, OCH$_2$Ar), 4.61- 4.60 (m, 1H, OCH$_2$Ar), 4.52- 4.42 (m, 2H, O*C*H_2Ar), 4.15- 4.11 (m, 1H, H-7a), 4.00- 3.92 (m , 4H, H-7b, H-3, H-4, H-5), 3.62-3.56 (m, 2H, H-6), 2.25 (tdd, $J_{2b,2a}$ = 12.3, $J_{2b,3}$ = 5.4, $J_{2b,1}$ = 3.7 Hz, 1H, H-2b), 2.04 (ddd, $J_{2a,2b}$ = 12.3, $J_{2a,3}$ = 10.2, $J_{2a,1}$ = 4.4 Hz, 1H, H-2a).**^{13}C NMR (126 MHz, CDCl$_3$) δ** 138.9 (4 °C, Ar-C), 138.6 (4 °C, Ar-C), 138.5 (4 °C, Ar-C), 134.3 (C-8), 128.4 (CH, Ar-C), 128.4 (CH, Ar-C), 128.3 (CH, Ar-C), 128.2 (CH, Ar-C), 127.9 (CH, Ar-C), 127.8 (CH, Ar-C), 127.7 (CH, Ar-C), 127.7 (CH, Ar-C), 127.6 (CH, Ar-C), 127.5 (CH, Ar-C), 127.5 (CH, Ar-C), 127.4 (CH, Ar-C), 127.3 (CH, Ar-C), 117.0 (C-9), 97.1 (C-1), 74.9 (C-3), 74.3 (O*C*H$_2$Ar), 73.5 (O*C*H$_2$Ar), 73.0 (C-4), 69.9 (C-5), 69.6 (C-6), 68.9 (O*C*H$_2$Ar), 67.9 (C-7), 31.17 (C-2).
Spectroscopic data was in agreement with previously reported literature.[5]

O-(3,4,6-Tri-*O*-benzyl-2-deoxy-D-galactopyranosyl)-*N*-[(carboxybenzyl)]-l-serine methyl ester **(4g)**

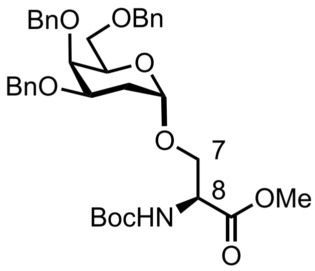

Prepared according to general glycosylation procedure A from hemiacetal **2a** (30 mg, 69 μmol), acceptor **3f** (11.6 mg, 53.1 μmol), catalyst **1e** (1.5 mg, 2.7 μmol). After 24 hours and following purification by column chromatography (8:2 Hexane:EtOAc), **4g** was obtained as a colourless oil (14.8 mg, 44 %, α only). R_f = 0.36 (8:2 Hexane:EtOAc). A 72% yield was achieved with no change in stereoselectivity when **3f** and **1e** were pre-stirred for 3h before the addition of the hemiacetal **2a**.
^{1}H NMR (400 MHz, CDCl$_3$) δ 7.46- 7.20 (m, 15H, Ar-H), 5.34 (d, $J_{7a,8}$ = 9.0 Hz, 1H, NH), 4.94 (d, $J_{1,2b}$ = 3.5 Hz, 1H, H-1), 4.91 (d, *J* = 11.6 Hz, 1H, OCH$_2$Ar), 4.59 (m, 3H, 2 x OCH$_2$Ar, H-5), 4.46 (m, 3H, H-8, OCH$_2$Ar), 4.06 (dd, $J_{7a,7b}$ = 9.8, $J_{7a,8}$ = 3.3 Hz, 1H, H-7a), 3.88 (br. s, 1H, H-4), 3.80 (ddd, $J_{3,2a}$ = 12.4, $J_{3,2b}$ = 3.7, $J_{3,4}$ = 2.4 Hz, 1H, H-3), 3.71 (t, $J_{5,4}$ = $J_{5,6a}$ = 6.4 Hz, 1H, H-5), 3.66 (s, 3H), 3.62- 3.51 (m, 3H, H-7a, H-6a and H6b), 2.21 (td, $J_{2b,2a}$ = 12.4, $J_{2b,1}$ = $J_{2b,3}$ = 3.7 Hz, 1H, H-2b), 1.96 (dd, $J_{2a,2b}$ = $J_{2a,3}$ = 12.4, $J_{2a,1}$ = 4.5 Hz, 1H, H-2a), 1.47 (s, 9H, C(CH$_3$)$_3$). **^{13}C NMR (126 MHz, CDCl$_3$) δ** 171.1 (C=O), 155.6 (4° C, *C*(CH$_3$)$_3$), 138.9 (4° C, Ar-C), 138.5 (4° C, Ar-C), 138.2 (4° C, Ar-C), 128.6 (Ar-C), 128.5 (Ar-C), 128.5 (Ar-C), 128.4 (Ar-C), 127.9 (Ar-C), 127.8 (Ar-C), 127.7 (Ar-C), 127.7 (Ar-C), 127.6 (Ar-C), 98.0 (C-1), 74.6 (C-3), 74.4 (OCH$_2$Ar), 73.6 (OCH$_2$Ar), 72.9 (C-4), 70.7 (OCH$_2$Ar), 70.4 (C-5), 69.2 (C-6), 67.5 (C-7), 53.7 (C-8), 52.4 (CH3), 31.0 (C-2), 28.5 (3C, C(CH$_3$)$_3$).
Spectroscopic data was in agreement with previously reported literature.[4]

Methyl 4-*O*-(2-deoxy-3,4,6-tri-*O*-benzyl-α-D-galactopyranosyl)-6-*O*-triisopropylsilyl-2,3-di-*O*-benzyl-a-D-glucopyranoside (**4h**)

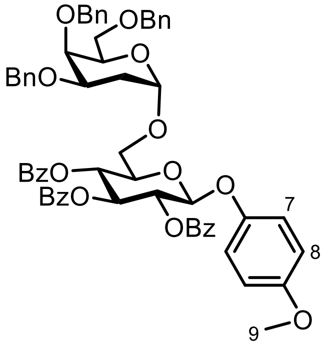

Prepared according to general glycosylation procedure A from hemiacetal donor **2a** (30 mg, 69 µmol), acceptor **3g** (31.8 mg, 53 µmol) and catalyst **1e** (1.5 mg, 2.7 µmol). After 24 hours and following purification using column chromatography (7:3 Hexane:EtOAc), **4h** was afforded as a colourless oil (28.0 mg, 52 %, α only).

^{1}H NMR (500 MHz, $CDCl_3$) δ 7.99 (d, *J* = 8.1 Hz, 2H, Ar H), 7.94 (d, *J* = 7.7 Hz, 1H, Ar H), 7.87 (d, *J* = 7.8 Hz, 1H, Ar H), 7.57- 7.44 (m, 4H, Ar H), 7.43- 7.21 (m, 20H, Ar H), 6.97 (d, $J_{8,7}$ = 9.0 Hz, 2H, H-8), 6.76 (d, $J_{7,8}$ = 9.0 Hz, 2H, H-7), 5.93 (t, $J_{3,2}$ = $J_{3,4}$ = 9.6 Hz, 1H, H-3), 5.75 (t, $J_{2,3}$ = $J_{2,1}$ = 8.5 Hz, 1H, H-2), 5.66 (t, $J_{4,3}$ = $J_{4,5}$ = 9.6 Hz, 1H, H-4), 5.25 (d, $J_{1,2}$ = 8.5 Hz, 1H, H-1), 5.01 (d, $J_{1',2'b}$ = 3.5 Hz, 1H, H-1'), 4.89 (d, *J* = 11.7 Hz, 1H, OCH_2Ar), 4.57 (d, *J* = 11.7 Hz, 1H, OCH_2Ar), 4.52 (s, 2H, OCH_2Ar), 4.38 (d, *J* = 12.0 Hz, 1H, OCH_2Ar), 4.31 (d, *J* = 12.0 Hz, 1H, OCH_2Ar), 4.08 (dt, $J_{5,4}$ = 9.6, $J_{5,6b}$ = 4.5 Hz, 1H, H-5), 3.93 (dd, $J_{6a,6b}$ = 11.0, $J_{6a,5}$ = 5.3 Hz, 1H, H-6a), 3.90- 3.84 (m, 2H, H-3', H-4'), 3.81- 3.76 (m, 1H, H-5'), 3.75- 3.64 (m, 4H, H-9, H-6b), 3.51- 3.42 (m, 2H, H-6'), 2.17 (td, $J_{2'b,2'a}$ = 12.5, $J_{2'b,3'}$ = 3.5 Hz, 1H, H-2'b), 1.97 (dd, $J_{2'a,2'b}$ = 12.7, $J_{2'a,3}$ = 4.7 Hz, 1H, H-2'a). **^{13}C NMR (126 MHz, $CDCl_3$) δ** 165.9 (C=O), 165.2 (2, C=O), 155.7 (4 °C, Ar-C), 151.1 (4 °C, Ar-C), 138.9 (4 °C, Ar-C), 138.6 (4 °C, Ar-C), 133.4 (Ar-C), 133.3 (Ar-C), 133.2 (Ar-C), 129.8 (Ar-C), 129.3 (Ar-C), 128.3 (Ar-C), 127.5 (Ar-C), 127.3 (Ar-C), 118.9 (C-8), 114.5 (C-7), 100.7 (C-1), 98.2 (C-1'), 74.8 (C-4'), 74.3 (OCH_2Ar), 73.2 (C-5), 73.2 (C-5'), 73.1 (C-3), 73.0 (OCH_2Ar), 71.8 (C-2), 70.6 (C-4), 70.5 (OCH_2Ar), 69.8 (C-3'), 69.7 (C-), 69.5 (C-6'), 66.2 (C-6), 55.6 (C-9), 30.8 (C-2').

HRMS: Calculated for $C_{61}H_{58}O_{14}$ 1037.3719, Observed 1037.3698

3-(*N*-benzoyloxycarbonyl) aminopropyl-3,4,6-tri-*O*-benzyl-2-deoxy-D-galactopyranoside (**4i**)

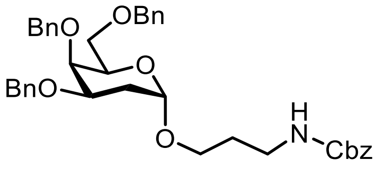

Prepared according to general glycosylation procedure A from hemiacetal donor **2a** (30 mg, 69 μmol), acceptor **3h** (11.1 mg, 53 μmol) and catalyst (1.5 mg, 2.7 μmol). After 24 hours and following purification using column chromatography (7:3 Hexane:EtOAc), **4i** was afforded as a colourless oil (29.6 mg, 89 %, 7:1 α:β).

^{1}H NMR (500 MHz, $CDCl_3$) δ 7.55- 7.13 (m, 20 H, Ar-H), 5.43 (br. s, 1H, NH), 5.10 (s, 2H, CH_2Ph), 4.99 (d, $J_{1,2b}$ = 3.6 Hz, 1H, H-1), 4.93 (d, J = 11.7 Hz, 1H, CH_2Ph), 4.68- 4.52 (m, 4H, 4 x CH_2Ph), 4.49- 4.40 (m, 1H, CH_2Ph), 3.94- 3.76 (m, 4H, H-3, H-4, H-5, H-7a), 3.58 (dd, $J_{6a,6b}$ = 9.6, $J_{6a,5}$ = 6.6 Hz, 1H, H-6a), 3.51- 3.45 (m, 2H, H-7b, H-6b), 3.43- 3.35 (m, 1H, H-9), 3.29- 3.21 (m, 1H, H-9), 2.25 (td, $J_{2b,2a}$ = 12.4, $J_{2b,1}$ = 3.8 Hz, 1H, H-2b), 2.01 (dd, $J_{2a,2b}$ = 12.4, $J_{2a,1}$ = 4.6, 1H, H-2a), 1.84- 1.76 (m, 2H, H-8).**^{13}C NMR (126 MHz, $CDCl_3$) δ** 156.5 (C=O), 138.8 (4° C, Ar-C), 138.5 (4° C, Ar-C), 137.9 (4° C, Ar-C), 136.7 (4° C, Ar-C), 128.5 (Ar C), 128.4 (Ar C), 128.3 (2C, Ar C), 128.2 (2C, Ar C), 128.1 (Ar C), 127.9 (Ar C), 127.7 (Ar C), 127.6 (Ar C), 127.5 (Ar C), 127.3 (Ar C), 97.9 (C-1), 75.0 (C-3), 74.2 (CH_2Ph), 73.4 (CH_2Ph), 73.4 (CH_2Ph), 73.0 (C-5), 70.6 (CH_2Ph), 70.4 (C-4), 70.0 (C-7), 66.6 (CH_2Ph), 65.1 (C-3), 38.7 (C-9), 31.1 (C-2), 29.4 (C-8). Spectroscopic data was in agreement with previously reported literature.[7]

Cholesteryl 2-deoxy-3,4,6-tri-*O*-benzyl-α-D-galactopyranoside (**4j**)

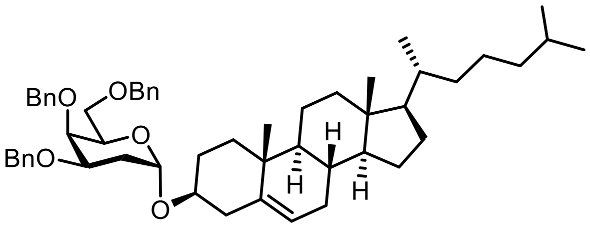

Prepared according to general glycosylation procedure A from hemiacetal donor **2a** (30 mg, 69 μmol), acceptor **3i** (20.5 mg, 53 μmol) and catalyst **1e** (1.5 mg, 2.7 μmol). After 24 hours and following purification using column chromatography (7:3 Hexane:EtOAc), **4j** was afforded as a white solid (39.7 mg, 93 %, 5:1 α:β).

^{1}H NMR (500 MHz, $CDCl_3$) δ 7.81- 6.99 (m, 15H, Ar H), 5.29 (d, J = 5.0 Hz, 1H, H-7), 5.18 (d, $J_{1,2}$ = 3.5 Hz, 1H, H-1), 4.97 (d, J = 11.6 Hz, 1H, OCH_2Ar), 4.70- 4.62 (m, 3H, OCH_2Ar), 4.54 (d, J = 11.7 Hz, 1H, OCH_2Ar), 4.47 (d, J = 11.7 Hz, 1H, OCH_2Ar), 4.07- 3.95 (m, 3H, H-3, H-4, H-5),

3.68- 3.63 (m, 2H, H-6), 3.53- 3.44 (m, 1H), 2.32 (m, 2H), 2.26 (dd, $J_{2b,2a}$ = 11.9, $J_{2b,3}$ = 3.7 Hz, H-2b), 2.09- 1.94 (m, 3H, H-2a), 1.93- 1.81 (m, 3H), 1.67- 1.26 (m, 14H), 1.23- 1.06 (m, 7H), 1.04- 1.01 (m, 6H), 0.95 (d, *J* = 6.5 Hz, 4H), 0.91 (d, *J* = 1.8 Hz, 3H), 0.90 (d, *J* = 1.8 Hz, 3H, CH_3). **^{13}C NMR (126 MHz, $CDCl_3$) δ** 140.9 (4° C), 139.0 (4° C, Ar C), 138.7 (4° C, Ar C), 138.2 (4° C, Ar C), 128.5 (CH, Ar C), 128.4 (CH, Ar C), 128.4 (CH, Ar C), 128.2 (CH, Ar C), 128.1 (CH, Ar C), 127.9 (CH, Ar C), 127.8 (CH, Ar C), 127.7 (CH, Ar C), 127.6 (CH, Ar C), 127.5 (2C, Ar C), 127.3 (CH, Ar C), 121.7 (C-7), 95.7 (C-1), 76.2, 75.1 (C-4/C-3), 74.3 (OCH_2Ar), 73.5 (OCH_2Ar), 73.2 (C-4/C-3), 70.5 (OCH_2Ar), 69.9 (C-5), 69.7 (C-6), 56.8 (CH_3), 56.2, 50.1 (CH_3), 42.4, 40.1, 39.8, 39.6, 37.1, 36.7, 36.2, 35.8 (CH), 32.0, 31.9 (CH), 31.7 (C-2), 28.3, 28.1, 27.9 (CH), 24.3, 23.9, 22.9, 22.6 (CH_3), 21.1, 19.4 (CH_3), 18.8 (CH_3).
Spectroscopic data was in agreement with previously reported literature.[9]

3-*O*-(2-deoxy-3,4,6-tri-O-benzyl-2-deoxy-α-D-galactopyranoside)-1,2:5,6-di-*O*-isopropylidene-α-D-glucofuranoside (**4k**)

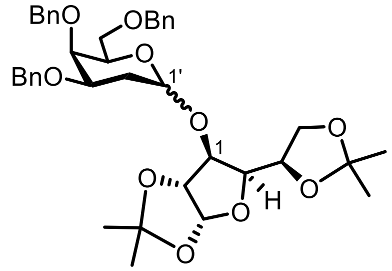

Prepared according to general glycosylation procedure A from hemiacetal donor **2a** (30 mg, 69 μmol), acceptor **3j** (14 mg, 53 μmol) and catalyst **1e** (1.5 mg, 2.7 μmol). After 24 hours and following purification using column chromatography (12:1 to 4:1 Hexane:EtOAc), **4k** was afforded as a colourless oil (16 mg, 43%, 4:1 α:β).
^{1}H NMR (500 MHz, $CDCl_3$): δ 7.36–7.26 (m, 15H, ArH), 5.83 (d, *J* = 3.6 Hz, 1H, H-1), 5.25 (d, *J* = 3.1 Hz, 1H, H-1'), 4.94 (d, *J* = 11.6 Hz, 1H, CH_2Ph), 4.64–4.49 (m, 4H, 4 x CH_2Ph), 4.44 (1 H, d, *J* = 11.8 Hz, 1H, CH_2Ph), 4.23 (d, *J* = 2.8 Hz, 1H, H-3), 4.20–4.15 (m, 1H, H-5'), 4.11–4.07 (m, 2H, H4, H-6'), 4.01–3.85 (m, 4H, H-3', H-4; H-5, H-6'), 3.63 (dd, 1H, *J* = 9.5, 6.2 Hz, H-6), 3.57 (dd, 1H, *J* = 9.5, 6.2 Hz, H-6), 2.24 (td, *J* = 12.4, 3.7 Hz, 1H, H-2' ax), 2.00 (dd, *J* = 12.7, 4.5 Hz, 1H, H-2' eq), 1.48 (s, 3H, CH_3), 1.40 (s, 3H, CH_3), 1.33 (s, 3H, CH_3), 1.21 (s, 3H, CH_3). **^{13}C NMR (126 MHz, $CDCl_3$): δ** 138.7 (Ar C), 138.3 (Ar C), 138.0 (Ar C), 128.44 (Ar CH), 128.40 (Ar CH), 128.24 (Ar CH), 128.10 (Ar CH), 127.8 (Ar CH), 127.63 (Ar CH), 127.60 (Ar CH), 127.6 (Ar CH), 127.3 (Ar CH), 111.8 ($C(CH_3)_2$), 109.1 ($C(CH_3)_2$), 105.3 (C-1), 99.5 (C-1'), 83.5 (C-2), 81.3 (C-4), 80.9 (C-3), 74.4 (C-3'), 74.3 (CH_2Ph), 73.6 (CH_2Ph), 73.1(C-4') , 72.6 (C-5'), 71.0 (CH), 70.5 (CH_2Ph), 70.0 (C-6), 67.6 (C-6'), 31.0 (C-2'), 26.9 (CH_3), 26.8 (CH_3), 26.1 (CH_3), 25.4 (CH_3).

Spectroscopic data was in agreement with previously reported literature.[7]

Isopropyl-2-deoxy-3,4,6-tri-*O*-benzyl-α-D-galactopyranoside (**4l**)

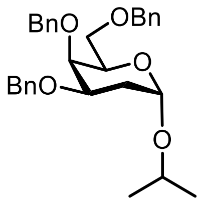

Prepared according to general glycosylation procedure A from hemiacetal donor **2a** (15 mg, 34.5 μmol), acceptor **3k** (2.0 μL, 26.6 μmol) and catalyst **1e** (727 μg, 1.3 μmol). After 3 hours and following purification using column chromatography (8:2 Hexane:EtOAc), **4l** was afforded as a colourless oil (8.7 mg, 69%, α only).

^{1}H NMR (500 MHz, $CDCl_3$) δ 7.40- 7.20 (m, 15H, Ar-H), 5.09 (d, $J_{1,2b}$ = 3.6 Hz, 1H, H-1), 4.93 (d, J = 11.6 Hz, OCH_2Ar), 4.65- 4.57 (m, 3H, OCH_2Ar), 4.50 (d, J = 11.8 Hz, 1H, OCH_2Ar), 4.50 (d, J = 11.8 Hz, 1H, OCH_2Ar), 4.01- 3.92 (m, 3H, H-3, H-4, H-5), 3.87 (p, $J_{7,8}$ = $J_{7,9}$ = 6.2 Hz, 1H, H-7), 3.62 (dd, $J_{6a,6b}$ = 9.4, $J_{6a,5}$ = 7.1 Hz, 1H, H-6a), 3.56 (dd, $J_{6b,6a}$ = 9.3, $J_{6b,5}$ = 5.9 Hz, 1H, H-6b), 2,23 (td, $J_{2b,2a}$ = 12.3, $J_{2b,3}$ = 3.9 Hz, 1H, H-2b), 1.94 (dd, $J_{2a,2b}$ = 12.3, $J_{2a,3}$ = 5.2 Hz, H-2a), 1.17 (d, $J_{8/9,7}$ = 6.2 Hz, 3H, H-8/9), 1.12 (d, $J_{9/8,7}$ = 6.2 Hz, 3H, H-9/8). **^{13}C NMR (126 MHz, $CDCl_3$) δ** 139.1 (4° C, Ar-C), 138.8 (4° C, Ar-C), 138.3 (4° C, Ar-C), 128.5 (Ar-C), 128.3 (Ar-C), 127.9 (Ar-C), 127.8 (Ar-C), 127.6 (Ar-C), 127.5 (Ar-C), 127.4 (Ar-C), 95.7 (C-1), 75.2 (C-4), 74.4 (OCH_2Ar), 73.6 (OCH_2Ar), 73.3 (C-3), 70.6 (OCH_2Ar), 69.9 (C-5), 69.7 (C-6), 68.5 (C-7), 31.8 (C-2), 23.5 (C-8/9), 21.5 (C-9/8).

Spectroscopic data was in agreement with previously reported literature.[6]

Tert-butyl-3,4,6-tri-*O*-benzyl-2-deoxy-D-galactopyranoside **4m**

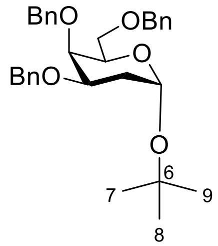

Prepared according to general glycosylation procedure A from hemiacetal donor **2a** (20 mg, 46 μmol), acceptor **3l** (2.6 mg, 35.4 μmol) and catalyst **1e** (970 μg, 1.3 μmol). After 6 hours and following purification using column chromatography (8:2 Hexane:EtOAc), **4m** was afforded as a colourless oil (7.3 mg, 41 %, α only).

^{1}H NMR (500 MHz, $CDCl_3$) δ 7.41- 7.22 (m, 15H, Ar-H), 5.29 (dd, $J_{1,2b}$ = 3.8 Hz, 1H, H-1), 4.94 (d, J = 11.6 Hz, 1H, CH_2Ar), 4.65- 4.58 (m, 3H, CH_2Ar), 4.50 (d, J = 11.8 Hz, 1H, CH_2Ar), 4.43 (d, J = 11.7 Hz, 1H, CH_2Ar), 4.09 (ddd, $J_{5,6a}$ = 7.7, $J_{5,6b}$ = 5.6, $J_{5,4}$ = 1.1 Hz, 1H, H-5), 4.03- 3.94 (m, 2H, H-3, H-4), 3.64 (dd, $J_{6a,6b}$ = 9.2, $J_{6a,5}$ = 7.7 Hz, 1H, H-6a), 3.51 (dd, $J_{6b,6a}$ = 9.2, $J_{6b,5}$ = 5.6 Hz, 1H, H-6b), 2.25 (td, $J_{2b,2a}$ = 11.9, $J_{2b,1}$ = 3.8 Hz, 1H, H-2b), 1.84 (ddt, $J_{2a,2b}$ = 12.0 Hz, $J_{2a,3}$ = 4.5 Hz, $J_{2a,1}$ = 1.5 Hz, 1H, H-2a), 1.22 (s, 9H, H-7, H-8, H-9). **^{13}C NMR (126 MHz, $CDCl_3$) δ** 139.3 (4° C), 138.9 (4° C), 138.4 (4° C), 128.5 (3C, Ar-C), 128.3 (2C, Ar-C), 128.3 (Ar-C), 127.8 (2C, Ar-C), 127.7 (Ar-C), 127.6 (Ar-C), 127.5 (Ar-C), 127.5 (Ar-C), 127.4 (3C, Ar-C), 92.5 (C-1), 75.3 (C-3), 74.5 (CH_2Ar), 74.4 (CH_2Ar), 73.4 (C-4), 70.5 (CH_2Ar), 69.6 (C-6), 69.4 (C-5), 32.9 (C-2), 28.8 (3C, C-7, C-8, C-9)
HRMS: Calculated for $C_{31}H_{38}O_5$ $[M+Na]^+$ 513.2611 observed 513.2588
Spectroscopic data was in agreement with previously reported literature.[4]

p-Tolyl 2-deoxy-3,4,6-tri-*O*-benzyl-α-D-thiogalactopyranoside (**4n**)

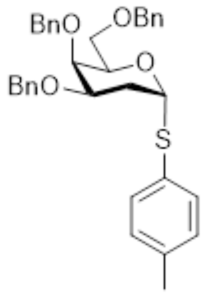

Prepared according to general glycosylation procedure A from hemiacetal donor **2a** (30 mg, 69 μmol), acceptor **3m** (6.6 mg, 53 μmol) and catalyst **1e** (1.5 mg, 2.7 μmol). After 2 hours and following purification using column chromatography (9:1 Hexane:EtOAc), **4n** was afforded as a colourless oil (6.2 mg, 21%). R_f (9:1 Hexane:EtOAc) = 0.53
^{1}H NMR (400 MHz, $CDCl_3$) δ 7.45- 7.43 (m, 2H, Ar-H), 7.35- 7.25 (m, 13H, Ar-H), 7.22- 7.19 (m, 2H), 7.10- 7.09 (m, 2H, Ar-H), 4.98 (d, $J_{1,2b}$ = 2.5 Hz, 1H, H-1), 4.84 (d, *J* = 12.4 Hz, 1H, CH_2Ph)), 4.64 (td, $J_{5,6b}$ = 6.0, $J_{5,4}$ = 1.5 Hz, 1H, H-5), 4.53 (d, *J* = 12.3 Hz, 1H, CH_2Ph), 4.43 (t, *J* = 8.8 Hz, 1H, CH_2Ph), 4.21 (d, *J* = 12.1 Hz, 1H, CH_2Ph), 3.59 (dd, $J_{6a,6b}$ = 10.0, $J_{6a,5}$ = 6.6 Hz, 1H, H-6a), 3.49 (dd, $J_{6b,6a}$ = 10.0, $J_{6b,5}$ = 6.0 Hz, 1H, H-6b), 3.43 (dt, $J_{3,2a}$ = 5.8, $J_{3,4}$ = 2.7 Hz, 1H, H-3), 3.40 (br d, $J_{4,3}$ = 2.7 Hz, 1H, H-4), 2.54 (ddd, $J_{2a,2b}$ = 14.6, $J_{2a,3}$ = 6.0, $J_{2a,1}$ = 4.0 Hz, 1H, H-2a), 2.37 (s, 3H, CH_3), 2.01 (ddt, $J_{2b,2a}$ = 14.6, $J_{2b,1}$ = 2.5, $J_{2b,3}$ = 1.2 Hz, H-2b).**^{13}C NMR (101 MHz, $CDCl_3$) δ** 138.4 (4° C, Ar C), 138.1 (4° C, Ar C), 137.8 (4° C, Ar C), 137.4 (4° C, Ar C), 132.9 (4° C, Ar C), 132.7 (Ar CH), 129.8 (Ar CH), 128.3 (Ar CH), 127.8 (Ar C), 127.6 (Ar CH), 127.5(Ar CH), 127.4 (Ar CH), 127.3 (Ar CH), 95.3 (CH, C-1), 73.3 (C-4), 73.2 (CH_2Ph), 71.6 (CH_2Ph), 70.1 (C-6), 68.7 (CH_2Ph), 65.3 (C-5), 43.1 (C-3), 30.2 (C-2), 21.2 (CH_3).
HRMS: Calculated for $C_{34}H_{36}O_4S$ $[M+Na]^+$ 563.2232, observed 563.2228

2,5-Pyrrolidinedione- 2-deoxy-3,4,6-tri-*O*-benzyl-a-D-galactopyranoside (**4o**)

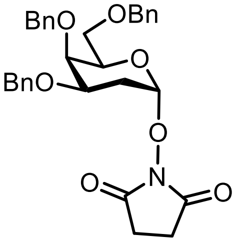

Prepared according to general glycosylation procedure A from hemiacetal donor **2a** (30 mg, 69 μmol), acceptor **3n** (6.1 mg, 53 μmol) and catalyst **1e** (1.5 mg, 2.7 μmol). After 24 hours and following purification using column chromatography (7:3 Hexane:EtOAc), **4o** was afforded as a pale yellow oil (21.5 mg, 76 %, α only).

^{1}H NMR (500 MHz, CDCl$_3$) δ 7.36- 7.26 (m, 15H, Ar-H), 5.58 (t, $J_{1,2}$ = 1.8 Hz, 1H, H-1), 4.93 (d, *J* = 11.4 Hz, 1H, OC*H*$_2$Ph), 4.66- 4.61 (m, 4H, H-3, 3 x OCH$_2$Ph), 4.52 (d, *J* = 11.8 Hz, 1H, OCH$_2$Ar), 4.47 (d, *J* = 11.8 Hz, 1H, OCH$_2$Ar), 4.06- 4.02 (m, 2H, H-4 and H-5), 3.60- 3.52 (m, 2H, H-6a, H-6b), 2.69- 2.57 (m, 4H, 2 x COCH$_2$), 2.36- 2.33 (m, 2H, H-2a, H-2b).**^{13}C NMR (126 MHz, CDCl$_3$) δ** 171.3 (2C, C=O), 138.7 (4° C, Ar-C) , 138.3 (4° C, Ar-C), 138.2 (4° C, Ar-C), 128.5 (Ar-CH), 128.4 (Ar-CH), 128.3 (Ar-CH), 128.2 (Ar-CH), 127.7 (Ar-CH), 127.7 (Ar-CH), 127.6 (Ar-CH), 127.5 (Ar-CH), 127.3 (Ar-CH), 102.6 (C-1), 74.5 (CH$_2$Ph), 73.6 (C-5), 73.2 (CH$_2$Ph), 72.6 (C-4), 71.9 (C-3), 70.6 (CH$_2$Ph), 70.0 (C-6), 28.7 (C-2), 25.5 (2 x CO*C*H$_2$). Spectroscopic data was in agreement with previously reported literature.[8]

Methyl 3-O-benzyl-2-O-(3,4,6-tri-O-benzyl-α-D-lyxo-hexapyranosyl)-4,6-O-benzylidene-α-D-glucopyranoside (**4p**)

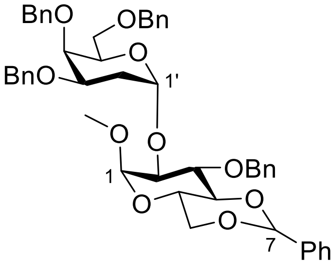

Prepared according to general glycosylation procedure A from hemiacetal donor **2a** (30 mg, 69 μmol), acceptor **3p** (19.8 mg, 53 μmol) and catalyst **1e** (1.5 mg, μmol). After 6 hours and following purification using column chromatography (8:2 Hexane:EtOAc), **4p** was afforded as a colourless oil (10.8 mg, 24 μmol, 45 %, α only).

^{1}H NMR (500 MHz, CDCl$_3$) δ 7.48- 7.44 (m, 2H, ArH), 7.39- 7.24 (m, 20 H, ArH), 7.19 (dd, *J* = 5.0, 1.9 Hz, 3H, ArH), 5.51 (s, 1H, H-7), 5.14 (d, *J* = 3.4 Hz, 1H, H-1'), 4.91- 4.84 (m, 3H, H-1, OC*H*$_2$Ph),

4.65 (d, *J* = 11.4 Hz, 1H, OC*H*$_2$Ph), 4.58 (d, *J* = 11.6 Hz, 1H, OC*H*$_2$Ph), 4.52 (d, *J* = 1.0 Hz, 2H, OC*H*$_2$Ph), 4.37 (m, 2H, OC*H*$_2$Ph), 4.27 (dd, *J* = 10.1, 4.8 Hz, 1H, H-6a), 4.19 (t, *J* = 6.3 Hz, 1H, H-5'), 3.94- 3.86 (m, 3HH-2, H-3, H-3'), 3.80 (td, *J* = 9.9, 4.8 Hz, 1H, H-5), 3.75 (m, 1H, H-4'), 3.69 (t, *J* = 10.3 Hz, 1H, H-6b), 3.60- 3.51 (m, 2H, H-6'a, H-4), 3.45 (dd, *J* = 10.0, 6.6 Hz, 1H, H-6'b), 3.43 (s, 3H), 2.25 (td, *J* = 12.5, 3.7 Hz, 1H, H-2'a), 2.07 (dd, *J* = 12.8, 4.6 Hz, 1H, H-2'b). **^{13}C NMR (126 MHz, CDCl$_3$) δ** 138.8 (4° C), 138.7 (4° C), 138.5 (4° C), 138.43 (4° C), 137.37 (4° C), 128.9 (CH), 128.4 (CH), 128.21 (CH), 128.19 (CH), 128.17 (CH), 128.15 (CH), 127.9 (CH), 127.6 (CH), 127.49 (CH), 127.47 (CH), 127.4 (CH), 127.3 (CH), 126.0 (CH), 101.2 (PhCO$_2$) , 97.2 (C-1), 94.0 (C-1'), 82.1 (C-4), 77.3 (C-3), 75.3 (CH$_2$Ph), 74.4, 74.3 (C-3', CH$_2$Ph), 72.9, 72.8 (C-2, C-4', CH$_2$Ph), 70.4 (CH$_2$Ph), 69.5, 69.4 (C-5', C-6'), 69.0 (C6), 62.4 (C-5), 55.2 (OCH$_3$), 30.9 (C-2').
Spectroscopic data is all in accordance with reported literature.{Balmond, 2012 #134}

6-*O*-(2-deoxy-3,4,6-tri-*O*-benzyl-α-D-glucopyranosyl)-1,2,3,4-di-*O*-isopropylidene-α-D-glucopyranoside (**5a**)

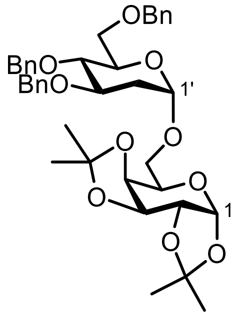

Prepared according to general glycosylation procedure A from hemiacetal donor **2e** (30 mg, 69 µmol), acceptor **3a** (14 mg, 53 µmol) and catalyst **1e** (1.5 mg, 2.7 µmol). After 3 hours and following purification using column chromatography (12:1 to 4:1 Hexane:EtOAc), **5a** was afforded as a colourless oil (23 mg, 63%, α only).
^{1}H NMR (400 MHz, CDCl$_3$) δ 7.39–7.20 (m, 15H, ArH), 5.54 (d, *J* = 5.1 Hz, 1H, H-1), 5.05 (d, *J* = 2.6 Hz, 1H, H-1'), 4.91 (d, *J* = 10.8 Hz, 1H, CH$_2$Ph), 4.72–4.61 (m, 4H, H-3, 3 x CH$_2$Ph), 4.59–4.52 (m, 2H, 2 x CH$_2$Ph), 4.34 (dd, *J* = 5.0, 2.4 Hz, 1H, H-2), 4.25 (dd, *J* = 7.9, 1.9 Hz, 1H, H-4), 4.03 (ddd, *J* = 9.1, 6.7, 4.7 Hz, 1H, H-3'), 3.99–3.96 (m, 1H, H-5), 3.85–3.75 (m, 3H, H-5', H-6a', H-6a), 3.72–3.66 (m, 3H, H-4', H-6b', H-6b), 2.36 (ddd, *J* = 12.6, 5.1, 1.2 Hz, 1H, H-2'), 1.76 (ddd, *J* = 13.0, 11.5, 3.7 Hz, 1H, H-2' ax), 1.55 (m, 3H, CH$_3$), 1.47 (m, 3H, CH$_3$), 1.37 (m, 3H, CH$_3$), 1.36 (m, 3H, CH$_3$). **^{13}C NMR (101 MHz, CDCl$_3$) δ** 138.8 (Ar C), 138.6 (Ar C), 138.3 (Ar C), 138.2 (Ar C), 128.4 (Ar CH), 128.3 (Ar CH), 128.3 (Ar CH), 128.0 (Ar CH), 127.9 (Ar CH), 127.6 (Ar CH), 127.6 (Ar CH), 127.6 (Ar CH), 127.5 (Ar CH), 109.3 (C(CH$_3$)$_2$), 108.5 (C(CH$_3$)$_2$), 97.3 (C-1'), 96.3 (C-1), 78.2 (C-4'), 77.6 (C-3'), 75.0 (CH$_2$Ph), 73.4 (CH$_2$Ph), 71.8 (CH$_2$Ph), 71.0 (C4 and C-5'), 70.7 (C-2 and C-3), 68.8 (C-6), 65.7 (C-5), 65.4 (C-6'), 35.4 (C-2'), 26.2 (C(CH$_3$)), 26.0 (C(CH$_3$)), 24.9 (C(CH$_3$)), 24.6 (C(CH$_3$)).

Spectroscopic data was in agreement with previously reported literature.[9]

Methyl 3,4,6-tri-*O*-benzyl-2-deoxy-α-D-glucopyranoside (**5b**)

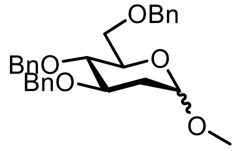

Prepared according to general glycosylation procedure A from hemiacetal donor **2e** (20 mg, 46 μmol), acceptor **3b** (1.4 μL, 35.4 μmol) and catalyst **1e** (970 μg, 1.8 μmol). After 3 hours and following purification by column chromatography (4:1 Hexane:EtOAc), **5b** was obtained as a colourless oil (6.9 mg, 15.4 μmol, 43 %, α/β 4:1).
NMR data for α-anomer: **^{1}H NMR (400 MHz, $CDCl_3$) δ** 7.43- 7.23 (m, 15H), 4.89 (d, *J* = 10.9 Hz, 1H, OCH_2Ar), 4.85 (d, $J_{1,2}$ = 2.9 Hz, 1H, H-1), 4.69- 4.62 (m, 3H, OCH_2Ar), 4.52 (dd, *J* = 11.6, 2.2 Hz, 2H, OCH_2Ar), 3.97 (ddd, $J_{3,4}$ = 11.5, $J_{3,2a}$ = 8.8, $J_{3,2b}$ = 5.1 Hz, 1H, H-3), 3.81- 3.71 (m, 2H, H-5, H-6), 3.69 (dd, $J_{6b,6a}$ = 10.0, $J_{6b,5}$ = 1.8 Hz, 1H, H-6b), 3.61 (t, $J_{4,5}$ = 9.2 Hz, 1H, H-4), 3.32 (s, 3H, OMe), 2.28 (ddd, $J_{2b,2a}$ = 13.0, $J_{2b,3}$ = 5.1, $J_{2b,1}$ = 1.5 Hz, 1H, H-2b), 1.72 (ddd, $J_{2a,2b}$ = 13.1, $J_{2a,3}$ = 11.5, $J_{2a,1}$ = 2.9 Hz, 1H, H-2a).**^{13}C NMR (126 MHz, $CDCl_3$) δ** 138.8 (4° C, Ar-C), 138.7 (4° C, Ar-C), 138.3 (4° C, Ar-C), 128.5 (2C, Ar-C), 128.4 (Ar-C), 128.1 (Ar-C), 128.0 (2C, Ar-C), 127.9 (Ar-C), 127.8 (Ar-C), 127.7 (Ar-C), 127.6 (Ar-C), 98.7 (C-1), 78.3 (C-4), 77.8 (C-3), 75.1 (OCH_2Ar), 73.6 (OCH_2Ar), 71.9 (OCH_2Ar), 70.8 (C-5), 69.1 (C-6), 54.7 (OCH_3), 35.5 (C-2).
Selected signals for β-anomer: **^{1}H NMR (400 MHz, $CDCl_3$) δ** 4.92 (d, *J* = 11.0 Hz, 1H, OCH_2Ar), 4.38 (dd, *J* = 9.7, 1.9 HZ, 1H), 3.53 (s, OCH_3), 2.36 (ddd, $J_{2b,2a}$ = 12.5, $J_{2b,3}$ = 5.1, $J_{2b,1}$ = 2.0 Hz, 1H, H-2b), 1.66 (td, $J_{2a,2b}$ = 12.1, $J_{2a,3}$ = 9.8, 1H, H-2a). **^{13}C NMR (126 MHz, $CDCl_3$) δ** 138.5, 138.4, 129.9, 129.2, 128.6, 128.5, 128.2, 127.8, 100.9 (C-1), 79.5, 78.3, 75.3, 75.1, 71.6, 69.5, 56.8, 36.8 (C-2).
Spectroscopic data was in agreement with previously reported literature.[9]

Methyl 2,3,4-tri-*O*-benzyl-6-*O*-(2-deoxy-3,4,6-tri-*O*-benzyl-α-D-lyxohexapyranosyl)-α-D-glucopyranoside (**5c**)

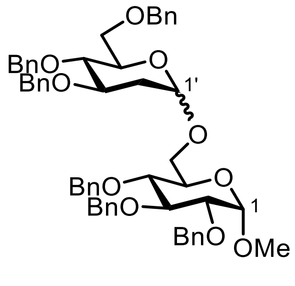

Prepared according to general glycosylation procedure A from hemiacetal donor **2e** (30 mg, 69 μmol), acceptor **3c** (25 mg, 53 μmol) and catalyst **1e** (1.5 mg, 2.7 μmol). After 12 hours and following purification using column chromatography (12:1 to 4:1 Hexane:EtOAc), **5c** was afforded as a colourless oil (26 mg, 55%, α:β 12:1).

^{1}H NMR (400 MHz, CDCl$_3$) δ 7.44–7.20 (m, 30H, ArH), 5.06 (br s, 1H, H-1'), 5.05 (d, *J* = 10.9 Hz, 1H, CH_2Ph), 4.98 (d, *J* = 11.2 Hz, 1H, CH_2Ph), 4.94 (dd, *J* = 11.0 Hz, 1H, CH_2Ph), 4.88–4.84 (m, 2H, 2 x CH_2Ph), 4.76–4.60 (m, 7H, H-1, 6 x CH_2Ph), 4.55 (dd, *J* = 11.0 Hz, 1H, CH_2Ph), 4.47 (dd, *J* = 12.1 Hz, 1H, CH_2Ph), 4.06 (t, *J* = 9.3 Hz, 1H, H-3), 4.02–3.97 (m, 1H, H-3'), 3.88 (dd, *J* = 11.3, 4.4 Hz, 1H, H-6a'), 3.80 (ddd, *J* = 10.0, 4.3, 1.5 Hz, 1H, H5'), 3.74 (ddd, *J* = 9.9, 3.3, 1.8 Hz, 1H, H-5), 3.69–3.54 (m, 3H, H-4, H6b', H-6a), 3.60–3.54 (m, 3H, H-2, H-4', H-6b), 3.41 (s, 3H, CH_3), 2.36 (ddd, *J* = 13.0, 5.0, 1.1 Hz, 1H, H-2'$_{eq}$), 1.675 (ddd, *J* = 12.9, 11.6 Hz, 3.5 Hz, 1H, H-2'$_{ax}$). **^{13}C NMR (101 MHz, CDCl$_3$) δ** 128.48 (Ar CH), 128.42 (Ar CH), 128.37 (Ar CH), 128.3 (Ar CH), 128.30 (Ar CH), 128.2 (Ar CH), 128.1 (Ar CH), 128.0 (Ar CH), 127.9 (Ar CH), 127.9 (Ar CH), 127.8 (Ar CH), 127.7 (Ar CH), 127.6 (Ar CH), 127.6 (Ar CH), 127.5 (Ar CH), 127.4 (Ar CH), 97.9 (C-1), 97.8 (C-1'), 82.2 (C-3), 80.0 (C-4'), 78.1 (C-4), 77.8 (C-2), 77.2 (C-3'), 75.8 (CH_2Ph), 74.9 (CH_2Ph), 74.8 (CH_2Ph), 73.4 (CH_2Ph), 73.3 (CH_2Ph), 71.7 (CH_2Ph), 70.9 (C-5), 69.8 (C-5'), 68.7 (C-6), 65.7 (C-6'), 55.1 (OCH_3), 35.3 (C-2').

Spectroscopic data was in agreement with previously reported literature.[9]

Benzyl 2-deoxy-3,4,6-tri-*O*-benzyl-α-D-glucopyranoside (**5d**)

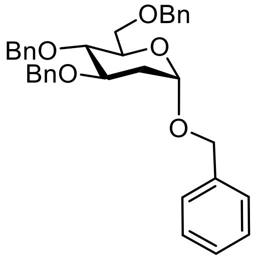

Prepared according to general glycosylation procedure A from hemiacetal donor **2e** (30 mg, 69 μmol), acceptor **3d** (6 μL, 53 μmol) and catalyst **1e** (1.5 mg, 2.7 μmol). After 3 hours and following purification using column chromatography (12:1 to 4:1 Hexane:EtOAc), **5d** was afforded as a colourless oil (28 mg, 77%, α only).

^{1}H NMR (400 MHz, CDCl$_3$) δ 7.40–7.31 (m, 18H, ArH), 7.23 (dd, *J* = 7.6, 1.9 Hz, 2H, ArH), 5.11 (d, *J* = 2.5 Hz, 1H, H-1), 4.95 (d, *J* = 10.8 Hz, 1H, CH_2Ph), 4.74–4.69 (m, 4H, 4 x CH_2Ph), 4.58 (d, *J* = 11.7 Hz, 2H, CH_2Ph), 4.50 (d, *J* = 11.9 Hz, 1H, CH_2Ph), 4.10 (ddd, *J* = 11.5, 8.8, 5.0 Hz, 1H, H-3), 3.89 (ddd, *J* = 9.7, 3.9, 1.8 Hz, 1H, H-5), 3.84 (dd, *J* = 10.4, 4.0 Hz, 1H, H-6a), 3.72 (*J* = 10.4, 1.9 Hz, 1H, H-6b), 3.69 (t, *J* = 9.3 Hz, 1H, H-4), 2.38 (ddd, *J* = 13.0, 5.1, 1.2 Hz, 1H, H-2a), 1.81 (ddd, *J* = 13.0, 11.5, 3.7 Hz, 1H, H-2b). **^{13}C NMR (101 MHz, CDCl$_3$) δ** 138.7 (Ar C),

138.5 (Ar C), 138.2 (Ar C), 137.7 (Ar C), 128.39 (Ar CH), 128.4 (Ar CH), 128.4 (Ar CH), 128.0 (Ar CH), 127.9 (Ar CH), 127.9 (Ar CH), 127.7 (Ar CH), 127.6 (Ar CH), 127.5 (Ar CH), 96.8 (C-1), 78.4 (C-4), 77.8 (C-3), 75.0 (CH_2Ph), 73.5 (CH_2Ph), 71.9 (CH_2Ph), 71.0 (C-5), 68.94 (C-6), 68.89 (CH_2Ph), 35.5 (C-2).
Spectroscopic data was in agreement with previously reported literature.[9]

Allyl 2-deoxy-3,4,6-tri-*O*-benzyl-α-D-glucopyranoside (**5e**)

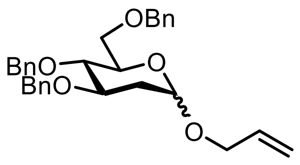

Prepared according to general glycosylation procedure A from hemiacetal donor **2e** (30 mg, 69 μmol), acceptor **3e** (5 μL, 53 μmol) and catalyst (1.5 mg, 2.7 μmol). After 3 hours and following purification using column chromatography (12:1 to 4:1 Hexane:EtOAc), **5e** was afforded as a colourless oil (24 mg, 73% $\alpha:\beta$ 6:1).
^{1}H NMR (400 MHz, $CDCl_3$) δ 7.41–7.21 (m, 15H, ArH), 5.93 (dddd, *J* = 17.2, 10.4, 6.1, 5.2 Hz, 1H, H-8), 5.30 (dq, *J* = 17.2, 1.7, 1H, H-9a), 5.20 (dq, *J* = 10.4, 1.5 Hz, 1H, H-9b), 5.05 (dd, *J* = 3.4, 1.1 Hz, 1H, H-1), 4.93 (d, *J* = 10.9 Hz, 1H, CH_2Ph), 4.74–4.50 (m, 5H, 5 x CH_2Ph), 4.17 (ddt, *J* = 13.0, 5.1, 1.5 Hz, 1H, H-7a), 4.06 (ddd, *J* = 11.5, 8.7, 5.1 Hz, 1H, H-3), 3.98 (ddt, *J* = 13.0, 6.1, 1.4 Hz, 1H, H-7b), 3.84–3.64 (m, 4H, H-4, H-5, 2 x H-6), 2.35 (ddd, *J* = 13.0, 5.1, 1.3 Hz, 1H, H-2b), 1.78 (m, *J* = 13.0, 11.5, 3.6 Hz, 1H, H-2a). **^{13}C NMR (101 MHz, $CDCl_3$) δ** 138.8 (Ar C), 138.6 Ar C), 138.2 (Ar C), 134.2 (C-8), 128.4 (Ar CH), 128.3 (Ar CH), 128.3 (Ar CH), 127.9 (Ar CH), 127.9 (Ar CH), 127.6 (Ar CH), 127.6 (Ar CH), 127.5 (Ar CH), 117.1 (C-9), 96.80 (C-1), 78.3 (C-4), 77.7 (C-3), 75.0 (CH_2Ph), 73.5 (CH_2Ph), 71.8 (CH_2Ph), 70.9 (C-5), 68.9 (C-6), 67.7 (C-7), 35.5 (C-2).
Spectroscopic data was in agreement with previously reported literature.[9]

Isopropyl-2-deoxy-3,4,6-tri-*O*-benzyl-α-D-glucopyranoside (**5g**)

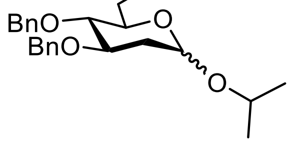

Prepared according to general glycosylation procedure A from hemiacetal donor **2e** (30 mg, 69 μmol), acceptor **3k** (5 μL, 53 μmol) and catalyst (1.5 mg, 2.7 μmol). After 3 hours and following purification using column chromatography (12:1 to 4:1 Hexane:EtOAc), **5g** was afforded as a colourless oil (25 mg, 77%, α:β 5:1).

^{1}H NMR (400 MHz, CDCl$_3$) δ 7.39–7.21 (m, 15H, ArH), 5.13–5.06 (br d, *J* = 3.2 Hz, 1H, H-1), 4.97–4.89 (d, *J* = 11.7 Hz, 1H, CH$_2$Ph), 4.69–4.57 (m, 4H, CH$_2$Ph), 4.54–4.41 (m, 3H, CH$_2$Ph, H-3), 4.05–3.92 (m, 3H, H-3, CH$_2$Ph), 3.92–3.84 (septet, *J* = 6.2 Hz, 1H, CH-*iPr*), 3.66–3.52 (m, 3H, H5, H-6a, H-6b), 2.31–2.19 (m, 1H, H-2 ax), 1.98–1.90 (m, 1H, H-2 eq)), 1.18 (d, *J* = 6.3 Hz, 3H, CH$_3$-*iPr*), 1.13 (d, *J* = 6.3 Hz, 3H, CH$_3$-*iPr*). **^{13}C NMR (101 MHz, CDCl$_3$) δ** 139.1 (Ar-C), 138.8 (Ar-C), 138.7(Ar-C), 138.3 (Ar-C), 128.6 (Ar-C), 128.5 (Ar-C), 128.4 (Ar-C), 128.3 (Ar-C), 128.1 (Ar-C), 128.0 (Ar-C), 127.8 (Ar-C), 127.7 (Ar-C), 127.6 (Ar-C), 127.5 (Ar-C), 95.7 (C-1), 75.2 (C-4), 74.3 (CH$_2$Ph), 73.7 (CH$_2$Ph), 73.3 (C-3), 70.6 (CH$_2$Ph), 70.0 (C-5), 69.7 (C-6), 68.4 (C-*iPr*),23.5 (*iPr*-CH$_3$), 21.7 (iPr-CH$_3$).

Spectroscopic data was in agreement with previously reported literature.[10]

Cholesteryl -2-deoxy-3,4,6-tri-*O*-benzyl-D-glucopyranoside **(5f)**

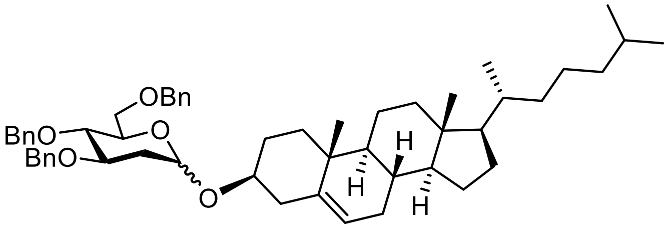

Prepared according to general glycosylation procedure A from hemiacetal donor **2e** (20 mg, 46 μmol), acceptor **3i** (13.7 mg, 35.4 μmol) and catalyst **1e** (970 μg, 1.3 μmol). After 3 hours and following purification using column chromatography (8:2 Hexane:EtOAc), **5f** was afforded as a white solid (10.1 mg, 36 %, 3:2 α:β). A 70% yield was achieved with no change in stereoselectivity when **3i** and **1e** were pre-stirred for 3h before the addition of the hemiacetal **2e**.

Data for α anomer: **^{1}H NMR (400 MHz, CDCl$_3$) δ** 7.45- 7.15 (m, 15H), 5.30 (d, *J* = 5.0 Hz, 1H, H-7), 5.16 (d, $J_{1,2b}$ = 3.4 Hz, 1H, H-1), 4.92 (d, *J* = 10.7 Hz, 1H, OCH$_2$Ar), 4.76- 4.65 (m, 3H, OCH$_2$Ar), 4.53 (dd, *J* = 11.4, 8.9 Hz, 2H, OCH$_2$Ar), 4.05 (ddd, $J_{3,2a}$ = 11.4, $J_{3,4}$ = 9.4, $J_{3,2b}$ = 5.0 Hz, 1H, H-3), 3.89 (ddd, $J_{5,4}$ = 9.4, $J_{5,6a}$ = 3.9, $J_{5,6b}$ = 2.0 Hz, 1H, H-5), 3.83 (dd, $J_{6a,6b}$ = 10.5, $J_{6a,5}$ = 4.0 H, 1H, H-6a), 3.71 (dd, $J_{6b,6a}$ = 10.5, $J_{6b,5}$ = 2.0 Hz, 1H, H-6b), 3.64 (t, $J_{4,5}$ = $J_{4,3}$ = 9.4 Hz, 1H, H-4), 3.54- 3.44 (m, 1H), 2.35- 2.25 (m, 3H, H-2a), 2.03 (dt, *J* = 12.6, 3.5 Hz, 1H), 1.97 (m, 1H), 1.94- 1.80 (m, 3H), 1.76 (td, $J_{2b,2a}$ = 12.3, $J_{2b,3}$ = 3.7 Hz, 1H, H-2b), 1.65- 1.24 (m, 14H), 1.23- 1.06 (m, 8H), 1.02 (s, 3H), 0.94 (d, *J* = 6.4 Hz, 3H), 0.90 (d, *J* = 2.3 Hz, 3H), 0.89 (d, *J* = 2.3 Hz, 3H), 0.70 (s, 3H). **^{13}C NMR (126 MHz, CDCl$_3$) δ** 140.9 (4° C), 138.9 (4° C, Ar C), 138.6 (4° C, Ar C), 137.2

(4° C, Ar C), 128.4 (2C, Ar C), 128.3 (Ar C), 128.0 (Ar C), 127.9 (Ar C), 127.6 (2C, Ar C), 127.5 (Ar C), 121.7 (C-7), 95.0 (C-1), 78.5 (C-4), 77.8 (C-3), 75.9, 75.0 (OCH_2Ar), 73.4 (OCH_2Ar), 71.8 (OCH_2Ar), 70.7 (C-5), 69.0 (C-6), 56.8, 56.1 (CH), 50.2 (CH_3), 42.3, 40.0, 39.8, 39.5, 37.1, 36.8, 36.2 (C-2), 35.9, 35.8 (CH), 31.9 (CH), 28.3, 28.0, 27.7 (CH), 24.3, 23.8, 22.8 (CH_3), 22.6, 21.1, 19.4 (CH_3), 18.7 (CH_3), 11.9 (CH_3).

Data for β anomer: **^{1}H NMR (400 MHz, $CDCl_3$) δ** 7.41- 7.20 (m, 15H, Ar-H), 5.36 (dt, *J* = 4.4, 1.9 Hz, 1H), 4.93 (d, *J* = 10.9 Hz, 1H, OCH_2Ar), 4.71 (d, *J* = 11.7 Hz, 1H, OCH_2Ar), 4.66- 4.56 (m, 5H, H-1, 4 x OCH_2Ar), 3.79 (dd, $J_{6b,6a}$ = 10.7, $J_{6b,5}$ = 1.9 Hz, 1H, H-6b), 3.74- 3.66 (m, 2H, H-3, H-6a), 3.61 (tt, J = 11.3, 4.7 Hz, 1H), 3.50 (t, $J_{4,3}$ = $J_{4,5}$ = 9.1 Hz, 1H, H-4), 3.43 (ddd, $J_{5,4}$ = 9.7, $J_{5,6a}$ = 5.2, $J_{5,6b}$ = 1.9 Hz, 1H, H-5), 2.38- 2.31 (m, 2H), 2.30- 2.23 (m, 1H, H-2), 2.08- 1.96 (m, 3H), 1.91- 1.81 (m, 2H), 1.75- 1.67 (m, 1H, H-2), 1.65- 1.23 (m, 20H), 1.22- 1.06 (m, 7H), 1.02 (s, 3H, CH_3), 0.95 (d, J = 6.4 Hz, 3H), 0.90 (d, J = 2.2 Hz, 3H), 0.89 (d, J = 2.2 Hz, 3H), 0.71 (s, 3H). **^{13}C NMR (126 MHz, $CDCl_3$) δ** 140.7 (4° C), 138.5 (4° C, Ar-C), 138.4 (4° C, Ar-C), 138.3 (4° C, Ar-C), 128.4 (Ar-C), 128.4 (Ar-C), 128.3 (Ar-C), 128.0 (Ar-C), 127.7 (Ar-C), 127.6 (Ar-C), 127.5 (Ar-C), 98.0 (C-1), 79.6 (C-3), 78.2 (C-4), 78.1, 75.2 (C-5), 74.9 (3C, OCH_2Ar), 73.4, 71.4, 69.5 (C-6), 56.8, 56.2, 50.2, 42.3, 39.8, 39.5, 38.9, 37.3 (C-2), 37.2, 36.8, 38.2, 35.8, 32.0 (CH_2), 31.9, 29.7, 28.3, 28.0, 24.3, 23.8, 22.8 (CH_3), 22.6, 21.1 (CH_2), 19.4 (3C, CH_3), 18.7, 11.9.

Spectroscopic data was in agreement with previously reported literature.[5]

6-*O*-(2-deoxy-3,4-di-*O*-benzyl-α-D-xyloranosyl)-1,2,3,4-di-*O*-isopropylidene-α-D-glucopyranoside (**6a**)

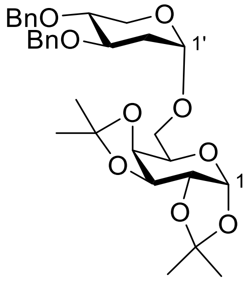

Prepared according to general glycosylation procedure A from hemiacetal donor **2f** (15.7 mg, 50 μmol), acceptor **3a** (10 mg, 53 μmol) and catalyst **1e** (1 mg, 1.9 μmol). After 24 hours and following purification using column chromatography (8:2 Hexane:EtOAc), **6a** was afforded as a pale-yellow oil (9.3 mg, 44 %, α only). R_f = 0.35 (8:2 Hexane:EtOAc). A 58% yield was achieved with no change in stereoselectivity when **3a** and **1e** were pre-stirred for 3h before the addition of the hemiacetal **2f**.

^{1}H NMR (500 MHz, $CDCl_3$) δ 7.37- 7.29 (m, 10H), 5.55 (d, $J_{1,2}$ = 5.0 Hz, 1H, H-1), 4.91 (t, $J_{1',2'b}$ = 3.0 Hz, 1H, H-1'), 4.78 (d, *J* = 11.7 Hz, 1H), 4.79-4.62 (m, 4H, H-3, OCH_2Ar), 4.34 (dd, $J_{2,1}$ = 5.0, $J_{2,3}$ = 2.4 Hz, 1H, H-2), 4.29 ($J_{4,3}$ = 7.9, $J_{4,5}$ = 1.9 Hz, 1H, H-4), 3.99 (td, $J_{5,6a}$ = 6.8, $J_{5,4}$ = 1.9 Hz,

1H, H-5), 3.92 ($J_{3',2'a}$ = 10.2, $J_{3',4'}$ = 7.9, $J_{3',2'b}$ = 4.8 Hz, 1H, H-3'), 3.78- 3.66 (m, 3H, H-6a, H-6b, H-5'a), 3.62 (dd, $J_{5'b,5'a}$ = 11.2, $J_{5'b,4}$ = 9.2 Hz, 1H, H-5'b), 3.53 (ddd, $J_{4'5'b}$ = 9.2, $J_{4',3'}$ = 7.9, $J_{4',5'a}$ = 4.9 Hz, 1H, H-4'), 2.26 (ddd, $J_{2'b,2'a}$ = 13.2, $J_{2'b,1'}$ = 5.0, $J_{2'b,3}$ = 3.0 Hz, 1H, H-2'b), 1.69 (ddd, $J_{2'a,2'b}$ = 13.4, $J_{2'a,3'}$ = 10.3, $J_{2'a,1'}$ = 3.4 Hz, 1H, H-2'a), 1.56 (s, 3H, CH_3), 1.47 (s, 3H, CH_3), 1.37 (s, CH_3), 1.36 (s, CH_3). **^{13}C NMR (126 MHz, $CDCl_3$) δ** 138.8 (4 °C, Ar-C), 138.5 (4 °C, Ar-C), 128.4 (Ar-C), 128.3 (Ar-C), 127.8 (Ar-C), 127.7 (OCH_2Ar), 127.6 (OCH_2Ar), 127.5 (Ar-C), 109.3 (4° C, $C(CH_3)_2$), 108.6 (4° C, $C(CH_3)_2$), 97.7 (C-1'), 96.4 (C-1), 77.7 (C-4'), 76.2 (C-3'), 72.9 (OCH_2Ar), 72.1 (OCH_2Ar), 70.9 (C-4), 70.7 (C-3), 70.6 (C-2), 65.8 (C-5), 65.7 (C-6), 61.2 (C-5'), 35.1 (C-2'), 26.1 (CH_3), 26.0 (CH_3), 24.9 (CH_3), 24.5 (CH_3).
HRMS: Calculated for $C_{31}H_{40}O_9$ $[M+Na]^+$ 579.2565, Observed 579.2565

Benzyl 2-deoxy-3,4-di-*O*-benzyl-α-D-xylopyranoside (**6b**)

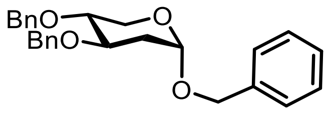

Prepared according to general glycosylation procedure A from hemiacetal donor **2f** (20 mg, 63.6 μmol), acceptor **3d** (5.3 mg, 48.9 μmol) and catalyst **1e** (1.3 mg, 1.3 μmol). After 3 hours and following purification using column chromatography (8:2 Hexane:EtOAc), **6b** was afforded as a white solid (17.3 mg, 87 %, 7:3 α:β). R_f = 0.53 (7:3 Hexane:EtOAc).
^{1}H NMR (400 MHz, $CDCl_3$) δ 7.46- 7.12 (m, 15H, Ar-H), 4.93 (t, $J_{1,2b}$ = 2.8 Hz, H-1), 4.78 (d, J = 11.7 Hz, 1H, OCH_2Ar), 4.74- 4.65 (m, 4H, 4 x OCH_2Ar), 4.43 (d, J = 11.9 Hz, 1H, OCH_2Ar), 3.95 (ddd, $J_{3,2a}$ = 10.5, $J_{3,4}$ = 8.1, $J_{3,2b}$ = 4.9 Hz, 1H, H-3), 3.76 (dd, $J_{5b,5a}$ = 11.1, $J_{5b,4}$ = 5.1 Hz, 1H, H-5b), 3.65 (dd, $J_{5a,5b}$ = 11.1, $J_{5a,4}$ = 9.6 Hz, 1H, H-5), 3.58-3.53 (m, 1H, H-4), 2.29- 2.23 (m, 1H, H-2b), 1.70 (ddd, $J_{2a,2b}$ = 13.6, $J_{2a,3}$ = 10.5, $J_{2a,1}$ = 3.6 Hz, 1H, H-2a). **^{13}C NMR (126 MHz, $CDCl_3$) δ** 138.8 (4° C, Ar-C), 138.6 (4° C, Ar-C), 137.7 (4° C, Ar-C), 128.4 (Ar-C), 128.3 (Ar-C), 128.0 (Ar-C), 127.9 (Ar-C), 127.8 (Ar-C), 127.7 (Ar-C), 127.6 (Ar-C), 96.5 (C-1), 77.9 (C-4), 76.3 (C-3), 73.0 (OCH_2Ar), 72.2 (OCH_2Ar), 68.8 (OCH_2Ar), 61.1 (C-5), 35.3 (C-2).
Selected resonances for β anomer: **^{1}H NMR (400 MHz, $CDCl_3$) δ** 4.85 (d, J = 11.9 Hz, 1H, OCH_2Ar), 4.62 (d, J = 11.8 Hz, 1H, OCH_2Ar), 4.59 (dd, $J_{1,2a}$ = 7.9, $J_{1,2b}$ = 2.8 Hz, 1H, H-1), 4.54 (d, J = 12.0 Hz, 1H, OCH_2Ar), 4.06 (dd, $J_{6b,6a}$ = 11.9, $J_{6b,5}$ = 4.4 Hz, 1H, H-6b), 3.29 (dd, $J_{6a,6b}$ = 11.9, $J_{6a,5}$ = 8.1 Hz, 1H, H-6a), 1.77- 1.73 (m, 1H, H-2). **^{13}C NMR (126 MHz, $CDCl_3$) δ** 138.5 (4° C, Ar-C), 138.4 (4° C, Ar-C), 137.7 (4° C, Ar-C), 127.9 (Ar-C), 127.8 (Ar-C), 127.7 (Ar-C), 127.6 (Ar-C), 127.5 (Ar-C), 98.4 (C-1), 77.1, 76.6, 72.8, 71.4 (OCH_2Ar), 72.8, 70.0 (OCH_2Ar), 62.9 (CH_2, C-6), 34.7 (C-2).
HRMS: Calculated for $C_{26}H_{28}O_4$ $[M+Na]^+$ 427.1880, observed 427.1881

Cholesteryl -2-deoxy-3,4-di-*O*-benzyl-α-D-xylopyranoside (**6c**)

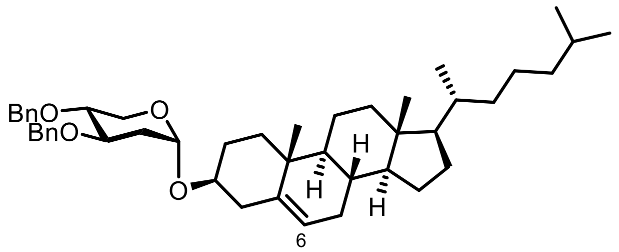

Prepared according to general glycosylation procedure A from hemiacetal donor **2f** (20 mg, 63 μmol), acceptor **3i** (18.9 mg, 49 μmol) and catalyst **1e** (1.3 mg, 2.5 μmol). After 24 hours and following purification using column chromatography (7:3 Hexane:EtOAc), **6c** was afforded as a pale-yellow oil (18.3 mg, 55 %, α only).

^{1}H NMR (500 MHz, $CDCl_3$) δ 7.44- 7.18 (m, 10 H, Ar-H), 5.36 (d, J = 4.1 Hz, 1H, H-6), 5.02 (t, $J_{1,2b}$ = 2.9 Hz, 1H, H-1), 4.79 (d, J = 11.1 Hz, 1H, OCH_2Ar), 4.76- 4.66 (m, 3H, OCH_2Ar), 3.94 (ddd, $J_{3,2a}$ = 10.3, $J_{3,4}$ = 8.0, $J_{3,2b}$ = 4.7 Hz, 1H, H-3), 3.75- 3.64 (m, 2H, H-5), 3.59- 3.42 (m, 2H, H-4), 2.37- 2.30 (m, 1H), 2.20 (ddd, $J_{2b,2a}$ = 13.1, $J_{2b,3}$ = 4.8, $J_{2b,1}$ = 2.5 Hz, 1H, H-2b), 2.07- 1.95 (m, 2H), 1.92- 1.80 (m, 3H), 1.70 (ddd, $J_{2a,2b}$ = 13.1, $J_{2a,3}$ = 10.2, $J_{2a,1}$ = 3.4 Hz, 1H, H-2a), 1.65- 1.24 (m, 14H), 1.22- 1.06 (m, 6H), 1.05- 1.01 (m, 5H), 0.94 (d, 3H), 0.90 (dd, J = 6.6, 2.2 Hz, 6H, 2 x CH_3), 0.70 (s, CH_3).

^{13}C NMR (126 MHz, $CDCl_3$) δ 140.9 (4° C), 138.9 (4° C, Ar C), 138.6 (4° C, Ar C), 128.4 (CH, Ar C), 128.4 (CH, Ar C), 128.4 (CH, Ar C), 127.8 (CH, Ar C), 127.7 (CH, Ar C), 127.7 (2C, Ar C), 127.6 (2C, Ar C), 127.5 (CH, Ar C), 121.7 (C-6), 98.0 (CH), 95.1 (C-1), 77.9 (C-4), 76.4 (C-3), 75.9, 73.0 (OCH_2Ar), 72.1 (OCH_2Ar), 61.1 (C-5), 56.8 (CH), 56.2 (CH), 50.2 (CH), 42.3, 40.0, 39.8, 39.5, 37.1, 36.8, 36.2, 35.8 (C-2), 35.7, 31.9, 28.3, 28.0, 27.7, 24.3, 23.8, 22.8 (CH_3), 22.6 (CH_3), 21.1, 19.4 (CH_3), 18.7 (CH_3), 11.9 (CH_3).

HRMS: Calculated for $C_{46}H_{66}O_4$ $[M+Na]^+$ 705.4853, Observed: 705.4843.

6-O-(3,4,6-Tri-O-tert-butyldimethylsilyl-α-D-lyxo-hexapyranosyl)-1,2:3,4-di-O-isopropylidene-α-D-galactopyranoside (**7a**)

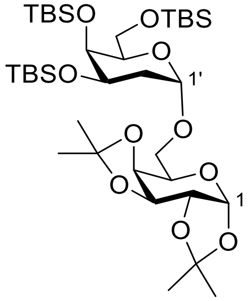

Prepared according to general glycosylation procedure A from hemiacetal donor **2c** (30 mg, 59 μmol), acceptor **3a** (11.9 mg, 45 μmol) and catalyst **1e** (1.3 mg, 2.3 μmol). After 6 hours

and following purification using column chromatography (8:2 Hexane:EtOAc), **7a** was afforded as a colourless oil (32.0 mg, 73 μmol, 94 %, α only).
^{1}H NMR (500 MHz, $CDCl_3$) δ 5.50 (d, *J* = 5.0 Hz, 1H), 4.90 (d, *J* = 3.3 Hz, 1H), 4.90 (d, *J* = 3.3 Hz, 1H), 4.60 (dd, *J* = 7.9, 2.4 Hz, 1H), 4.30 (dd, *J* = 5.0, 2.4 Hz, 1H), 4.20 (dd, *J* = 7.9, 1.9 Hz, 1H), 4.05 (ddd, *J* = 11.7, 4.3, 2.3 Hz, 1H), 3.95 (td, *J* = 6.3, 1.9 Hz, 1H), 3.84 (d, *J* = 2.3 Hz, 1H), 3.75 (dd, *J* = 10.3, 6.9 Hz, 1H), 3.70 – 6.54 (m, 2H), 3.63- 3.54 (m, 2H), 2.09 (td, *J* = 12.1, 3.6 Hz, 1H), 1.62 (dd, *J* = 12.4, 4.4 Hz, 1H, H-2'b), 1.52 (s, 3H, CH_3), 1.43 (s, 3H, CH_3), 1.33 (s, 3H, CH_3), 1.32 (s, 3H, CH_3), 0.92- 0.86 (m, 27H, $SiC(CH_3)_3$), 0.12- 0.03 (m, 18H, $SiCH_3$). **^{13}C NMR (126 MHz, $CDCl_3$) δ** 109.4, 108.6, 97.6, 96.5, 72.6, 71.4, 70.9, 70.1, 68.4, 66.7, 65.4, 62.2, 33.8, 26.4 ($SiC(CH_3)_3$), 26.32 ($SiC(CH_3)_3$), 26.27 (CH_3), 26.1 (CH3), 26.0 ($SiC(CH_3)_3$), 25.2 (CH_3), 24.6 (CH_3), 18.8 ($SiC(CH_3)_3$), 18.7 ($SiC(CH_3)_3$), 18.4 ($SiC(CH_3)_3$), -3.8 ($SiCH_3$), -4.2 ($SiCH_3$), -4.6 (SiCH3), -4.8 ($SiCH_3$), -5.1 ($SiCH_3$), -5.2 ($SiCH_3$).
All spectroscopic data is in accordance with the literature.{Balmond, 2012 #134}

6-O-(3,4,6-Tri-O-methyl-α-D-lyxo-hexapyranosyl)-1,2:3,4-di-O-isopropylidene-α-D-galactopyranoside **8a**

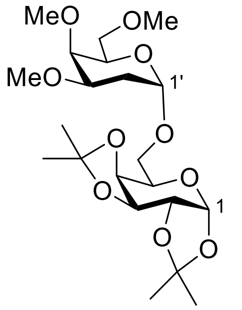

Prepared according to general glycosylation procedure A from hemiacetal donor **2d** (15 mg, 73 μmol), acceptor **3a** (14.6 mg, 56 μmol) and catalyst **1e** (1.5 mg, 2.8 μmol). After 6 hours and following purification using column chromatography (1:1 Hexane:EtOAc), **8a** was afforded as a colourless oil (12.3 mg, 27 μmol, 49 %, α only).
^{1}H NMR (500 MHz, $CDCl_3$) δ 5.52 (d, *J* = 5.0 Hz, 1H, H-1), 5.00 (dd, *J* = 3.8, 1.2 Hz, 1H, H-1'), 4.61 (dd, *J* = 7.9, 2.4 Hz, 1H, H-3), 4.31 (dd, *J* = 5.0, 2.4 Hz, 1H, H-2), 4.24 (dd, *J* = 7.9, 2.0 Hz, 1H, H-4), 3.96 (td, *J* = 6.6, 1.9 Hz, 1H, H-5), 3.92- 3.87 (m, 1H, H-5'), 3.74 (dd, *J* = 10.7, 6.7 Hz, 1H, H-6a), 3.69- 3.61 (m, 3H, H-6b, H-3', H-4'), 3.59- 3.56 (m, 1H, H-6'a), 3.56 (s, 3H, OCH_3), 3.47 (dd, *J* = 9.4, 5.8 Hz, 1H, H-6'b), 3.40 (s, 3H, OCH_3), 3.39 (s, 3H, OCH_3), 2.05- 1.91 (m, 2H, H-2'a,H-2'b), 1.53 (s, 3H, CH_3), 1.44 (s, 3H, CH_3), 1.34 (s, 3H, CH_3), 1.33 (s, 3H, CH_3). **^{13}C NMR (126 MHz, $CDCl_3$) δ** 109.4 (4° C), 108.6 (4° C), 97.6 (C-1'), 96.4 (C-1), 77.2, 75.8 (C-3'), 74.4 (C-4'), 71.4 (C-6'), 71.1 (C-4), 70.7 (C-2), 70.6 (C-3), 69.4 (C-5'), 65.9 (C-6), 65.7 (C-6), 60.9 (OCH_3), 59.1 (OCH_3), 56.1 (OCH_3), 30.7 (C-2'), 26.1 (CH_3), 26.0 (CH_3), 24.9 (CH_3), 24.6 (CH_3).
All spectroscopic data is in accordance with the literature.{Balmond, 2012 #134}

General Glycosylation procedure B

Hemiacetal donor (1 eq) and hemiacetal acceptor (2 eq) were added to microwave tubes/ RBF and placed under N_2. The vessels were sealed, and anhydrous DCM was added to dissolve the substrates. The catalyst (5 mol %) was added, and the reactions were stirred until the reaction was deemed to be complete by TLC, after which the reaction mixture was concentrated. The crude products were purified using silica gel flash column chromatography.

General Glycosylation procedure C (Symmetrical trehalose)

Hemiacetal donor was added to microwave tubes/ RBF and placed under N_2. The vessels were sealed, and anhydrous DCM was added to dissolve the substrates. The catalyst (5 mol %) was added, and the reactions were stirred until the reaction was deemed to be complete by TLC, after which the reaction mixture was concentrated. The crude products were purified using silica gel flash column chromatography.

Synthesis of trehalose derivatives

3,4,6-Tri-*O*-benzyl-2-deoxy-α-D-galactopyranosyl 3',4',6'-tri-O-acetyl-2-deoxy-α-D-glucopyranoside (**9a**)

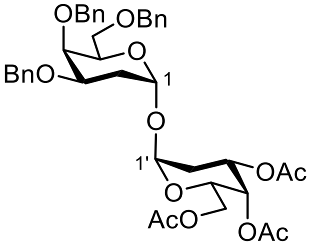

Prepared according to general glycosylation procedure B from hemiacetal donor **2a** (46 mg, 69 µmol), hemiacetal acceptor **2g** (26.7 mg, 92 µmol) and catalyst **1e** (1.3 mg, 2.3 µmol). After 24 hours and following purification using column chromatography (7:3 Hexane:EtOAc), **9a** was afforded as a colourless oil (21.8 mg, 67 %). R_f = 0.36 (7:3 Hexane:EtOAc).

^{1}H NMR (400 MHz, CDCl$_3$) δ 7.40- 7.28 (m, 15H, Ar-H), 5.35 (dd, $J_{4',3'}$ = 9.4, $J_{1',2'b}$ = 3.5 Hz, 2H, H-1', H-4'), 5.33- 5.27 (m, 2H, H-1, H-3'), 4.97 (d, J = 11.5 Hz, 1H, OCH_2Ar), 4.77- 4.62 (m, 3H, OCH_2Ar), 4.51 (d, J = 11.7 Hz, 1H), 4.44 (d, J = 11.7 Hz, 1H), 4.15- 4.08 (m, 3H, H-5', H-6'a, H-6'b), 3.97- 3.93 (m, 2H, H-3, H-4), 3.88 (t, $J_{5,6a}$ = $J_{5,4}$ = 6.9 Hz, 1H, H-5), 3.62 (dd, $J_{6a,6b}$ = 9.4, $J_{6a,5}$ = 6.9 Hz, 1H, H-6a), 3.57 (dd, $J_{6b,6a}$ = 9.6, $J_{6b,5}$ = 5.9 Hz, 1H, H-6b), 2.34 (dd, $J_{2b,2a}$ = 11.7, $J_{2b,3}$ = 3.8 Hz, 1H, H-2b), 2.16 (s, 3H, C(O)CH_3), 2.13 (dd, $J_{2'b,2'a}$ = 12.4, $J_{2'b,3}$ = $J_{2'b,1}$ = 3.5 Hz, 1H, H-2'b),

2.06 (s, 3H, C(O)CH_3), 2.03 (s, 3H, C(O)CH_3), 1.97- 1.93 (m, 1H, H-2), 1.82 (dd, $J_{2'a,2'b}$ = 12.4, $J_{2'a,3}$ = 5.3 Hz, 1H, H-2'a).**^{13}C NMR (101 MHz, $CDCl_3$) δ** 170.7 (C=O), 170.5 (C=O), 170.3 (C=O), 138.9 (4 °C, Ar-C), 138.4 (4 °C, Ar-C), 138.1 (4 °C, Ar-C), 128.6 (Ar-C), 128.5 (Ar-C), 128.4 (Ar-C), 128.3 (Ar-C), 127.9 (Ar-C), 127.8 (Ar-C), 127.8 (Ar-C), 127.7 (Ar-C), 127.6 (Ar-C), 93.5 (C-1'), 92.7 (C-1), 74.6 (C-3), 74.5 (OCH_2Ar), 73.7 (OCH_2Ar), 73.1 (C-4), 70.9 (C-5), 70.8 (OCH_2Ar), 69.6 (C-6), 67.2 (C-5'), 66.7 (C-4'), 66.2 (C-3'), 62.6 (C-6'), 30.8 (C-2), 29.9 (C-2'), 21.1 (C(O)CH_3), 20.8 (2C, C(O)CH_3).
HRMS: Calculated for $C_{39}H_{46}O_{12}$ $[M+Na]^+$ 729.2881 Observed, 729.2869

3,4,6-Tri-*O*-benzyl-2-deoxy-α-D-galactopyranosyl 3',4',6'-tri-*O*-benzyl-2-deoxy-α-D-galactopyranoside **(9b)**

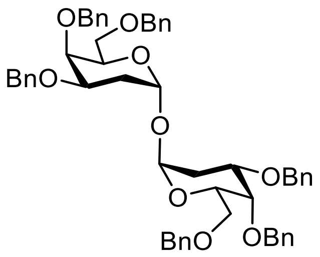

Prepared according to general glycosylation procedure B from hemiacetal donor **2a** (40 mg, 92 µmol) and catalyst **1e** (1.3 mg, 2.3 µmol). After 24 hours and following purification using column chromatography (7:3 Hexane:EtOAc), **9b** was afforded as a colourless oil (25.8 mg, 66 %). R_f = 0.46 (7:3 Hexane:EtOAc).
^{1}H NMR (400 MHz, $CDCl_3$) δ 7.44- 7.01 (m, 30H), 5.28 (t, $J_{1,2b}$ = 3.5 Hz, 1H, H-1), 4.97 (dd, J = 11.6, 2.4 Hz, 2H, OCH_2Ar), 4.65 (d, J = 11.8 Hz, 2H, OCH_2Ar), 4.61 (d, J = 3.2 Hz, 3H, OCH_2Ar), 4.52 (d, J = 11.6 Hz, 2H, OCH_2Ar), 4.45 (d, J = 11.7 Hz, 2H, OCH_2Ar), 3.97 (d, $J_{4,5}$ = 2.8 Hz, 2H, H-4), 3.92- 3.85 (m, 4H, H-3, H-5), 3.66 (dd, $J_{6a,6b}$ = 9.3, $J_{6a,5}$ = 7.3 Hz, 2H, H-6a), 3.57 (dd, $J_{6b,6a}$ = 9.3, $J_{6b,5}$ = 5.6 Hz, 2H, H-6b), 2.28 (td, $J_{2b,2a}$ = 12.4, $J_{2b,1}$ = 3.6 Hz, 2H, H-2b), 1.89 (dd, $J_{2a,2b}$ = 12.4, $J_{2a,3}$ = 4.1 Hz, 2H, H-2a).**^{13}C NMR (126 MHz, $CDCl_3$) δ** 139.0 (4° C, Ar C), 138.6 (4° C, Ar C), 138.2 (4° C, Ar C), 128.6 (4C, OCH_2Ar), 128.4 (2C, OCH_2Ar), 128.3 (2C, OCH_2Ar), 128.0 (2C, OCH_2Ar), 127.9 (2C, OCH_2Ar), 127.7 (OCH_2Ar), 127.6 (OCH_2Ar), 127.5 (2C, OCH_2Ar), 93.5 (C-1), 74.5, 74.4 (C-3), 73.7, 73.1 (C-4), 70.6, 70.5 (C-5), 69.5 (C-6), 40.0 (C-2).
Spectroscopic data is in accordance with the literature.[7]

3,4,6-Tri-*O*-benzyl-2-deoxy-α-D-galactopyranosyl 3',4',6'-tetra-*O*-benzyl-2-deoxy-α-D glucopyranoside (**9c**)

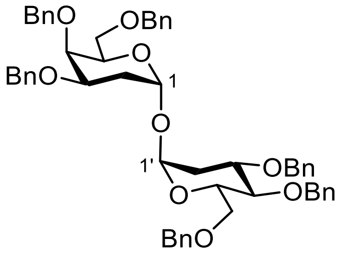

Prepared according to general glycosylation procedure B from hemiacetal donor **2a** (20 mg, 46 μmol), hemiacetal acceptor **2e** and catalyst (1.3 mg, 2.3 μmol). After 24 hours and following purification using column chromatography (3:1 Hexane:EtOAc), 9**c** was afforded as a colourless oil (29.2 mg, 75 %). R_f = 0.40 (3:1 Hexane:EtOAc).

^{1}H NMR (500 MHz, $CDCl_3$) δ 7.3- 7.25 (m, 30H, Ar-H), 5.24 (t, $J_{1,2}$ = 5.2 Hz, 2H, H-1, H-1'), 4.91 (dd, J = 17.1, 11.2 Hz, 2H, OCH_2Ar), 4.66- 4.60 (m, 4H, OCH_2Ar), 4.58 (s, 2H, OCH_2Ar), 4.53 (m, 2H, OCH_2Ar), 4.48 (d, J = 11.3 Hz, 1H, OCH_2Ar), 4.41 (d, J = 11.7 Hz, 1H, OCH_2Ar), 3.92 (td, J = 7.5, 4.8 Hz, 2H, H-3, H-5), 3.88- 3.81 (m, 2H, H-3', H-5'), 3.78 (dd, $J_{6a,6b}$ = 10.5, $J_{6a,5}$ = 3.7 Hz, 1H, H-6a), 3.75- 3.70 (m, 1H, H-4), 3.65 (dd, $J_{6b,6a}$ = 10.5, $J_{4',5'}$ = 1.7 Hz, 2H, H-4', H-6b), 3.61 (dd, $J_{6'a,6'b}$ = 9.4, $J_{6'a,5'}$ = 7.2 Hz, 1H, H-6'a), 3.53 (dd, $J_{6'b,6'a}$ = 9.4, $J_{6'b,5'}$ = 5.7 Hz, 1H, H-6'b), 2.23 (td, $J_{2'b,2'a}$ = 12.4, $J_{2\text{-}b,3'}$ = 3.8 Hz, 1H, H-2'b), 2.13 (ddd, $J_{2b,2a}$ = 13.1, $J_{2b,3}$ = 5.0, $J_{2b,1}$ = 1.5 Hz, 1H, H-2b), 1.86 (dd, $J_{2'a,2'b}$ = 12.4, $J_{2'a,3}$ = 4.6 Hz, 1H, H-2'a), 1.73 (ddd, $J_{2a,2b}$ = 12.9, $J_{2a,3}$ = 11.5, $J_{2a,1}$ = 3.6 Hz, 1H, H-2a).**^{13}C NMR (126 MHz, $CDCl_3$) δ** 139.0 (4° C, Ar C), 138.8 (4° C, Ar C), 138.6 (4° C, Ar C), 138.5 (4° C, Ar C), 138.3 (4° C, Ar C), 138.2 (4° C, Ar C), 128.6 (OCH_2Ar), 128.5 (4C, OCH_2Ar) 128.4 (OCH_2Ar), 128.3 (OCH_2Ar), 128.2 (OCH_2Ar), 128.0 (OCH_2Ar), 127.9 (OCH_2Ar), 127.8 (2C, OCH_2Ar), 127.7 (2C, OCH_2Ar), 127.6 (OCH_2Ar), 127.5 (OCH_2Ar), 93.6 (C-1), 93.1 (C-1), 78.3 (C-4), 77.4 (C-3), 75.3 (OCH_2Ar), 74.5 (OCH_2Ar), 74.4 (C-3' or C-5'), 73.7 (OCH_2Ar), 73.6 (OCH_2Ar), 73.2 (C-3 or C-5), 71.9 (OCH_2Ar), 71.7 (OCH_2Ar), 71.5 (H-4), 70.7 (C-5'), 70.6 (OCH_2Ar), 69.6 (C-6'), 68.9 (C-6), 35.4 (C-2), 30.9 (C-2').

HRMS: Calculated for $C_{54}H_{58}O_9$ $[M+Na]^+$ 873.3973, Observed 873.3962

3,4,6-Tri-*O*-benzyl-2-deoxy-α-D-galactopyranosyl 3',4',6'-tri-*O*-benzyl-2- acetyl-α-D-galactopyranoside (**9d**)

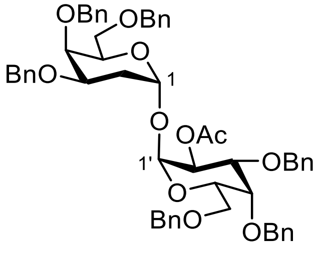

Prepared according to general glycosylation procedure B from hemiacetal donor **2a** (20 mg, 46 μmol), hemiacetal acceptor **2h** (45.3 mg, 92 μmol)and catalyst **1e** (1.3 mg, 2.3 μmol). After 24 hours and following purification using column chromatography (3:1 Hexane:EtOAc), **9d** was afforded as a colourless oil (28.8 mg, 69 %). R_f = 0.44 (3:1 Hexane:EtOAc).
^{1}H NMR (500 MHz, $CDCl_3$) δ 7.43- 7.18 (m, 30 H, Ar-H), 5.42 (dd, $J_{2,3}$ = 10.5, $J_{2,1}$ = 3.8 Hz, 1H, H-2'), 5.31 (d, $J_{1',2'a}$ = 3.8 Hz, 1H, H-1'), 5.23 (d, $J_{1,2b}$ = 3.9 Hz, 1H, H-1), 4.96 (dd, J = 11.5, 9.4 Hz, 2H, OCH_2Ar), 4.71 (d, J = 12.1 Hz, 1H, OCH_2Ar), 4.67- 4.57 (m, 5H, OCH_2Ar), 4.51 (d, J = 11.6 Hz, 1H, OCH_2Ar), 4.45 (q, J = 5.6 Hz, 2H, OCH_2Ar), 4.39 (d, J = 11.7 Hz, 1H, OCH_2Ar), 4.06 (d, $J_{4,5}$ = 1.3 Hz, 1H, H-4'), 4.01- 3.93 (m, 3H, H-4, H-5, H-5'), 3.92 (dd, $J_{3,2b}$ = 4.4, $J_{3,4}$ = 2.4 Hz, 1H, H-3), 3.88 (dd, $J_{3',2'a}$ = 10.5, $J_{3',4'}$ = 2.8 Hz, 1H, H-3'), 3.69- 3.60 (m, 2H, H-6a' and H-6a), 3.57 (dd, $J_{6'b,6'a}$ = 9.2, $J_{6'b,5'}$ = 5.6 Hz, 1H, H-6'b), 3.45 (dd, $J_{6b,6a}$ = 8.9, $J_{6b,5}$ = 5.2 Hz, 1H, H-6b), 2.29 (td, $J_{2b,2a}$ = 13.1, $J_{2b,1}$ = 3.9 Hz, 1H, H-2b), 1.98 (s, 3H, OCH_3), 1.91 (dd, $J_{2a,2b}$ = 13.1, $J_{2a,1}$ = 4.4 Hz, 1H, H-2a).**^{13}C NMR (126 MHz, $CDCl_3$) δ** 170.1 (4° C, C=O), 138.8 (4° C, Ar-C), 138.5 (4° C, Ar-C), 138.4 (4° C, Ar-C), 138.3 (4° C, Ar-C), 137.9 (4° C, Ar-C), 137.8 (4° C, Ar-C), 128.5 (2C, Ar-CH), 128.4 (6C, Ar-CH), 128.3 (6C, Ar-CH), 128.2 (2C, Ar-CH), 128.1 (2C, Ar-CH), 128.0 (2C, Ar-CH), 127.9 (2C, Ar-CH), 127.7 (3C, Ar-CH), 127.4 (2C, Ar-CH), 127.3 (2C, Ar-CH), 94.1 (C-1), 92.7 (C-1'), 76.5 (C-3'), 74.8 (OCH_2Ar), 74.4 (OCH_2Ar), 74.4 (C-4'), 74.3 (C-3), 73.6 (OCH_2Ar), 73.5 (OCH_2Ar), 72.7 (C-5'), 72.5 (OCH_2Ar), 70.4 (OCH_2Ar), 70.3 (C-2'), 70.0 (C-5, C-4), 68.8 (C-6), 68.6 (C-6'), 30.9 (C-2).
HRMS: Calculated for $C_{56}H_{60}O_{11}$ $[M+Na]^+$ 931.4028, observed 931.4010

3,4,6-Tri-*O*-benzyl-2-deoxy-α-D-galactopyranosyl 2',3',4'-tri-*O*-benzyl-6-acetyl-α-D-glucopyranoside (**9e**)

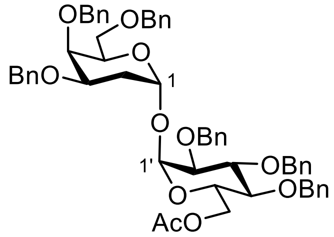

Prepared according to general glycosylation procedure B from hemiacetal donor **2a** (20 mg, 46 μmol), hemiacetal acceptor **2i** (45.3mg, 92 μmol) and catalyst **1e** (1.3 mg, 2.3 μmol). After 24 hours and following purification using column chromatography (3:1 Hexane:EtOAc), **9e** was afforded as a colourless oil (24.0 mg, 57 %). R_f = 0.44 (3:1 Hexane:EtOAc).

^{1}H NMR (500 MHz, $CDCl_3$) δ 7.39- 7.23 (m, 30H, Ar-H), 5.29 (d, *J* = 3.5 Hz, 2H, H-1, H-1'), 4.98 (d, *J* = 10.8 Hz, 1H, OCH_2Ar), 4.94 (d, *J* = 11.5 Hz, 1H, OCH_2Ar), 4.89 (d, J = 10.6 Hz, 1H, OCH_2Ar), 4.82 (d, *J* = 10.9 Hz, 1H, OCH_2Ar), 4.74 (d, *J* = 11.9 Hz, 1H, OCH_2Ar), 4.64 (d, *J* = 2.1 Hz, 2H, OCH_2Ar), 4.62 (d, *J* = 11.7 Hz, 1H, OCH_2Ar), 4.56 (dd, *J* = 11.3, 4.9 Hz, 2H, OCH_2Ar), 4.44 (d, *J* = 11.8 Hz, 1H, OCH_2Ar), 4.37 (d, *J* = 11.8 Hz, 1H, OCH_2Ar), 4.31 (dd, $J_{6a,6b}$ = 12.0, $J_{6a,5}$ = 4.7 Hz, 1H, H-6a), 4.24 (dd, $J_{6b,6a}$= 12.0, $J_{6b,5}$ = 2.3 Hz, 1H, H-6b), 4.20 (t, $J_{3',4'}$ = 6.4 Hz, 1H, H-3'), 4.07- 4.02 (m, 1H, H-3), 3.97 (d, $J_{4,5}$ = 9.2 Hz, 1H, H-4), 3.94 (d, $J_{5',6'}$ = 7.7 Hz, 1H, H-5'), 3.89- 3.85 (m, 1H, H-5), 3.57- 3.54 (m, 3H, H-2', H-6'), 3.50 (t, $J_{4',5'}$ = 8.9 Hz, 1H, H-4'), 2.32 (td, $J_{2a,2b}$ = 12.5, $J_{2a,3}$ = 3.7 Hz, 1H, H-2a), 2.03 (s, 3H, $COCH_3$), 1.95 (m, 1H, H-2b). **^{13}C NMR (126 MHz; $CDCl_3$) δ** 170.76 (4° C, C=O), 138.8 (4° C, Ar-C), 138.7 (4° C, Ar-C), 138.4 (4° C, Ar-C), 138.1 (4° C, Ar-C), 138.0 (4° C, Ar-C), 137.8 (4° C, Ar-C), 128.6 (Ar-C), 128.5 (Ar-C), 128.4 (Ar-C), 128.3 (Ar-C), 128.3 (Ar-C), 128.2 (Ar-C), 128.1 (Ar-C), 127.9 (Ar-C), 127.8 (Ar-C), 127.7 (Ar-C), 127.5 (Ar-C), 127.5 (Ar-C), 93.7 (C-1), 91.9 (C-1), 81.6 (C-4), 79.2 (C-2'), 77.5 (C-4'), 75.6 (OCH_2Ar), 75.3 (OCH_2Ar), 74.4 (OCH_2Ar), 74.3 (C-3), 73.6 (OCH_2Ar), 73.1 (C-5'), 72.4 (OCH_2Ar), 70.6 (C-3'), 70.6 (OCH_2Ar), 69.7 (C-6'), 69.2 (C-5), 63.1 (C-6), 30.9 (C-2), 20.8 (OCH_3).

HRMS: Calculated for $C_{56}H_{60}O_{11}$ $[M+Na]^+$ 931.4028, observed 931.3982

3,4,6-Tri-*O*-benzyl-2-deoxy-α-D-glucopyranosyl 2',3',4',6'-tetra-*O*-benzoyl-α-D-glucopyranoside (**9f**)

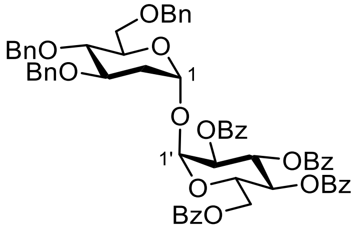

Prepared according to general glycosylation procedure B from hemiacetal donor **2c** (30 mg, 69 μmol), hemiacetal **2j** (82 mg, 138 μmol) and catalyst **1e** (1.3 mg, 2.3 μmol). After 24 hours and following purification using column chromatography (10:1 to 3:1 Hexane:EtOAc), **9f** was afforded as a colourless oil (27.0 mg, 39 %). R_f = 0.35 (3:1 Hexane:EtOAc).

^{1}H NMR (400 MHz, $CDCl_3$) δ 8.07–7.90 (m, 8H, ArH), 7.61–7.53 (m, 2H, ArH), 7.49–7.18 (m, 12H, ArH), 7.08–7.05 (m, 2H, ArH), 6.22 (t, 1H, *J* = 10.0 Hz, H-3), 5.73 (t, 1H, *J* = 9.8 Hz, H-4), 5.65 (d, 1H, *J* = 3.9 Hz, H-1), 5.52 (dd, 1H, *J* = 10.3, 3.9 Hz, H-2), 5.35 (d, 1H, *J* = 2.6 Hz, H-1'), 4.85–4.74 (m, 3H, 3 x CH_2Ph), 4.63 (dd, 1H, *J* = 11.8, 2.7 Hz, H-6), 4.54–4.45 (m, 3H, H-5, H6, CH_2Ph), 4.43 (d, 1H, *J* = 11.1 Hz, CH_2Ph), 4.26 (d, 1H, *J* = 12.2 Hz, CH_2Ph), 4.14 (ddd, *J* = 11.5, 8.2, 5.0 Hz, 1H, H-3'), 3.67 (dt, *J* = 10.0, 2.1 Hz, 1H, H5'), 3.63 (dd, *J* = 9.9, 8.3 Hz, H-4'), 3.19 (dd, *J* = 10.8, 2.8 Hz, 1H, H-6'), 3.11 (d, *J* = 10.8, 1.7 Hz, 1H, H-6'), 2.31 (ddd, *J* = 13.1, 5.1, 1.0

Hz, 1H, H-2' eq), 1.85 (ddd, J = 13.2, 11.16, 3.6 Hz, 1H, H-2' ax).**^{13}C NMR (101 MHz, $CDCl_3$) δ** 166.1 (C=O), 165.8 (C=O), 165.4 (C=O), 165.3 (C=O), 138.8 (Ar C), 138.6 (Ar C), 137.9 (Ar C), 133.5 (Ar CH), 133.4 (Ar CH), 133.2 (Ar CH), 129.9 (Ar CH), 129.8 (Ar CH), 129.73 (Ar CH), 129.7 (Ar CH), 129.6 (Ar CH), 129.1 (Ar CH), 128.9 (Ar CH), 128.6 (Ar CH), 128.5 (Ar CH), 128.5 (Ar CH), 128.3 (Ar CH), 128.2 (Ar CH), 128.1 (Ar CH), 128.0 (Ar CH), 127.8 (Ar CH), 127.7 (Ar CH), 127.6 (Ar CH), 127.5 (Ar CH), 127.3 (Ar CH), 94.1 (C-1'), 91.7 (C-1), 77.7 (C-4), 77.1 (C-3'), 74.5 (CH_2Ar), 73.3 (CH_2Ar), 72.5 (CH_2Ar), 71.5 (C-5'), 71.0 (C-2), 70.3 (C3), 69.7 (C-4), 68.4 (C-5), 67.8 (C-6'), 63.1 (C-6), 35.2 (C-2').
Spectroscopic data was in agreement with previously reported literature.[1]

3,4,6-Tri-*O*-benzyl-2-deoxy-α-D-glucopyranosyl 3',4',6'-tri-*O*-benzyl-2-deoxy-α-D-glucopyranoside (**9g**)

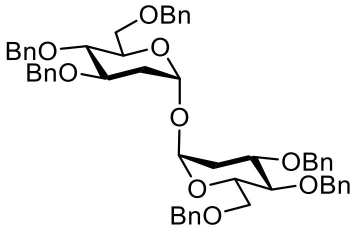

Prepared according to general glycosylation procedure C from hemiacetal donor **2e** (30 mg, 69 μmol) catalyst **1e** (1.3 mg, 2.3 μmol). After 6 hours and following purification using column chromatography (10:1 to 4:1 Hexane:EtOAc), **9g** was afforded as a white solid (34.0 mg, 56 %). R_f = 0.44 (3:1 Hexane:EtOAc).
^{1}H NMR (400 MHz, $CDCl_3$) δ 7.38–7.28 (m, 30H, ArH), 5.29 (d, J = 2.4 Hz, 2H, H-1), 4.95 (d, J = 10.7 Hz, 2H, CH_2Ph), 4.71–4.64 (m, 6H, 6 x CH_2Ph), 4.60–4.55 (m, 4H, 4 x CH_2Ph), 3.99 (ddd, J = 11.4, 8.7, 5.0 Hz, 2H, H-3), 3.83–3.76 (m, 4H, H-5, H-6a), 3.71–3.65 (m, 4H, H-4, H-6b), 2.19 (ddd, J = 13.0, 5.0, 1.2 Hz, 2H, H-2_b), 1.77 (ddd, J = 13.0, 11.5, 3.6 Hz, 2H, H-2_a). **^{13}C NMR (101 MHz, $CDCl_3$) δ** 138.6 (Ar C), 138.4 (Ar C), 138.2 (Ar C), 128.44 (Ar CH), 128.38 (Ar CH), 128.1 (Ar CH),127.8 (Ar CH), 127.7 (Ar CH), 127.64 (Ar CH), 127.63 (Ar CH), 92.8 (C-1), 78.1 (C-4), 77.3 (C-4), 75.2 (CH_2Ph), 73.5 (CH_2Ph), 71.8 (CH_2Ph), 71.5 (C-5), 68.8 (C-6), 35.1 (C-2).
Spectroscopic data was in agreement with previously reported literature.[1]

3,4,6-Tri-*O*-benzyl-2-deoxy-α-D-glucopyranosyl 2',3',4',6'-tetra-*O*-acetyl-α- D glucopyranoside (**9h**)

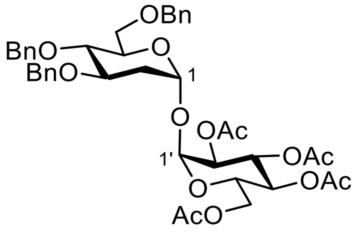

Prepared according to general glycosylation procedure B from hemiacetal donor **2e** (30 mg, 69 μmol), hemiacetal acceptor **2k** (48 mg, 138 μmol) and catalyst **1e** (1.3 mg, 2.3 μmol). After 24 hours and following purification using column chromatography (10:1 to 3:1 Hexane:EtOAc) **9h** was afforded as a colourless oil (26.0 mg, 49 %). R_f = 0.3 (3:1 Hexane:EtOAc).

^{1}H NMR (400 MHz, $CDCl_3$) δ 7.42–7.22 (m, 15H, ArH), 5.52 (dd, *J* = 10.1, 9.6 Hz, 1H, H-3), 5.38 (d, *J* = 3.8 Hz, 1H, H-1), 5.25 (d, *J* = 2.6 Hz, 1H, H-1'), 5.12 (t, *J* = 9.8 Hz, 1H, H-4), 5.04 (dd, *J* = 10.3, 3.8 Hz, 1H, H-2), 4.93 (d, *J* = 11.0 Hz, 1H, CH_2Ph), 4.76–4.71 (m, 2H, 2 x CH_2Ph), 4.64–4.50 (m, 3H, 3 x CH_2Ph), 4.30 (dd, *J* = 12.3, 4.6 Hz, 1H, H-6), 4.12–3.99 (m, 3H, H-3', H-5, H-6), 3.78–3.71 (m, 2H, H-5', H-6'), 3.65 (t, *J* = 9.0 Hz, 2H, H-4', H-6'), 2.22–2.17 (m, 1H, H-2'b), 2.12 (s, 3H, $C(O)CH_3$), 2.10 (s, 3H, $C(O)CH_3$), 2.06 (s, 3H, $C(O)CH_3$), 2.02 (s, 3H, $C(O)CH_3$), 1.84 (ddd, *J* = 13.2, 11.5, 3.7 Hz, 1H, H-2'a). **^{13}C NMR (101 MHz, $CDCl_3$) δ** 170.6 (C=O), 170.2 (C=O), 169.9 (C=O), 169.5 (C=O), 138.5 (Ar C), 138.5 (Ar C), 138.0 (Ar C), 129.0 (Ar CH), 128.5 (Ar CH), 128.4 (Ar CH), 128.3 (Ar CH), 128.2 (Ar CH), 128.0 (Ar CH), 127.83 (Ar CH), 127.8 (Ar CH), 127.7 (Ar CH), 127.7 (Ar CH), 127.6 (Ar CH), 125.3 (Ar CH), 93.6 (C1'), 91.4 (C-1), 77.9 (C-4'), 77.0 (C-5), 75.0 (CH_2Ph), 73.6 (CH_2Ph), 72.4 (CH_2Ph), 72.1 (C-5'), 70.1 (C-3), 69.9 (C-2), 68.7 (C-4), 68.6 (C-6'), 67.8 (C-3'), 61.8 (C-6), 35.2 (C-2'), 20.9 ($C(O)CH_3$), 20.7 ($C(O)CH_3$), 20.64 ($C(O)CH_3$), 20.61 ($C(O)CH_3$).

Spectroscopic data was in agreement with previously reported literature.[1]

Iterative synthesis of the tetra saccharide 11

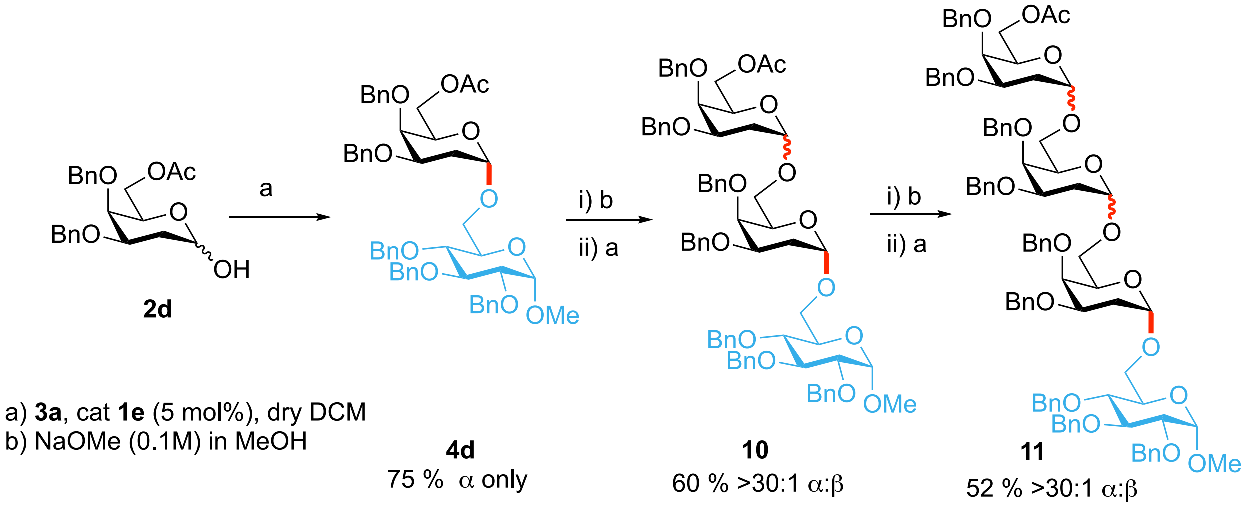

Methyl (3,4-di-*O*-benzyl-2-deoxy-α-D-lyxo-hexapyranosyl) -(1→6)-(3,4-di-*O*-benzyl-6-*O*-acetyl-2 deoxy-α-D-lyxo-hexapyranosyl)-(1→6)-2,3,4-tri-*O*-benzyl-α-D-glucopyranoside (**10**)

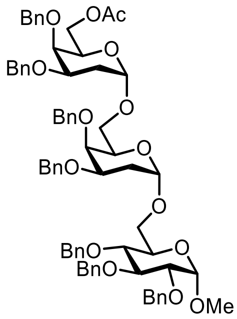

Compound **4d** (140 mg, 168 μmol) was treated with 0.1 M NaOMe in 5 ml MeOH for 1h and then it was neutralised with DOWEX 50.Then it was filtered off and washed with DCM (2 x 10 mL). The solvent was evaporated *in vacuo* to afford 130 mg of the product which was treated with hemiacetal **2b** (75 mg, 194 μmol) and catalyst **1e** (6 mg, 10 μmol) according to general glycosylation procedure A. The reaction was stirred at RT for 16 h before being concentrated *in vacuo*. Following purification using column chromatography (12:1 to 4:1 Hexane:EtOAc), **10** was afforded as a white solid (90 mg, 60% α:β >30:1).

^{1}H NMR (400 MHz, $CDCl_3$) δ 7.51 – 7.08 (m, 35H, Ar*H*), 5.00 (d, *J* = 3.2 Hz, 1H, H-1'), 4.98 (d, *J* = 10.8 Hz,1H, OCH*H*Ph), 4.92 (dd, *J* = 11.7, 4.2 Hz, 2H, OCH_2Ph), 4.86 – 4.74 (m, 3H, OCH_2Ph), 4.71 – 4.63 (m, 2H, H-1, OCH*H*Ph), 4.63 – 4.55 (m, 5H, H-1'', OCH_2Ph), 4.50 (d, *J* = 13.7 Hz, 3H, OCH_2Ph), 3.86 (ddd, *J* = 12.0, 4.6, 2.3 Hz, 1H, OCH_2Ph, H-3), 3.80 – 3.60 (m, 8H), 3.57 – 3.42 (m, 3H), 3.36 – 3.25 (m, 4H), 2.17 (td, *J* = 12.3, 3.7 Hz, 1H, H-2a'), 2.06 (qd, *J* = 12.4, 4.2 Hz, 2H, H-2a'', H-2b'), 1.90 (s, 3H, COCH_3),1.76 (dd, *J* = 12.5, 4.5 Hz, 1H, H-2b'').**^{13}C NMR**

(101 MHz, $CDCl_3$) δ 170.8 (C=O), 138.8, 138.8, 138.6, 138.5, 138.5, 138.5, 138.3, 128.6, 128.6,128.6, 128.5, 128.5, 128.4, 128.2, 128.2, 128.1, 127.8, 127.8, 127.7, 127.6, 127.3 (Ar*C*), 98.4 (C-1'), 98.1 (C-1), 97.5 (C-1''), 82.2 (C-3), 80.2 (C-2), 78.0 (C-4), 77.5, 77.4, 77.2, 76.8, 76.0, 75.0, 74.9, 74.6, 74.2, 73.4, 73.0, 72.6, 70.6, 70.5, 70.0,69.9, 68.9, 66.6 (C-4''), 66.0 (C-6'), 64.1 (C-6''), 55.3 (OCH_3), 30.9 (C-2''),30.8 (C-2') 20.9 (COCH_3).
Spectroscopic data was in agreement with previously reported literature.[6]

Methyl (6-*O*-acetyl-3,4-di-O-benzyl-2-deoxy-α-D-lyxo-hexapyranosyl) -(1→6)-(3,4-di-*O* benzyl-2-deoxy-α-D-lyxo-hexapyranosyl)-(1→6)-(3,4-di-*O*-benzyl-2-deoxy-α-D-lyxo hexapyranosyl)-(1→6)-2,3,4-tri-*O*-benzyl-α-D-glucopyranoside (**11**)

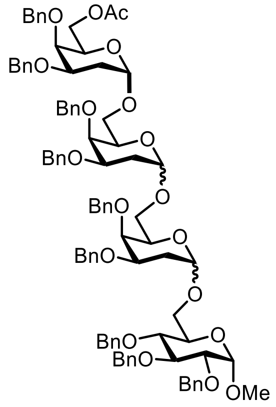

Compound 10 (90 mg, 77 μmol) was treated with 0.1 N NaOMe in 5 ml MeOH for 1h and then neutralised to pH 7 with DOWEX 50.Then it was filtered off and washed with DCM (2 x 10 mL). The solvent was evaporated *in vacuo* to afford 82 mg of the product which was treated with hemiacetal **2b** (34 mg, 88 μmol) and catalyst **1e** (3 mg, 5 μmol) according to general glycosylation procedure A. The reaction was stirred for 16 h and after purification using column chromatography (12:1 to 4:1 Hexane:EtOAc), **11** was afforded as a white solid (38 mg, 52% α:β >30:1).
^{1}H NMR (400 MHz, $CDCl_3$) δ 7.42 – 7.17 (m, 45H, ArH), 5.01 – 4.87 (m, 5H), 4.85 – 4.73 (m, 4H), 4.72 –4.46 (m, 14H), 4.11 (dd, *J* = 12.5, 6.2 Hz, 2H), 3.97 (t, *J* = 9.2 Hz, 1H), 3.92 – 3.84 (m, 3H), 3.83 –3.76 (m, 3H), 3.76 – 3.59 (m, 8H), 3.57 – 3.44 (m, 4H), 3.40 – 3.23 (m, 5H), 2.17 (dtd, *J* = 16.1, 12.3, 3.6 Hz, 2H), 2.05 (ddq, *J* = 12.2, 9.4, 4.0 Hz, 2H), 1.90 (s, 3H), 1.85 (dd, *J* = 12.7, 4.6 Hz, 1H), 1.73 (dd, *J* = 12.4, 4.4 Hz, 1H).**^{13}C NMR (101 MHz, $CDCl_3$) δ** 170.8, 138.8, 138.8, 138.7, 138.6, 138.6, 138.6, 138.4, 138.2, 128.6,128.6, 128.6, 128.5, 128.5, 128.4, 128.4, 128.4, 128.2, 128.1, 127.8, 127.8, 127.8, 127.7, 127.7, 127.7,127.6, 127.5, 127.3, 98.6, 98.1, 97.7, 97.5, 82.2, 80.1, 77.8, 77.5, 77.4, 77.2, 76.8, 76.0, 75.3, 75.1, 75.0, 74.6, 74.2, 74.1, 73.4, 72.8, 72.6, 72.5, 70.7, 70.5, 70.4, 70.0, 70.0, 69.5, 69.0, 66.5, 66.2, 66.1, 64.1, 55.2, 31.7, 31.1, 31.0, 30.9, 29.8, 25.5, 20.9.
Spectroscopic data was in agreement with previously reported literature.[7]

Mechanistic Study

^{1}H NMR titration study of the hemiacetal 2a and catalyst 1e

^{1}H NMR titration of catalyst **1e** toward to acceptor **2a** was studied by ^{1}H NMR titrations in CD_2Cl_2. 1 mmol of the catalyst **1e** was taken in CD_2Cl_2 (0.6 mL, conc 16.67 mM). Sequentially different equivalent of the acceptor was added to make the ration 1:1, 1:5, 1:10 and 1:20 (Figure S2). After each addition of the acceptor, the mixture was stirred for 15 min in rt before the ^{1}H NMR was recorded and the final volume was kept to 0.6 mL so that the concentration of the catalyst remains unchanged throughout the course of the experiment.

(a)

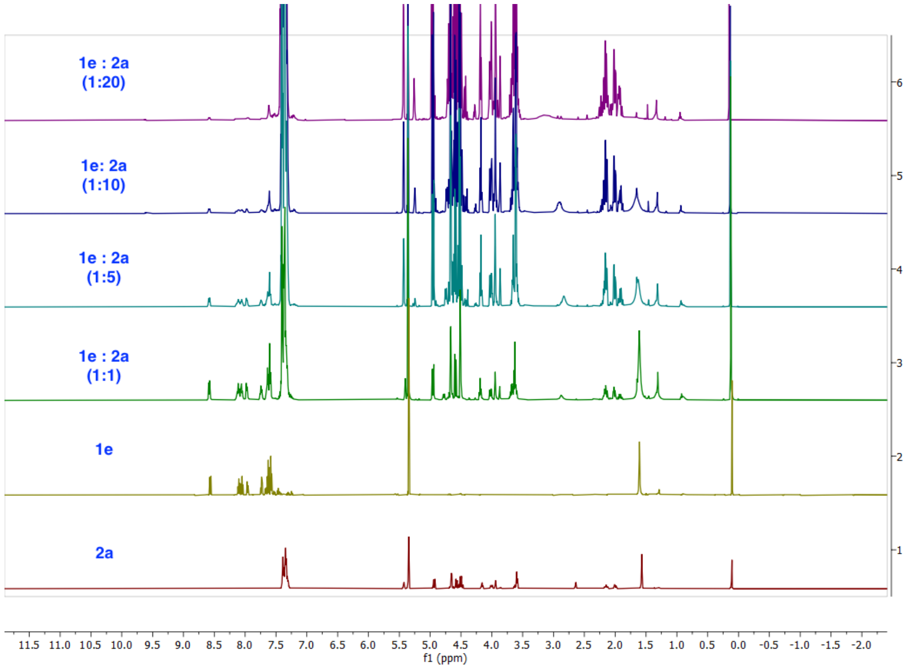

(b)

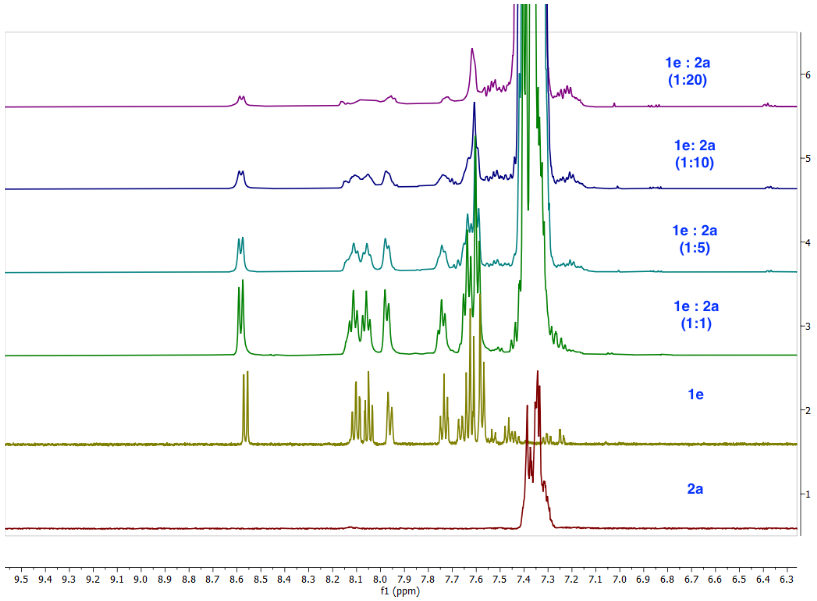

(c)

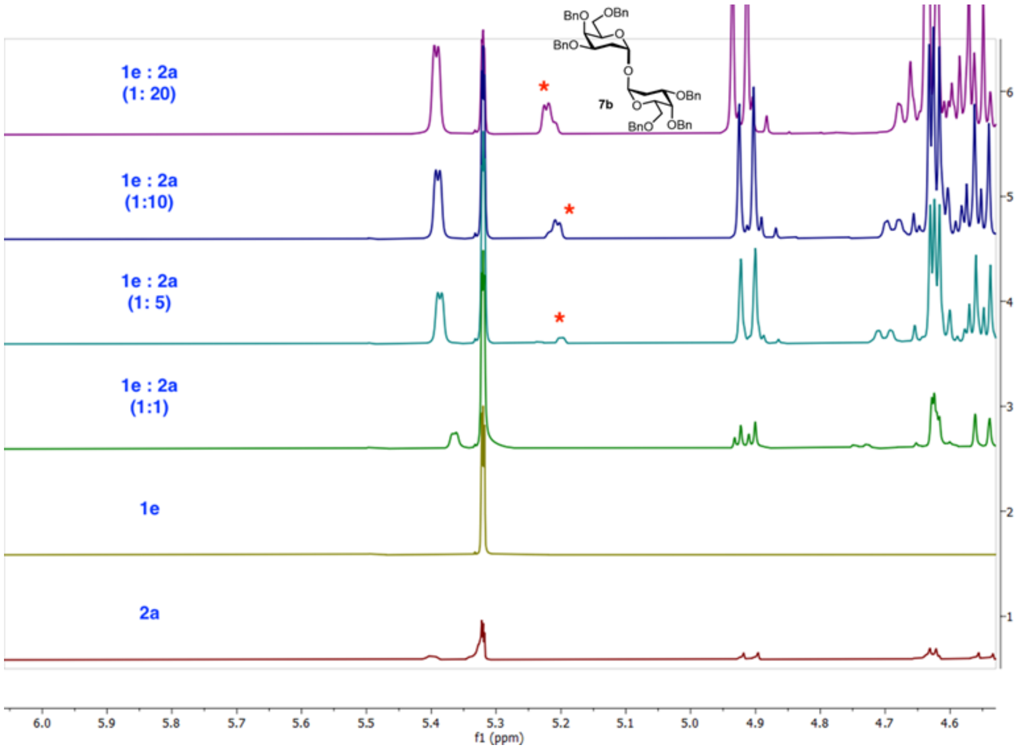

Fig S1 (a) ^{1}H NMR (298 K, CD_2Cl_2) of Cat.**1e** (16.6 mM) upon addition of different amounts of hemiacetal **2a** (b) Zoomed in aromatic region of cat. **1e** from (a). (c) Zoomed in anomeric region of dimer from (a).

^{1}H NMR titration study of the nucleophile 3a and catalyst 1e

^{1}H NMR titration of catalyst **1e** toward to acceptor **3a** was studied by ^{1}H NMR titrations in CD_2Cl_2. 1 mmol of the catalyst **1e** was taken in CD_2Cl_2 (0.6 mL, conc 16.67 mM). Sequentially different equivalent of the acceptor was added to make the ratio 1:1, 1:5, 1:10 and 1:20 (fig S2a, S2b, S2c). Every time after addition of the acceptor, the mixture was stirred for 15 min at room temperature, before the ^{1}H NMR was recorded and the final volume kept to 0.6 mL so that the concentration of the catalyst remained unchanged throughout the course of the experiment.

(a)

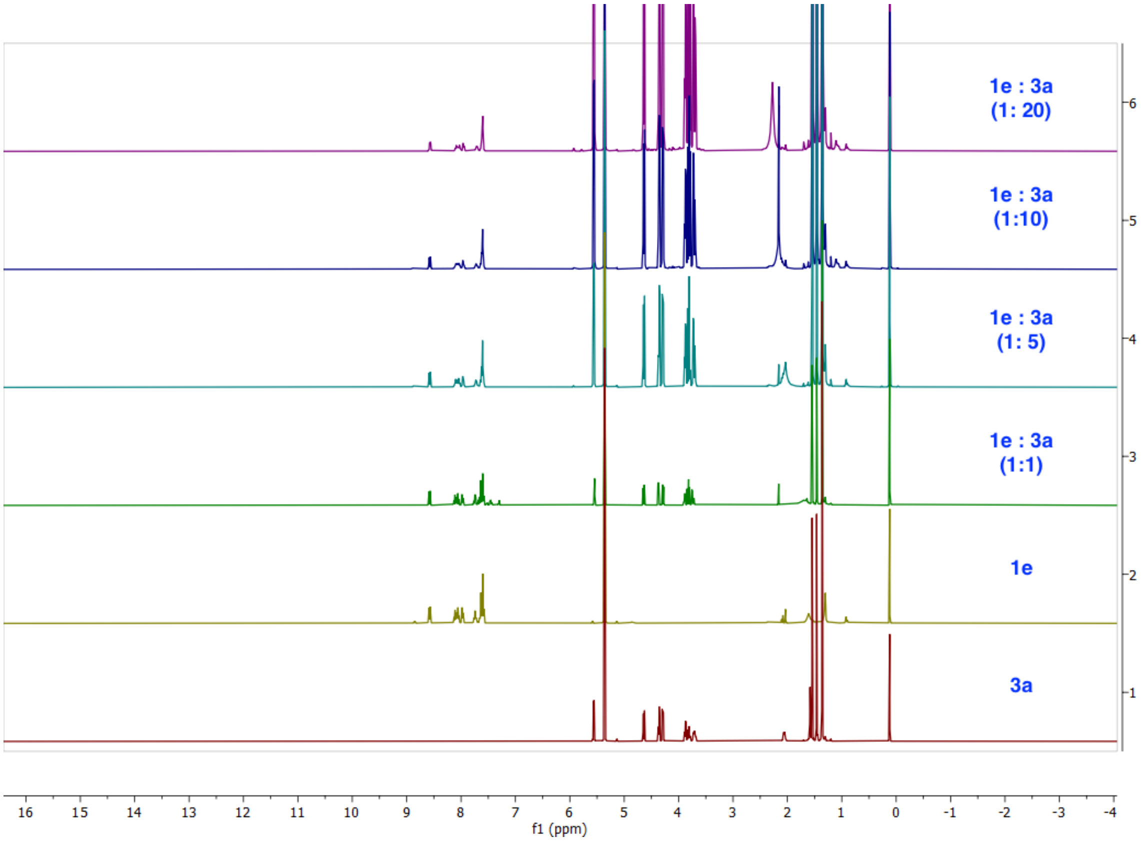

1e : 3a
(1: 20)
1e : 3a
(1:10)
1e : 3a
(1: 5)
1e : 3a
(1:1)
1e
3a
f1 (ppm)

(b)

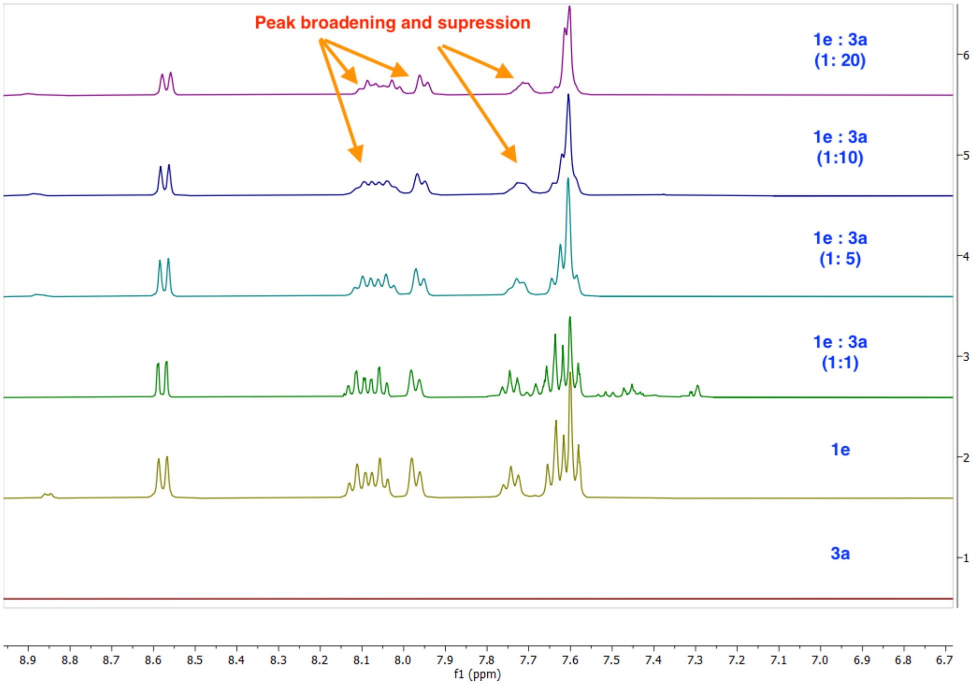

Peak broadening and supression
1e : 3a
(1: 20)
1e : 3a
(1:10)
1e : 3a
(1: 5)
1e : 3a
(1:1)
1e
3a
f1 (ppm)

(c)

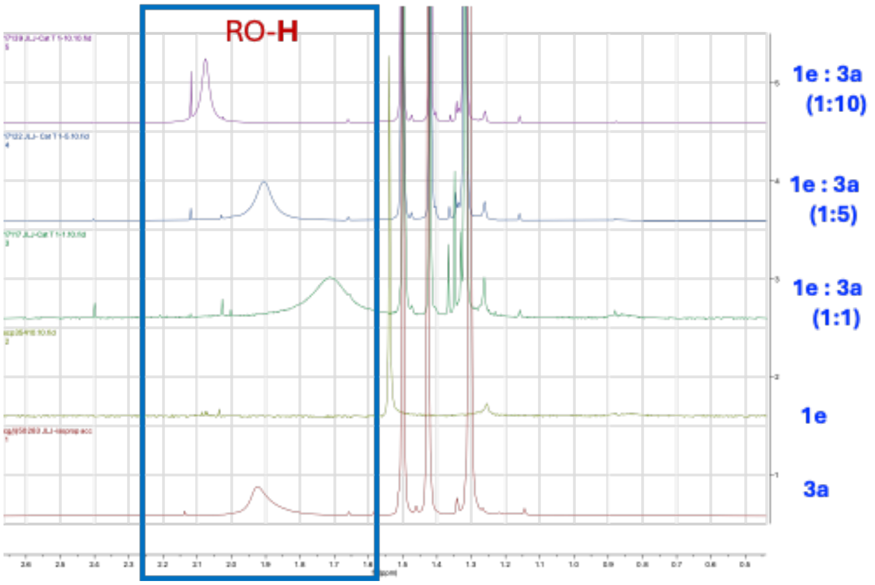

(d)

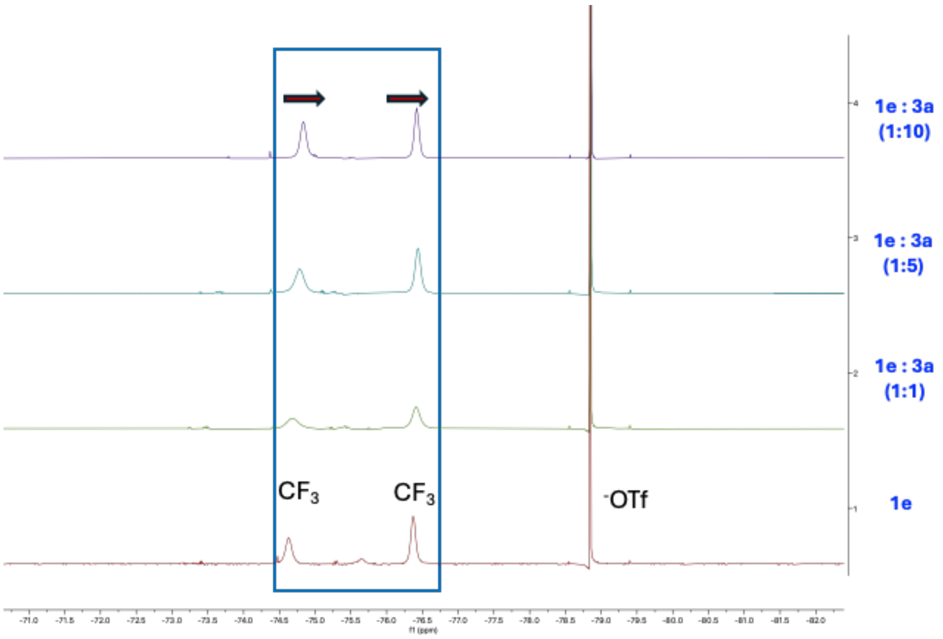

Fig S2 (a) ^1H NMR (298 K, CD_2Cl_2) of Cat.**1e** (16.6 mM) upon addition of different amounts of acceptor **3a** (b) Zoomed in aromatic region of cat. **1e**; (c) Zoomed in OH region of acceptor **3a** and (d) ^{19}F NMR (471 MHz, CD_2Cl_2) spectra of Cat.**1e** (16.6 mM) upon addition of different amounts of acceptor **3a.**

IR study between acceptor 3a and catalyst 1e.

10 mM of solution of the acceptor **3a** and 10 mM solution of the catalyst **1e** were prepared in CD_2Cl_2, and IR data was recorded (figures S3a, S3b). Then another solution of 10mM was prepared with equimolar mixture of catalyst and the acceptor and the mixture was stirred for 15 min and IR was recorded (Figure 3c).

(a) **(b)** **(c)**

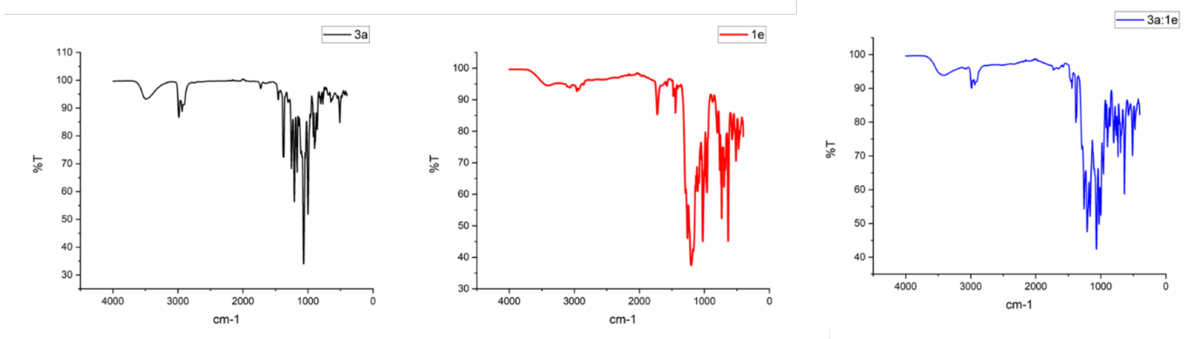

(d)

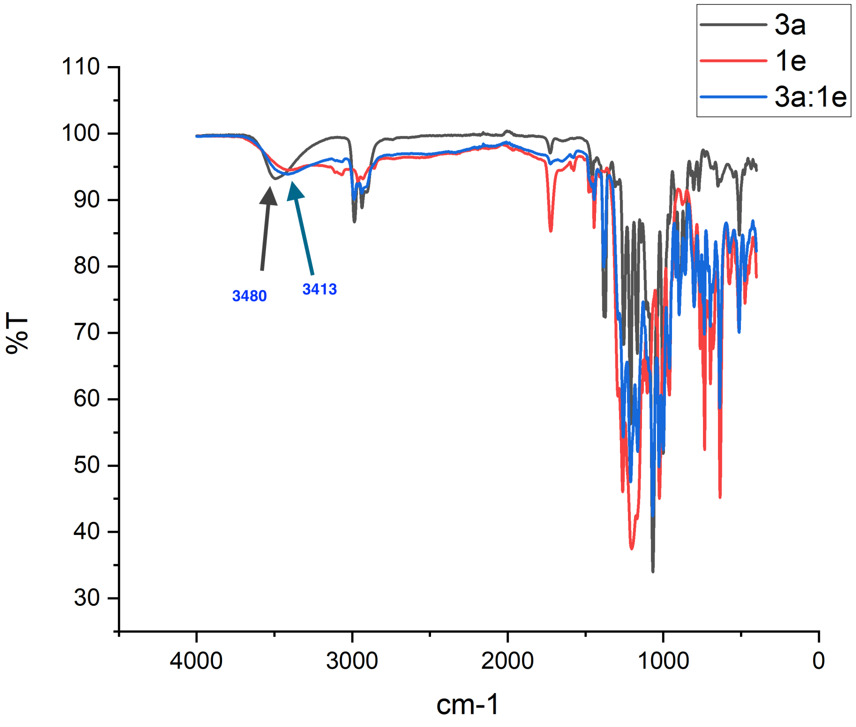

Figure S3. (a), (b) and (c) IR spectra of free acceptor, free catalyst and acceptor : catalyst mixture. (d) Superimposed spectra of (a), (b) and (c)

Kinetics Study

Standard Conditions

Table S4. Concentration of product **4a**, acceptor **3a** and donor **2a**, calculated by ^{1}H NMR under standard conditions (CD_2Cl_2 as solvent).

Time, min	Acceptor (3a), mmol/mL	Product (4a), mmol/mL	Donor (2a), mmol/mL
0	0.030000	0.00000	0.040000
10	0.029700	0.000300	0.039612
20	0.027900	0.002100	0.035396
30	0.026550	0.003450	0.032520
40			
50	0.024390	0.005610	0.027972
60	0.023610	0.006360	0.026488
70	0.022890	0.007080	0.025000
80	0.022221	0.007776	0.023668
90	0.021582	0.008415	0.022988
100	0.021126	0.008871	0.022096
110			
120	0.020268	0.009729	0.020200
130	0.019866	0.010131	0.019604
140			
150	0.019407	0.010890	0.018096
160	0.018867	0.011130	0.017776
190			
200	0.018291	0.011706	0.016804
210	0.017964	0.012033	0.016460
220	0.017856	0.012141	0.015872
230	0.017541	0.012456	0.015324
240	0.017340	0.012657	0.014856
250	0.017241	0.012759	0.014760
260	0.017046	0.012954	0.014596
270	0.016854	0.013146	0.014336
280	0.016665	0.013332	0.014184
290	0.016482	0.013515	0.013744
300	0.016302	0.013695	0.013604
310	0.016128	0.013869	0.012616
320			

330	0.015789	0.014208	0.012192
340	0.015705	0.014292	0.011904
360	0.015543	0.014457	0.011692
370	0.015462	0.014535	
380	0.015306	0.014694	0.011296
390	0.015150	0.014847	0.011204

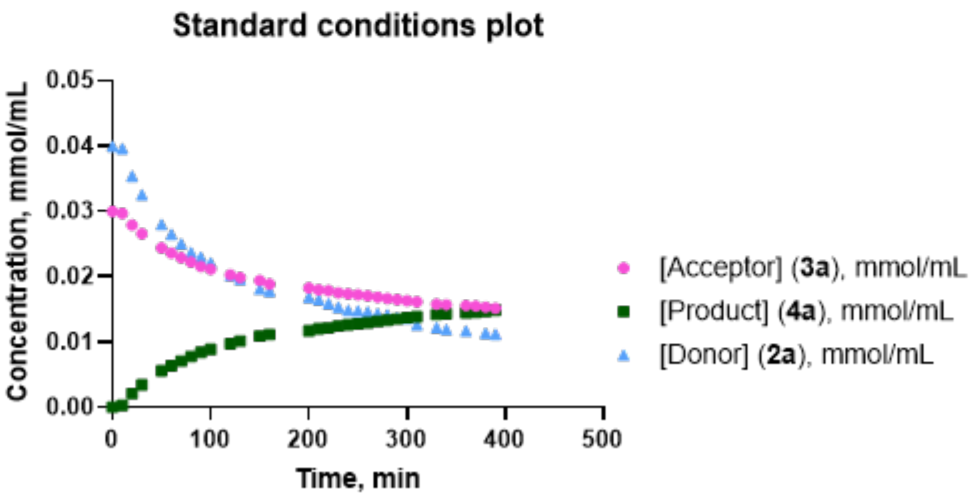

Figure S4. Kinetics profile for product and the reagents at standard conditions.

Acceptor's Concentration Dependence

Table S5. Concentration for product **4a** calculated by ^{1}H NMR under standard condition (CD_2Cl_2 as solvent) with different concentration of acceptor **3a**.

Time, min	[4a], mmol/mL using 0.3 mmol/mL 3a	[4a], mmol/mL using 0.45 mmol/mL 3a	[4a], mmol/mL using 0.6 mmol/mL 3a
0	0	0	0
10	0.002070	0.002844	0.003924
20	0.003390	0.004680	0.006900
30		0.006255	
40	0.005520	0.007605	0.010410
50	0.006420	0.008775	0.011610
60	0.007080	0.009765	

70	0.007710		0.014196
80	0.008400		0.014886
100	0.008700	0.011385	0.016524
110		0.012150	0.017142
120	0.009630	0.012690	0.017742
130	0.010140	0.013140	0.018330
140			0.019182
150	0.010740	0.014130	0.019728
160	0.011070	0.014715	0.019998
170			0.020262
180	0.011670	0.015210	0.020784
190	0.011850	0.015795	0.021036
200	0.012060		0.021288
210	0.012390	0.016425	0.021780
220	0.012600	0.015570	0.022020
230	0.012810		0.022728
240	0.012930	0.017235	
250	0.012990	0.017505	0.023190
260	0.013260	0.017775	
270	0.013440	0.017820	0.023412
280	0.013380	0.018090	
290	0.013830	0.018270	0.023634
300		0.018720	0.024066
310	0.014070	0.018810	0.024288
320	0.014280	0.018810	
330	0.014430	0.018990	
340	0.014430	0.019080	
350	0.014550	0.019305	
360	0.014640	0.019485	

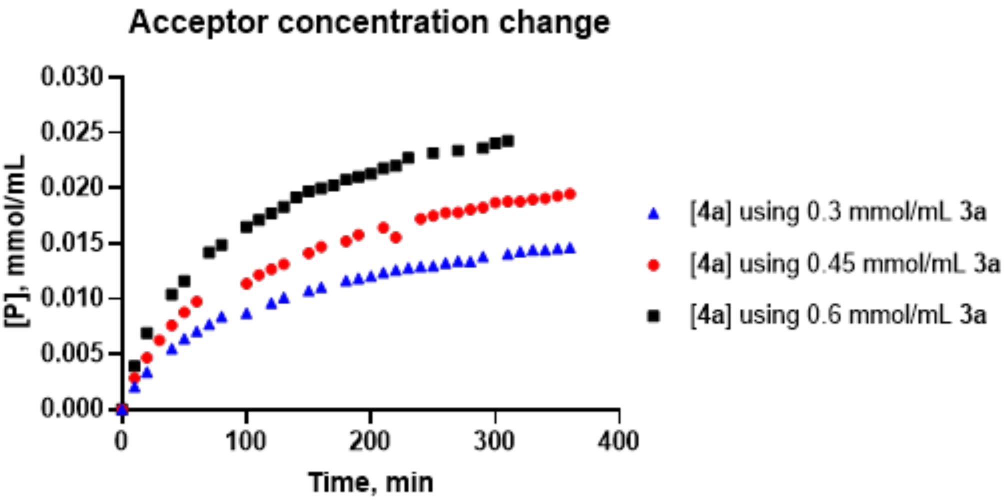

Figure S5. Kinetics profile for different concentration acceptor **3a**.

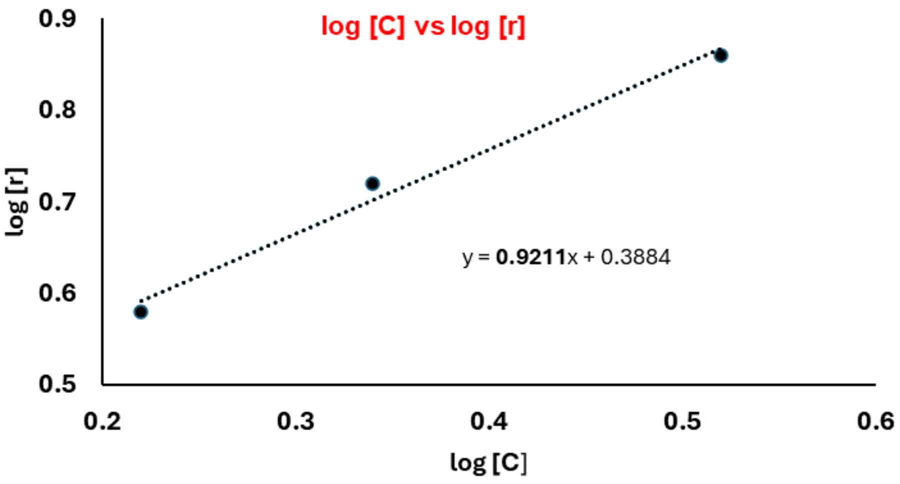

Figure S6. Plot between of logarithm of rate and concentration of acceptor **3a**.

Donor's Concentration Dependence

Table S6. Concentration of product **4a** calculated by ^{1}H NMR under standard condition (CD_2Cl_2 as solvent) with different concentration of hemiacetal **2a**.

Time, min	[4a], mmol/mL using 0.04 mmol/mL 2a	[4a], mmol/mL using 0.06 mmol/mL 2a	[4a], mmol/mL using 0.08 mmol/mL 2a

0	0	0	0
10	0.004260	0.002070	0.00522
20	0.006630	0.003390	0.00801
30	0.008310	-	0.00993
40	0.009780	0.005520	0.01137
50	0.010890	0.006420	0.01248
60	0.011760	0.007080	0.01344
70	0.012510	0.007710	0.01428
80	0.013320	0.008400	0.015
90		0.008700	0.01554
100	0.014070	-	0.01611
110	0.014760	0.009630	0.01656
120	0.015240	0.010140	
130	0.015630	-	0.01734
140	0.015870	0.010740	
150	0.016230	0.011070	0.01806
160	0.016470	-	
170	0.016770	0.011670	0.01851
180	0.017040	0.011850	0.01887
190	0.017250	0.012060	0.01899
200	0.017550	0.012390	
210	0.017760	0.012600	0.01947
220	0.017850	0.012810	0.01962
230	0.017970	0.012930	0.01986
240	0.018180	0.012990	0.01998
250	0.018390	0.013260	0.02028
260	0.018630	0.013440	0.02028
270	0.018750	0.013380	
280	0.018870	0.013830	0.0204
290	0.018990	-	0.02055
300	0.019230	0.014070	0.02082
310	0.019230	0.014280	0.02082
320	0.019350	0.014430	0.02097
330	0.019470	0.014430	0.02112
340	0.019590	0.014550	0.02112
350	0.019590	0.014640	0.02112

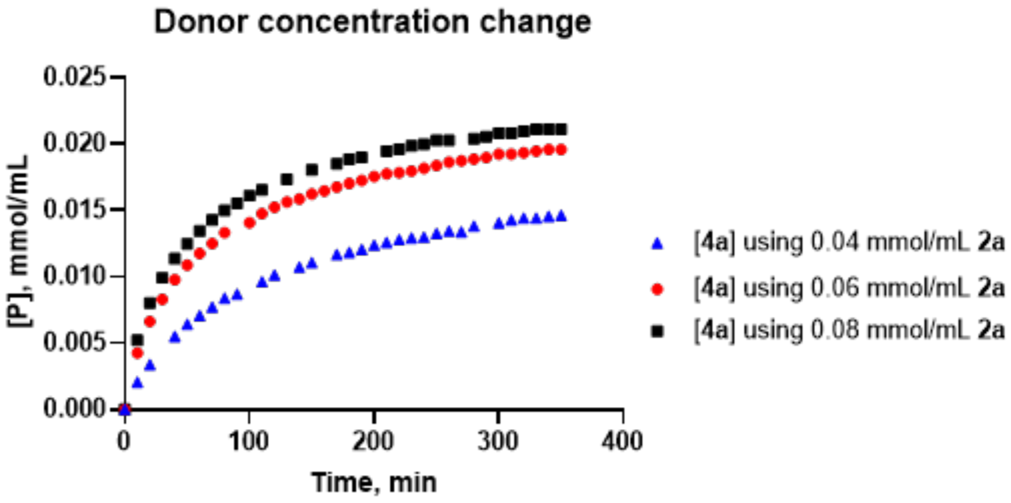

Figure S7. Kinetics profile for different concentration donor **2a**

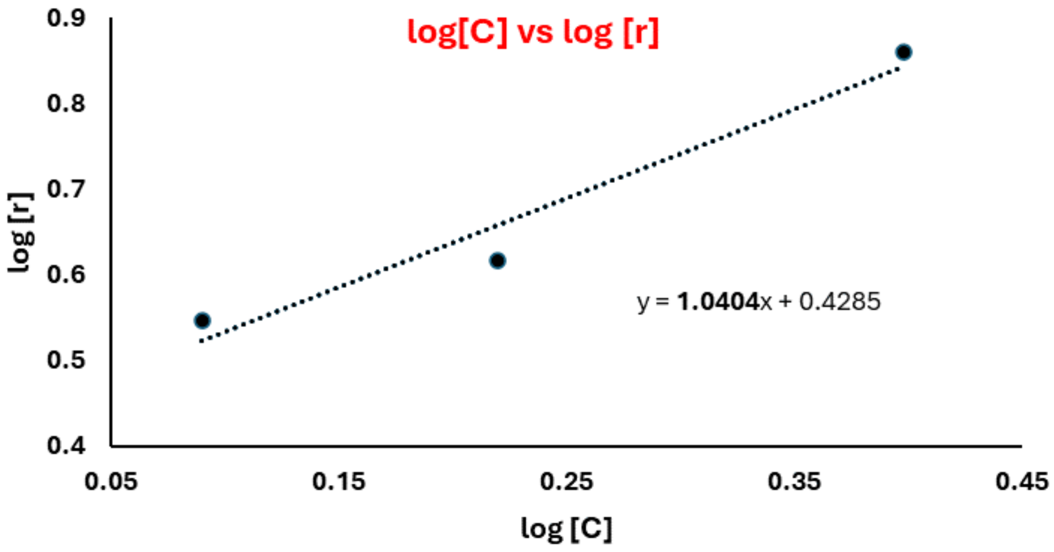

Figure S8. Plot between of logarithm of rate and concentration of donor **2a**.

Catalyst's Concentration Dependence

Table S7. Concentration for product **4a** calculated by ^{1}H NMR under standard condition (CD_2Cl_2 as solvent) with different concentration of the catalyst **1e**.

Time, min	[4a], mmol/mL using 1.45 µmol/mL 1e	[4a], mmol/mL using 2.18 µmol/mL 1e	[4a], mmol/mL using 2.90 µmol/mL 1e
0	0	0	0
10	0.003105	0.004151	0.003825
20	0.005085	0.006598	0.006525
30	-	0.008036	0.008505
40	0.008280	0.009719	0.010305
50	0.009630	0.010817	0.011655
60	0.010620	0.011780	0.013995
70	0.011565	0.012569	0.014715
80	0.012600	0.013235	0.015480
90	0.013050	0.013719	0.016380
100	-	0.014331	0.017100
110	0.014445	-	0.017460
120	0.015210	0.015306	0.018090
130	-	-	0.018360
140	0.016110	0.016014	0.018990
150	0.016605	-	0.019485
160	-	0.016483	0.019845
170	0.017505	0.016791	0.020160
180	0.017775	0.017110	0.020745
190	0.018090	0.017175	0.020925
200	0.018585	0.017441	0.021240
210	0.018900	0.017647	0.021510
220	0.019215	0.017857	0.021825
230	0.019395	0.018000	0.022050
240	0.019485	0.018072	0.022185
250	0.019890	0.018145	0.022500
260	0.020160	-	0.022860
270	0.020070	0.018519	0.022950
280	0.020745	-	0.022950
290	-	0.018672	0.023175
300	0.021105	0.018828	0.023445
310	0.021420	0.018908	-
320	0.021645	0.018907	0.023670
330	0.021645	-	0.024075
340	0.021825	0.019149	0.024210
350	0.021960	0.019231	0.024345

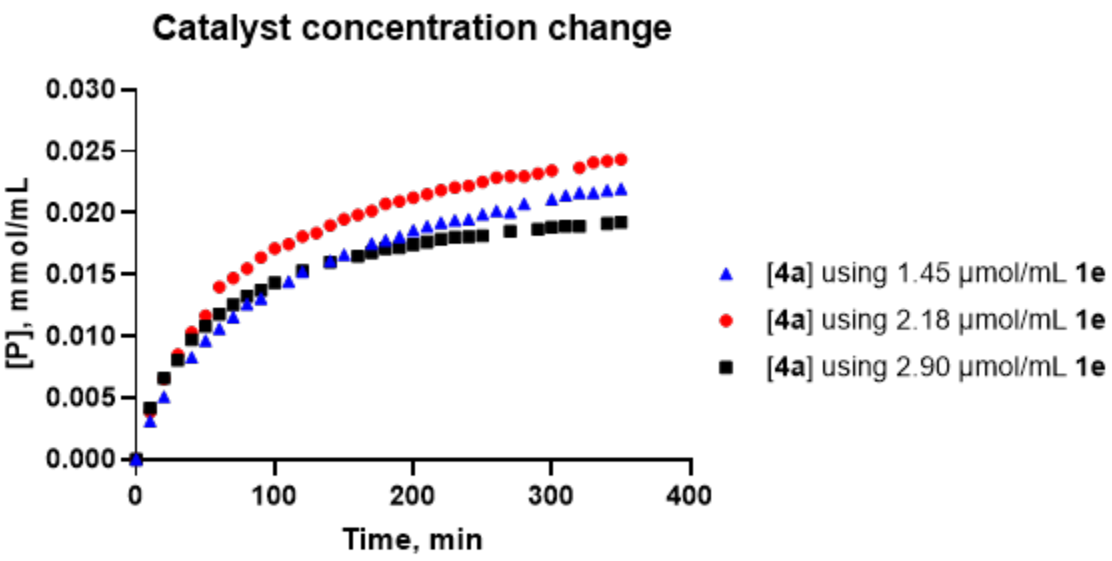

Figure S9. Kinetics profile for different concentration catalyst **1e**.

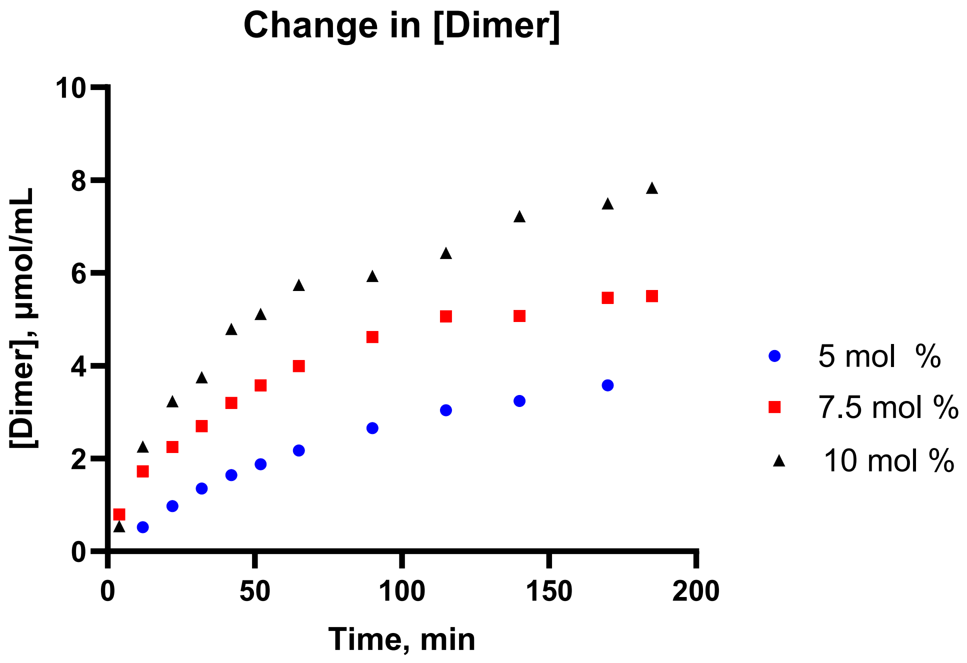

Figure S10. Kinetics profile for the concentration of dimerized hemiacetal **7b** with changing concentration of catalyst.

^{1}H-NMR reaction monitoring reveals H-shifts associated to donor and acceptor inetreactions with the catalyst.

Superimposing the ^{1}H NMR spectra taken during the kinetic monitoring of the reaction between hemiacetal **2a** and acceptor **3a** reveals a shift in ppm for the signals of both **2a** and **3a** over time, while a less significant shift was observed for product **4a**. The anomeric signals

for hemiacetal **2a** exhibit the largest change in ppm, with the beta anomer shifting 0.016 ppm while the alpha anomer shifted 0.0041 ppm.

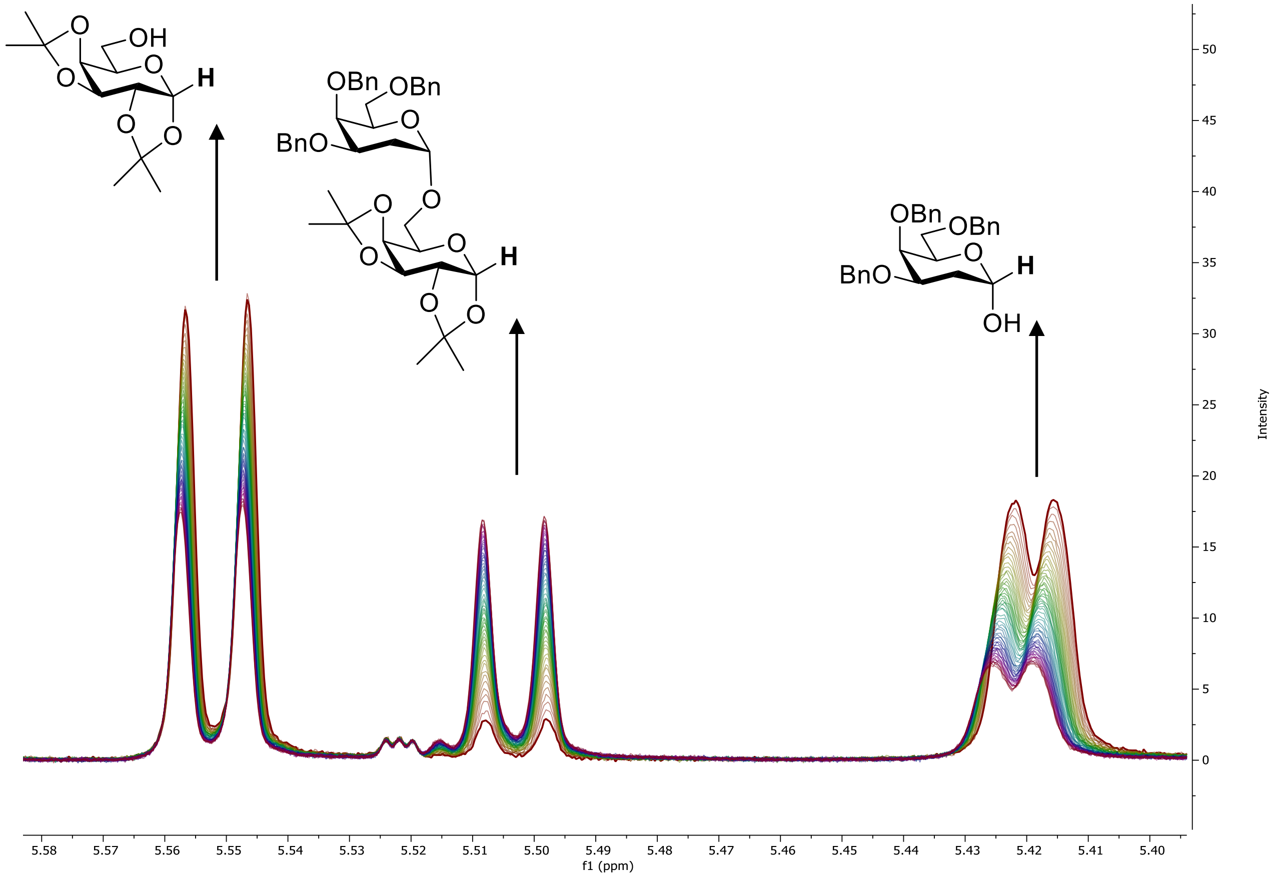

Figure S11. Three shifts associated to anomeric signals for **3a** (5.55 ppm), **4a** (5.50 ppm) and **2a** (5.42 ppm) being consumed/produced over time visualised by the superimposition of kinetics ^{1}H spectra.

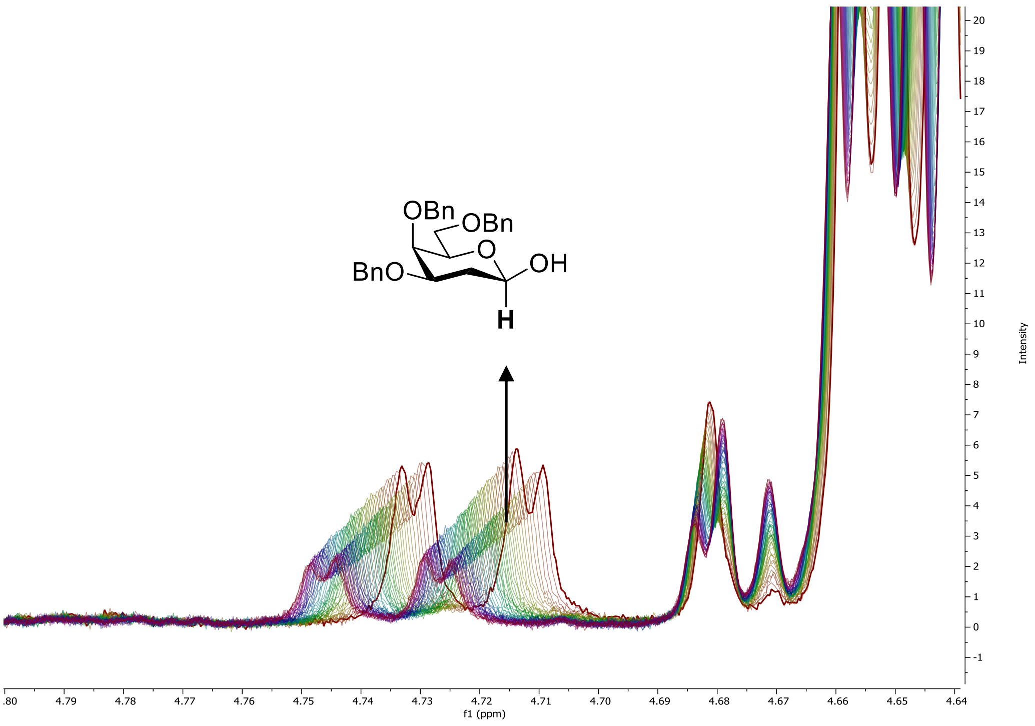

Figure S12. Anomeric peak of the beta anomer of **2a** (4.73 ppm) being consumed over time.

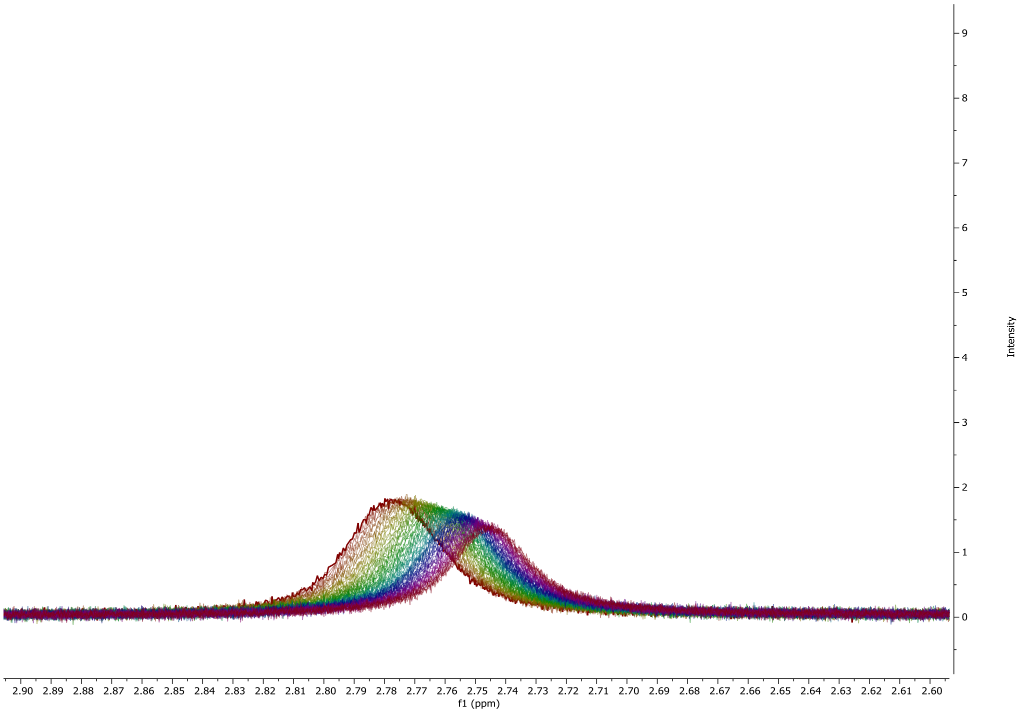

Figure S13. Expansion showing the shift of OH signal over the course of the reaction run at standard conditions (superimposed spectra)

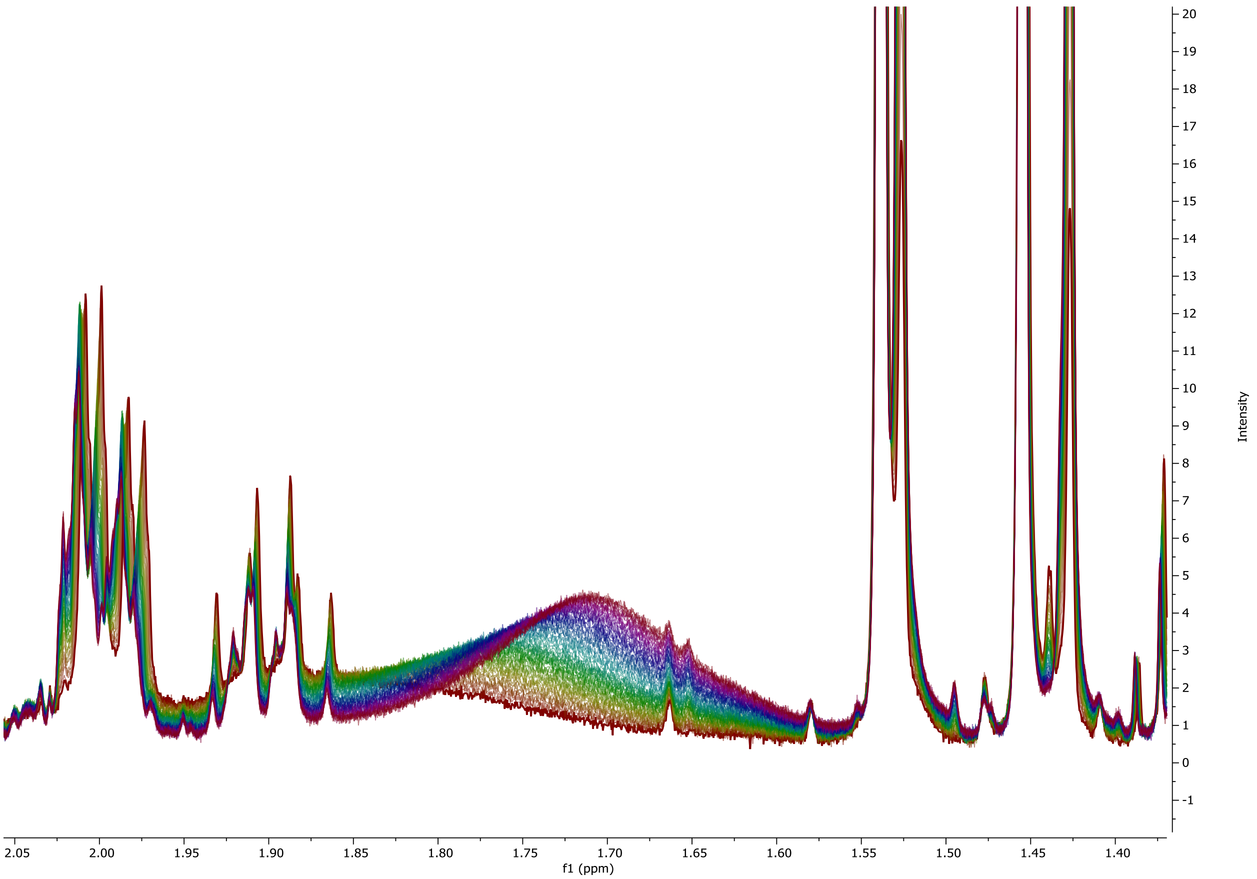

Figure S14. Production of H_2O over the course of the kinetic run at standard conditions visualised by the superimposition of ^{1}H NMR spectra.

Deuterium kinetic study

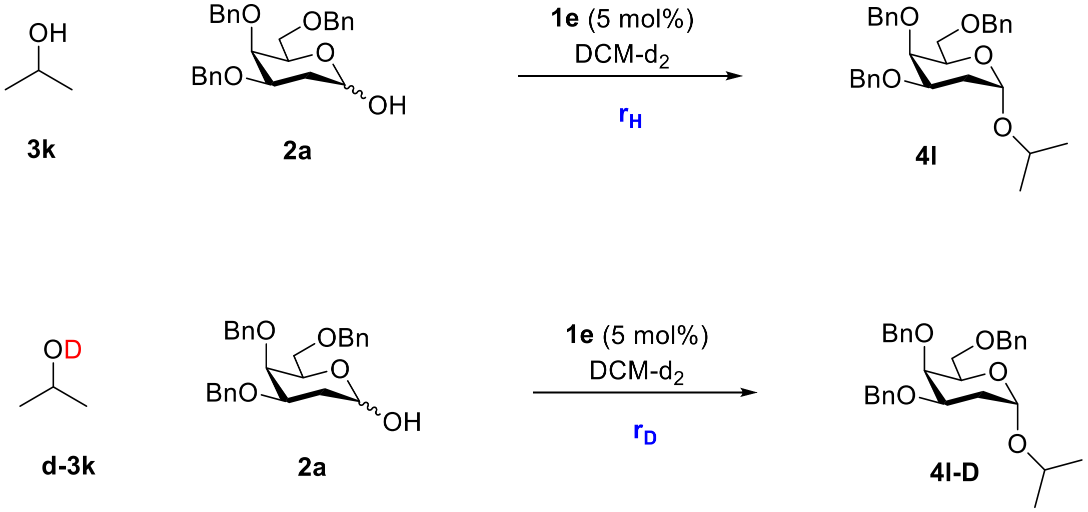

Scheme S2. Comparative kinetic study between isopropanol and deuterated isopropanol.

Table S9. Concentration for product **4l** and **4l-D** calculated by ^{1}H NMR under standard condition (CD_2Cl_2 as solvent).

Time, min	[4l], μmol/mL	[4l-D], μmol/mL
0	0	0
2	1.5318	0.7252
4	2.6566	0.9879
6	3.6223	1.4282
8	4.5288	1.7575
10	5.1541	2.1571
12	5.9311	2.5160
14	6.5009	2.8823
16	7.1299	3.1783
18	7.6257	3.4891
20	8.1326	3.8221
22	8.4434	4.1329
24	8.9614	4.3771
26	9.4054	4.6731
28	9.8124	4.9691
30	10.1195	5.2614
32	10.4747	5.4686
34	10.7522	5.7979
36	11.1148	6.0384
38	11.3516	6.2900
40	11.6180	6.4824
42	11.9029	6.8746
44	12.1397	6.9930
46	12.3839	7.3815
48	12.6059	7.4518
50	12.8834	7.6775
52	13.0647	7.7404
54	13.1905	8.0549
56	13.5013	8.2843
58	13.6900	8.5803
60	13.8084	8.7727
62	13.8898	8.9170
64	14.0341	9.0428
66	14.3560	9.3721
68	14.5891	9.5756
70	14.6520	9.6866
72	14.8481	9.9826

74	14.9739	10.0973
76	15.0775	10.3304
78	15.1848	10.5376
80	15.3402	10.7448
82	15.4327	10.9890
84	15.5030	11.1333
86	15.7398	11.1888
88	15.8138	11.2998
90	15.9063	11.5958
92	16.0950	11.7549
94	16.1690	11.8770
96	16.2837	11.9584

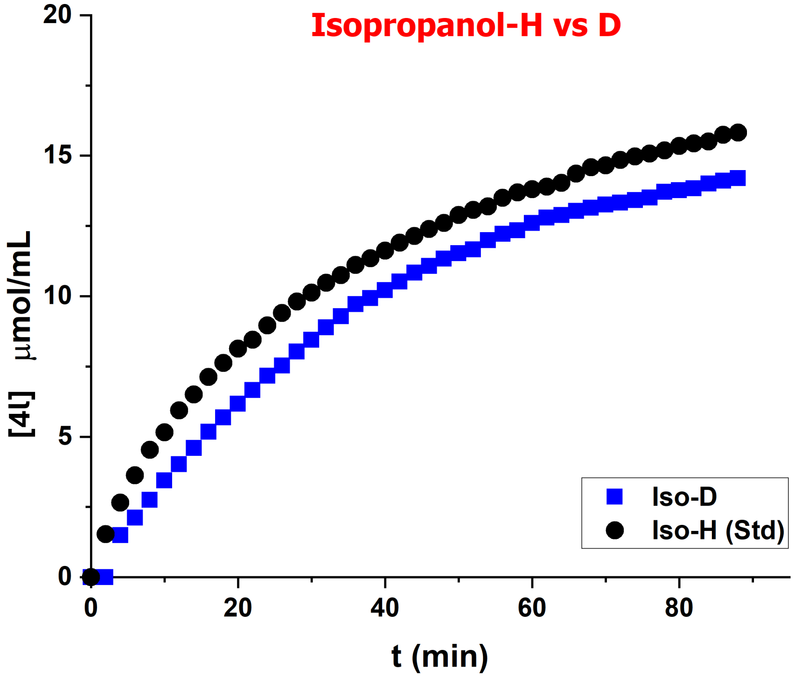

Figure S15. Kinetics profile for isopropanol and deuterated isopropanol under initial rate conditions.

Deuterated and protonated hemiacetal kinetic profiles

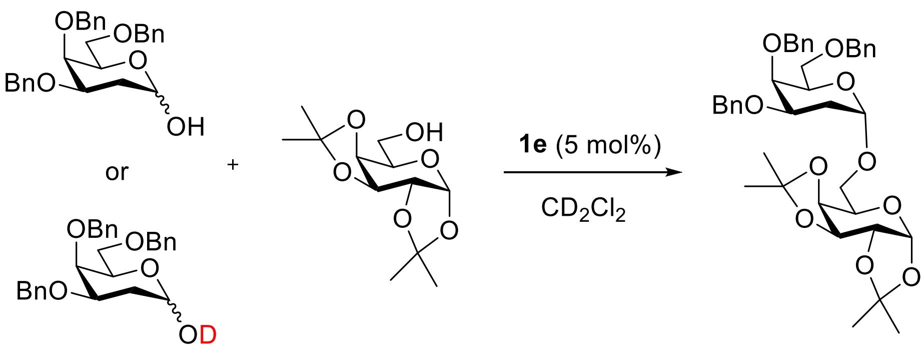

Scheme S3. Comparative kinetic study between **2a** and deuterated **2a**.

Two hemiacetal donors with either protonated or deuterated hydroxyls at C-1 were prepared and compared directly in the model reaction using time sequenced ^1H NMR (Scheme S2). Product formation was monitored over time by analysis of the integrations of newly forming peaks. The deuterated substrate was seen to react with a slower rate (0.02239 $mmols^{-1}$ for the deuterated reaction vs 0.02823 $mmols^{-1}$ for the standard). rH/rD is calculated to be 1.26, showing the difference in reaction profile. This result shows the presence of a primary kinetic isotope effect, revealing the importance of the hemiacetal proton abstraction by the catalyst **1e** in the collapse of TS3.

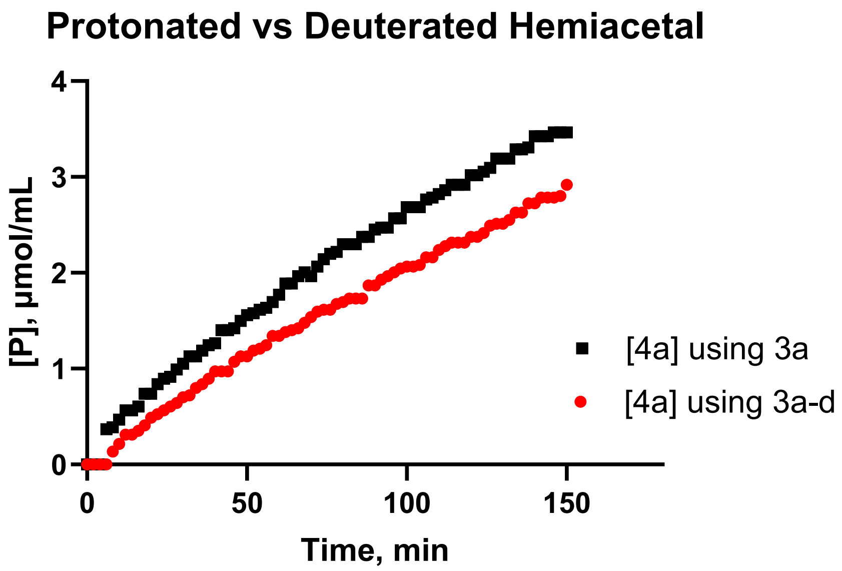

Figure S16. Kinetic profiles of model reaction run with deuterated and protonated OH hemiacetals

Control experiments with base

5 mol % both DTBP was added to the reaction between **2a** and **3a** in the presence of **1e** under the optimised standard conditions, 28% overall conversion was observed in 24 hours. When DTBP was added in stoichiometric amounts, no product was observed (Scheme S3).

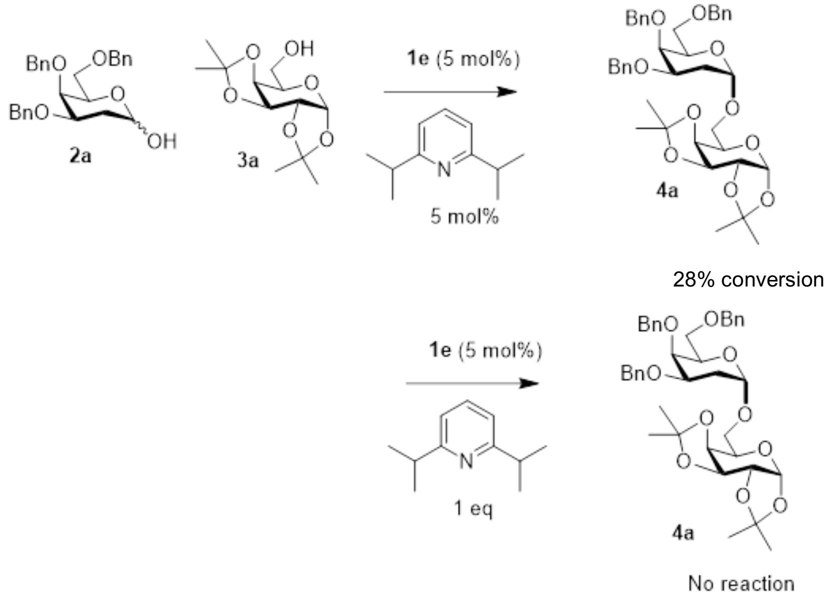

Scheme S4. Catalyst poisoning experiment with DTBP

Control experiment with TfOH

A model glycosylation reaction using hemiacetal **2a** and acceptor **3a** was tested following general glycosylation procedure A, but with alternate acid additives used instead of catalyst **1e**. Both 5 mol% and 1 mol% TfOH were tested, as well as 5 mol% trifluoroacetic acid. When 5 mol% TfOH was used the reaction produced an inseparable mixture of compounds aswell as significant degradation of starting materials. 5 mol% TFA produced no reaction, and 1 mol% TfOH showed some product formation, however significant amounts of hemiacetal dimerization was also observed along with other side products (Figure S14). The anomeric signals of product **4a** and dimer **7b** can be seen at δ 5.05 and 5.28 ppm respectively. We conclude that using 1 mol% gives lower product yields and more byproduct formation than catalyst **1e** and is not an effective catalyst to this reaction.

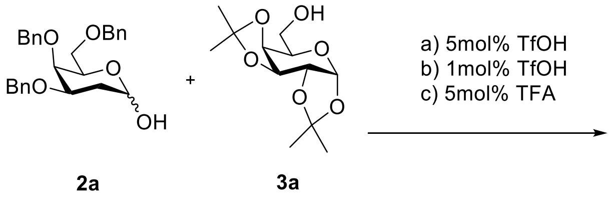

Scheme S5: Control reactions with acids TfOH and TFA.

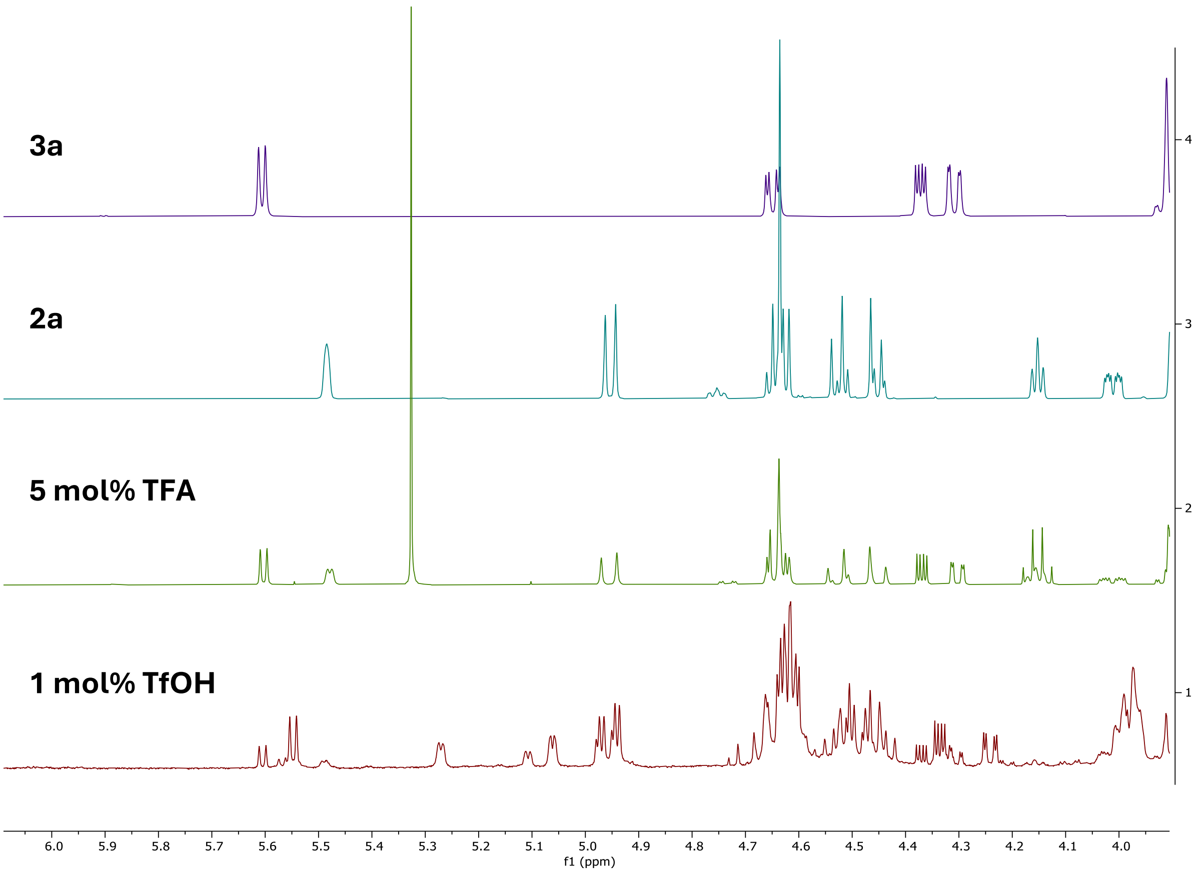

Figure S17. Stacked ^{1}H NMR spectra of starting materials **3a** and **2a**, and the anomeric region of crude reactions with 5 mol% TFA, and 1 mol% TfOH.

Anomerisation studies

An α:β mixture of disaccharide **4k** was subjected to the standard reaction conditions in the presence and absence of a nucleophile (MeOH) to assess whether anomerization can take place during the reaction (Figure S15). Only starting material or hydrolised furanose hemiacetal was observed. No evidence of anomerisation of the disaccharide starting material or formation of a methyl glycoside product **4b** (in the latter experiment) was found.

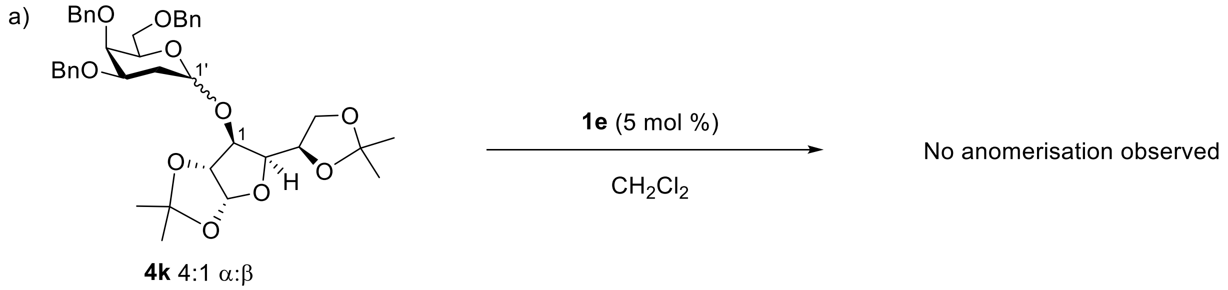

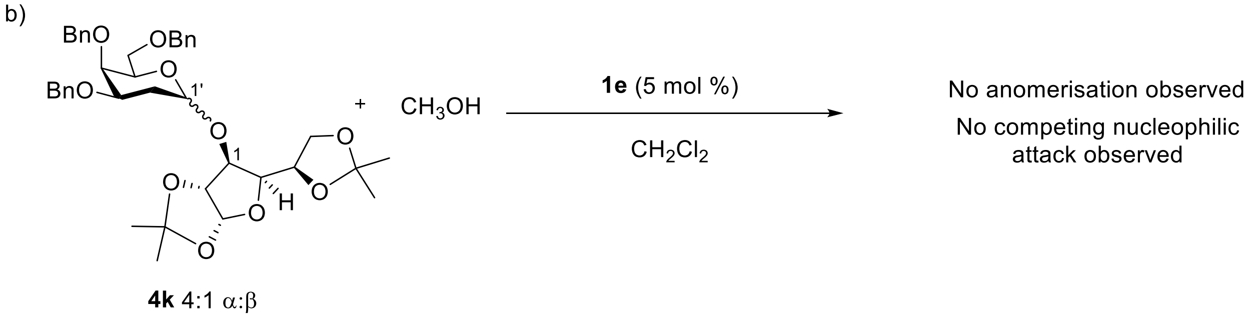

Scheme S6. a) Anomerisation experiment carried out on an α:β mixture formed using this methodology b) Mixture subject to reaction conditions with additional nucleophile to observe possible competing reactivity

Upon treatment of catalyst **1e**, the starting material reacts to form a new species, as shown in figures S18-S19. New signals in the ^{1}H NMR spectra of the crude reaction mixtures can be seen clearly at δ 5.97, 5.93, 5.05 and 4.81, as well as low intensity signals from residual starting material. The H-1 α and H-1 β signals of **4k** can be seen at 5.83 and 5.99 ppm respectively. The products of these reactions were not able to be isolated due to the scale of the reaction, however the new peak at 5.83 ppm corresponds to the anomeric of a furanose hemiacetal formed from hydrolysis of the glycosidic bond,[8] likely due to the acidic conditions and moisture present during the reaction.

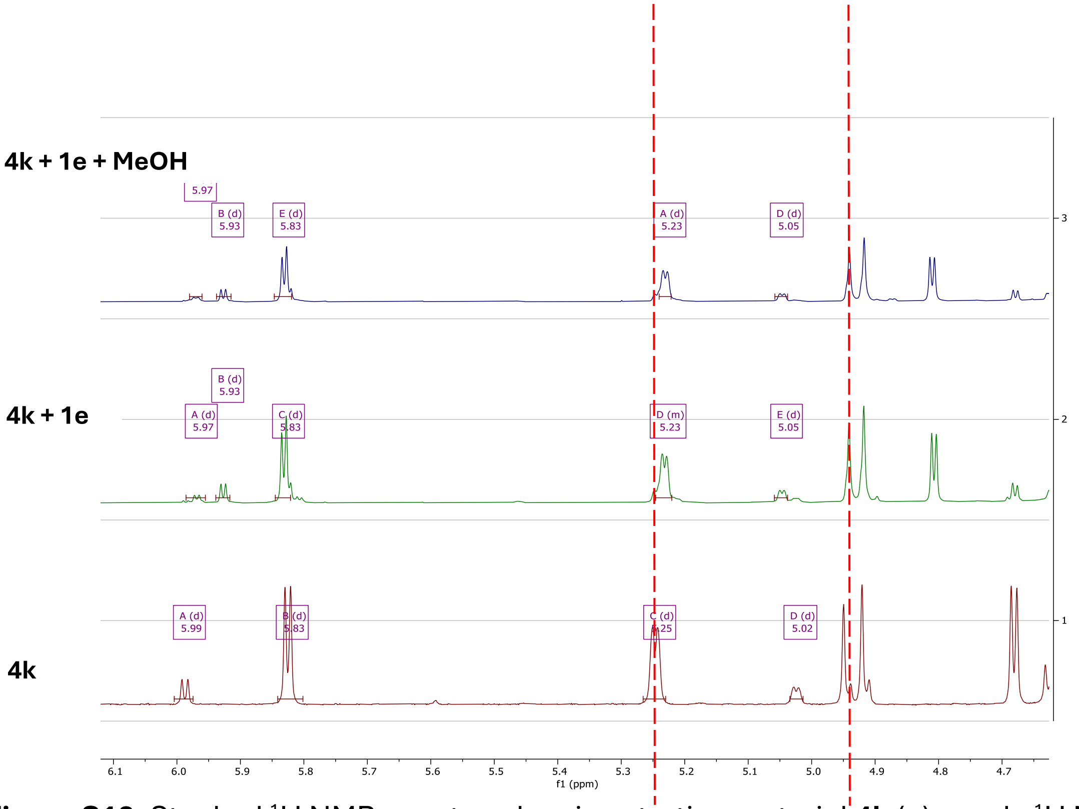

Figure S18. Stacked ^1H NMR spectra showing starting material **4k** (a), crude ^1H NMR spectra of the reaction of **4k** with catalyst **1e** (b) and **4k** with catalyst **1e** and methanol (c). All spectra are referenced to the $CDCl_3$ residual peak.

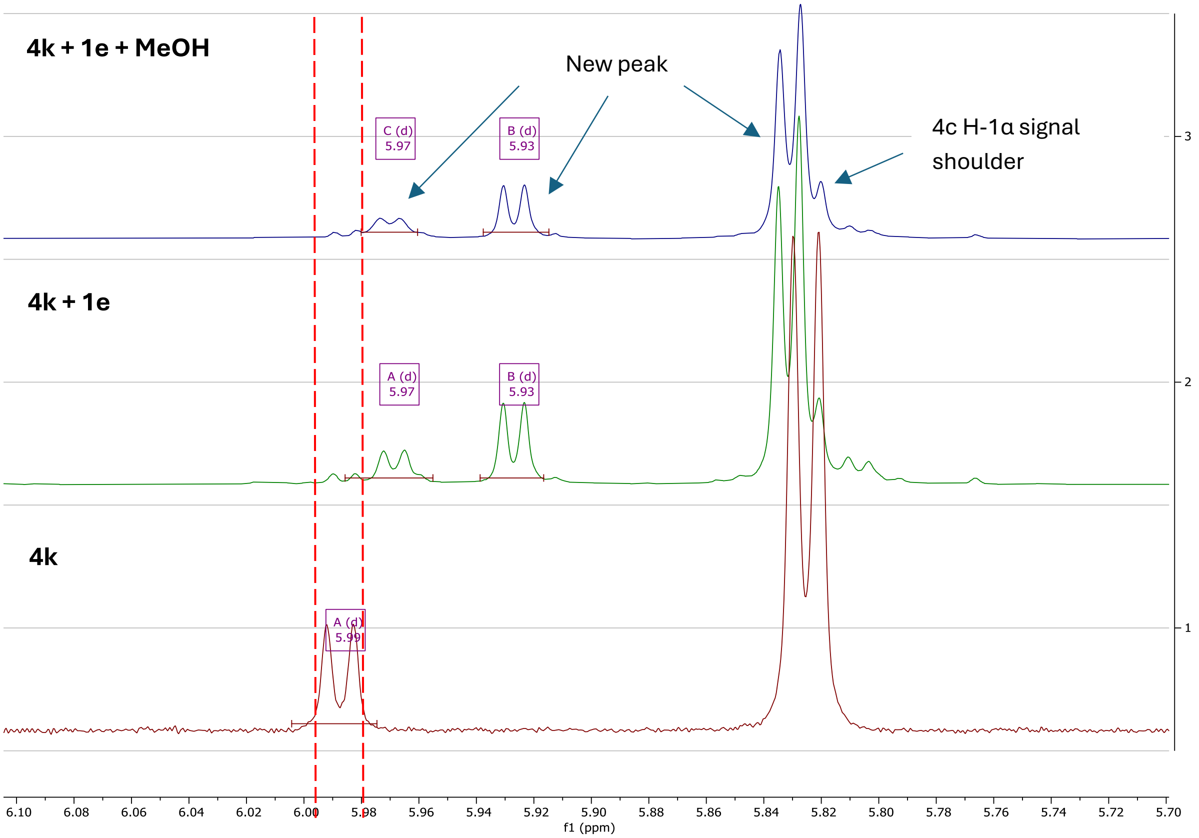

Figure S19. Stacked ^{1}H spectra, zoomed in around anomeric peaks showing the new signals and reduction in starting material peaks.

NMR investigations on the anomeric selectivity of the trehaloside reaction.

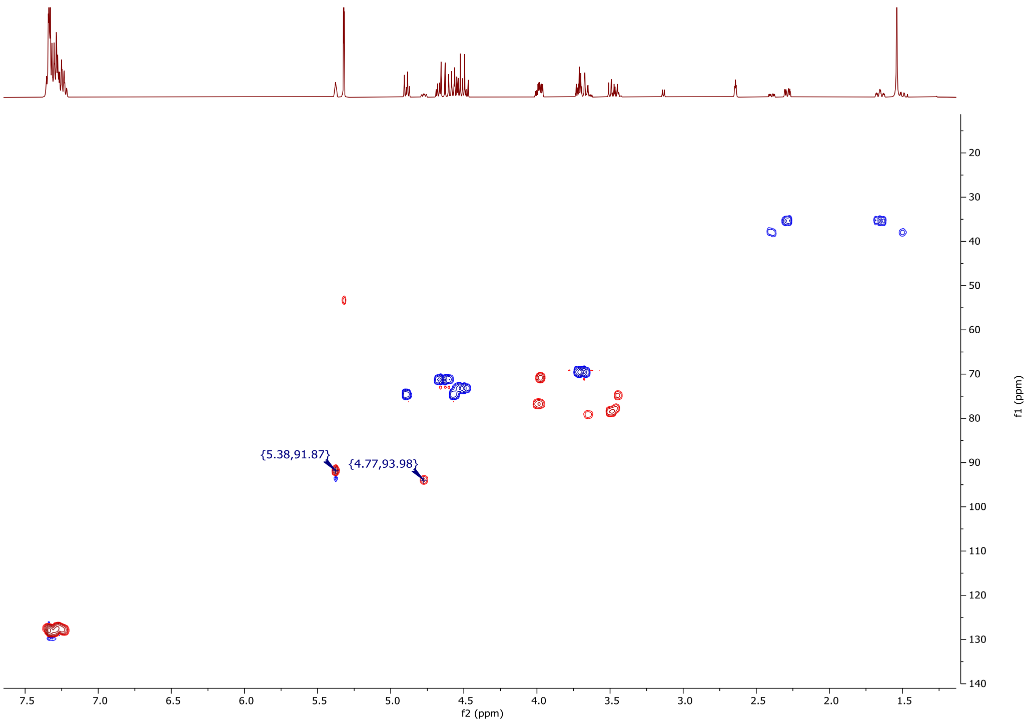

Figure S20. HSQC spectra of 2-deoxyglucose hemiacetal **2e**, alpha (5.38 ppm) and beta (4.77 ppm) anomers highlighted.

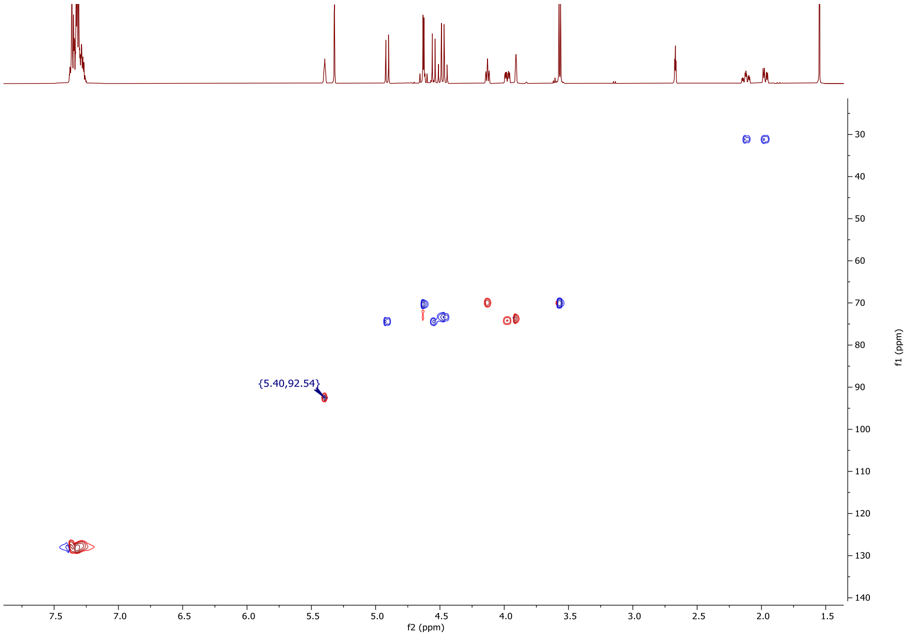

Figure S21. HSQC spectra in CD_2Cl_2 of 2-deoxygalactose hemiacetal **2a**, only alpha anomer (5.40 ppm) observed in HSQC.

A)

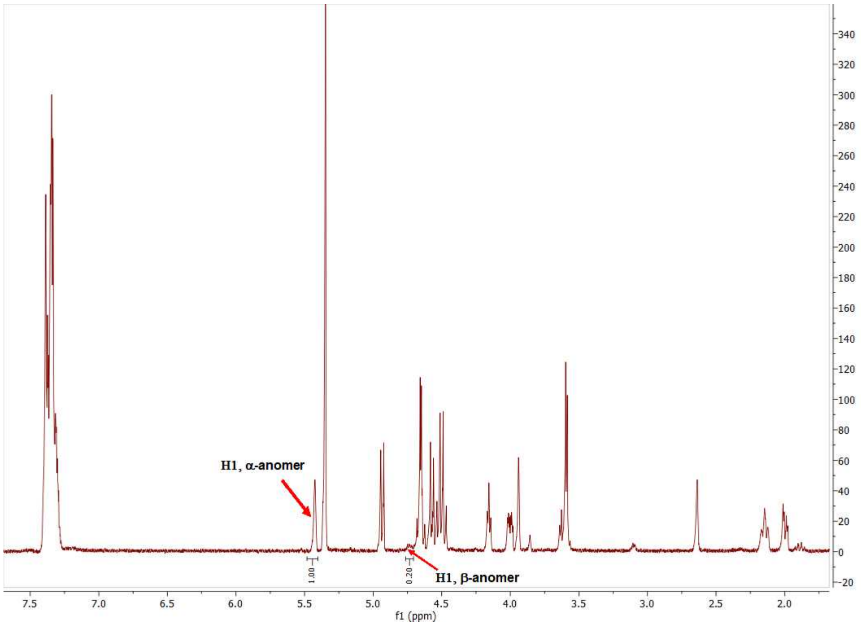

B)

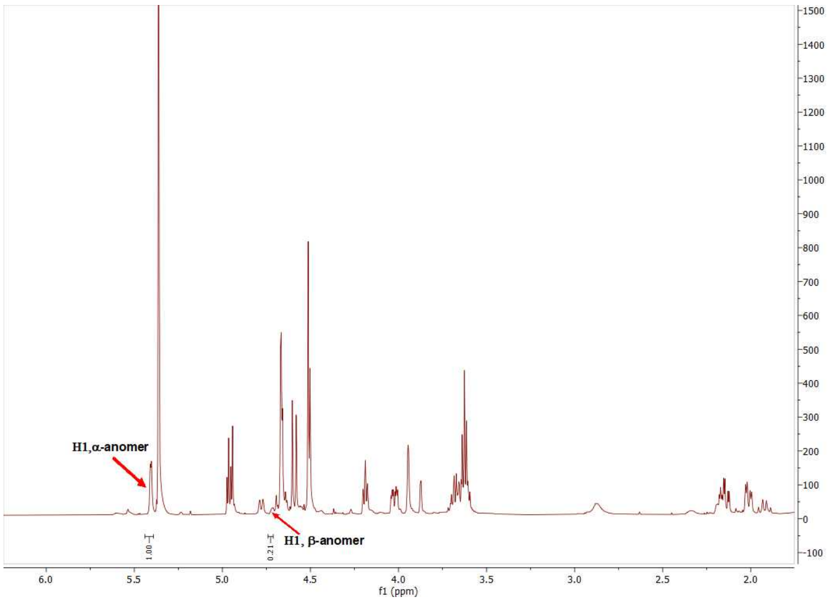

Figure S22. A) 1H-NMR spectra in CD_2Cl_2 of **2a** showing 1:0.2 alpha:beta ratio and B) 1H-NMR spectra of a 1:1 mixture of **2a** and **1e** showing the anomeric ratio remains unchanged in the presence of the catalyst **1e**

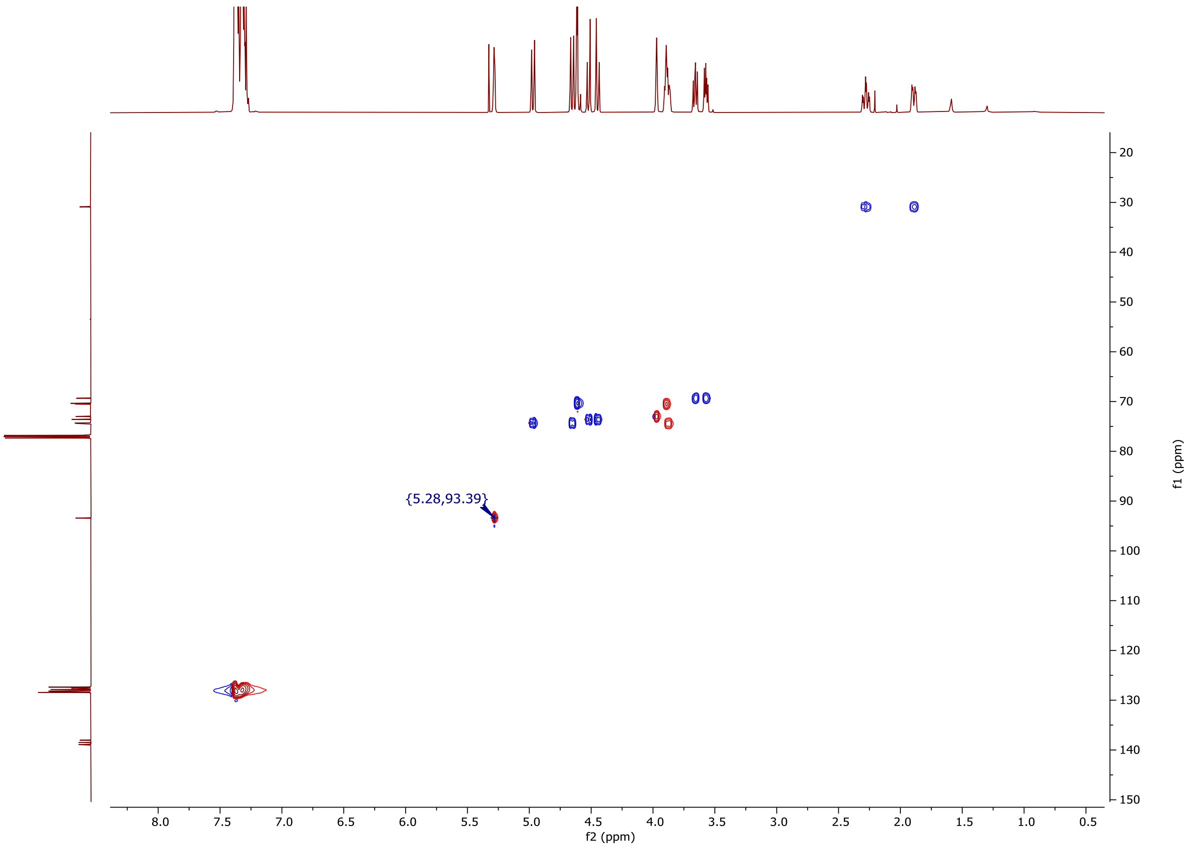

Figure S23. HSQC spectra of 2-deoxygalactoside homodimer **9b** product in $CDCl_3$.

^{1}H and HSQC NMR spectra were taken at different time points during the reaction between galactoside **2a** and glucoside **2e** to form mixed trehaloside **9c** from 0 to 18 h. The new anomeric signals as well as the α:β ratio of the hemiacetal starting materials were monitored over time. The alpha-alpha product can be seen to be the major compound formed, with no observable beta anomers visible in the HSQC spectra. The relative ratio of the alpha:beta signals for glucose hemiacetal **2e** and **2a** remain consistent throughout the experiment.

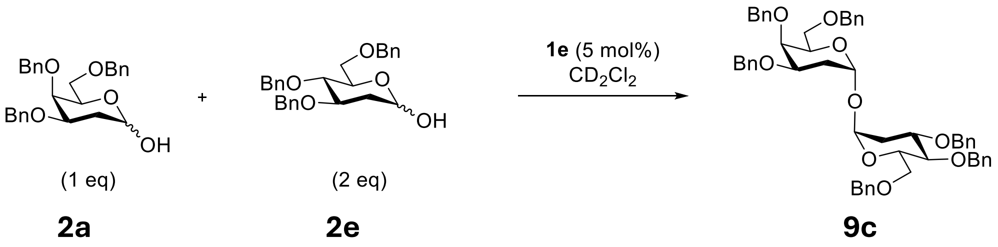

Time = 0 - starting HSQC spectra with **2a** and **2e** (no catalyst)

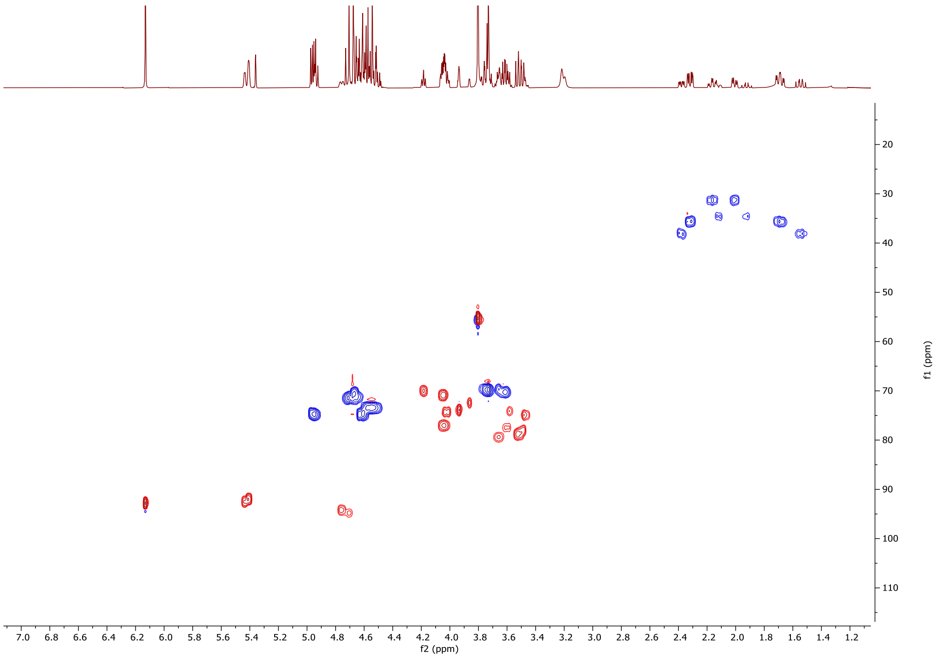

Figure S24A. HSQC spectra of galactoside **2a** and glucoside **2e** at time 0h with no catalyst

Time = 4h

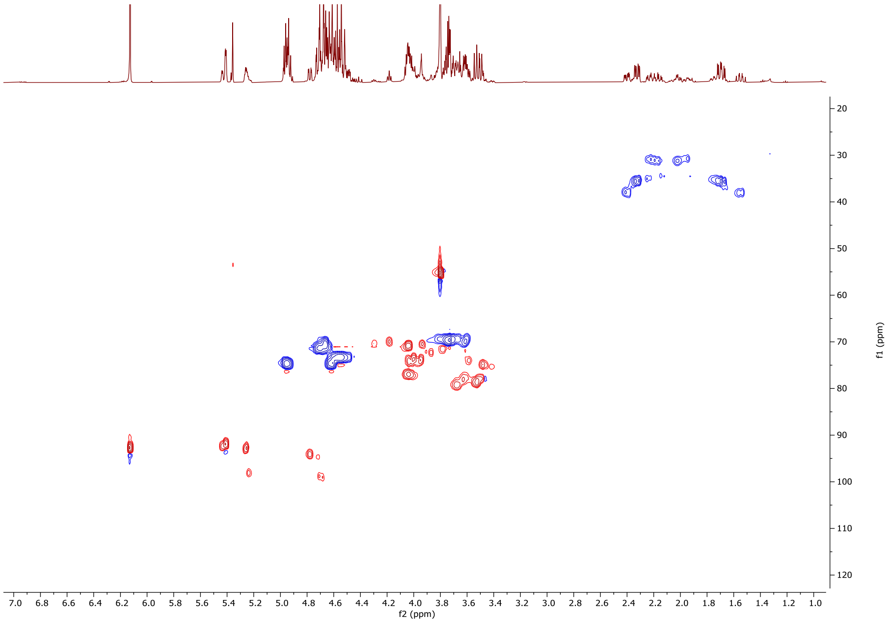

Zoomed in anomeric region of 4h spectra

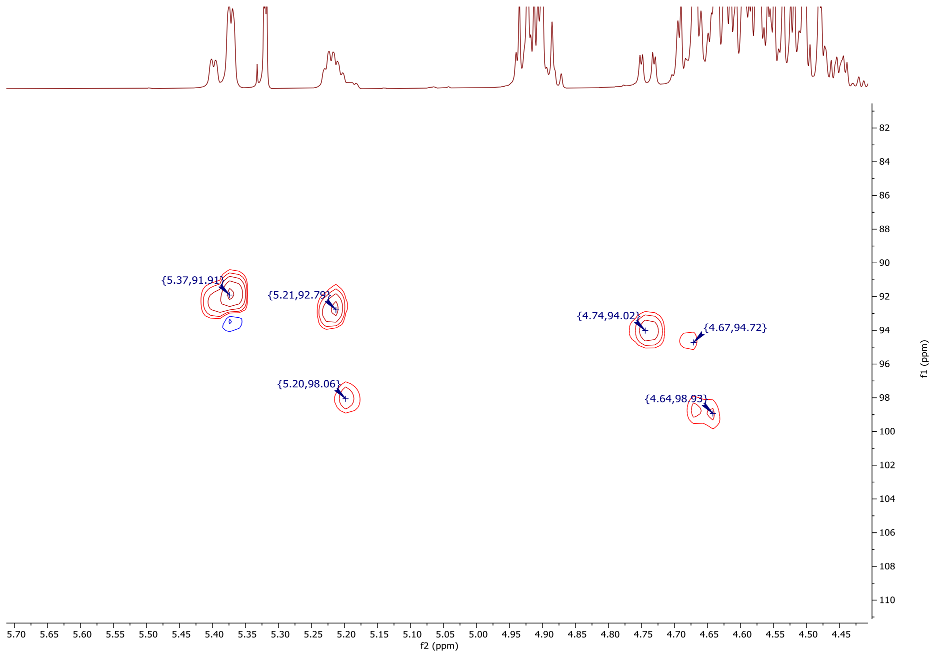

Figure S24B. HSQC spectra of galactoside **2a** and glucoside **2e** in the presence of **1e** to form mixed trehaloside **9c** at time 4h and zoomed-in spectra.

Time = 18h

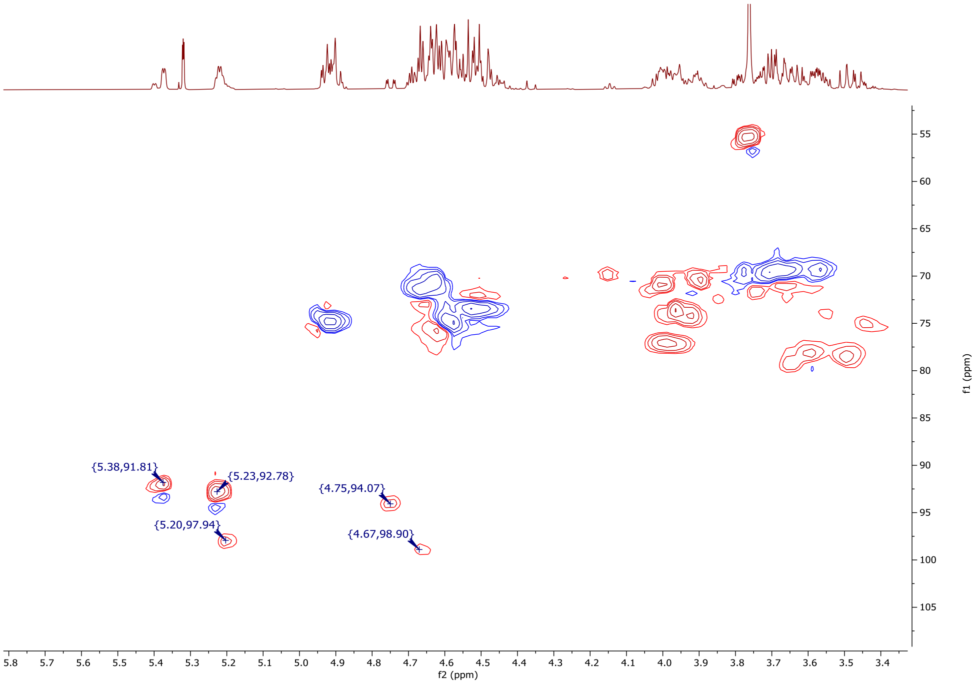

Figure S24C. HSQC spectra in CD_2Cl_2 of galactoside **2a** and glucoside **2e** in the presence of **1e** to form mixed trehaloside **9c** at time 18h.

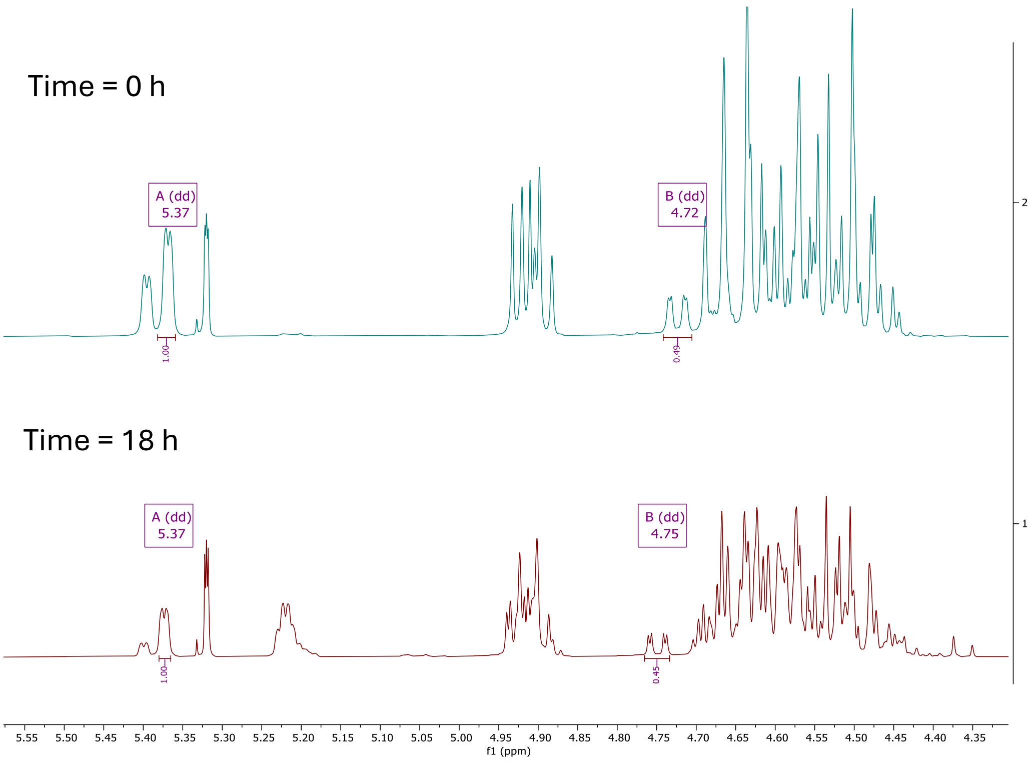

Figure S25A. 1H-NMR spectra of reaction mixture at 0 h after 18 h, anomeric H for **2e** highlighted. Anomeric signals for: **2a** H1α δ5.40 ppm, H1β δ 4.70 ppm; **2e** H1α 5.38 ppm, H1β δ 4.77 ppm; trehaloside **9c** H1 and H1' δ5.23 ppm.

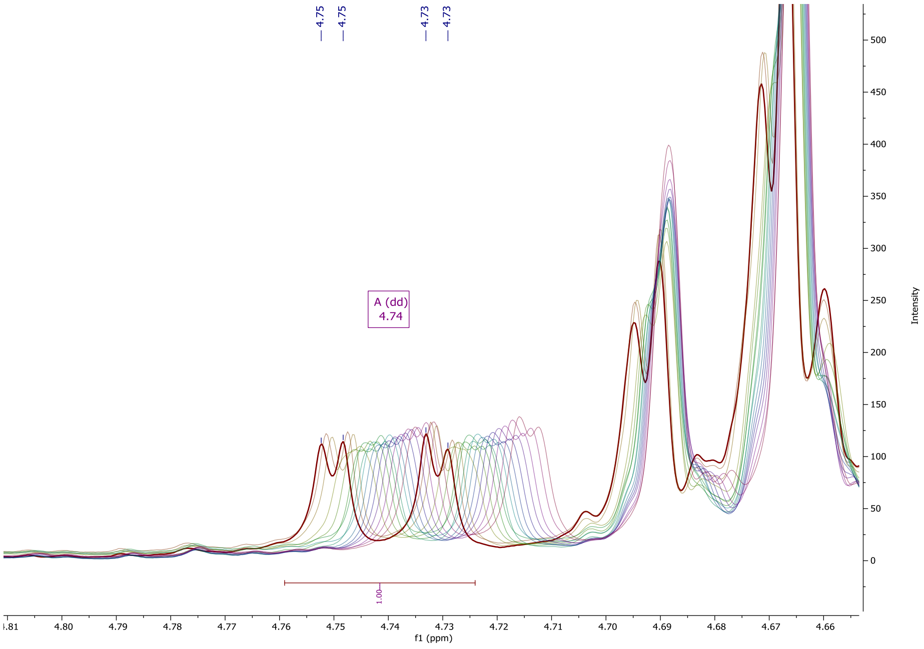

Figure S25B. Superimposed 1H NMR spectra of the reaction over time, showing the shift in beta-anomeric signal of the glucose hemiacetal **2e**. Anomeric ratios of unreacted **2a** remain unchanged throughout the reaction – see table below.

Table S10. Integration values for **2e** anomeric signals throughout the trehaloside reaction.

Data point	Time, min	**2e**		**2a** from H-NMR spectra	
		Alpha	Beta	Alpha	Beta
0	0	1	0.47	1	0.20
1	10	1	0.53	1	-
2	20	1	0.54	1	-
3	30	1	0.52	1	-
4	40	1	0.53	1	-
5	50	1	0.52	1	-
6	60	1	0.51	1	-
7	70	1	0.52	1	-
8	80	1	0.52	1	-
9	90	1	0.51	1	-
10	100	1	0.55	1	-
11	110	1	0.52	1	-
12	130	1	0.53	1	-
13	160	1	0.54	1	-
14	190	1	0.55	1	-
15	220	1	0.55	1	-
16	250	1	0.55	1	-
17	1080	1	0.55	1	0.21

Computational Studies

All density functional theory (DFT) calculations were performed using the Amsterdam Density Functional (AMS2023.101) software package.[9,10] The generalized gradient approximation (GGA) exchange-correlation functional BLYP was used for all computations, which consists of the Becke exchange, and the Lee–Yang–Parr (LYP) correlation functional.[11] In addition, dispersion effects have been included using the D3(BJ) approximation by Grimme *et al.*[12] Scalar relativistic effects are accounted for using the zeroth-order regular approximation (ZORA).[13] The basis set used, denoted TZ2P, is of triple-ζ quality for all atoms and has been improved by four sets of polarization functions.[14] The polarization functions are 2p and 3d on H, 3d and 4f on C, P, and Cl. No frozen core approximation has been employed. For all calculations, the accuracies of the fit scheme (Zlm fit) and the integration grid (Becke grid) were set to VERYGOOD.[15] No symmetry constraints were used for all computations. To account for bulk solvation of dichloromethane, we used the conductor-like screening model (COSMO).[16] For selected cases, energies were refined by performing

single-point calculations at the COSMO(DCM)-ZORA-M06-2X[17]/TZ2P level on geometries previously optimized at the COSMO(DCM)-ZORA-OLYP/TZ2P level, which verified the computed trends using OLYP. Conformer searches were performed using the algorithm of the RDKit. 5000 conformers of a given system were randomly generated. The generated conformers were compared to each other via the root mean square (RMS), and structures that were too similar were removed. The unique conformers were optimized by a universal force field (UFF). Again, the obtained conformers were compared by RMS, and duplicates were removed. The final conformers were sorted according to their energy. All these steps were automatically executed by the RDKit algorithm.[18] It would be too computationally expensive to optimize all generated conformers at a DFT level of accuracy. Therefore, several conformers were manually selected for re-optimization with DFT. All calculated stationary points have been verified by performing a vibrational analysis calculation,[19] to be energy minima (no imaginary frequencies) or transition states (only one imaginary frequency). The character of the normal mode associated with the imaginary frequency of the transition state has been inspected to ensure that it is associated with the reaction of interest. The optimized structures were illustrated using CYLview.[20] For the thermochemistry calculations, we used a standard approach whereby the geometries were optimized, and the vibrational frequencies were obtained through numerical differentiation of the analytical gradient. Enthalpies at 298.15 K and 1 atm (ΔH) were calculated from the electronic bond energies and vibrational frequencies by using a standard thermochemistry relation for an ideal gas [Eq. S1].

$$\Delta H = \Delta E_{\mathrm{trans}} + \Delta E_{\mathrm{rot}} + \Delta E_{\mathrm{vib,0}} + \Delta(\Delta E_{\mathrm{vib,298}}) + \Delta(p\mathrm{V}) \qquad \text{(S1)}$$

$\Delta E_{\mathrm{trans,298}}$, $\Delta E_{\mathrm{rot,298}}$, and $\Delta E_{\mathrm{vib,0}}$ are the differences between the reactants in the translational, rotational, and zero-point vibrational energy, respectively, whereas $\Delta E_{\mathrm{vib,298}}$ takes the vibrational energy change upon going from 0 to 298.15 K into account. Entropies were corrected according to the approach of Martin-Hay-Pratt.[21] This is important because the ideal gas approximation ignores the solvent suppression effect on the rotational and translational degrees of freedom of the solute, which can lead to a large overestimation of the entropy contributions to the Gibbs energy in solution. Finally, the change of the Gibbs energy (ΔG) in solution was then calculated according to Eq. S2. Kinetic isotope effects were computed using the Bigeleisen–Mayer approach through the reduced partition functions.

$$\Delta G = \Delta H - T\Delta S \qquad \text{(S2)}$$

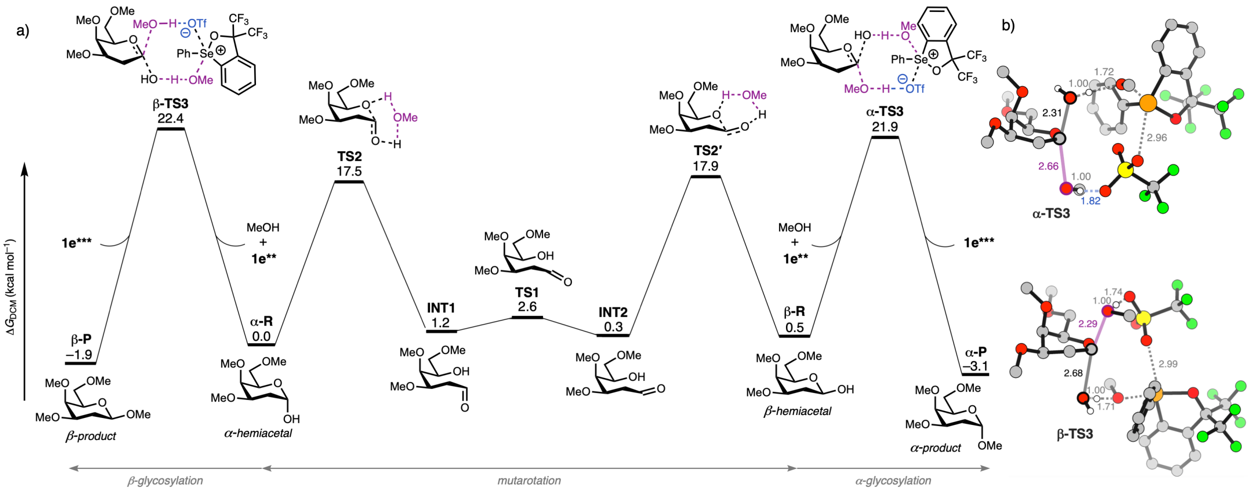

Figure S26. a) Reaction profile of organocatalytic glycosylation reactions (ΔG_{DCM} in kcal mol^{-1}) between 2-deoxy-galactosyl hemiacetal donor (**α/β-R**), two methanol acceptor molecules, and organocatalyst **1e** in dichloromethane (*i.e.*, an alternative computed pathway, which is higher in energy compared to Figure 2 of the main-text using one methanol molecule and not in agreement with our kinetic data). b) Key transition state structures for the organocatalytic glycosylation reactions (**α/β-TS3**) with key bond lengths (in Å). All non-polar hydrogens are omitted for clarity reasons. Atom colours: carbon (grey), fluorine (green), hydrogen (white), oxygen (red), selenium (orange), and sulfur (yellow). Computed at COSMO(DCM)-ZORA-BLYP-BJ(D3)/TZ2P.

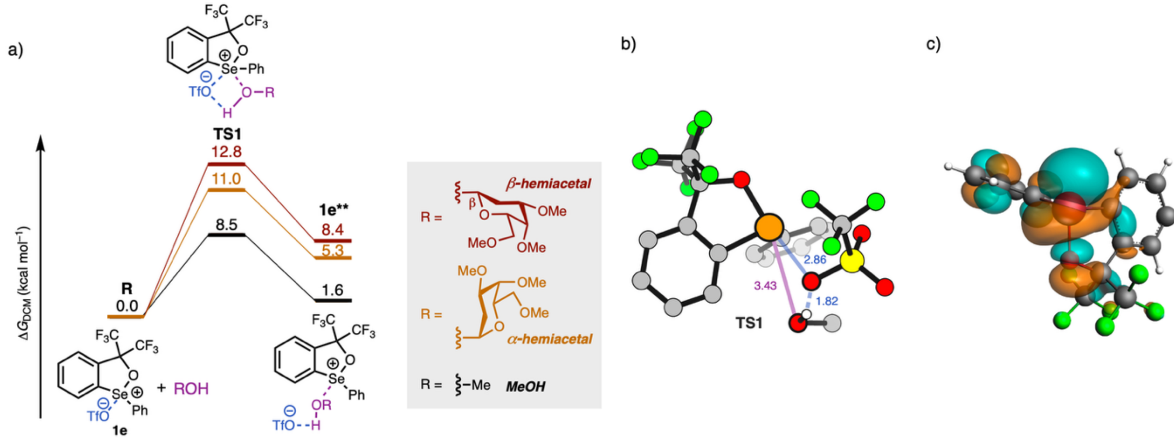

Figure S27. a) Interaction of catalyst **1e** and a series of nucleophiles following an S_N2-f transition state with key bond lengths (in Å). All non-polar hydrogens are omitted for clarity reasons. Atom colours: carbon (grey), fluorine (green), hydrogen (white), oxygen (red), selenium (orange), and sulfur (yellow). c) Catalyst LUMO (isovalue = 0.03 Bohr$^{-3/2}$). The shape of the LUMO orbital enforces high

directionality in chalcogenonium salts, favoring an interaction angle of approximately 180°. Computed at COSMO(DCM)-ZORA-BLYP-BJ(D3)/TZ2P.

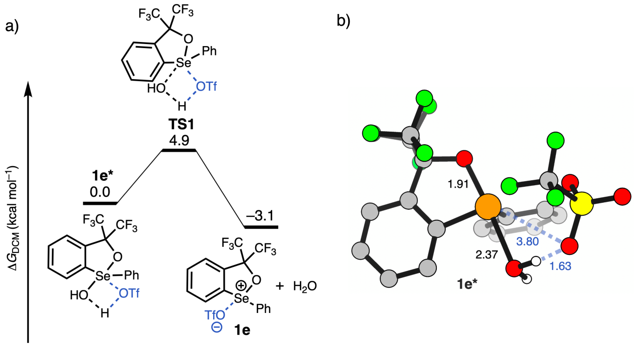

Figure S28. a) Regeneration of catalyst **1e** from **1e*** following an S_N2-f transition state with key bond lengths (in Å). All non-polar hydrogens are omitted for clarity reasons. Atom colours: carbon (grey), fluorine (green), hydrogen (white), oxygen (red), selenium (orange), and sulfur (yellow). Computed at COSMO(DCM)-ZORA-BLYP-BJ(D3)/TZ2P.

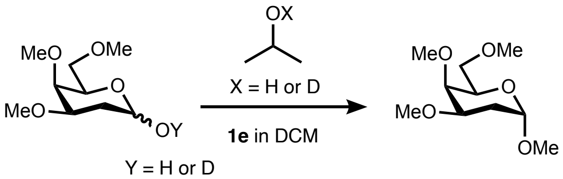

DFT-computed KIE

X = H or D:	1.304
Y = H or D:	1.032
X and Y = (50% H/D):	1.168
X and Y = H or D:	1.392

Figure S29. Calculated KIEs for the rate-determining step of the glycosylation reactions based on **α-TS3**. Four different scenarios are considered, as the deuterium on the alcohol can freely exchange with the hemiacetal. For the systems with the deuterated alcohol, proton transfer leads to a pronounced KIE. In contrast, deuteration of only the hemiacetal shows little to no effect, since that proton is not involved in the rate-determining step.

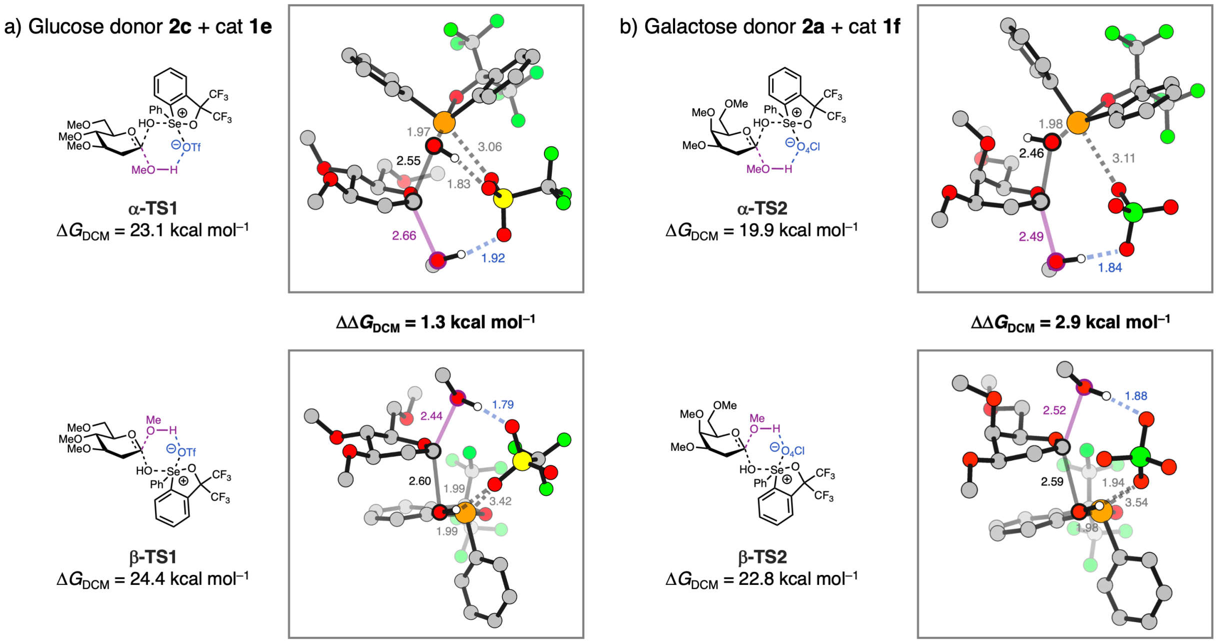

Figure S30. Computed key transition state of the glycosylation reactions of (a) methylated glucose donor **2c** and cat **1e** and (b) methylated galactose donor **2a** and cat **1f** with key bond lengths (in Å). All non-polar hydrogens are omitted for clarity reasons. Atom colours: carbon (grey), chloride (dark green), fluorine (green), hydrogen (white), oxygen (red), selenium (orange), and sulfur (yellow). Computed at COSMO(DCM)-ZORA-BLYP-BJ(D3)/TZ2P.

Table S11. Cartesian coordinates (in Å), energies (in kcal mol⁻¹), and the number of imaginary frequencies of all stationary points and transition states, computed at COSMO(DCM)-ZORA-BLYP-BJ(D3)/TZ2P.

MeOH
E = −676.03
H = −642.30
G = −659.36
***N*imag** = 0

O	-5.73786032	6.70854593	-2.63615935
C	-6.03026419	5.43780328	-1.99702643
H	-6.48117063	5.58272035	-1.00639002
H	-6.69819290	4.82357157	-2.61526830
H	-5.07421239	4.92012953	-1.88172064
H	-6.57967250	7.18427004	-2.74799837

α-R 2-deoxy-3,4,6-tri-*O*-methyl-α-D-galactopyranoside
E = −3986.93
H = −3808.84
G = −3847.71
***N*imag** = 0

C	3.05586640	15.84381957	-5.24231062
C	2.70950354	18.32341672	-5.61332798
C	3.73749848	17.18263147	-5.54470171
O	1.96412184	15.57576432	-6.13213923
C	0.96803085	16.63643530	-6.20557067
C	1.60739398	17.97289656	-6.62884436
O	2.62627084	15.86531868	-3.86851733
H	2.22247861	18.44322601	-4.63242602
O	3.30294143	19.58250364	-5.98972137
H	4.23968933	17.10255763	-6.51437361
H	4.49362561	17.37318554	-4.77754103
H	3.73931784	15.00328174	-5.40770828
O	-0.90258079	15.15647387	-6.54557207
C	-0.11072134	16.17979878	-7.17382677
H	0.51739465	16.77247520	-5.21262540
O	2.10672575	17.83040429	-7.97290512
H	3.42875956	20.33073573	-4.03101992
C	-1.90590670	14.63861611	-7.43703926
H	-2.46017656	13.87525038	-6.88489809
H	-1.44528168	14.18572882	-8.32833474
H	-2.59725580	15.43338433	-7.75807235
H	0.34839603	15.79577830	-8.09591381
H	-0.74918614	17.03894898	-7.43756786
C	4.05401690	20.20063976	-4.92730568
H	4.94479178	19.61535812	-4.66082058

H	4.36838282	21.17996989	-5.29722159
H	0.83274143	18.75357518	-6.60384011
H	2.25040281	14.98776763	-3.66855395
C	2.03713163	19.03366178	-8.76498606
H	2.33888278	18.75166254	-9.77763635
H	1.00822789	19.42313711	-8.78921087
H	2.70884251	19.80756274	-8.37742130

β-R 2-deoxy-3,4,6-tri-*O*-methyl-β-D-galactopyranoside

E = −3986.15

H = −3808.18

G = −3847.18

***N*imag** = 0

C	2.99527351	15.81098382	-5.23491407
C	2.71952225	18.28110113	-5.63613753
C	3.72312557	17.11652171	-5.57393522
O	1.94266704	15.55562175	-6.19372817
C	0.95437266	16.61556387	-6.23662232
C	1.59553145	17.96060465	-6.63825087
H	2.55365430	15.85262806	-4.22741214
H	2.24546398	18.40174406	-4.64624416
O	3.32969838	19.53132523	-6.00673186
H	4.21061630	17.00960179	-6.55136702
H	4.48898955	17.29973647	-4.81379771
O	3.84749308	14.68267815	-5.23289550
O	-0.92596864	15.15179936	-6.58568710
C	-0.14127702	16.18795877	-7.20033648
H	0.51293408	16.73613959	-5.23298990
O	2.07160359	17.85022521	-7.99240107
H	3.51691955	20.24363733	-4.03935135
C	-1.93311954	14.64534269	-7.47964376
H	-2.47997859	13.86990988	-6.93714943
H	-1.47607991	14.21035436	-8.38154521
H	-2.63008487	15.44250767	-7.78183148
H	0.30564679	15.82554233	-8.13695098
H	-0.78044940	17.05484234	-7.43468374
C	4.11679349	20.12150264	-4.95386665
H	5.00683475	19.52086400	-4.72200839
H	4.43317513	21.10301009	-5.31581615
H	0.82714979	18.74496375	-6.57750365
H	2.62566043	19.84779181	-8.36338414
C	1.96655865	19.06744461	-8.75970505
H	2.26380785	18.81246672	-9.78071266
H	0.92914691	19.43404568	-8.76556620
H	4.37087092	14.69564270	-6.05735302

1e

E = −5442.29

H = −5280.23

G = −5336.32

Nimag = 0

C	-9.19487402	-8.23816086	7.82797441
C	-9.93549396	-9.42085313	7.72659956
C	-9.92482186	-10.33434297	8.78503957
C	-9.18759031	-10.07587494	9.94456238
C	-8.44231960	-8.89765904	10.06409236
C	-8.45966451	-8.00600195	8.99553192
H	-10.51511552	-9.63021142	6.83509472
H	-10.49963112	-11.25203235	8.70059454
H	-9.18689494	-10.78982391	10.76315965
H	-7.86504148	-8.68540049	10.95452245
Se	-7.49897804	-6.31136852	8.92139810
C	-9.12740067	-7.14556295	6.76696695
C	-8.55056081	-7.69718865	5.41033866
C	-10.54221659	-6.47704972	6.57533778
O	-8.26147378	-6.09958015	7.17783918
C	-5.74801591	-6.91122125	8.30122031
C	-5.42648018	-8.26241284	8.17406620
C	-4.15162180	-8.59631021	7.71035411
C	-3.23485861	-7.59017708	7.37968041
C	-3.58169187	-6.24129647	7.51568449
C	-4.84867804	-5.88822695	7.98897984
H	-6.13924361	-9.03892065	8.42816918
H	-3.87781412	-9.64285299	7.60755078
H	-2.24581516	-7.85959948	7.01856491
H	-2.86787698	-5.46185533	7.26377838
H	-5.11877528	-4.84489721	8.11940066
F	-7.35438964	-8.30426873	5.64170469
F	-8.33945054	-6.70035886	4.51283531
F	-9.37626579	-8.61319782	4.82936204
F	-11.47681922	-7.35450870	6.11576751
F	-10.49292439	-5.43139625	5.71042786
F	-10.97402241	-6.00109436	7.77689662
F	-4.49375525	-7.68497873	12.85353264
F	-3.42653591	-5.75908259	12.72450234
F	-3.75474874	-6.96247058	10.90859624
S	-5.94693961	-5.67132405	11.81247462
O	-6.41636191	-5.36849590	13.16628232
O	-5.61671053	-4.54440384	10.93318753
O	-6.77479773	-6.73840816	11.13239935
C	-4.30134437	-6.57959338	12.08830730

1e without TfO$^-$

E = −4329.18

H = −4188.26

G = −4234.21

***N*imag** = 0

C	-1.21663652	-0.84329238	0.20703707
C	-2.08591814	-1.91855534	-0.00321018
C	-1.96822717	-3.05872912	0.79879378
C	-1.00048149	-3.13780329	1.80687188
C	-0.12482616	-2.07013266	2.03359146
C	-0.25910278	-0.95221555	1.21844898
H	-2.84069539	-1.87406894	-0.77984883
H	-2.64185392	-3.89377900	0.63165870
H	-0.92260920	-4.03060724	2.42000003
H	0.63186682	-2.11875791	2.80939387
Se	0.82626778	0.64339281	1.33911509
C	-1.20978261	0.47232683	-0.55771729
C	-0.99758107	0.25326176	-2.10104950
C	-2.51106142	1.30362283	-0.24058043
O	-0.11048168	1.29938820	-0.13618439
C	2.46215615	0.03268865	0.54088491
C	2.53571890	-1.12953997	-0.24072887
C	3.77174370	-1.48126837	-0.77753408
C	4.90083182	-0.68361083	-0.53286890
C	4.80788308	0.47173717	0.25226476
C	3.57927930	0.84406281	0.79960360
H	1.65975938	-1.74180028	-0.42211890
H	3.85732239	-2.37748900	-1.38525993
H	5.85965452	-0.96929825	-0.95677406
H	5.68709881	1.08129570	0.43913871
H	3.49152912	1.73753472	1.41132969
F	0.10990833	-0.51351710	-2.29659102
F	-0.80995112	1.42655904	-2.75098997
F	-2.05667395	-0.38282317	-2.66683074
F	-3.63524369	0.64788655	-0.62770263
F	-2.49632909	2.51586940	-0.84491848
F	-2.58206314	1.50659114	1.10500950

TfO$^-$

E = −1091.71

H = −1070.78

G = −1096.64

***N*imag** = 0

O	-8.21262854	-2.17371197	1.82034706
C	-9.12762433	0.08780942	2.86773289
F	-9.23891689	0.68753889	1.64732711

F	-8.86878383	1.06082758	3.78845278
F	-10.33772161	-0.46352988	3.17304351
S	-7.75799008	-1.22848954	2.86051513
O	-6.55130853	-0.44939495	2.51568324
O	-7.79347235	-1.75130001	4.24162596

TS1-S_N2-f 1e-MeOH
E = −6117.88
H = −5921.20
G = −5983.74
Nimag = 1, −55.9927

C	-9.30042769	-8.36878512	7.85790616
C	-9.94101865	-9.60318571	7.71129690
C	-9.77113409	-10.58436780	8.69339627
C	-8.97044157	-10.34743964	9.81557339
C	-8.32367952	-9.11761908	9.98051685
C	-8.50617242	-8.15874831	8.99006192
H	-10.56146681	-9.80160353	6.84503412
H	-10.26858842	-11.54257924	8.57612217
H	-8.84242807	-11.11984985	10.56822469
H	-7.68069897	-8.92853535	10.83105303
Se	-7.76085069	-6.36623635	9.01283614
C	-9.39009300	-7.19817767	6.88908854
C	-8.88081087	-7.59395548	5.45423757
C	-10.85103125	-6.60815554	6.86490799
O	-8.54561553	-6.12421070	7.31314033
C	-5.93986712	-6.69069263	8.45975598
C	-5.50474810	-7.93697272	7.99543362
C	-4.17767656	-8.05810654	7.58785183
C	-3.31734351	-6.95035816	7.64284459
C	-3.77374300	-5.71390077	8.11142309
C	-5.09789409	-5.57272403	8.53411943
H	-6.17421549	-8.78886252	7.96023099
H	-3.81287689	-9.01626696	7.22863317
H	-2.28414430	-7.05752313	7.32371800
H	-3.10184230	-4.86182409	8.15958255
H	-5.46196737	-4.62826518	8.92589547
F	-7.64091717	-8.14628531	5.55409828
F	-8.78828840	-6.51438888	4.63920506
F	-9.69935923	-8.50326472	4.85821891
F	-11.76490643	-7.51918346	6.43709702
F	-10.94154181	-5.51478372	6.06752384
F	-11.19155755	-6.23187758	8.12933832
F	-9.83411299	-4.10255365	10.52233563
F	-9.27769137	-2.42797589	11.84511873
F	-10.23772440	-4.22469035	12.68624196

S	-7.64567371	-4.56030719	12.02182206
O	-6.82509121	-3.99239467	10.93732283
O	-7.27404545	-4.18571755	13.39096452
O	-7.94443130	-6.01639258	11.84055823
C	-9.35378585	-3.78013452	11.75695266
O	-5.85057964	-7.77504746	11.49354317
C	-4.57326174	-7.10536214	11.59613236
H	-4.64553983	-6.06035314	11.27136978
H	-4.19246965	-7.13497183	12.62596851
H	-3.87589463	-7.63217208	10.93921892
H	-6.53418094	-7.16826127	11.85421295

1e-MeOH**
E = −6125.91
H = −5928.61
G = −5990.58
***N*imag** = 0

C	-9.03131651	-8.27528800	7.88598156
C	-9.48328067	-9.58852477	8.05494887
C	-9.32400624	-10.21985263	9.29177231
C	-8.72309044	-9.55037426	10.36145444
C	-8.27258604	-8.23426060	10.21270583
C	-8.43551875	-7.62497001	8.97236301
H	-9.95067459	-10.11715991	7.23242308
H	-9.67074845	-11.24149313	9.41636402
H	-8.59738491	-10.04821193	11.31851139
H	-7.79579642	-7.73205341	11.04309391
Se	-7.88058017	-5.81053414	8.51617909
C	-9.14150058	-7.46518445	6.59811616
C	-8.43997475	-8.19698009	5.39385768
C	-10.65339885	-7.14226204	6.28722562
O	-8.48928024	-6.21682021	6.73585400
C	-5.98691365	-6.08023069	8.14601753
C	-5.40881226	-7.34916241	8.13617184
C	-4.05056856	-7.45384753	7.83301373
C	-3.30077680	-6.30690438	7.54766455
C	-3.90187930	-5.04364681	7.56132956
C	-5.26068732	-4.91882615	7.86303836
H	-5.98973669	-8.23451778	8.36425209
H	-3.57893934	-8.43247468	7.82862155
H	-2.24257254	-6.39816081	7.31767954
H	-3.31824444	-4.15429402	7.34033475
H	-5.73483966	-3.94175621	7.87014457
F	-7.16619268	-8.52594371	5.74336247
F	-8.37256417	-7.40075193	4.29598237
F	-9.08295567	-9.34466385	5.03582617

F	-11.40043607	-8.26397453	6.09380993
F	-10.78764189	-6.35768313	5.18689321
F	-11.18630119	-6.46599031	7.34507000
F	-4.55315480	-9.47350751	12.85030936
F	-2.90040282	-8.89036573	11.51180371
F	-4.94698963	-9.09223225	10.71847643
S	-4.53379124	-6.85800287	12.18851033
O	-3.80457486	-6.64761167	13.44736787
O	-4.04832588	-6.13046428	11.01028590
O	-6.02355442	-6.83232207	12.34482080
C	-4.21516499	-8.68751142	11.79227363
O	-7.41648117	-5.30580992	10.73661732
C	-6.83359514	-3.97055320	10.84323535
H	-6.93458236	-3.63034256	11.87762595
H	-5.77922657	-3.99109916	10.55349676
H	-7.40116327	-3.30997344	10.18431385
H	-6.83539548	-5.92572684	11.28629790

TS1-S$_N$2-f 1e-2-deoxy-3,4,6-tri-*O*-methyl-α-D-galactopyranoside

E = −9430.31

H = −9089.75

G = −9169.57

***N*imag** = 1, −14.5709

C	-8.83356441	-8.62549907	8.09036080
C	-9.19153528	-9.97739164	8.10156675
C	-8.73807179	-10.79663178	9.14077444
C	-7.94194018	-10.28013524	10.16892736
C	-7.57994064	-8.92835266	10.17837491
C	-8.03309020	-8.13879954	9.12796925
H	-9.80924324	-10.39049833	7.31237109
H	-9.01162728	-11.84753869	9.14426291
H	-7.59903156	-10.92871019	10.97006403
H	-6.95361987	-8.50596173	10.95633543
Se	-7.68642316	-6.23400956	8.92227379
C	-9.24908781	-7.59409582	7.05105299
C	-8.89855367	-8.05103335	5.58769929
C	-10.77676738	-7.23875979	7.21286661
O	-8.52590994	-6.37212091	7.24317278
C	-5.84646301	-6.26520034	8.32714996
C	-5.31533908	-7.37548710	7.66104374
C	-3.99680190	-7.30505762	7.21382372
C	-3.23148498	-6.15406118	7.45375233
C	-3.78094091	-5.06057692	8.13228801
C	-5.10758314	-5.10042244	8.56945837
H	-5.90730186	-8.26804297	7.49433713

H	-3.56299098	-8.15050006	6.68758484
H	-2.20105939	-6.11393214	7.11168775
H	-3.18311117	-4.17288305	8.31751535
H	-5.55476803	-4.25726944	9.08698215
F	-7.59887599	-8.45126226	5.53439679
F	-9.05750174	-7.04260791	4.69729936
F	-9.67610713	-9.09411139	5.18903184
F	-11.57342478	-8.32949629	7.06860069
F	-11.17713935	-6.30021559	6.32033566
F	-10.97436094	-6.73557335	8.46495997
F	-10.21485702	-4.38668798	10.42607986
F	-10.09120992	-2.33488808	11.22551645
F	-10.19507719	-4.05039165	12.60505420
S	-7.79788425	-3.73306840	11.41131276
O	-7.42853012	-3.26165121	10.06307890
O	-7.36616867	-2.89849593	12.53766774
O	-7.58249036	-5.19893363	11.60627563
C	-9.69066167	-3.61677958	11.42157964
H	-3.49974150	-10.17810369	13.51114600
H	-6.08078829	-6.19208058	11.81174242
C	-1.02143675	-10.17640553	13.24929257
H	-0.00310441	-9.84985577	13.47776670
H	-1.37775282	-10.83977394	14.05165907
H	-1.03448508	-10.71940441	12.29773253
C	-4.17143319	-6.78342364	11.72220115
C	-3.68874280	-9.24455060	11.56540121
C	-3.44004155	-7.87006228	10.92797428
O	-3.80176051	-6.80599104	13.11094551
C	-4.00463693	-8.09380449	13.75844536
C	-3.23680074	-9.22126201	13.03582784
O	-5.57232764	-6.98976205	11.54345077
H	-4.76895627	-9.45955368	11.56392257
O	-3.01502315	-10.31461357	10.87286955
H	-2.36626595	-7.65873558	10.93833325
H	-3.78971261	-7.84431418	9.89497610
H	-3.88627886	-5.77949974	11.38844780
O	-4.56571117	-7.17988525	15.91556196
C	-3.57613807	-7.94753140	15.20952437
H	-5.07526914	-8.34018813	13.72958345
O	-1.82497364	-8.98012253	13.18510059
H	-4.70959219	-10.90130843	9.77922459
C	-4.18092686	-6.94514696	17.28156953
H	-4.98211173	-6.36223379	17.74311436
H	-3.23809877	-6.37937625	17.33371141
H	-4.05741377	-7.89444638	17.82605141
H	-2.59638405	-7.45203286	15.26473876

H	-3.48263379	-8.94778888	15.66384354
C	-3.64622563	-10.66167775	9.62636620
H	-3.56580986	-9.85241128	8.88798022
H	-3.12649995	-11.54420303	9.24567174

1e-2-deoxy-3,4,6-tri-*O*-methyl-α-D-galactopyranoside**

E = −9436.43

H = −9095.29

G = −9175.34

Nimag = 0

C	-8.53552440	-8.90517617	7.80037865
C	-8.62072599	-10.27389211	7.52338679
C	-8.04782599	-11.19305359	8.40790370
C	-7.40976058	-10.76093732	9.57473360
C	-7.31611317	-9.39632324	9.86504227
C	-7.86496010	-8.49666568	8.95812707
H	-9.13063944	-10.62327947	6.63315204
H	-8.11346653	-12.25435004	8.18749122
H	-6.98661822	-11.48478415	10.26477764
H	-6.84578702	-9.04309154	10.77239433
Se	-7.85608514	-6.56000390	9.15362589
C	-9.16182194	-7.79014194	6.97023044
C	-8.62321446	-7.79012470	5.49207335
C	-10.73530605	-7.89385211	7.02366253
O	-8.84127085	-6.51794941	7.51418624
C	-6.13047393	-6.11482870	8.35342987
C	-5.28874309	-7.09575286	7.82610227
C	-4.06636404	-6.69535669	7.28095032
C	-3.70970264	-5.34084285	7.26809961
C	-4.57212565	-4.37524355	7.79869214
C	-5.79770615	-4.75602801	8.35458214
H	-5.56869349	-8.14315672	7.83356059
H	-3.39487065	-7.44351389	6.86888995
H	-2.75582997	-5.03899280	6.84406724
H	-4.29383615	-3.32512188	7.78877057
H	-6.47138367	-4.01995220	8.78159837
F	-7.26186977	-7.76555376	5.50605195
F	-9.04756308	-6.70121349	4.80327329
F	-9.01152120	-8.89963777	4.80207499
F	-11.19990153	-9.05609905	6.48739371
F	-11.33053675	-6.86520048	6.36924212
F	-11.13559222	-7.84694138	8.32517072
F	-7.30468991	-1.24940142	11.39045354
F	-6.01122391	-3.02933254	11.50140262
F	-7.13488559	-2.36996204	13.28059933

S	-8.64870553	-3.57868442	11.39719087
O	-8.46500153	-3.68560073	9.93440842
O	-8.42016414	-4.85206108	12.13442563
O	-9.83037577	-2.83142030	11.84623526
C	-7.18640151	-2.49401856	11.93311276
H	-4.44990357	-8.44685424	14.65208682
H	-7.29506339	-5.97713340	11.68056595
C	-1.98512196	-8.08521816	14.51364368
H	-1.06445479	-7.50230590	14.60245386
H	-2.32873154	-8.37652070	15.51713608
H	-1.80146624	-8.98508565	13.91663117
C	-5.30680108	-6.31011260	11.49825664
C	-4.54970264	-8.51496473	12.49072979
C	-4.42578082	-7.54476472	11.30512368
O	-5.17772608	-5.70961725	12.75737035
C	-5.28506189	-6.60456967	13.91093629
C	-4.27973651	-7.76361126	13.80727008
O	-6.72082482	-6.70581054	11.28165461
H	-5.57786838	-8.90697783	12.55476378
O	-3.63814995	-9.62285546	12.38289585
H	-3.38686832	-7.20644138	11.23711492
H	-4.68280064	-8.02767786	10.35956157
H	-5.06095940	-5.52511660	10.77961439
O	-6.27413099	-4.99163451	15.38452613
C	-5.07538665	-5.74063784	15.14399458
H	-6.29958631	-7.02340416	13.94028997
O	-2.95052249	-7.21952809	13.87867184
H	-5.05289366	-11.03646391	11.75255753
C	-6.07315589	-3.93716865	16.33789843
H	-7.03063742	-3.42168736	16.44760177
H	-5.31353138	-3.22517731	15.98057425
H	-5.75761964	-4.33487648	17.31562375
H	-4.22405522	-5.06544245	14.97799446
H	-4.84212698	-6.38240291	16.01003751
C	-4.07427413	-10.63183341	11.45379796
H	-4.14050119	-10.24507404	10.42746267
H	-3.32878829	-11.42968903	11.48468143

TS1-S$_N$2-f 1e-2-deoxy-3,4,6-tri-*O*-methyl-β-D-galactopyranoside

E = −9427.30

H = −9086.46

G = −9167.81

***N*imag** = 1, −39.7963

C	-8.68918869	-8.34930602	7.96329365
C	-8.92133921	-9.65476112	7.51847709
C	-8.36207263	-10.72694558	8.22136138

C	-7.57597137	-10.51009752	9.35815551
C	-7.33645992	-9.21107854	9.82052544
C	-7.90261906	-8.16211559	9.10392053
H	-9.52356287	-9.83733170	6.63595299
H	-8.54095803	-11.73968164	7.87228188
H	-7.14229042	-11.35199881	9.89008039
H	-6.71233858	-9.02249713	10.68698033
Se	-7.75600987	-6.28129817	9.57055835
C	-9.22383736	-7.07677860	7.31969507
C	-8.79061822	-6.95954263	5.81219963
C	-10.78643464	-6.98135655	7.50011582
O	-8.67921957	-5.91974348	7.95993014
C	-5.96136823	-5.88948364	9.00002689
C	-5.24539527	-6.73863841	8.14752000
C	-3.96944897	-6.34279319	7.75179734
C	-3.43867537	-5.12433100	8.20190259
C	-4.17089979	-4.29594108	9.05750853
C	-5.44564326	-4.67548768	9.47562415
H	-5.66437062	-7.67852652	7.80662495
H	-3.38764639	-6.98431049	7.09573093
H	-2.43944168	-4.83015359	7.89317728
H	-3.73980879	-3.37112118	9.42454105
H	-6.01469227	-4.07014283	10.17347527
F	-7.44330914	-7.12393734	5.71505592
F	-9.09721175	-5.74383032	5.29576233
F	-9.38648090	-7.90774708	5.03814731
F	-11.43560068	-8.02101949	6.91134895
F	-11.28644403	-5.82890368	6.98814474
F	-11.07483605	-7.00565326	8.83167444
F	-10.53067599	-5.28551474	11.38700020
F	-10.59724522	-3.70024680	12.91921780
F	-10.72734021	-5.81765240	13.51767958
S	-8.24696816	-5.01431943	12.79957484
O	-7.79917587	-4.05380935	11.77750410
O	-7.98430258	-4.67452028	14.19922332
O	-7.97703701	-6.44450109	12.44500551
C	-10.13766939	-4.95176937	12.65063566
H	-1.32533276	-4.81668637	13.14080226
H	-6.36256679	-7.14811101	12.26465853
C	-0.78256858	-4.18711256	10.77941761
H	-0.93723179	-3.60583264	9.86535629
H	-0.30954127	-3.54370542	11.53714275
H	-0.12783014	-5.04026441	10.57021019
C	-4.42869961	-7.00550184	12.48008847
C	-1.95108627	-6.76913262	12.44397865
C	-3.15613436	-7.42241022	11.74489831

O	-4.56691455	-5.57462089	12.46716561
C	-3.48353531	-4.91397864	13.15637553
C	-2.12035583	-5.23671561	12.50542238
O	-5.56314849	-7.54659690	11.84014357
H	-1.90919296	-7.12686289	13.48841264
O	-0.69235188	-7.07176576	11.81144007
H	-3.21829595	-7.07736375	10.70737010
H	-3.07216826	-8.51400078	11.75778228
H	-4.39206333	-7.33475821	13.53689826
O	-4.80593325	-3.17545029	14.15706119
C	-3.80728309	-3.42655693	13.15472765
H	-3.44827003	-5.26588517	14.20213763
O	-2.08608133	-4.61331641	11.21153474
H	-0.22315185	-8.66619878	13.09620818
C	-5.64061825	-2.04841767	13.84394772
H	-6.36854910	-1.96083934	14.65437564
H	-6.17585351	-2.21706589	12.89949045
H	-5.05233040	-1.11940418	13.77690783
H	-4.18026278	-3.14684423	12.16169930
H	-2.89982233	-2.83712038	13.36311599
C	-0.25613915	-8.42734940	12.02204195
H	-0.90808392	-9.15173508	11.51470599
H	0.75080572	-8.49944372	11.60272467

1e-2-deoxy-3,4,6-tri-*O*-methyl-β-D-galactopyranoside**

E = −9435.45

H = −9094.91

G = −9172.16

***N*imag** = 0

C	-7.99470153	-8.24331130	8.00482180
C	-7.79271797	-9.52394577	7.47931115
C	-7.05019079	-10.45918396	8.20685739
C	-6.52198203	-10.13407379	9.45992711
C	-6.71597930	-8.85827134	10.00000625
C	-7.43637912	-7.93503312	9.25035563
H	-8.21092593	-9.79392380	6.51663218
H	-6.89379199	-11.45098289	7.79292625
H	-5.95855930	-10.87142768	10.02436383
H	-6.32659718	-8.59526864	10.97369556
Se	-7.82074984	-6.09987252	9.78121304
C	-8.81781375	-7.13887234	7.35273922
C	-8.24723791	-6.74534298	5.94029718
C	-10.33601647	-7.56019053	7.27905397
O	-8.78865076	-5.95780774	8.14280923
C	-6.22398360	-5.19334672	9.13225687

C	-5.14143541	-5.88714410	8.59362297
C	-4.07399609	-5.14560218	8.08413522
C	-4.11193157	-3.74731229	8.10941075
C	-5.21199065	-3.07561751	8.65468586
C	-6.28233563	-3.79864739	9.18470867
H	-5.12147805	-6.97067283	8.56400366
H	-3.21462892	-5.66432647	7.66900264
H	-3.27766202	-3.17789579	7.70853236
H	-5.23572887	-1.98978850	8.67667849
H	-7.13019496	-3.29136392	9.63345526
F	-6.92474006	-6.44491606	6.05379160
F	-8.87533290	-5.65515118	5.43167409
F	-8.36817577	-7.75782064	5.03577613
F	-10.53400754	-8.65913216	6.50002364
F	-11.11343556	-6.55937456	6.79385455
F	-10.77327356	-7.85526734	8.53494738
F	-9.71286733	-3.72810195	15.32604151
F	-9.35438574	-2.01126284	13.99005571
F	-7.73995117	-3.46107064	14.38455325
S	-9.65361220	-4.36423438	12.70926814
O	-11.11732612	-4.25105977	12.74478061
O	-8.97800269	-3.75819432	11.54943296
O	-9.14444971	-5.72554066	13.06387498
C	-9.07791963	-3.32692521	14.19164308
H	-2.15404661	-4.57693032	13.03367254
H	-7.70450260	-6.16271986	12.46418127
C	-1.39043378	-4.93818228	10.67890810
H	-1.36673407	-4.73233159	9.60533959
H	-0.81185974	-4.16504398	11.20706623
H	-0.94761672	-5.92056373	10.87856170
C	-5.70167893	-6.05088164	12.62438763
C	-3.25516942	-6.43745604	12.93845717
C	-4.53629067	-6.97514741	12.28000256
O	-5.45104587	-4.71071430	12.19785423
C	-4.26120757	-4.14731143	12.81197006
C	-3.00922536	-4.99033769	12.47844507
O	-6.89153299	-6.50828189	11.97273387
H	-3.39111406	-6.41533348	14.03383234
O	-2.09574621	-7.23379133	12.63575370
H	-4.39827024	-6.99196606	11.19405831
H	-4.76417208	-7.98405003	12.63725287
H	-5.90242832	-6.05576883	13.70916297
O	-5.05147491	-1.79768830	12.92558113
C	-4.10392783	-2.70346987	12.33315286
H	-4.39335638	-4.14285798	13.90667881
O	-2.77568443	-4.89306357	11.06461290

H	-2.14156859	-8.32111946	14.43266444
C	-6.25347098	-1.62017095	12.14309322
H	-6.88310205	-0.92020871	12.69799057
H	-6.78615888	-2.56465820	12.00676249
H	-6.01020144	-1.18742288	11.16117597
H	-4.16088712	-2.67061153	11.23970262
H	-3.11063647	-2.35535551	12.64111995
C	-2.05816575	-8.48614487	13.34801242
H	-2.85975904	-9.16492200	13.02573483
H	-1.09179457	-8.94347012	13.12175951

TS2

E = −4649.43

H = −4441.10

G = −4486.08

Nimag = 1, −1146.7652

H	5.79657470	0.41435544	7.74896447
H	5.24142811	3.53110262	12.72612641
H	3.21849389	4.90797001	5.32504689
H	5.79089824	1.92799584	6.79556917
H	9.76728123	3.37155877	10.90957431
C	9.64799122	3.54668440	9.83101087
H	6.31657249	3.86451163	7.95121372
C	6.17708166	1.43867045	7.70189844
H	6.66055791	2.21608703	11.30371806
H	7.27321087	1.42445130	7.66949769
H	4.79890287	5.81666052	12.25084974
C	5.82631091	4.07524279	11.97197909
O	6.00562773	5.35690605	12.15685331
O	8.38804260	3.05171156	9.34108801
H	7.82128898	3.38845046	11.95997892
O	5.69809930	2.10673149	8.88595181
H	2.71845316	3.38737500	6.13030318
C	6.05231076	3.50185258	8.95288717
C	6.93461073	3.27496097	11.32325200
O	4.42705762	3.90742560	10.75723679
C	4.83310246	4.31196889	9.43149938
H	7.45405578	4.82526442	9.89380897
C	7.24685413	3.74391629	9.88974850
H	3.36634247	3.08043414	8.42543559
H	2.75361216	4.67435463	8.95011634
H	1.95076270	4.93908915	6.59009774
C	2.89132015	4.46535270	6.26936646
C	3.61820141	4.14702106	8.51478726
H	9.74975826	4.62381834	9.63005248

O	3.93922218	4.69677190	7.22705292
H	5.12994065	5.37324702	9.43388081
H	10.42757911	3.00005836	9.29416162
C	3.24309670	7.03302666	11.28059397
H	3.13387762	7.85440735	11.99275679
H	3.99286961	7.29533509	10.52576825
H	2.28275397	6.83559431	10.79742795
H	3.80383124	4.92722586	11.32908737
O	3.64661358	5.83893261	12.01544432

INT1

E = −3982.96

H = −3806.6

G = −3846.53

***N*imag** = 0

H	5.83316511	0.29791170	7.97275358
H	5.60382882	3.67027722	13.11257945
H	3.54131797	5.01788441	5.17085595
H	5.89618065	1.76828497	6.95599928
H	9.72468585	3.68734867	10.81861198
C	9.53333749	3.82622505	9.74409578
H	6.21926981	3.77622330	8.03976673
C	6.21092011	1.32235265	7.91086840
H	6.49595147	2.24823443	11.40194566
H	7.30554997	1.31891456	7.96696597
H	3.52229049	4.38087212	11.02172013
C	6.22673624	4.16449004	12.33869502
O	6.33549910	5.38565769	12.32292627
O	8.30586463	3.20139357	9.33069711
H	7.96580057	3.10880470	11.85986275
O	5.63162167	2.03655516	9.02262071
H	2.99717678	3.43898776	5.82022899
C	5.93820642	3.44374473	9.04801381
C	6.97055922	3.23316299	11.40584938
O	4.14458828	3.71811844	10.67301451
C	4.66131034	4.21999892	9.41231005
H	7.24548846	4.86055076	10.00039110
C	7.13453610	3.76627959	9.96771780
H	3.37393349	3.00874558	8.15972384
H	2.64762698	4.55248772	8.69275510
H	2.12852116	4.93992135	6.26921174
C	3.11602906	4.51071111	6.04065647
C	3.57627040	4.07551793	8.33826711
H	9.51775335	4.90388273	9.52238369
O	4.03124750	4.71032689	7.13266046
H	4.92753935	5.28136316	9.51234777

H	10.33532243	3.34645457	9.17724510

TS1

E = −3980.77

H = −3804.39

G = −3845.11

Nimag = 1, −60.2579

H	5.05631015	0.33958996	8.94861141
H	5.27094477	3.06983268	11.91252727
H	3.62422235	4.06221069	4.92943579
H	5.42893363	1.41158859	7.56133324
H	9.85636361	4.09788850	10.55059333
C	9.41214541	4.60037118	9.68042484
H	6.28025056	3.49084263	8.09445917
C	5.63808812	1.20486348	8.62052293
H	7.41638353	1.78448516	10.71789956
H	6.70950140	0.98626113	8.73474212
H	3.97265074	5.72547980	10.57384151
C	6.28306762	2.74717325	12.21540391
O	6.49385656	2.36109431	13.36105609
O	8.29116947	3.86828504	9.14674812
H	8.31891824	2.97177346	11.65599295
O	5.22893765	2.31319787	9.44443612
H	2.72841624	3.10406189	6.15010020
C	5.90502125	3.54446378	9.12345278
C	7.36226978	2.79167902	11.15410862
O	4.40506610	4.85542820	10.50962218
C	4.89541260	4.69953500	9.15406674
H	7.03331853	4.82050804	10.48985145
C	7.14365969	3.82618722	10.03320523
H	3.20388833	3.53586674	8.47049072
H	2.99364148	5.30495673	8.31634187
H	2.36406870	4.84447563	5.93368101
C	3.16403474	4.08835691	5.92051425
C	3.71168084	4.47504729	8.20718969
H	9.10887019	5.61766958	9.96925419
O	4.20342607	4.41919015	6.85829202
H	5.43753291	5.60536996	8.84442401
H	10.15633235	4.65361390	8.88166045

INT2

E = −3982.63

H = −3805.8

G = −3847.37

Nimag = 0

H	5.74276297	0.29278054	8.16002441
H	6.32588014	4.85318588	12.32453054
H	3.37017643	4.77008672	5.07531932
H	6.31815717	1.64547705	7.13618878
H	9.74466478	3.69256016	10.70579918
C	9.49473562	4.02756191	9.68840725
H	6.23061321	3.77909676	7.97499933
C	6.25664837	1.25776015	8.16356729
H	6.61485904	2.01339515	11.14726459
H	7.27170838	1.13736761	8.56087233
H	3.68456116	4.82543750	10.96215565
C	6.29241643	3.74324093	12.37490855
O	5.75873482	3.18666916	13.32619816
O	8.29788355	3.40500766	9.19073286
H	8.05417101	2.90084306	11.66121919
O	5.46469831	2.13539801	8.98966741
H	2.75874797	3.32668501	5.94339322
C	5.90217386	3.50317639	8.98740580
C	7.02988170	3.01723110	11.27395959
O	4.24554663	4.09082890	10.65580527
C	4.69833065	4.39769743	9.31221561
H	7.14436087	4.85540905	10.12157779
C	7.10509439	3.76959664	9.93034321
H	3.24750460	3.15235557	8.29308086
H	2.67319485	4.80356006	8.67241315
H	2.02808936	4.93334284	6.25026754
C	2.96652289	4.40447071	6.02304820
C	3.53795873	4.21199960	8.32812008
H	9.39275246	5.12360044	9.68950744
O	3.96087890	4.66084017	7.03061579
H	5.04287276	5.44011409	9.26130474
H	10.30268905	3.73540421	9.01281596

TS2'
E = --4648.64
H = −4440.31
G = −4485.72
***N*imag** = 1, −1016.3681

H	5.62418502	0.32964443	8.03768759
H	6.09940167	5.66865170	11.26075806
H	3.24290413	4.43073015	5.07776881
H	5.56733531	1.71855592	6.91142086
H	9.81063738	3.37450648	10.83818070
C	9.69758744	3.39162512	9.74520739
H	6.40305475	3.72063999	7.79629074
C	6.02667654	1.32507754	7.82999049

H	6.63752022	2.64053965	11.45064746
H	7.11388980	1.26199590	7.69636195
H	4.20954155	5.19847204	12.71726381
C	5.93441822	4.65565060	11.67289285
O	5.42517450	4.58727470	12.87174424
O	8.39531165	2.93867130	9.33062212
H	7.88220126	3.84485734	11.84541147
O	5.68932384	2.14730997	8.96475303
H	2.71034540	3.08293024	6.13156112
C	6.11382068	3.51792723	8.83477432
C	6.98674706	3.65636894	11.23925367
O	4.58266116	4.24261137	10.56999147
C	4.93648567	4.45042550	9.19063299
H	7.62774094	4.85459992	9.55048108
C	7.32333143	3.81109600	9.74202787
H	3.39581828	3.13532664	8.43358888
H	2.86180189	4.82020071	8.70208497
H	2.00720612	4.71693860	6.34263270
C	2.92286430	4.16184741	6.08761167
C	3.69124953	4.19024195	8.34149277
H	9.89145711	4.41187833	9.38061032
O	4.00092001	4.50766953	6.97541154
H	5.26329275	5.49154521	9.03176749
H	10.42178677	2.70298757	9.30241847
C	3.15385905	6.92708256	11.99898150
H	2.93956228	7.36055102	12.97808585
H	4.08248889	7.34977179	11.59939510
H	2.32703741	7.12763725	11.31426452
H	3.71685455	4.96749405	11.20337304
O	3.27225048	5.47732141	12.15394959

β-TS3-1MeOH
E = −10103.73
H = −9729.22
G = −9815.62
***N*imag** = 1, −99.7856

C	1.59175075	5.43833833	7.81974977
C	1.79015545	5.61350255	6.44414183
C	2.96205203	6.21935483	5.98073914
C	3.93906794	6.66038185	6.87960368
C	3.76671339	6.46791201	8.25397923
C	2.60035081	5.84596366	8.69477555
H	1.03435706	5.29272829	5.73655538
H	3.10449274	6.35805059	4.91288248
H	4.83715011	7.15134427	6.51523270

H	4.51104027	6.79596150	8.96843422
Se	2.26954866	5.39702840	10.56915804
C	0.33020253	4.84995930	8.47707765
C	-0.96256995	5.57281971	7.93820055
C	0.26357528	3.29862181	8.18994138
O	0.35934566	5.03923552	9.84858178
C	1.66272249	7.10433476	11.32125388
C	1.24022099	8.14824393	10.50088501
C	0.80535120	9.33180989	11.10585370
C	0.79280775	9.44807937	12.50170250
C	1.21808448	8.38317828	13.30364929
C	1.66083223	7.19320582	12.71453177
H	1.24263547	8.04997880	9.42090820
H	0.47506150	10.15983534	10.48384925
H	0.45037859	10.37032017	12.96400702
H	1.20693711	8.47175421	14.38678013
H	2.00148589	6.36666556	13.33170331
F	-0.80993108	6.92538599	8.03521886
F	-2.06199656	5.23606250	8.66284227
F	-1.23768712	5.28677160	6.62733730
F	0.29295353	2.98172342	6.86109659
F	-0.84699258	2.72415622	8.72339731
F	1.35063863	2.69976845	8.76947141
F	2.39495755	2.37362346	12.32243209
O	4.01638476	4.61503139	13.54705038
O	4.62791588	2.33693171	14.33916232
S	3.67438631	3.46849623	14.42320212
F	1.14013187	3.72181957	13.53531249
F	1.63223263	1.70652066	14.28084205
C	5.23438016	3.60602332	10.74304110
C	6.92282223	4.49174512	9.10908981
C	6.59775636	4.15802237	10.57715735
O	4.53767074	3.08829588	9.79477530
C	5.01711609	3.06820179	8.36553765
C	6.52850217	3.27894821	8.25176539
H	4.75680379	3.56975563	11.71115644
H	6.32252942	5.35780283	8.78931544
O	8.30698716	4.76719296	8.90509318
H	7.29771181	3.38097218	10.91741458
H	6.70468688	5.00924852	11.25297099
O	4.18187579	5.82715340	10.97603345
O	4.76493879	1.78984928	6.39913049
C	4.50959611	1.74372920	7.80535175
H	4.48844146	3.90487185	7.90120213
O	7.20488917	2.09532321	8.68434892
H	8.09512313	6.85599550	9.00852401

C	4.36521068	0.56968304	5.74395326
H	4.59819734	0.69212038	4.68385609
H	3.28659571	0.39449588	5.86765671
H	4.91956923	-0.28969606	6.14835783
H	3.43477011	1.64684468	8.00973281
H	5.04400447	0.90893277	8.27869980
C	8.71906413	6.05202991	9.42467302
H	8.66724063	6.08076883	10.52075945
H	9.75518605	6.19194284	9.11011423
H	6.73925734	3.48620031	7.19485273
H	9.12127735	2.49356702	7.91145662
C	8.33944815	1.72732855	7.86654530
H	8.71601798	0.78769161	8.27742750
H	8.02627217	1.57330525	6.82514923
H	7.22984773	0.11657006	12.69559510
H	4.21209735	5.68030302	11.94733292
H	7.52821590	0.85354374	11.09341646
H	7.70622771	1.83342267	12.57815882
O	5.74188286	1.39732346	11.93816693
C	7.13108810	1.03050829	12.09569517
H	5.38451540	1.65143199	12.82229454
O	3.24030517	3.82495833	15.77862331
C	2.11390407	2.77239514	13.59746151

β-TS3-2MeOH

E = −10789.25

H = −10381.12

G = −10470.01

Nimag = 1, −47.6480

C	-1.13397734	6.20988305	12.87370118
C	-2.16411023	7.15817535	12.83641617
C	-2.29772486	8.00022127	11.72747990
C	-1.41149104	7.90343734	10.65021496
C	-0.37726325	6.96134365	10.67527204
C	-0.25631031	6.13455612	11.78927919
H	-2.85865694	7.24535680	13.66402952
H	-3.09878841	8.73426409	11.70878907
H	-1.51893150	8.55995331	9.79083307
H	0.33157806	6.87728401	9.85958657
Se	1.14707317	4.76657988	11.94655164
C	-0.87426522	5.22268230	14.02287068
C	-0.58158849	6.01002853	15.35812853
C	-2.11732520	4.26538254	14.19269077
O	0.21853501	4.41623877	13.75681749
C	2.52423727	5.82562897	12.87576248

C	2.64926870	7.19178638	12.62900024
C	3.67884979	7.89718626	13.26402995
C	4.55778949	7.23481150	14.12983684
C	4.40073954	5.86616076	14.37624874
C	3.37450778	5.14935561	13.75003142
H	1.96943519	7.70460748	11.95720775
H	3.78757658	8.96328288	13.08164207
H	5.35701982	7.78665137	14.61772166
H	5.07359950	5.35147487	15.05740622
H	3.24573903	4.08934267	13.93389797
F	0.45075310	6.87971851	15.15722873
F	-0.21448239	5.17388569	16.36893546
F	-1.64588089	6.74564403	15.81124702
F	-3.27429098	4.91481305	14.53436698
F	-1.89909770	3.31256926	15.14049478
F	-2.35299919	3.62004668	13.01285849
O	2.00955171	5.32209215	10.24260391
C	1.66060213	4.42574001	9.16268293
H	2.28687942	4.69538334	8.30462194
H	1.84558719	3.37935323	9.43198698
H	0.60693867	4.54565292	8.87355846
H	3.61158646	5.90347720	10.20889802
C	5.59044162	3.87482352	10.87998512
C	7.25551197	4.63247434	9.15745681
C	6.94029605	4.46356814	10.65831152
O	4.89542659	3.29589518	9.96831452
C	5.39265556	3.08969659	8.56675723
C	6.90253776	3.32588108	8.43670335
H	5.01804401	4.05807225	11.78232477
H	6.63016458	5.43801959	8.74670349
O	8.63516938	4.92121947	8.92232166
H	7.67791089	3.78127452	11.09637771
H	6.98611611	5.40695128	11.20791620
O	4.55442529	6.23173245	10.15352075
O	5.13384644	1.53116824	6.79715332
C	4.90758170	1.68960059	8.20208000
H	4.84774771	3.84457865	7.99196679
O	7.64596799	2.24323038	9.00765167
H	8.35333534	6.99576678	8.73100618
C	4.77772191	0.20679820	6.34984618
H	4.97695060	0.17328371	5.27646243
H	3.71327066	0.00428081	6.53605684
H	5.38424111	-0.55353146	6.86325788
H	3.84045369	1.59602677	8.44821804
H	5.46384025	0.93981114	8.78151200
C	8.99827264	6.27845637	9.25864659

H	8.93386877	6.45882736	10.34020204
H	10.03338868	6.40951678	8.93564340
H	7.12129162	3.43109559	7.36744222
H	9.13188049	2.07758545	7.53240427
C	8.38206116	1.45887863	8.04328761
H	8.88263446	0.67052740	8.61025210
H	7.70934019	1.01132600	7.30090136
H	6.55965888	1.42469459	14.06344687
H	4.60738209	6.90452036	10.85549336
H	6.89360991	3.14416380	13.73010774
H	5.20001940	2.56139752	13.81589174
O	6.29004677	2.06133359	12.08468595
C	6.22161882	2.30957749	13.51183836
H	5.56153010	1.42200067	11.85541743
O	3.06506459	2.48700927	12.24799343
C	1.97544144	0.15568985	12.87991555
F	2.67861951	0.09052007	14.04473817
F	1.76839546	-1.11576684	12.43842359
F	0.76716016	0.71829537	13.14383375
S	2.91317617	1.17779938	11.58913106
O	4.19338989	0.43340576	11.43498125
O	2.03850271	1.14184149	10.40812560

α-TS3-2MeOH

E = −10106.80

H = −9732.21

G = −9818.15

***N*imag** = -39.028

C	1.12169812	7.41096669	9.71995754
C	0.25274206	8.08425263	8.85221672
C	0.67001328	8.40708064	7.55727497
C	1.95009858	8.06070817	7.11662531
C	2.83038217	7.38837721	7.97074094
C	2.39775480	7.08337851	9.25731391
H	-0.74352399	8.35734320	9.18006159
H	-0.01245230	8.92855586	6.89168849
H	2.26827908	8.30676190	6.10716125
H	3.82465485	7.10596110	7.64919187
Se	3.52077559	6.14633555	10.56225833
C	0.78855133	7.00309493	11.16275557
C	0.49433444	8.28873550	12.02976041
C	-0.44320893	6.01737276	11.17615559
O	1.84758530	6.32612781	11.74797988
C	4.34514806	7.67202242	11.50693441
C	4.82472104	8.77447914	10.80215147

C	5.44062620	9.80879798	11.51576927
C	5.57835683	9.72149391	12.90654202
C	5.09082833	8.60416867	13.59376968
C	4.46120829	7.56940482	12.89330476
H	4.72323190	8.83269205	9.72330144
H	5.81170333	10.68000234	10.98213401
H	6.06230835	10.52582104	13.45442701
H	5.19186048	8.53715280	14.67391604
H	4.05137310	6.71604568	13.42502900
F	1.54444303	9.15519509	11.93245970
F	0.34240197	7.98586153	13.34918136
F	-0.62796576	8.97073498	11.64057504
F	-1.59998404	6.57407336	10.69771302
F	-0.71041387	5.56599487	12.43616630
F	-0.16445777	4.93411503	10.40425565
F	0.77990684	3.26134093	5.73447268
O	2.64763594	3.69735945	9.06156223
F	0.50912240	4.85603700	7.23061673
O	2.97170976	1.92294477	7.35149351
S	2.01178111	2.78953142	8.08793598
F	2.43639156	4.64578425	6.18110628
C	5.68661778	3.84803342	9.14589094
C	8.21344304	4.07337158	9.34874246
C	6.96484833	4.11923494	8.43798354
O	5.58885907	3.55035811	10.39910194
C	6.77249384	3.61826464	11.31964822
C	7.85271828	4.54665413	10.76111392
H	4.73508201	3.80841086	8.63227680
H	8.56024241	3.03128457	9.43470740
O	9.27504308	4.89062104	8.85849215
H	6.87177581	5.10443321	7.96588304
H	7.02613014	3.38895748	7.62374352
O	5.03774820	6.11637642	9.27232430
O	7.21155432	4.04559989	13.61769139
C	6.16442530	4.05853483	12.64850643
H	7.14875426	2.59347425	11.38415777
O	7.37796090	5.90772560	10.73013575
H	10.25146839	3.33466849	7.83587457
C	6.75681971	4.53527862	14.89809378
H	7.61256169	4.48564475	15.57412250
H	5.93901232	3.91213609	15.28583253
H	6.41184200	5.57596272	14.81400494
H	5.35131752	3.37187777	12.92557585
H	5.74736763	5.07065571	12.54215450
C	9.89765622	4.36134411	7.66504303
H	9.20659771	4.37196295	6.81185165

H	10.74697405	5.01230414	7.44813661
H	8.73070587	4.46858203	11.40953211
H	9.11693336	6.92119861	11.32043733
C	8.06876774	6.81917439	11.62274794
H	7.55737822	7.77828552	11.52953214
H	8.00986845	6.46407588	12.65727915
H	4.80886136	-0.35047747	9.22802380
H	5.84437610	6.28558704	9.81743942
H	6.27784719	0.37183648	9.93745562
H	4.67831715	1.10102343	10.26348847
O	5.50475398	1.43245395	8.35300504
C	5.29497748	0.59114984	9.51126382
H	4.61559097	1.58228368	7.94313694
O	0.79529348	2.10566224	8.54903181
C	1.39546660	3.95887765	6.72952463

α-TS3-2MeOH

E = −10789.95

H = −10381.10

G = −10470.57

***N*imag** = -62.5979

C	-1.01758165	6.09873111	12.75143043
C	-1.72337490	6.95585056	13.60561697
C	-1.29080122	8.27356391	13.78602722
C	-0.15617685	8.74578396	13.11903083
C	0.55890372	7.89927673	12.26474997
C	0.11583733	6.58945704	12.09860457
H	-2.60327077	6.60252778	14.13095533
H	-1.84404668	8.93060886	14.45176494
H	0.17723916	9.77034499	13.26177414
H	1.44816520	8.23993154	11.74732850
Se	1.03297969	5.29617723	10.92791166
C	-1.37950099	4.63213711	12.46784979
C	-1.33911894	3.79902051	13.80813562
C	-2.80723741	4.55260714	11.80014175
O	-0.49184749	4.05776613	11.57742634
C	2.22893912	4.48066672	12.26259779
C	2.91095168	5.27998031	13.17722299
C	3.67605049	4.65561250	14.16897440
C	3.74753579	3.25949262	14.22736640
C	3.07390805	2.47922807	13.28159588
C	2.30254769	3.08934361	12.28810602
H	2.83913238	6.36158436	13.13723718
H	4.20247648	5.26384136	14.90024820
H	4.32785421	2.77929110	15.01084863
H	3.13806614	1.39486716	13.31974769

H	1.75340947	2.49317539	11.56828679
F	-0.12902395	3.96631511	14.41580063
F	-1.49643301	2.46556305	13.58068380
F	-2.29890498	4.16731154	14.71672648
F	-3.81937456	5.03198172	12.58962715
F	-3.13800192	3.27563627	11.46028601
F	-2.81005559	5.29319120	10.65301087
O	2.46541179	6.59328111	10.48317150
C	2.17197945	7.19233403	9.19642012
H	3.00943354	7.85260933	8.94934754
H	2.07314306	6.42912805	8.41394423
H	1.25181329	7.79157321	9.23966284
H	4.12714282	6.15330433	10.39988463
C	5.58489723	3.78392321	9.82011279
C	8.09734263	4.13904717	9.42185673
C	6.66337823	4.05532293	8.83319161
O	5.79029740	3.40735057	11.03654022
C	7.18965456	3.33289406	11.57751526
C	8.06423879	4.40561684	10.93589340
H	4.54123267	3.74324477	9.52312077
H	8.59334585	3.16461624	9.28986519
O	8.87963918	5.15465395	8.79187684
H	6.40130264	4.98634661	8.32247042
H	6.58888262	3.25564857	8.08628942
O	5.05453754	6.01531232	10.05260003
O	8.32388305	3.28607715	13.65849312
C	7.02487932	3.45308025	13.08741530
H	7.55294904	2.33690937	11.30689824
O	7.52740618	5.69315983	11.28614954
H	9.78776971	3.86661141	7.40225341
C	8.29582377	3.43983958	15.09230769
H	9.32079217	3.30319545	15.44363129
H	7.64316380	2.68457727	15.55396132
H	7.94042948	4.44334462	15.36817324
H	6.33288836	2.67605777	13.44072663
H	6.60538523	4.43710398	13.34020316
C	9.24928784	4.82462120	7.43388212
H	8.36953725	4.76902804	6.77897722
H	9.90399884	5.62764042	7.08916367
H	9.07503367	4.29918614	11.34692842
H	9.05931513	6.93969161	10.56085807
C	8.52523384	6.72500235	11.49117594
H	7.97912765	7.61008361	11.82486717
H	9.23403726	6.41368583	12.26920267
H	4.01672028	-0.28300874	9.67527887
H	5.70952740	6.17066142	10.76461800

H	5.23549008	0.38936575	10.79084780
H	3.73456949	1.29708899	10.46467207
O	5.19720593	1.30572871	8.95064998
C	4.49508407	0.64036647	10.02631586
H	4.52351437	1.57151006	8.27834139
O	2.60714913	3.59406188	9.08509934
C	0.79231399	1.98867876	8.02069888
F	1.14010158	0.97344299	8.86106026
F	0.35707749	1.44222668	6.85308553
F	-0.23548462	2.67286245	8.58417691
S	2.26929134	3.13752065	7.72136841
O	3.28987584	2.23865816	7.12230196
O	1.74277218	4.16727022	6.81437738

β-P
E = −4342.22
H = −4146.29
G = −4187.74
Nimag = 0

C	2.96177098	15.77850768	-5.30290232
C	2.71423441	18.24695559	-5.64736420
C	3.71063553	17.07462572	-5.61159406
O	1.91610109	15.55035524	-6.27688242
C	0.92962451	16.60756739	-6.27927654
C	1.57593493	17.96117498	-6.64547428
H	2.48886239	15.82144608	-4.30239961
H	2.24881189	18.35390771	-4.65155725
O	3.33113588	19.49950876	-5.99943122
H	4.20053314	16.97797848	-6.58687829
H	4.47408799	17.23488492	-4.84377664
O	3.85782050	14.69671380	-5.36695038
O	-0.98033006	15.18659065	-6.64458715
C	-0.17410287	16.21223300	-7.24794045
H	0.49431169	16.70119397	-5.26966270
O	2.03567994	17.88917146	-8.00833542
H	3.56627007	20.15483515	-4.01744307
C	-1.97108051	14.68251626	-7.55605681
H	-2.53640614	13.91497283	-7.02097241
H	-1.49821391	14.23739305	-8.44500180
H	-2.65589227	15.48204695	-7.88003755
H	0.26794226	15.84920685	-8.18661393
H	-0.79586483	17.09331572	-7.47723328
C	4.14439161	20.05629624	-4.94881147
H	5.03684804	19.44602411	-4.75442559
H	4.45682587	21.04661674	-5.28979111

H	0.81439250	18.74917115	-6.55287697
H	2.57538402	19.89914408	-8.32913370
C	1.91641345	19.12621210	-8.74023010
H	2.20454046	18.90178651	-9.77104753
H	0.87703990	19.48739070	-8.72567038
H	2.97887006	13.59831803	-3.80542718
C	3.30709060	13.45927175	-4.84502024
H	2.46315693	13.11954988	-5.45497194
H	4.11456463	12.72516248	-4.88219582

α-P

E = −4343.71

H = −4147.63

G = −4188.87

Nimag = 0

C	3.03556218	15.85983899	-5.22123078
C	2.70973849	18.33664934	-5.61847335
C	3.73119492	17.19103574	-5.52578175
O	1.96870797	15.59141562	-6.14510966
C	0.97122510	16.64789492	-6.21596346
C	1.61155698	17.98414524	-6.63819728
O	2.57692175	15.90213994	-3.86795039
H	2.21649881	18.46931875	-4.64240960
O	3.31305898	19.58858338	-6.00410903
H	4.24752549	17.09864942	-6.48702427
H	4.47627667	17.38502550	-4.74865580
H	3.71528555	15.00654796	-5.35445174
O	-0.89556098	15.16508143	-6.54345889
C	-0.10991345	16.18816984	-7.17953992
H	0.52186911	16.78438066	-5.22217841
O	2.11717303	17.83945192	-7.98000060
H	3.43367718	20.35318506	-4.05139378
C	-1.89475310	14.63394933	-7.43065077
H	-2.44372829	13.87071717	-6.87286467
H	-1.43140702	14.17728092	-8.31879877
H	-2.59237945	15.42078578	-7.75826588
H	0.34668517	15.80182594	-8.10207439
H	-0.75120717	17.04519904	-7.44415849
C	4.06204463	20.21275724	-4.94399495
H	4.94935270	19.62628421	-4.66860951
H	4.38198875	21.18766120	-5.32087138
H	0.83778459	18.76580982	-6.61825773
H	2.71627394	13.82956671	-3.54935705
C	2.03863257	19.03818060	-8.77767819
H	2.34990591	18.75561676	-9.78732204

H	1.00552992	19.41591045	-8.81018304
H	2.69907380	19.82151522	-8.38944721
C	1.99760058	14.65268852	-3.42475890
H	1.76389286	14.77967747	-2.36529048
H	1.08295909	14.42239805	-3.98323412

1e*
E = −5769.39
H = −5591.21
G = −5649.16
***N*imag** = 0

C	-9.21318768	-8.35458992	7.79722494
C	-9.88339456	-9.55959287	7.56071969
C	-9.74158550	-10.61510497	8.46682959
C	-8.94847923	-10.47594123	9.61041513
C	-8.27793858	-9.27495950	9.86623821
C	-8.42110483	-8.24282586	8.94470619
H	-10.50711446	-9.67827441	6.68229926
H	-10.25879758	-11.55102538	8.27678167
H	-8.84872097	-11.30051809	10.31035931
H	-7.66929325	-9.14978812	10.75302299
Se	-7.60382231	-6.47866032	9.09005497
C	-9.28536803	-7.10981633	6.91942988
C	-8.87418327	-7.41761965	5.43279472
C	-10.72168321	-6.46485618	7.01129270
O	-8.37336351	-6.12238944	7.37768685
C	-5.79985003	-6.84688361	8.42673848
C	-5.49007834	-8.05401331	7.79707823
C	-4.18894479	-8.23283560	7.32039101
C	-3.23408733	-7.21985644	7.47767645
C	-3.56969617	-6.02028867	8.11530473
C	-4.86573917	-5.82014534	8.60143405
H	-6.23097508	-8.83625777	7.67796290
H	-3.92432822	-9.16485658	6.82842001
H	-2.22469454	-7.36855341	7.10339206
H	-2.82721178	-5.23649182	8.23783641
H	-5.14040914	-4.89675061	9.10169498
F	-7.67343873	-8.05962249	5.41726176
F	-8.74269175	-6.28098553	4.70439435
F	-9.78154346	-8.21568925	4.80235198
F	-11.70066729	-7.31016412	6.58829019
F	-10.81196912	-5.32244899	6.28438235
F	-10.97712081	-6.14962350	8.31350068
F	-9.63277918	-4.48074239	10.83720663
F	-9.47236039	-2.29558316	10.59192085

F	-9.67724334	-3.16579520	12.60636475
S	-7.22714307	-3.46570516	11.51259770
O	-6.80323741	-3.66845396	10.11388347
O	-6.82469462	-2.20246818	12.14041824
O	-7.04447627	-4.66573501	12.38295571
C	-9.11676248	-3.34058135	11.38268569
O	-6.75905076	-7.02667341	11.23719349
H	-5.80356916	-7.21735053	11.17310805
H	-6.83172352	-6.13265639	11.70899842

TS1-S_N2-f 1e*-H_2O
E = −5761.9
H = −5583.81
G = −5644.21
Nimag = −62.6544

C	-9.29073636	-8.40197172	7.85962877
C	-9.93293805	-9.63268537	7.69102746
C	-9.72156967	-10.64867171	8.62917217
C	-8.87988512	-10.45048597	9.72911592
C	-8.23163922	-9.22454405	9.91659267
C	-8.45582790	-8.23280528	8.96837126
H	-10.58480351	-9.80229239	6.84181142
H	-10.21951151	-11.60439939	8.49487099
H	-8.72211564	-11.25037251	10.44668208
H	-7.56117073	-9.05273791	10.75113664
Se	-7.72094580	-6.43770616	9.03458624
C	-9.41746084	-7.19209117	6.94477170
C	-9.00284362	-7.53122382	5.46577441
C	-10.86667007	-6.57952791	7.03505516
O	-8.52313802	-6.15078730	7.35776363
C	-5.89886648	-6.74742784	8.47824978
C	-5.50479915	-7.92930888	7.84022912
C	-4.16730361	-8.05966804	7.47021195
C	-3.25564814	-7.02807827	7.74249272
C	-3.67175509	-5.85623990	8.38525473
C	-5.00796586	-5.70197637	8.76231268
H	-6.21337680	-8.72596950	7.64382072
H	-3.83404939	-8.96746719	6.97520867
H	-2.21413628	-7.14339637	7.45476410
H	-2.96001173	-5.06354791	8.59752819
H	-5.34800541	-4.80782600	9.27594720
F	-7.77631397	-8.12100442	5.46533756
F	-8.92638344	-6.41657941	4.69871161
F	-9.88194473	-8.38831226	4.87959725
F	-11.82063422	-7.46529518	6.64482774
F	-10.99257415	-5.46206350	6.27745646

F	-11.11460319	-6.23609805	8.33015762
F	-9.94206483	-4.26305011	10.46138042
F	-9.51084456	-2.40085844	11.56137059
F	-10.26491349	-4.16830439	12.64033843
S	-7.67254402	-4.33670523	11.90929967
O	-6.95510460	-3.83089326	10.72340735
O	-7.29169205	-3.75110930	13.20037999
O	-7.83406693	-5.82136357	11.92264852
C	-9.45646834	-3.75555757	11.63045880
O	-5.90073527	-7.74789228	11.57748730
H	-5.03992038	-7.35615791	11.34825160
H	-6.45917389	-6.98815463	11.86560591

H_2O

E = −322.93
H = −307.79
G = −321.26
***N*imag** = 0

O	0.00000000	-0.00000000	0.59189124
H	0.00000000	0.76824875	-0.00744562
H	-0.00000000	-0.76824875	-0.00744562

a-R 2-deoxy-3,4,6-tri-*O*-methyl-α-D-glucopyranoside

E = −3988.11
H = −3809.99
G = −3849.04
***N*imag** = 0

C	1.92119999	7.09922397	-0.69758645
C	1.68339679	9.58867463	-1.13765788
C	2.65149956	8.39630635	-1.04131082
O	0.83553899	6.85706225	-1.60495679
C	-0.14776939	7.92606448	-1.67616540
C	0.51212774	9.26818663	-2.07358471
O	1.47377164	7.18001537	0.66417463
H	1.26574032	9.82219869	-0.14710516
O	2.35113883	10.76103177	-1.65246428
H	3.14148607	8.26157171	-2.01268464
H	3.42871251	8.58545640	-0.29399926
H	-0.59886733	10.76161182	-4.02873695
O	-1.99887488	6.44450469	-2.12564846
C	-1.19747974	7.49722418	-2.68840169
H	-0.62314408	8.04985307	-0.69336715
H	0.88519477	9.19870575	-3.10675374
H	2.38850903	11.81986286	0.15871066
C	-3.01237670	5.99894398	-3.04448871
H	-3.57612660	5.21016096	-2.53975454

H	-2.56131923	5.59634740	-3.96445095
H	-3.69221226	6.82284930	-3.31172256
H	-0.70187951	7.14610271	-3.60872651
H	-1.83217359	8.36007640	-2.93726776
C	3.06735743	11.49944008	-0.64538234
H	3.88650522	10.90984188	-0.20967706
H	3.48565830	12.37930956	-1.14106263
O	-0.50722744	10.28360476	-1.98383109
C	-0.47580961	11.25340993	-3.05243904
H	-1.31840556	11.92856626	-2.88023319
H	0.46318700	11.81863232	-3.04299774
H	2.57040828	6.22646270	-0.83121726
H	1.08116734	6.31757978	0.89529240

α-TS3-Glc

E = -10104.64

H = -9730.15

G = -9814.76

***N*imag** = -26.8061

C	1.17935349	7.42616449	10.30085114
C	0.08380392	8.10004661	9.74575250
C	0.09479757	8.45614303	8.39370509
C	1.19432302	8.14792732	7.58726861
C	2.29249576	7.46957591	8.12613399
C	2.26034350	7.12035934	9.47241644
H	-0.77287255	8.34996469	10.36098653
H	-0.75940220	8.97929532	7.97260179
H	1.20229510	8.43029123	6.53798069
H	3.15035418	7.22645041	7.51111290
Se	3.71206321	6.11404816	10.34414514
C	1.30260617	6.99898904	11.77164846
C	1.19261673	8.26260368	12.71019122
C	0.18657046	5.93846128	12.11727275
O	2.52361473	6.39721386	12.01776313
C	4.91929028	7.59729095	10.90451370
C	5.66015432	8.30035002	9.95443319
C	6.53726743	9.29953703	10.39244030
C	6.67287581	9.57350561	11.75773409
C	5.92518889	8.85152057	12.69425366
C	5.03686548	7.85630940	12.27145889
H	5.56384639	8.06964520	8.90020077
H	7.11465621	9.85932223	9.66098163
H	7.36063373	10.34638915	12.09127723
H	6.02600545	9.05997859	13.75631509
H	4.43165207	7.31548692	12.98964752
F	2.10039442	9.20341510	12.31660742

F	1.47011140	7.95258407	14.00747455
F	-0.04000101	8.85987695	12.69444841
F	-1.09050533	6.41592051	11.97734451
F	0.30313249	5.48109258	13.39710002
F	0.31069463	4.86757997	11.28731385
F	-0.03126133	2.42909300	8.04586809
O	3.31896229	4.08140719	7.15425037
F	0.34813766	4.54213679	7.54410333
O	2.92062180	1.69470108	7.71232670
S	2.56911731	3.10599658	7.99116071
F	0.71335604	2.97032207	6.04193097
C	5.99961935	3.73515734	9.08374341
C	8.37198748	4.55579345	9.60110560
C	7.31090213	4.13136580	8.55066706
O	5.73104871	3.55484322	10.32247786
C	6.76706916	3.82139737	11.38876150
C	7.70741472	4.94618573	10.92676335
H	5.17747186	3.53692690	8.41368383
H	9.05028830	3.71408882	9.80681427
O	9.12142528	5.67998834	9.14097802
H	7.11688614	4.96127805	7.85708836
H	7.65307504	3.29571345	7.92306557
O	4.76752141	5.93167998	8.69348993
O	5.20556645	3.02651418	13.11783380
C	5.99159840	4.13023283	12.66754238
H	7.30220254	2.87270574	11.49415319
H	7.12990787	5.87134071	10.78589840
H	10.81747333	4.58503600	8.56358636
C	3.80568177	3.08524602	12.73016795
H	3.31668520	2.25911373	13.25139933
H	3.67744733	2.96767616	11.64922566
H	3.35834360	4.03699651	13.04405051
H	5.37283105	5.02635255	12.53088484
H	6.73847517	4.34829364	13.43741739
C	10.12333135	5.33507109	8.15924106
H	9.66843534	4.94953164	7.23637512
H	10.66460077	6.25661541	7.93538848
H	9.44506666	6.97675876	11.36779409
H	4.74517115	1.26314159	8.11071442
O	2.49116229	3.46805517	9.41420423
C	5.58971082	0.46974963	9.71196961
C	0.78896513	3.27106691	7.36501064
H	5.14274405	-0.52316369	9.58042869
H	4.19643027	5.38140826	8.10094668
H	6.61297342	0.35633127	10.07958453
H	5.00096675	1.03489280	10.44593224

O	5.66697780	1.17419499	8.44752578
O	8.67907022	5.11017600	11.96231463
H	9.73235111	6.46922392	13.06357881
H	8.11213901	7.04423824	12.56585929
C	9.00689297	6.49476877	12.24786220

β-TS3-Glc

E = −10102.67

H = −9729.01

G = −9813.38

***N*imag** = -28.0230

C	1.43236364	5.25109735	7.98835255
C	1.53597849	5.29140526	6.59246216
C	2.66889318	5.84971308	5.99332949
C	3.70525973	6.36961654	6.77606985
C	3.62958244	6.30852564	8.17129451
C	2.49508885	5.74218370	8.74835635
H	0.73661108	4.90130528	5.97306009
H	2.73697769	5.88501749	4.90968504
H	4.57706496	6.81588517	6.30584761
H	4.42650637	6.69122533	8.79646028
Se	2.29207946	5.47991754	10.67457226
C	0.22053873	4.73063651	8.78262803
C	-1.10663290	5.40313557	8.26243343
C	0.13763080	3.15805543	8.65840987
O	0.33843330	5.05525078	10.12309118
C	1.69554348	7.23677275	11.30736858
C	1.24881898	8.21727721	10.42431005
C	0.82780673	9.44247198	10.95153656
C	0.85163302	9.66165077	12.33491368
C	1.29975169	8.65859320	13.20176065
C	1.72984766	7.42885236	12.68971133
H	1.22258519	8.03843327	9.35482775
H	0.47925283	10.22244541	10.27931062
H	0.51924864	10.61535687	12.73680388
H	1.31587169	8.82657954	14.27546021
H	2.08488610	6.64790341	13.35697093
F	-0.94794377	6.75810000	8.20672471
F	-2.15468763	5.15444292	9.09217517
F	-1.47460305	4.98534913	7.01026866
F	0.09005583	2.71020173	7.36647109
F	-0.95867738	2.65510534	9.29813336
F	1.23671378	2.60739282	9.24195517
F	2.13576410	2.47302482	12.42049128
O	4.09161876	4.56817381	13.43396307
O	4.60539099	2.22478834	14.09367379

S	3.77338038	3.43134549	14.32931326
F	1.18118491	3.92525158	13.77788176
F	1.57668958	1.86845631	14.46584134
C	5.17861292	3.58747431	10.62690478
C	7.06927919	4.49580500	9.23249237
C	6.57433942	4.08052608	10.63171361
O	4.54563502	3.21628309	9.57667550
C	5.19711057	3.13133402	8.21674229
C	6.71277461	3.38925001	8.23459973
H	4.58371990	3.59290252	11.52982595
H	6.56984688	5.42610169	8.92108040
O	8.48432807	4.67460759	9.21387110
H	7.22181732	3.27858996	11.01280715
H	6.63653490	4.89477807	11.35863804
O	4.20680361	5.97723111	10.92738790
O	3.45240101	1.83689179	7.23758249
C	4.81294598	1.77549528	7.65486779
H	4.70113908	3.92596507	7.65496770
H	7.25268040	2.47209629	8.51600748
H	8.42661886	6.77373042	9.22431148
C	2.92582205	0.54366152	6.89034962
H	1.88773579	0.70106203	6.59132925
H	2.96083628	-0.13726362	7.75373556
H	3.48802203	0.09760498	6.05615145
H	4.96152859	0.99980375	8.42403201
H	5.47946615	1.55264679	6.80625023
C	8.91214178	5.94440418	9.75712228
H	8.69196079	6.02045600	10.83014464
H	9.99249037	5.99313265	9.60699147
H	9.11719630	3.62354910	6.96481971
C	6.79320466	0.79611739	11.71876347
C	2.06124654	2.88939782	13.71702227
O	3.56544216	3.77859785	15.73950439
H	5.15892990	1.60660462	12.51287714
H	6.76926103	-0.11851312	12.32356821
H	4.31384455	5.81448051	11.88871318
H	7.13099438	0.54775327	10.70908313
H	7.49955838	1.50304701	12.17667690
O	5.47013382	1.36604928	11.60355863
O	7.01860071	3.75306407	6.88575553
H	8.27501396	2.12275831	6.45326312
H	8.34296208	3.52990096	5.34971259
C	8.27429205	3.21992677	6.39466865

1f

E = −4911.57

H = −4758.65

G = −4810.11

Nimag = 0

C	-9.22094741	-8.24612216	7.78851152
C	-9.94818046	-9.43912305	7.71577468
C	-9.93256854	-10.32367840	8.79848289
C	-9.20245123	-10.02712831	9.95333665
C	-8.46932022	-8.83868665	10.04374305
C	-8.49260179	-7.97541582	8.95203811
H	-10.52093492	-9.67895574	6.82749165
H	-10.49720957	-11.24941206	8.73622082
H	-9.19691482	-10.71934224	10.79031532
H	-7.89438145	-8.60244571	10.93007310
Se	-7.55056811	-6.27257236	8.83670811
C	-9.15746447	-7.18320453	6.69761397
C	-8.55191794	-7.76482373	5.36617426
C	-10.57963333	-6.54353278	6.46679326
O	-8.31563018	-6.10996747	7.09023199
C	-5.78958384	-6.87447878	8.24438162
C	-5.44326869	-8.22468331	8.19585524
C	-4.15779882	-8.56074023	7.76431348
C	-3.25465166	-7.55819397	7.38840807
C	-3.62638785	-6.21031946	7.44513564
C	-4.90455938	-5.85452538	7.88469432
H	-6.14468842	-8.99869650	8.48623387
H	-3.86486091	-9.60634044	7.72282970
H	-2.25664852	-7.82935097	7.05429775
H	-2.92340355	-5.43319552	7.15813800
H	-5.19389299	-4.81032254	7.95252737
F	-7.34822316	-8.34202526	5.63231134
F	-8.34668179	-6.79207101	4.44143187
F	-9.35275325	-8.71342384	4.80334987
F	-11.49157852	-7.44816696	6.01455721
F	-10.53320639	-5.51949908	5.57640753
F	-11.03893741	-6.04500092	7.64864183
O	-4.41441922	-6.39321304	11.31296696
O	-5.77507742	-4.43453643	10.76094290
O	-6.85752847	-6.62018902	11.09283411
Cl	-5.69446240	-5.68962662	11.55593323
O	-5.91021097	-5.43641314	12.99993168

α-TS3-Gal-ClO_4^-

E = −9576.08
H = −9210.67
G = −9290.35
Nimag = -78.3817

C	1.14887067	7.38202107	9.76224081
C	0.22041671	7.98205097	8.90252047
C	0.56953865	8.25048502	7.57512697
C	1.84073503	7.92373975	7.09459669
C	2.78225038	7.32992649	7.94172143
C	2.41689507	7.08005387	9.26059819
H	-0.77055187	8.23662909	9.26014071
H	-0.15980434	8.71233615	6.91508975
H	2.10425138	8.12407243	6.05953013
H	3.76856053	7.05970067	7.58869142
Se	3.62358319	6.22649205	10.54509715
C	0.88593911	7.01842103	11.23205290
C	0.60279837	8.32888652	12.06603220
C	-0.32822023	6.01537106	11.33354154
O	1.97894104	6.37784103	11.79119996
C	4.42451448	7.78764181	11.45286304
C	4.88300936	8.87817881	10.71684481
C	5.49789494	9.93446870	11.39859268
C	5.65781694	9.87854933	12.78865113
C	5.19173019	8.77160565	13.50715507
C	4.56168023	7.71601304	12.83883667
H	4.76839502	8.90777905	9.63816636
H	5.85204915	10.79759365	10.84071840
H	6.14252430	10.69894739	13.31161657
H	5.31079495	8.72848345	14.58672786
H	4.16863889	6.87028194	13.39453363
F	1.63531565	9.20647405	11.90469685
F	0.49981604	8.06812832	13.39974436
F	-0.54199067	8.98336377	11.69396969
F	-1.52017185	6.54329352	10.91150226
F	-0.51653057	5.58570438	12.61559870
F	-0.07722796	4.91963017	10.56892084
H	4.54653565	1.64502965	7.86648742
O	2.48706802	3.68493004	9.16403169
O	3.29180610	4.19674743	6.91774272
O	2.75460859	1.88515281	7.52390252
Cl	2.35677743	3.33870337	7.71495520
O	0.95642027	3.52528105	7.25219316
C	5.74783054	3.87619302	9.04959921
C	8.25505548	4.06965349	9.47629099
C	7.08543763	4.14916041	8.46836670
O	5.52966666	3.56134335	10.28067083

C	6.64348697	3.55060670	11.29216321
C	7.77219838	4.48902493	10.87033436
H	4.85884927	3.94445087	8.43794705
H	8.60174331	3.02635720	9.55076799
O	9.34621047	4.91047269	9.10492907
H	7.03313145	5.14994634	8.02068136
H	7.20924892	3.44963728	7.63394825
O	5.09521339	6.24103530	9.22331185
O	6.94225762	3.84815740	13.62596367
C	5.95415175	3.93020905	12.59990981
H	6.99912549	2.51754763	11.32443528
O	7.28123885	5.84354136	10.88262735
H	10.41021871	3.40344445	8.09782499
C	6.44265302	4.33917471	14.88853511
H	7.25605341	4.23367008	15.60912419
H	5.57385561	3.75349916	15.21998906
H	6.15749432	5.39785469	14.80529515
H	5.11542978	3.24460279	12.78992247
H	5.56372040	4.95463980	12.52163100
C	10.06356175	4.43490756	7.94233330
H	9.44059581	4.47875673	7.03935353
H	10.92234300	5.09785307	7.81970506
H	8.58438468	4.37656754	11.59577027
H	9.08964460	6.91158953	10.96318387
C	8.15345636	6.81096299	11.52111323
H	7.60706121	7.75574554	11.51591272
H	8.35728091	6.50697508	12.55446263
H	5.07669499	-0.33292944	8.98758602
H	5.90277898	6.32927741	9.78512001
H	6.54124076	0.49923058	9.57392188
H	4.92441114	1.03847878	10.12326810
O	5.48769583	1.55666236	8.15893365
C	5.49788473	0.63695578	9.27737264

β-TS3-Gal-ClO_4^-

E = −9573.35

H = −9207.44

G = −9287.49

***N*imag** = -25.9283

C	1.64858520	5.43686609	7.86759960
C	1.88926067	5.61168120	6.49844083
C	3.07542164	6.21519561	6.06968157
C	4.02520504	6.65511328	6.99764907
C	3.81092035	6.46221477	8.36606631
C	2.63152832	5.84142217	8.77309352
H	1.15427191	5.29269237	5.76851069

H	3.24989103	6.35348810	5.00650560
H	4.93437288	7.14527319	6.66051028
H	4.53416144	6.78851918	9.10271376
Se	2.25236626	5.40233078	10.64169529
C	0.36235379	4.85418863	8.48273518
C	-0.90567952	5.59838280	7.91205711
C	0.28650247	3.30760275	8.17184665
O	0.35323855	5.02381101	9.85436276
C	1.60447917	7.11390137	11.35080297
C	1.25485330	8.16726215	10.50848335
C	0.80044288	9.35680895	11.08716032
C	0.69808814	9.47081843	12.47943720
C	1.05137686	8.39683400	13.30401505
C	1.51195144	7.20088812	12.74094630
H	1.32802038	8.07255874	9.43065787
H	0.52609656	10.19166213	10.44726010
H	0.34213688	10.39803493	12.92114918
H	0.97113487	8.48349288	14.38445545
H	1.79557230	6.36694687	13.37758581
F	-0.74130020	6.94800207	8.03019660
F	-2.03072656	5.26606789	8.59929371
F	-1.14608221	5.33207320	6.58966574
F	0.34898670	3.00504324	6.84026825
F	-0.84540728	2.73884016	8.66664344
F	1.34976836	2.68775249	8.77338019
H	5.47816157	1.65976125	12.76510219
O	3.86588480	4.62471452	13.69025987
O	4.83659328	2.46813368	14.33004375
Cl	5.02118477	3.96504757	14.40736273
O	6.29510777	4.33970239	13.72289467
O	5.03663567	4.39069647	15.83070557
C	5.23432709	3.53333738	10.69971815
C	6.89391155	4.44881613	9.04967442
C	6.57971303	4.12054803	10.52110305
O	4.51833174	3.04684424	9.75145793
C	4.98706697	3.02580999	8.31590505
C	6.49711825	3.23665807	8.19331121
H	4.74546592	3.50774496	11.66117318
H	6.29105517	5.31302925	8.73044485
O	8.27644200	4.72777814	8.83841337
H	7.30713131	3.37785149	10.87723937
H	6.65517066	4.97913644	11.19148449
O	4.13190256	5.84008599	11.09889625
O	4.72001183	1.74877272	6.35185553
C	4.47506559	1.70109486	7.75980491
H	4.45308770	3.86273731	7.85844281

O	7.17318658	2.05105146	8.61966066
H	8.06058165	6.81455702	8.97222978
C	4.32023168	0.52700503	5.69934828
H	4.54470207	0.65084032	4.63759934
H	3.24332609	0.34739859	5.83120397
H	4.88128405	-0.33008567	6.09930694
H	3.40194672	1.60388829	7.97220126
H	5.01384093	0.86639593	8.22823555
C	8.69115288	6.00671155	9.37053780
H	8.65162354	6.02061547	10.46735456
H	9.72326404	6.15354064	9.04623056
H	6.70148638	3.44519637	7.13521135
H	9.10244807	2.46555881	7.88814339
C	8.32264134	1.69972122	7.81504447
H	8.69261776	0.75252606	8.21438576
H	8.02767274	1.56531240	6.76564411
H	7.33357520	0.14264727	12.59624847
H	4.16494935	5.63233103	12.06168570
H	7.56919507	0.87358423	10.97957838
H	7.76324184	1.86998355	12.45430495
O	5.79494794	1.38028277	11.87454744
C	7.19936442	1.04853319	11.99187044

NMR Spectra

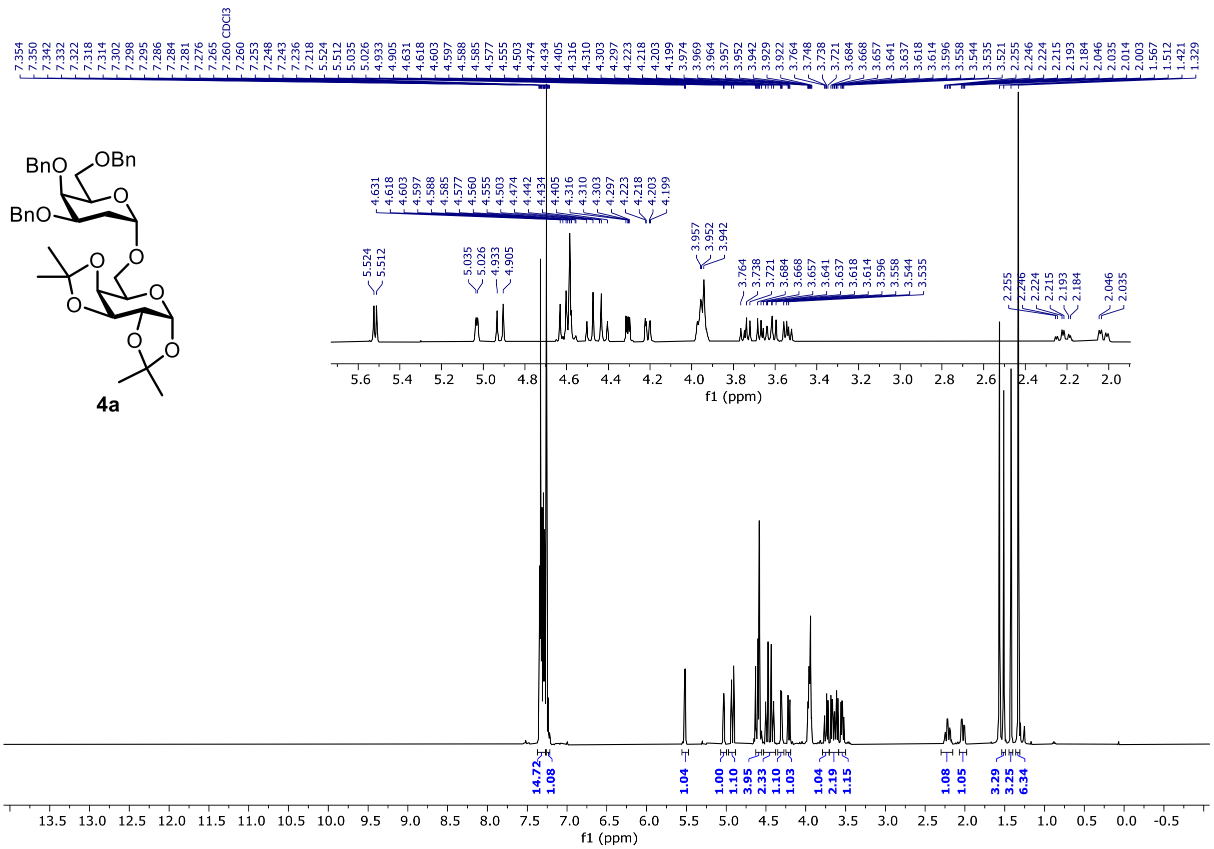

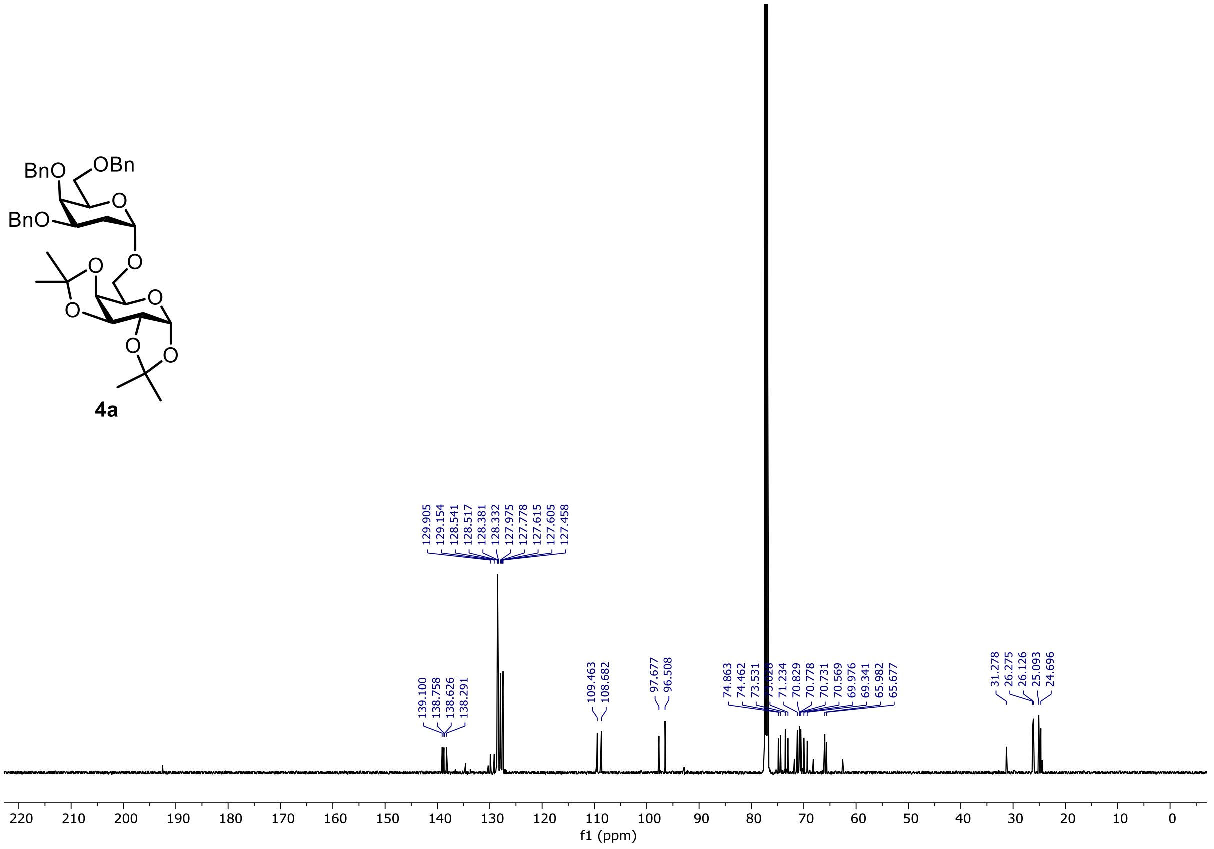

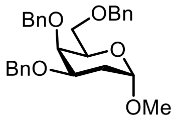

BnO
OBn
O
BnO
OMe

4b

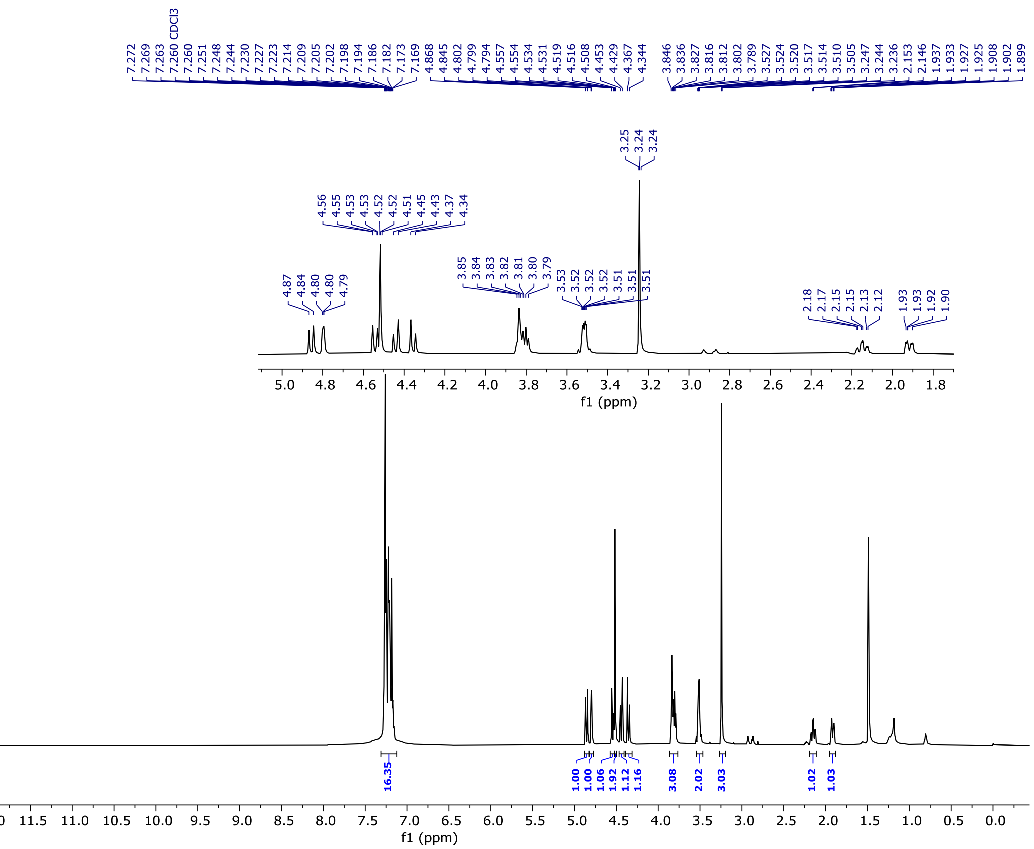

7.272 7.269 7.263 7.260 CDCl3 7.260 7.251 7.248 7.244 7.230 7.227 7.223 7.214 7.209 7.205 7.202 7.198 7.194 7.186 7.182 7.173 7.169 4.868 4.845 4.802 4.799 4.794 4.557 4.554 4.534 4.531 4.519 4.516 4.508 4.453 4.429 4.367 4.344 3.846 3.836 3.827 3.816 3.812 3.802 3.789 3.527 3.524 3.520 3.517 3.514 3.510 3.505 3.247 3.244 3.236 2.153 2.146 1.937 1.933 1.927 1.925 1.908 1.902 1.899
3.25 3.24 3.24
4.56 4.55 4.53 4.53 4.52 4.52 4.51 4.45 4.43 4.37 4.34
4.87 4.84 4.80 4.80 4.79
3.85 3.84 3.83 3.82 3.81 3.80 3.79
3.53 3.52 3.52 3.52 3.51 3.51 3.51
2.18 2.17 2.15 2.15 2.13 2.12
1.93 1.93 1.92 1.90
5.0 4.8 4.6 4.4 4.2 4.0 3.8 3.6 3.4 3.2 3.0 2.8 2.6 2.4 2.2 2.0 1.8
f1 (ppm)
16.35 1.00 1.00 1.06 1.92 1.12 1.16 3.08 2.02 3.03 1.02 1.03
13.5 13.0 12.5 12.0 11.5 11.0 10.5 10.0 9.5 9.0 8.5 8.0 7.5 7.0 6.5 6.0 5.5 5.0 4.5 4.0 3.5 3.0 2.5 2.0 1.5 1.0 0.5 0.0
f1 (ppm)

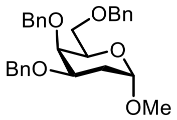

BnO
OBn
O
BnO
OMe

4b

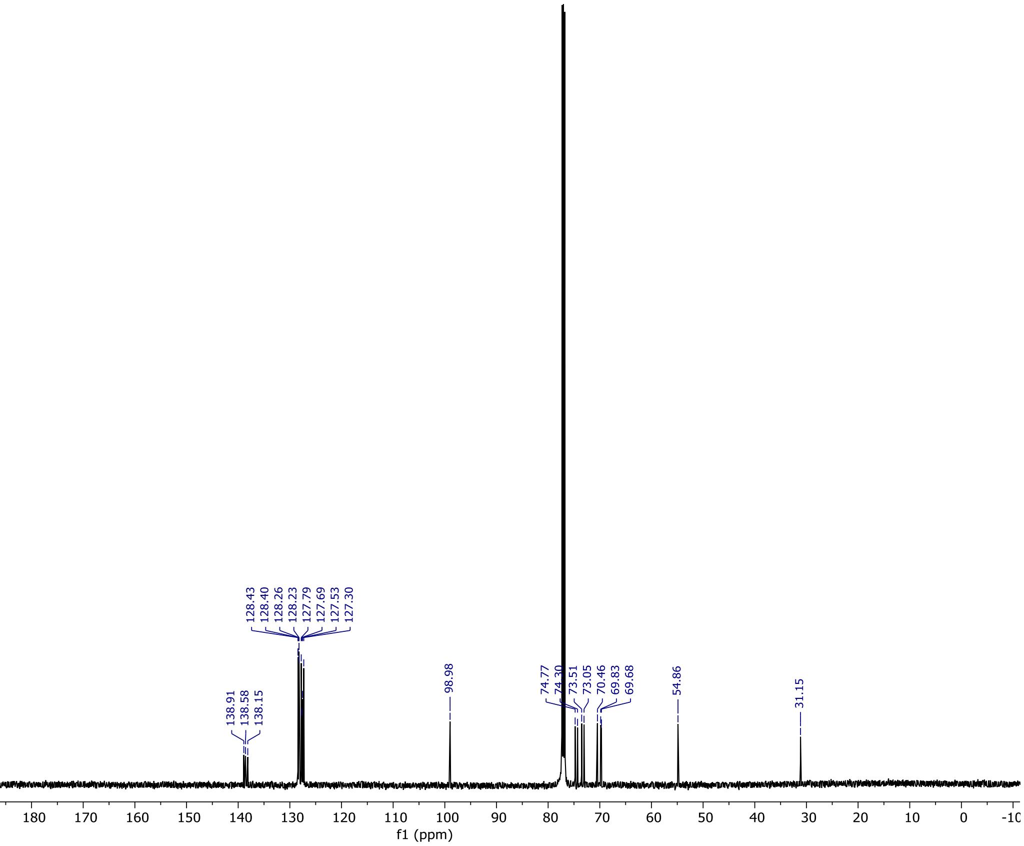

128.43 128.40 128.26 128.23 127.79 127.69 127.53 127.30
138.91 138.58 138.15
98.98
74.77 74.30 73.51 73.05 70.46 69.83 69.68
54.86
31.15
210 200 190 180 170 160 150 140 130 120 110 100 90 80 70 60 50 40 30 20 10 0 -10
f1 (ppm)

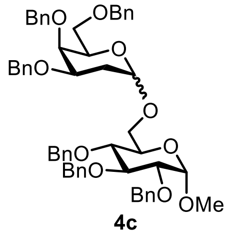

4c

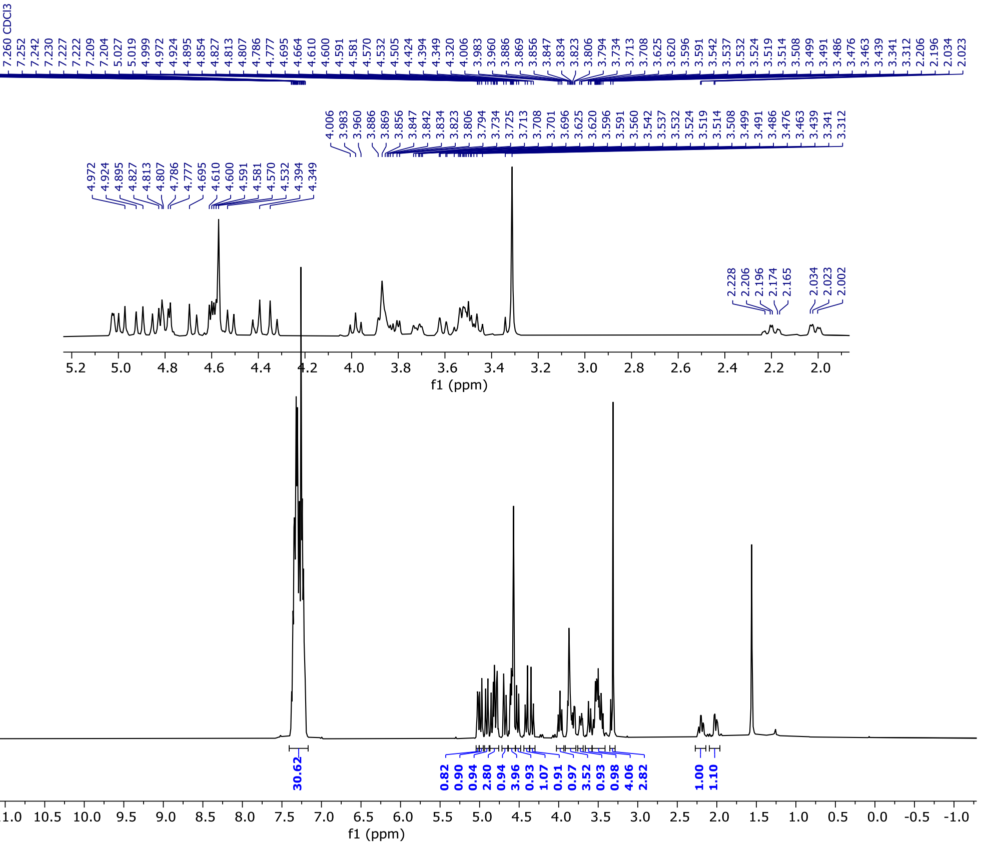
CDCl3
30.62
0.82
0.90
0.94
2.80
0.94
3.96
0.93
1.07
0.91
0.97
3.52
0.93
0.98
4.06
2.82
1.00
1.10
f1 (ppm)

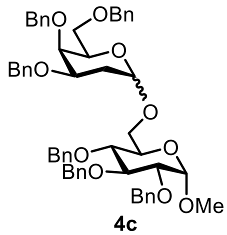

4c

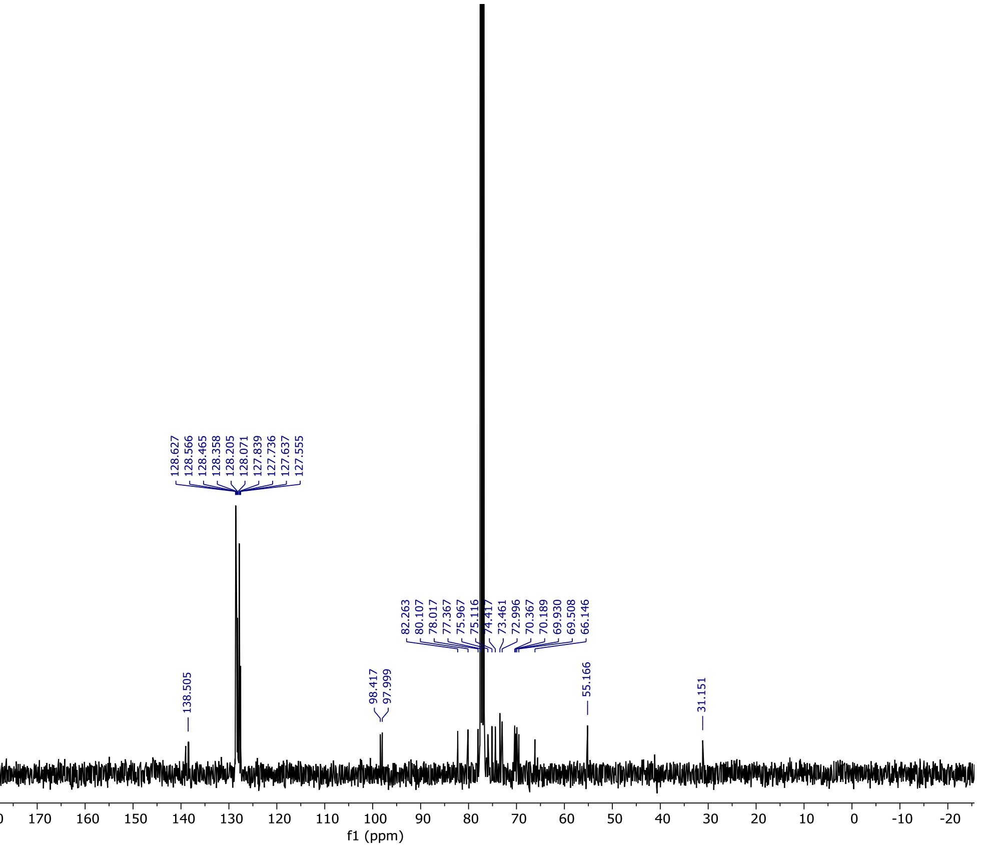
128.627
128.566
128.465
128.358
128.205
128.071
127.839
127.736
127.637
127.555
138.505
98.417
97.999
82.263
80.107
78.017
77.367
75.967
75.116
74.417
73.461
72.996
70.367
70.189
69.930
69.508
66.146
55.166
31.151
f1 (ppm)

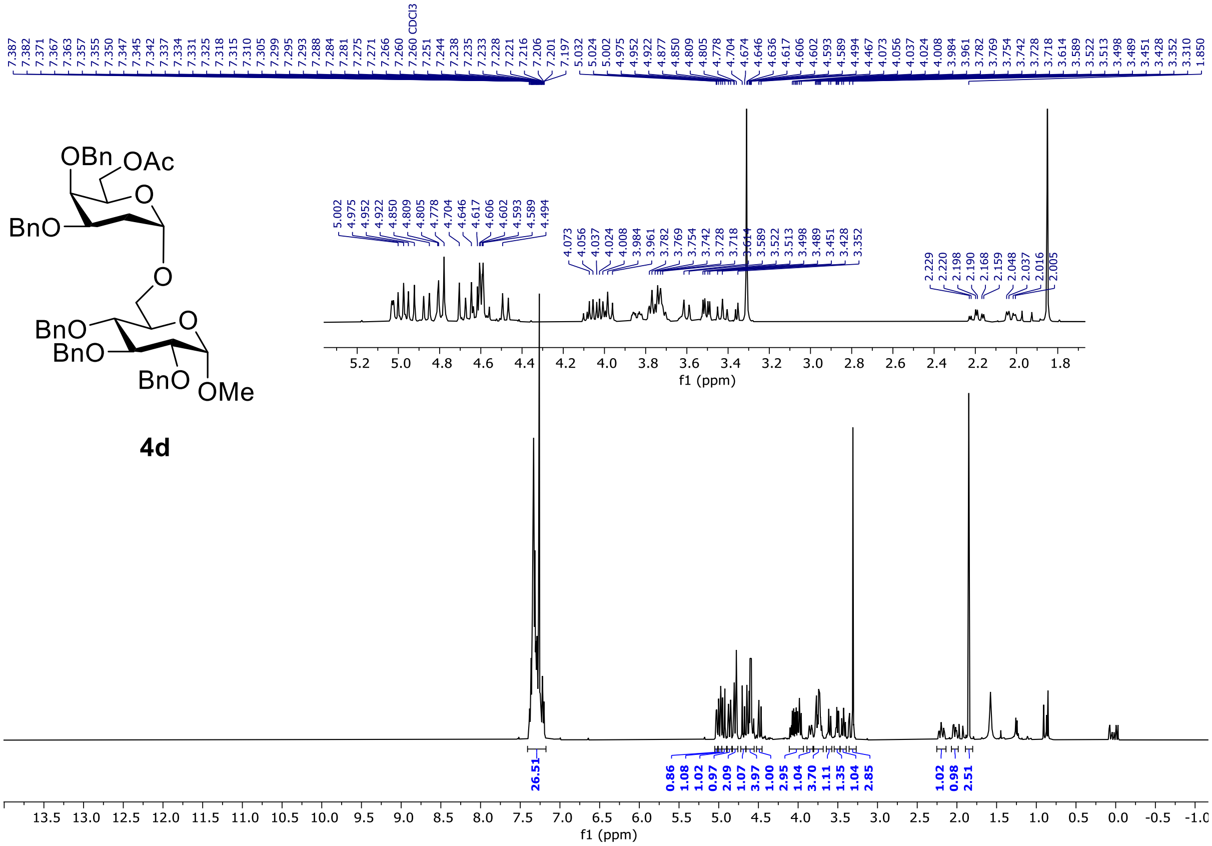

OBn
OAc
O
BnO
O
BnO
O
BnO
BnO
OMe
7.387 7.382 7.371 7.367 7.363 7.357 7.355 7.350 7.347 7.345 7.342 7.337 7.334 7.331 7.325 7.318 7.315 7.310 7.305 7.299 7.295 7.293 7.288 7.284 7.281 7.275 7.271 7.266 7.260 7.260 CDCl3 7.251 7.244 7.238 7.235 7.233 7.228 7.221 7.216 7.206 7.201 7.197 5.032 5.024 5.002 4.975 4.952 4.922 4.877 4.850 4.809 4.805 4.778 4.704 4.674 4.646 4.636 4.617 4.606 4.602 4.593 4.589 4.494 4.467 4.073 4.056 4.037 4.024 4.008 3.984 3.961 3.782 3.769 3.754 3.742 3.728 3.718 3.614 3.589 3.522 3.513 3.498 3.489 3.451 3.428 3.352 3.310 1.850
5.002 4.975 4.952 4.922 4.850 4.809 4.805 4.778 4.704 4.646 4.617 4.606 4.602 4.593 4.589 4.494
4.073 4.056 4.037 4.024 4.008 3.984 3.961 3.782 3.769 3.754 3.742 3.728 3.718 3.614 3.589 3.522 3.513 3.498 3.489 3.451 3.428 3.352
2.229 2.220 2.198 2.190 2.168 2.159 2.048 2.037 2.016 2.005
5.2 5.0 4.8 4.6 4.4 4.2 4.0 3.8 3.6 3.4 3.2 3.0 2.8 2.6 2.4 2.2 2.0 1.8
f1 (ppm)
26.51
0.86 1.08 1.02 0.97 2.09 1.07 3.97 1.00 2.95 1.04 3.70 1.11 1.35 1.04 2.85
1.02 0.98 2.51
13.5 13.0 12.5 12.0 11.5 11.0 10.5 10.0 9.5 9.0 8.5 8.0 7.5 7.0 6.5 6.0 5.5 5.0 4.5 4.0 3.5 3.0 2.5 2.0 1.5 1.0 0.5 0.0 -0.5 -1.0
f1 (ppm)

4d

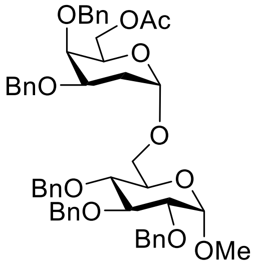

OBn
OAc
O
BnO
O
BnO
O
BnO
BnO
OMe

4d

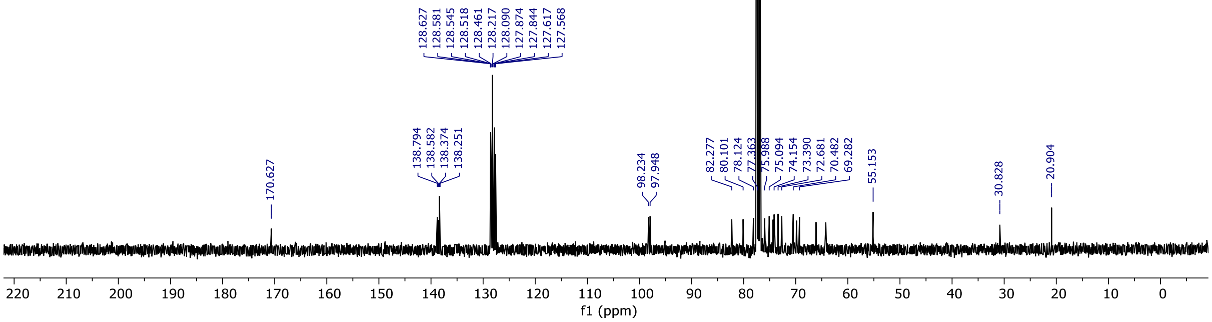

128.627 128.581 128.545 128.518 128.461 128.217 128.090 127.874 127.844 127.617 127.568
170.627
138.794 138.582 138.374 138.251
98.234 97.948
82.277 80.101 78.124 77.363 75.988 75.094 74.154 73.390 72.681 70.482 69.282
55.153
30.828
20.904
220 210 200 190 180 170 160 150 140 130 120 110 100 90 80 70 60 50 40 30 20 10 0
f1 (ppm)

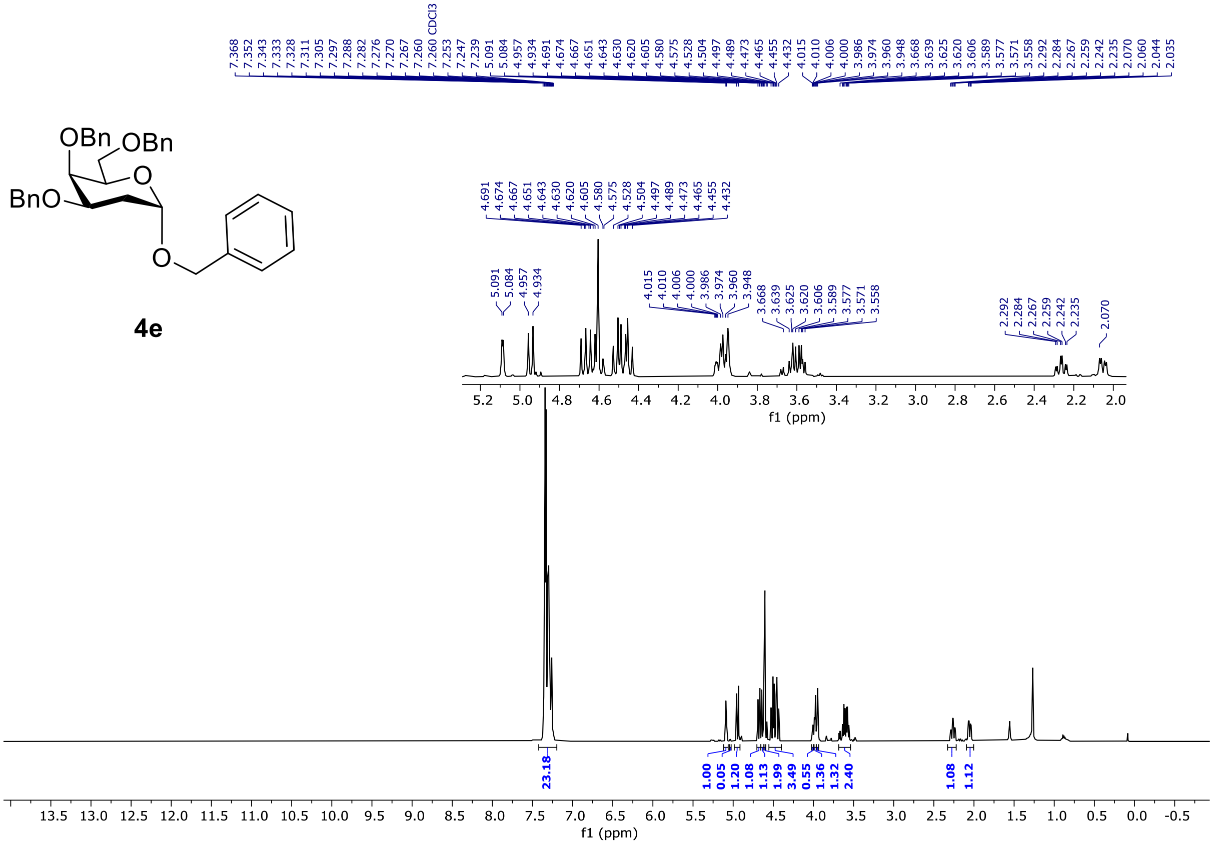

4e

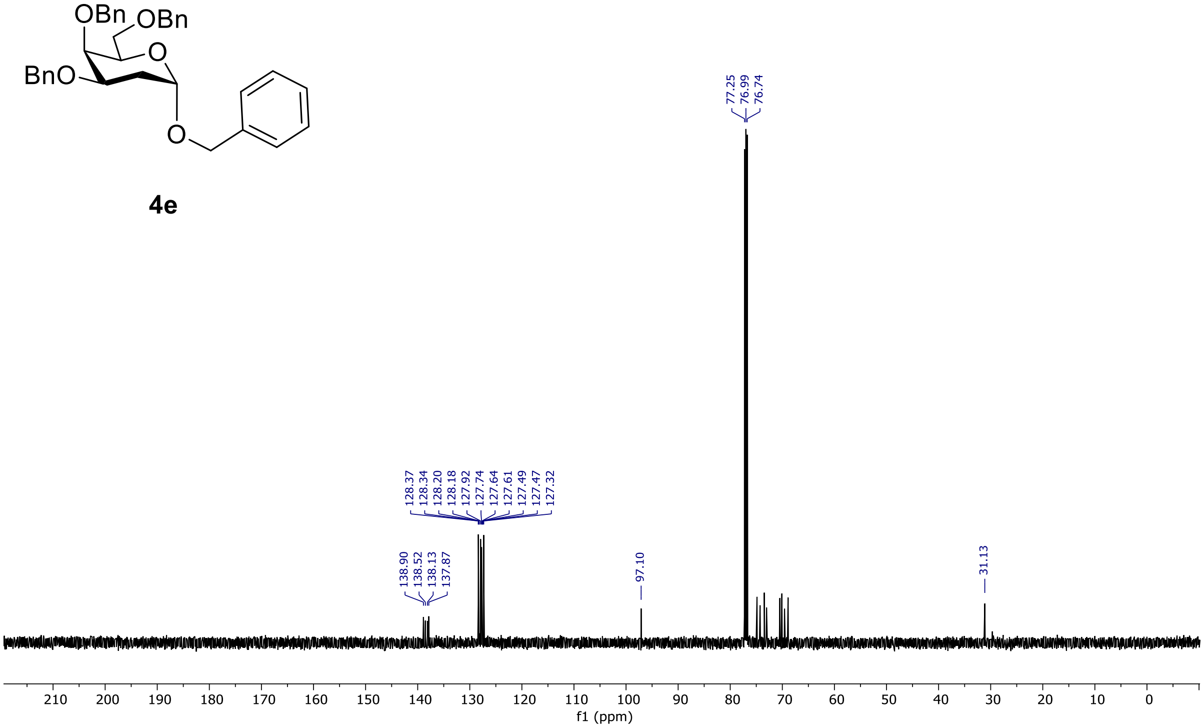

4e

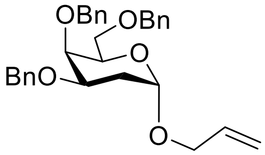

OBn
OBn
O
BnO
O

4f

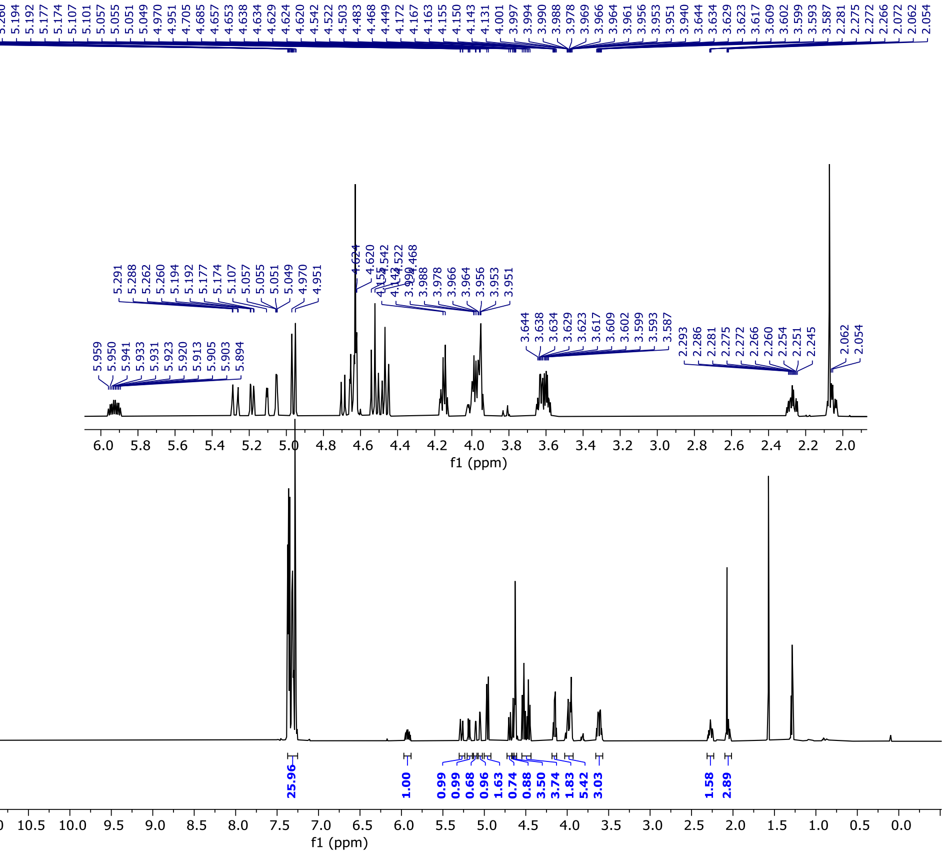

7.372 7.368 7.365 7.361 7.357 7.347 7.334 7.326 7.322 7.314 7.311 7.308 7.301 7.285 7.270 5.291 5.288 5.262 5.260 5.194 5.192 5.177 5.174 5.107 5.101 5.057 5.055 5.051 5.049 4.970 4.951 4.705 4.685 4.657 4.653 4.638 4.634 4.629 4.624 4.620 4.542 4.522 4.503 4.483 4.468 4.449 4.172 4.167 4.163 4.155 4.150 4.143 4.131 4.001 3.997 3.994 3.990 3.988 3.978 3.969 3.966 3.964 3.961 3.956 3.953 3.951 3.940 3.644 3.634 3.629 3.623 3.617 3.609 3.602 3.599 3.593 3.587 2.281 2.275 2.272 2.266 2.072 2.062 2.054
25.96
1.00
0.99
0.99
0.68
0.96
1.63
0.74
0.88
3.50
3.74
5.42
1.83
3.03
1.58
2.89
13.5 13.0 12.5 12.0 11.5 11.0 10.5 10.0 9.5 9.0 8.5 8.0 7.5 7.0 6.5 6.0 5.5 5.0 4.5 4.0 3.5 3.0 2.5 2.0 1.5 1.0 0.5 0.0
f1 (ppm)
6.0 5.8 5.6 5.4 5.2 5.0 4.8 4.6 4.4 4.2 4.0 3.8 3.6 3.4 3.2 3.0 2.8 2.6 2.4 2.2 2.0
f1 (ppm)

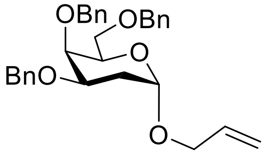

OBn
OBn
O
BnO
O

4f

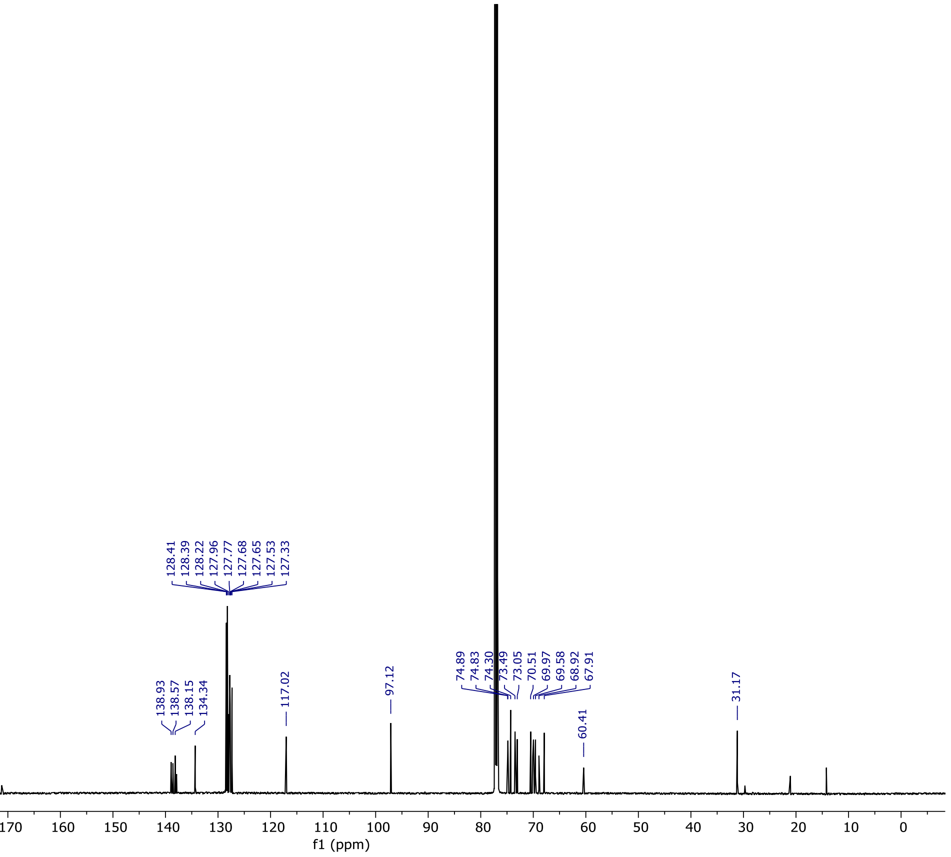

128.41 128.39 128.22 127.96 127.77 127.68 127.65 127.53 127.33
138.93 138.57 138.15 134.34
117.02
97.12
74.89 74.83 74.30 73.49 73.05 70.51 69.97 69.58 68.92 67.91
60.41
31.17
220 210 200 190 180 170 160 150 140 130 120 110 100 90 80 70 60 50 40 30 20 10 0
f1 (ppm)

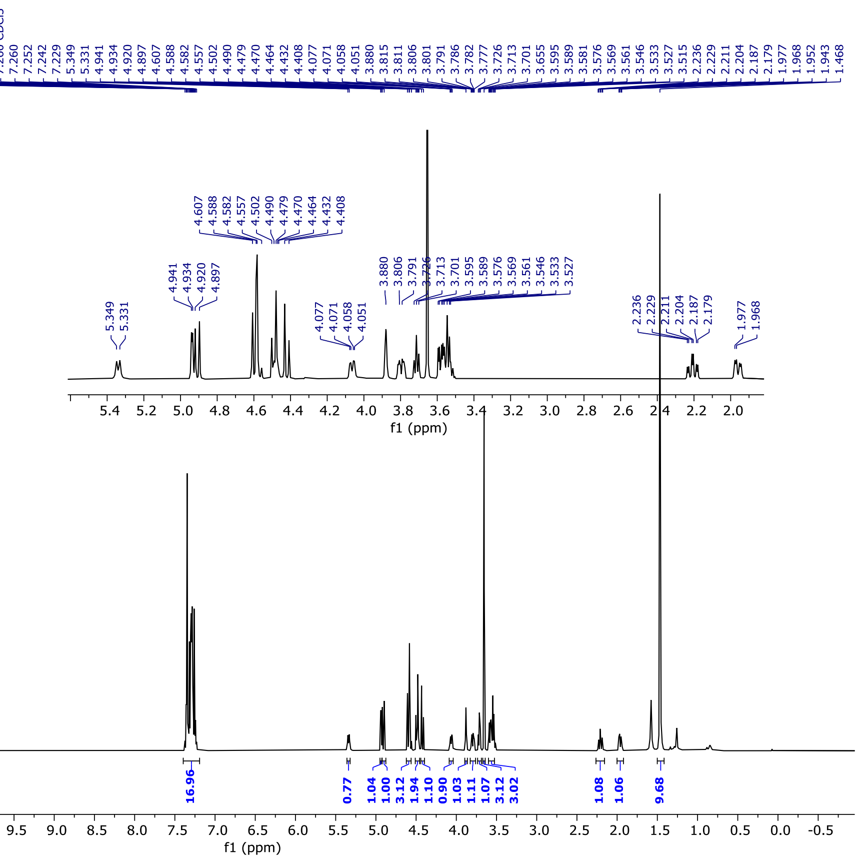

7.374 7.358 7.353 7.347 7.333 7.319 7.309 7.305 7.302 7.298 7.292 7.284 7.277 7.273 7.269 7.260 CDCl3 7.260 7.252 7.242 7.229 5.349 5.331 4.941 4.934 4.920 4.897 4.607 4.588 4.582 4.557 4.502 4.490 4.479 4.470 4.464 4.432 4.408 4.077 4.071 4.058 4.051 3.880 3.815 3.811 3.806 3.801 3.791 3.786 3.782 3.777 3.726 3.713 3.701 3.655 3.595 3.589 3.581 3.576 3.569 3.561 3.546 3.533 3.527 3.515 2.236 2.229 2.211 2.204 2.187 2.179 1.977 1.968 1.952 1.943 1.468
16.96 0.77 1.04 1.00 3.12 1.94 1.10 0.90 1.03 1.11 1.07 3.12 3.02 1.08 1.06 9.68
f1 (ppm)

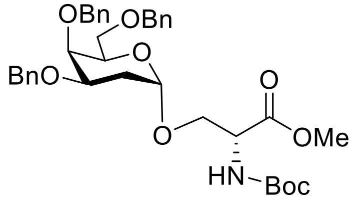

OBn
OBn
O
BnO
O
O
OMe
HN
Boc

4g

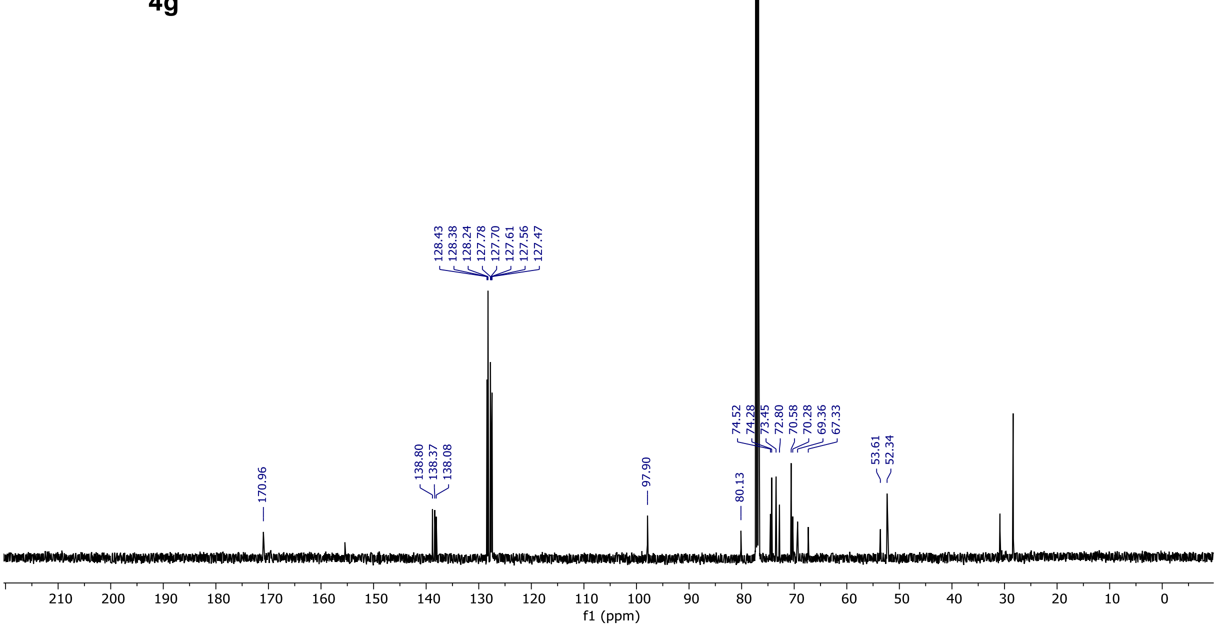

170.96
138.80 138.37 138.08
128.43 128.38 128.24 127.78 127.70 127.61 127.56 127.47
97.90
80.13
74.52 74.28 73.45 72.80 70.58 70.28 69.36 67.33
53.61 52.34
f1 (ppm)

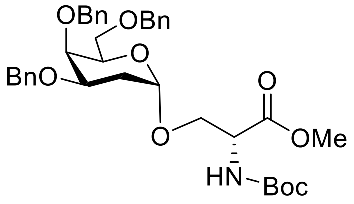

OBn
OBn
O
BnO
O
O
OMe
HN
Boc

4g

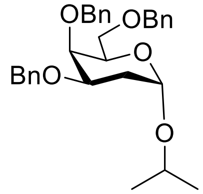

4l

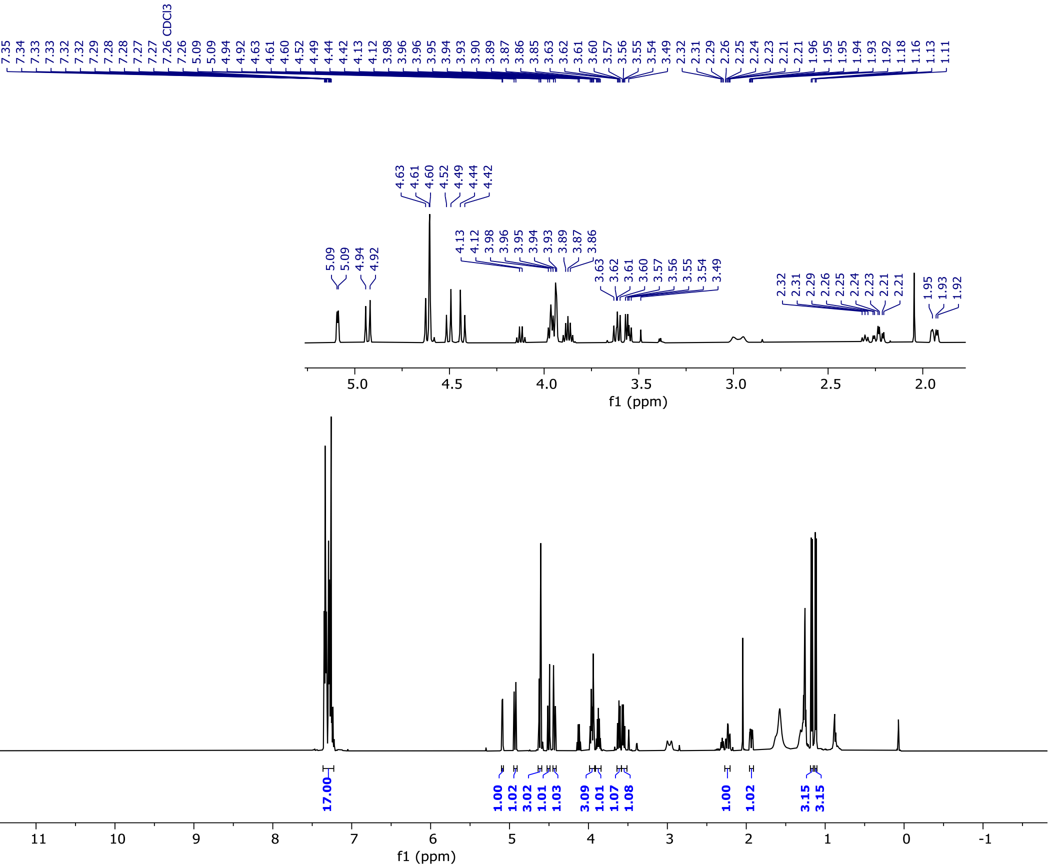

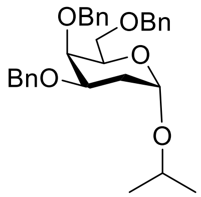
OBn
OBn
O
BnO
O

4l

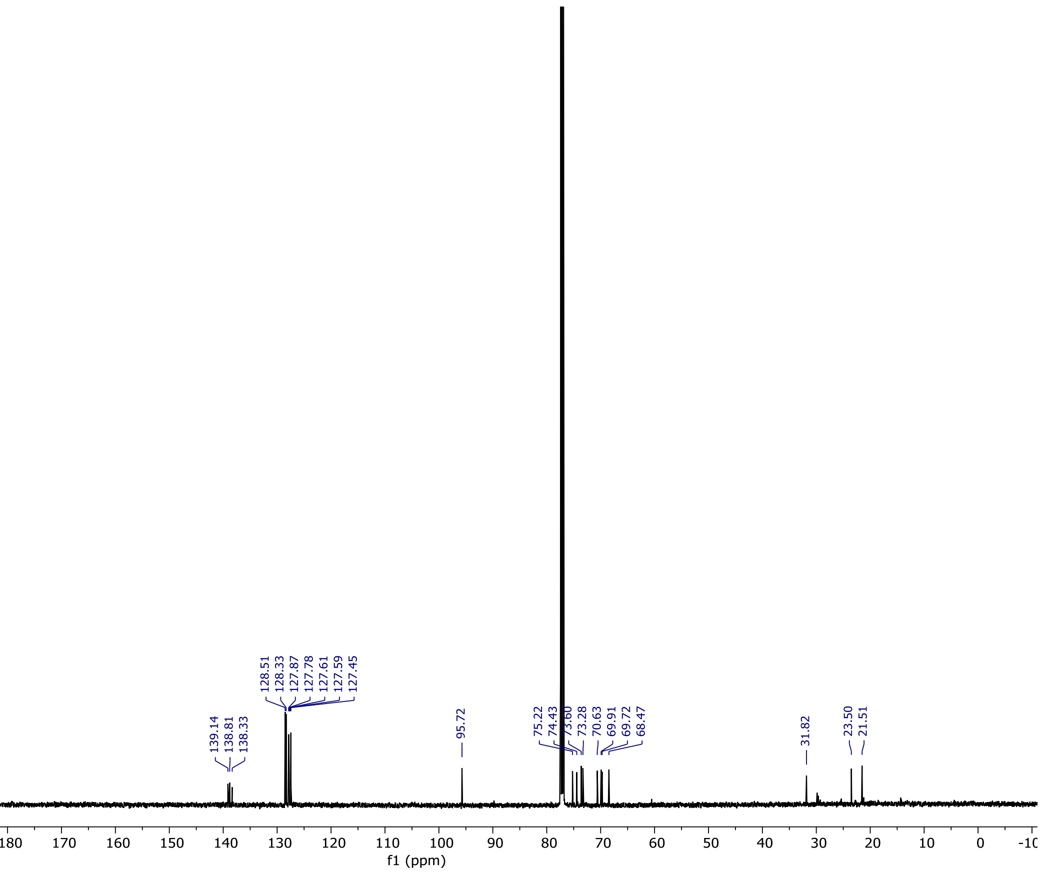
139.14
138.81
138.33
128.51
128.33
127.87
127.78
127.61
127.59
127.45
95.72
75.22
74.43
73.60
73.28
70.63
69.91
69.72
68.47
31.82
23.50
21.51
210 200 190 180 170 160 150 140 130 120 110 100 90 80 70 60 50 40 30 20 10 0
f1 (ppm)

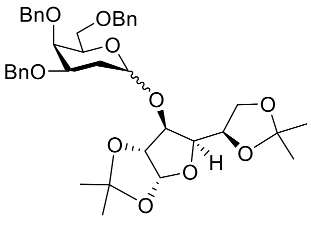

4k

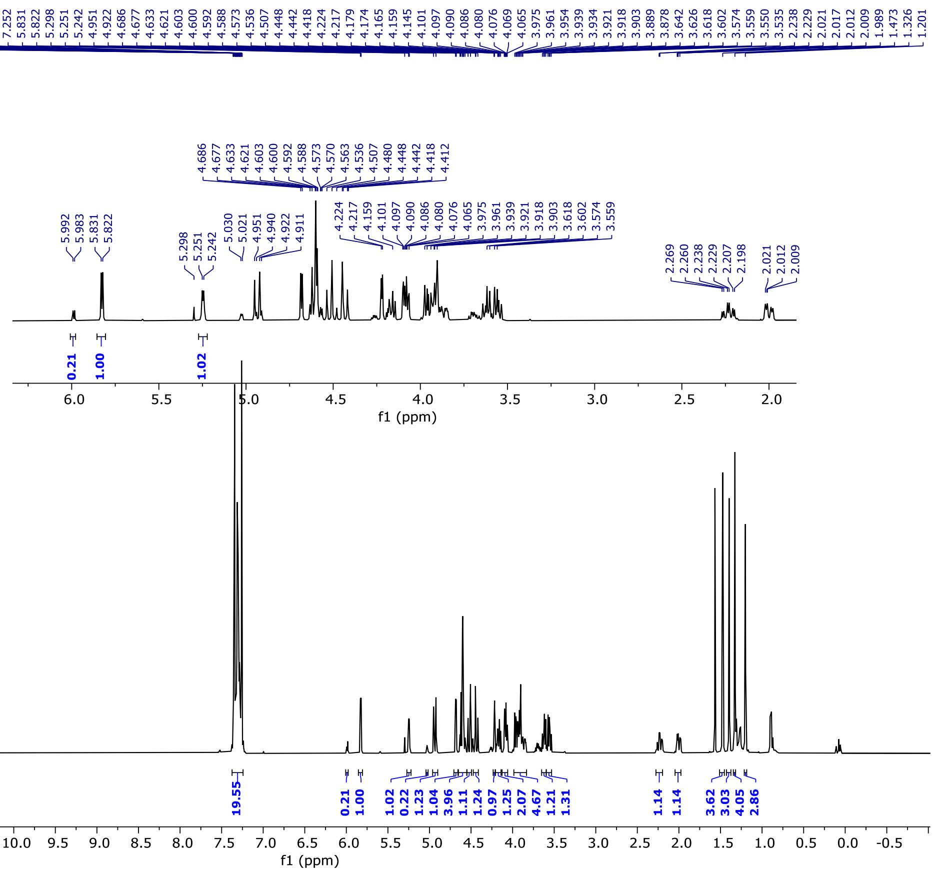
7.362 7.357 7.353 7.344 7.335 7.332 7.330 7.325 7.320 7.315 7.312 7.306 7.301 7.296 7.287 7.285 7.283 7.279 7.275 7.269 7.260 7.255 7.252 5.831 5.822 5.298 5.251 5.242 4.951 4.922 4.686 4.677 4.633 4.621 4.603 4.600 4.592 4.588 4.573 4.536 4.507 4.448 4.442 4.418 4.224 4.217 4.179 4.174 4.165 4.159 4.145 4.101 4.097 4.090 4.086 4.080 4.076 4.069 4.065 3.975 3.961 3.954 3.939 3.934 3.921 3.918 3.903 3.889 3.878 3.642 3.626 3.618 3.602 3.574 3.559 3.550 3.535 2.238 2.229 2.021 2.017 2.012 2.009 1.989 1.473 1.326 1.201
f1 (ppm)

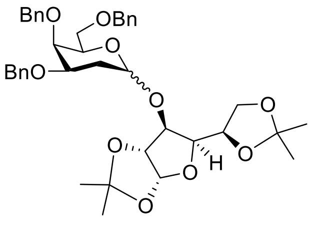

4k

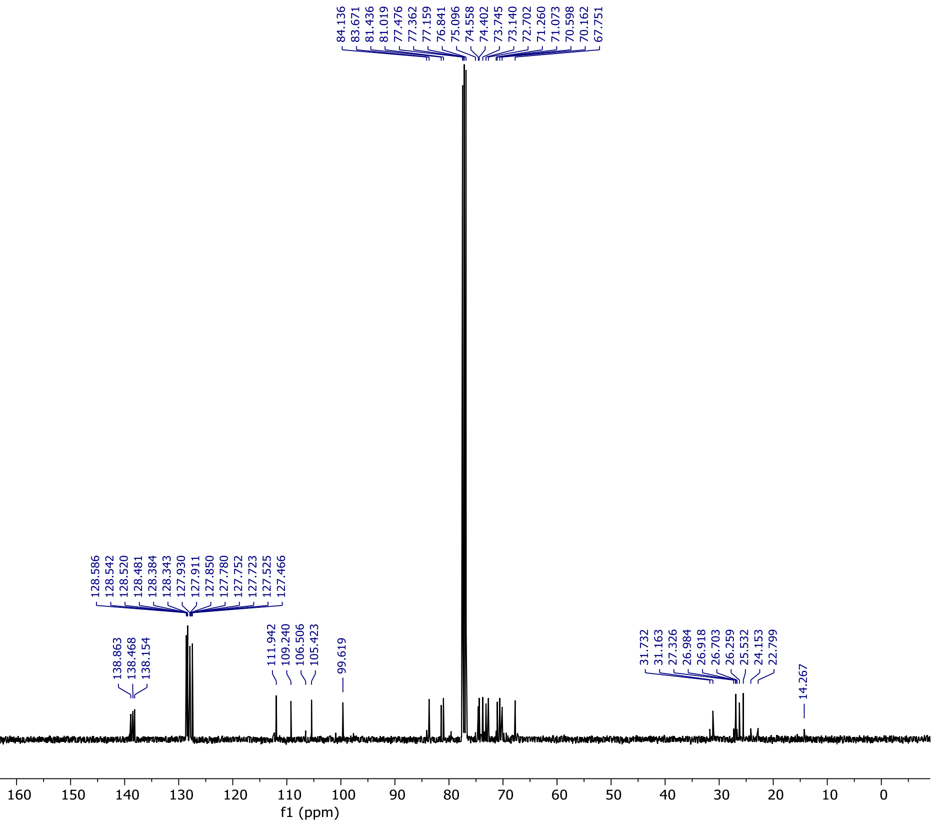
84.136 83.671 81.436 81.019 77.476 77.362 77.159 76.841 75.096 74.558 74.402 73.745 73.140 72.702 71.260 71.073 70.598 70.162 67.751
128.586 128.542 128.520 128.481 128.384 128.343 127.930 127.911 127.850 127.780 127.752 127.723 127.525 127.466
138.863 138.468 138.154
111.942 109.240 106.506 105.423
99.619
31.732 31.163 27.326 26.984 26.918 26.703 26.259 25.532 24.153 22.799
14.267
f1 (ppm)

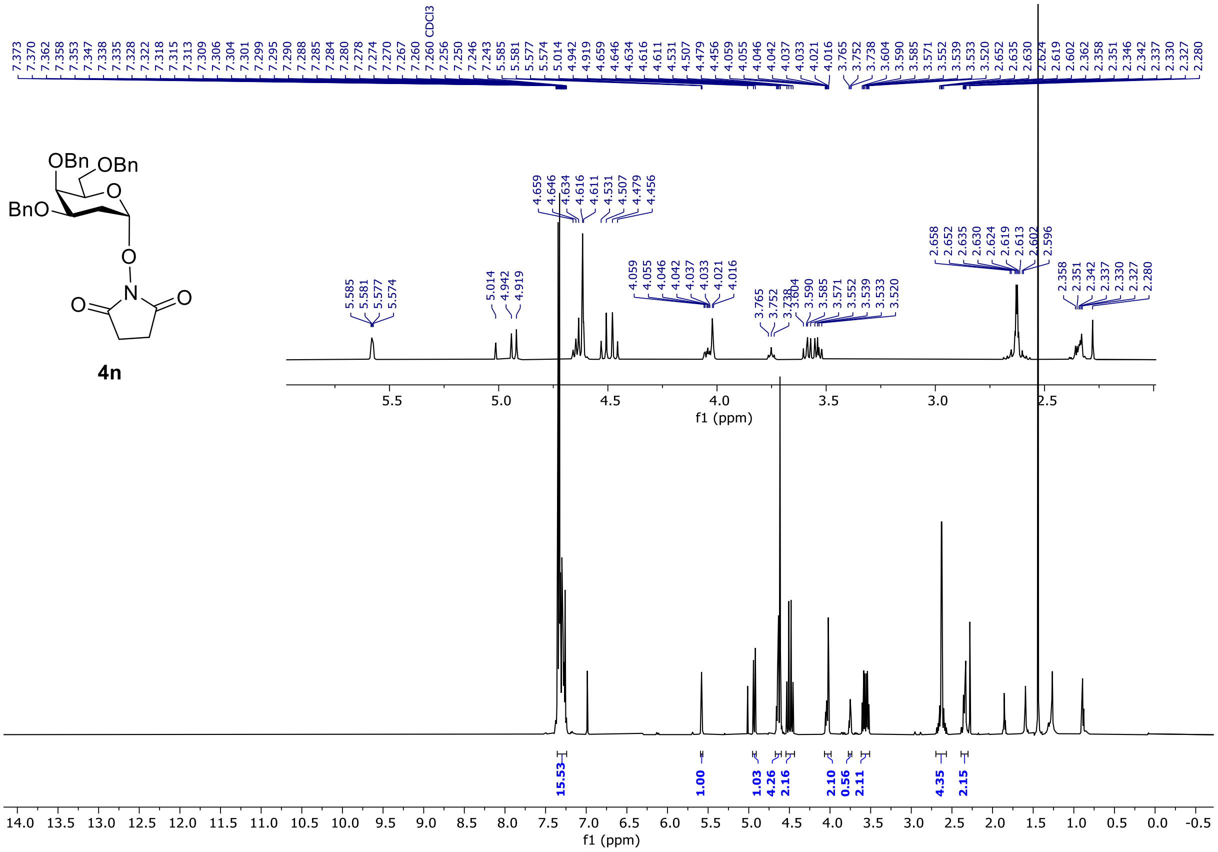

4n

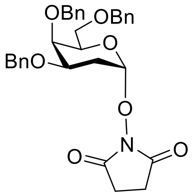
OBn
OBn
BnO
O
O
N
O
O

4n

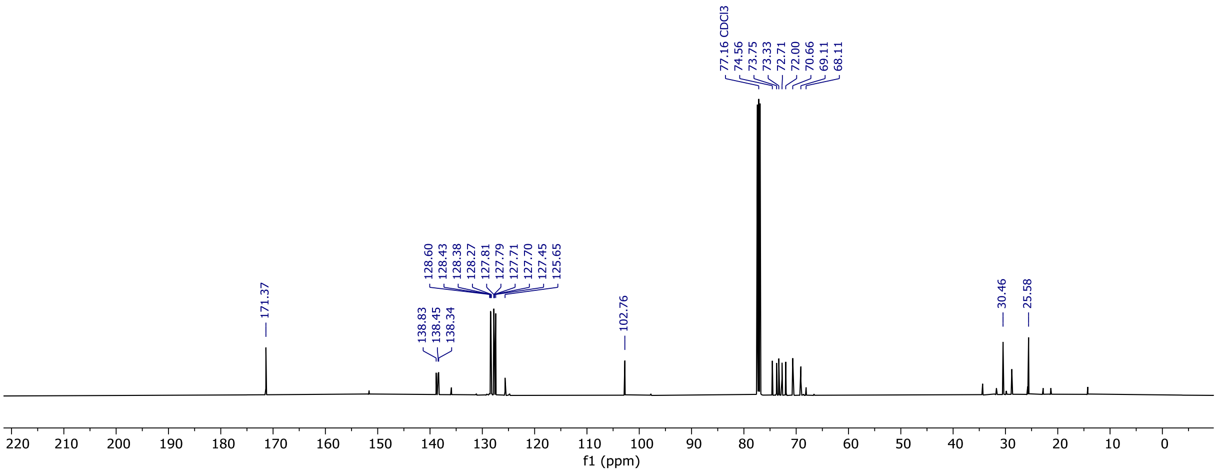
77.16 CDCl3
74.56
73.75
73.33
72.71
72.00
70.66
69.11
68.11
128.60
128.43
128.38
128.27
127.81
127.79
127.71
127.70
127.45
125.65
171.37
138.83
138.45
138.34
102.76
30.46
25.58
220 210 200 190 180 170 160 150 140 130 120 110 100 90 80 70 60 50 40 30 20 10 0
f1 (ppm)

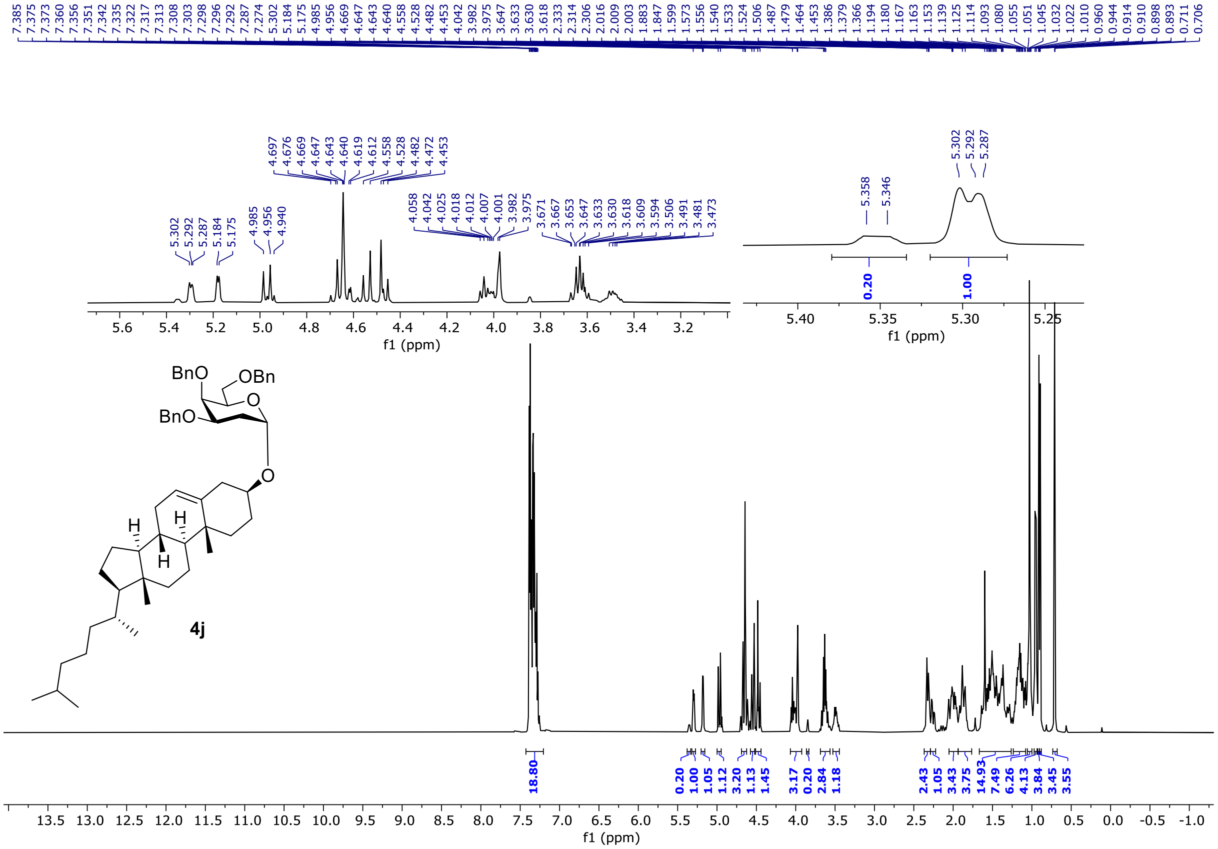
7.385 7.375 7.373 7.360 7.356 7.351 7.342 7.335 7.322 7.317 7.313 7.308 7.303 7.298 7.296 7.292 7.287 7.274 5.302 5.184 5.175 4.985 4.956 4.669 4.647 4.643 4.640 4.558 4.528 4.482 4.453 4.042 3.982 3.975 3.647 3.633 3.630 3.618 2.333 2.314 2.306 2.016 2.009 2.003 1.883 1.847 1.599 1.573 1.556 1.540 1.533 1.524 1.506 1.487 1.479 1.464 1.453 1.386 1.379 1.366 1.194 1.180 1.167 1.163 1.153 1.139 1.125 1.114 1.093 1.080 1.055 1.051 1.045 1.032 1.022 1.010 0.960 0.944 0.914 0.910 0.898 0.893 0.711 0.706
5.302 5.292 5.287 5.184 5.175
4.985 4.956 4.940
4.697 4.676 4.669 4.647 4.643 4.640 4.619 4.612 4.558 4.528 4.482 4.472 4.453
4.058 4.042 4.025 4.018 4.012 4.007 4.001 3.982 3.975
3.671 3.667 3.653 3.647 3.633 3.630 3.618 3.609 3.594 3.506 3.491 3.481 3.473
5.6 5.4 5.2 5.0 4.8 4.6 4.4 4.2 4.0 3.8 3.6 3.4 3.2
f1 (ppm)
5.358 5.346
5.302 5.292 5.287
0.20
1.00
5.40 5.35 5.30 5.25
f1 (ppm)
BnO
OBn
BnO
O
O
H
H
H
4j
18.80
0.20 1.00 1.05 1.12 3.20 1.13 1.45 3.17 0.20 2.84 1.18 2.43 1.05 3.43 3.75 14.93 7.49 6.26 4.13 3.84 3.45 3.55
13.5 13.0 12.5 12.0 11.5 11.0 10.5 10.0 9.5 9.0 8.5 8.0 7.5 7.0 6.5 6.0 5.5 5.0 4.5 4.0 3.5 3.0 2.5 2.0 1.5 1.0 0.5 0.0 -0.5 -1.0
f1 (ppm)

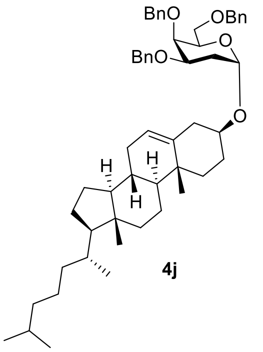
BnO
OBn
BnO
O
O
H
H
H

4j

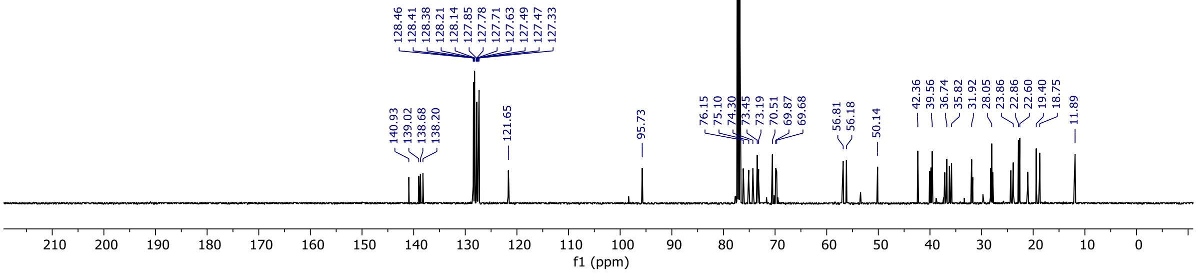
128.46
128.41
128.38
128.21
128.14
127.85
127.78
127.71
127.63
127.49
127.47
127.33
140.93
139.02
138.68
138.20
121.65
95.73
76.15
75.10
74.30
73.45
73.19
70.51
69.87
69.68
56.81
56.18
50.14
42.36
39.56
36.74
35.82
31.92
28.05
23.86
22.86
22.60
19.40
18.75
11.89
f1 (ppm)

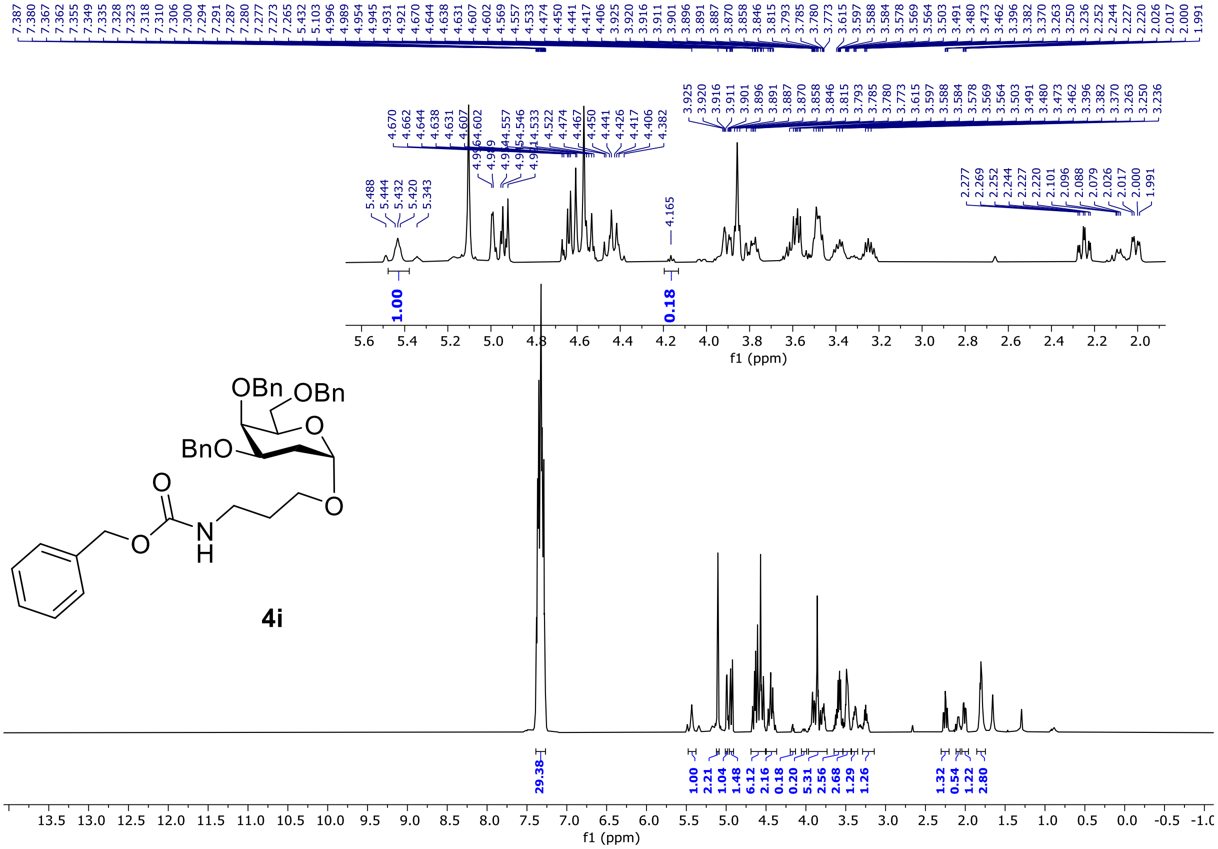
1.00
0.18
f1 (ppm)
29.38
1.00
2.21
1.04
1.48
6.12
2.16
0.18
0.20
5.31
2.56
2.68
1.29
1.26
1.32
0.54
1.22
2.80
f1 (ppm)
OBn
OBn
BnO
O
O
O
O
N
H

4i

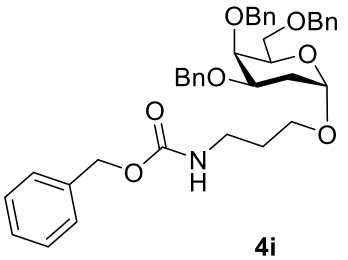
OBn
OBn
O
BnO
O
O
N
H
O

4i

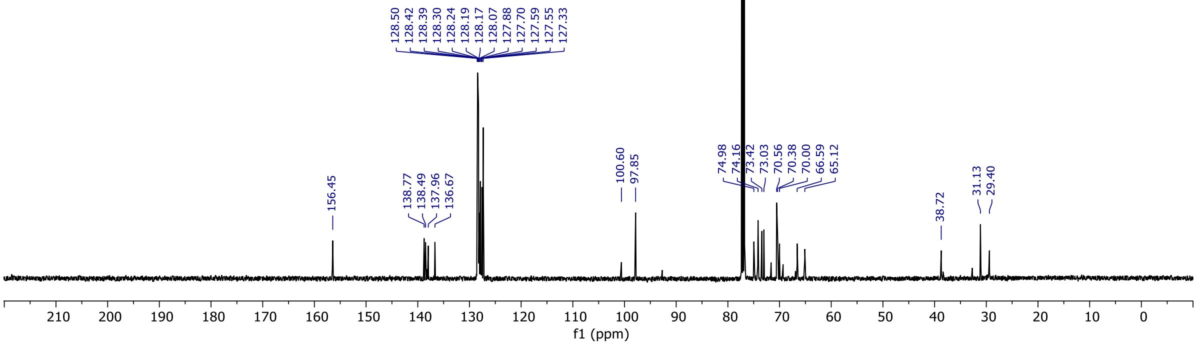
128.50
128.42
128.39
128.30
128.24
128.19
128.17
128.07
127.88
127.70
127.59
127.55
127.33
156.45
138.77
138.49
137.96
136.67
100.60
97.85
74.98
74.16
73.42
73.03
70.56
70.38
70.00
66.59
65.12
38.72
31.13
29.40
f1 (ppm)

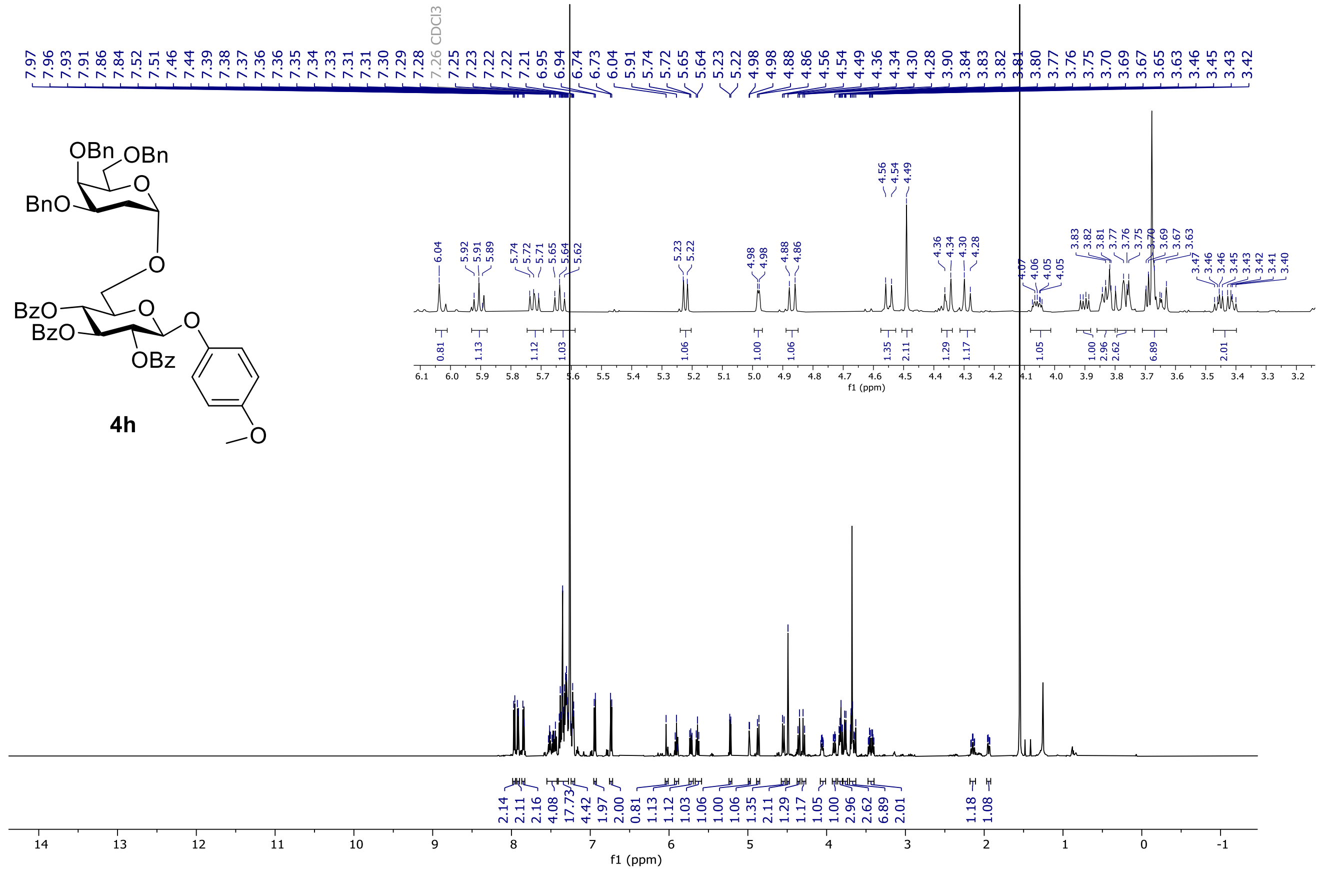
OBn
OBn
O
BnO
O
BzO
BzO
O
OBz
O
O
4h
7.97
7.96
7.93
7.91
7.86
7.84
7.52
7.51
7.46
7.44
7.39
7.38
7.37
7.36
7.36
7.35
7.34
7.33
7.31
7.31
7.30
7.29
7.28
7.26 CDCl3
7.25
7.23
7.22
7.22
7.21
6.95
6.94
6.74
6.73
6.04
5.91
5.74
5.72
5.65
5.64
5.23
5.22
4.98
4.98
4.88
4.86
4.56
4.54
4.49
4.36
4.34
4.30
4.28
3.90
3.84
3.83
3.82
3.81
3.80
3.77
3.76
3.75
3.70
3.69
3.67
3.65
3.63
3.46
3.45
3.43
3.42
f1 (ppm)
2.14
2.11
2.16
4.08
17.73
4.42
1.97
2.00
0.81
1.13
1.12
1.03
1.06
1.00
1.06
1.35
2.11
1.29
1.17
1.05
1.00
2.96
2.62
6.89
2.01
1.18
1.08

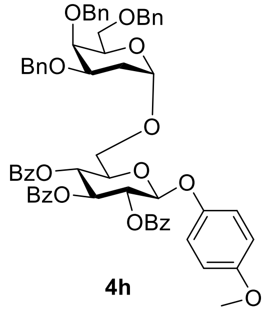
OBn
OBn
O
BnO
O
BzO
O
O
BzO
OBz
O
4h

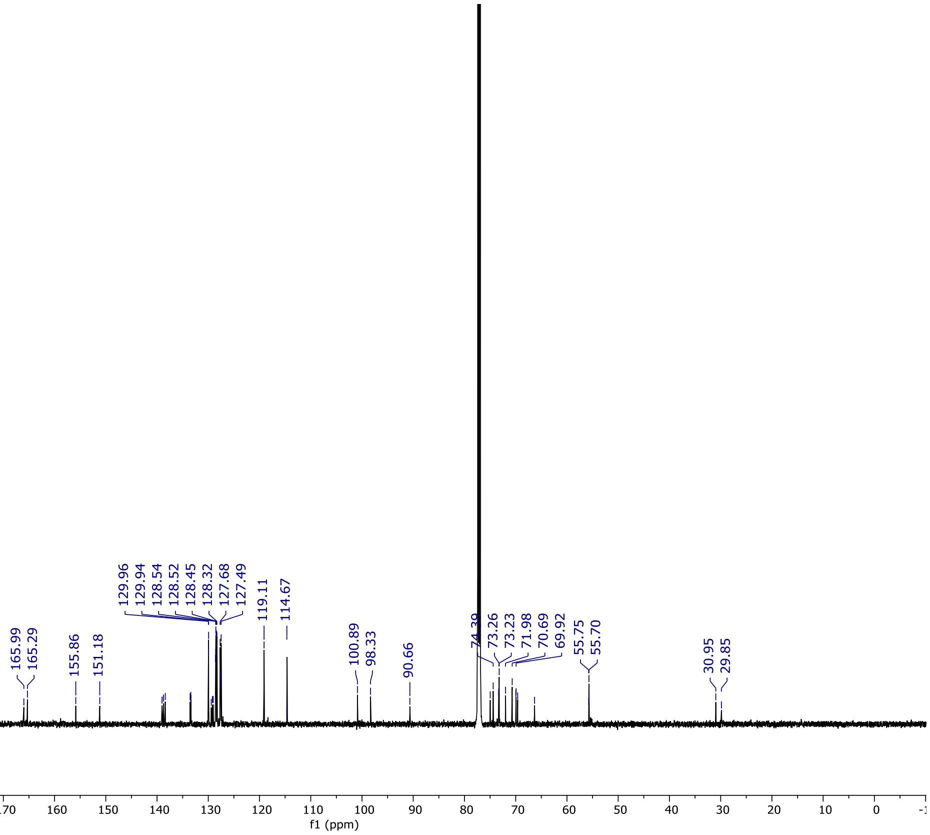
165.99
165.29
155.86
151.18
129.96
129.94
128.54
128.52
128.45
128.32
127.68
127.49
119.11
114.67
100.89
98.33
90.66
74.39
73.26
73.23
71.98
70.69
69.92
55.75
55.70
30.95
29.85
220 210 200 190 180 170 160 150 140 130 120 110 100 90 80 70 60 50 40 30 20 10 0
f1 (ppm)

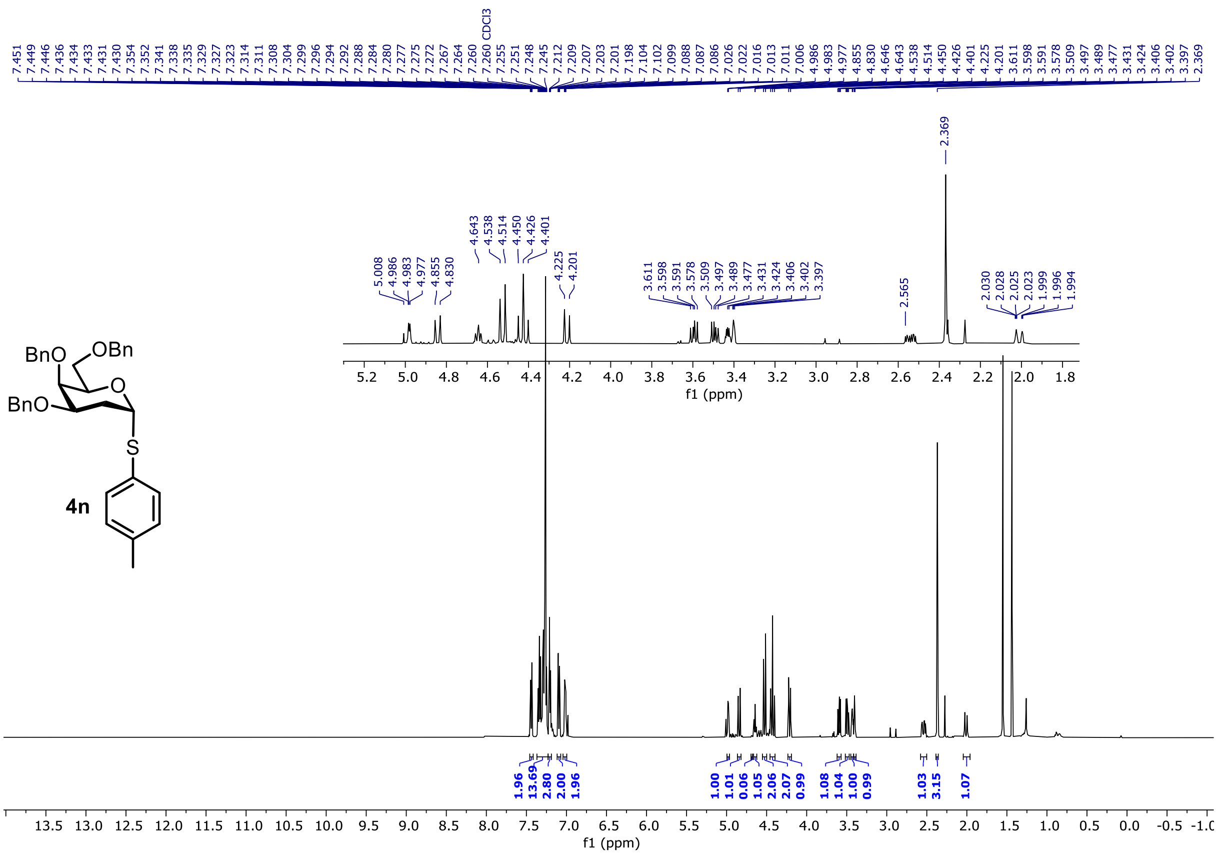
7.451 7.449 7.446 7.436 7.434 7.433 7.431 7.430 7.354 7.352 7.341 7.338 7.335 7.329 7.327 7.323 7.314 7.311 7.308 7.304 7.299 7.296 7.294 7.292 7.288 7.284 7.280 7.277 7.275 7.272 7.267 7.264 7.260 CDCl3 7.260 7.255 7.251 7.248 7.245 7.212 7.209 7.207 7.203 7.201 7.198 7.104 7.102 7.099 7.088 7.087 7.086 7.026 7.022 7.016 7.013 7.011 7.006 4.986 4.983 4.977 4.855 4.830 4.646 4.643 4.538 4.514 4.450 4.426 4.401 4.225 4.201 3.611 3.598 3.591 3.578 3.509 3.497 3.489 3.477 3.431 3.424 3.406 3.402 3.397 2.369
2.369
5.008 4.986 4.983 4.977 4.855 4.830
4.643 4.538 4.514 4.450 4.426 4.401
4.225 4.201
3.611 3.598 3.591 3.578 3.509 3.497 3.489 3.477 3.431 3.424 3.406 3.402 3.397
2.565
2.030 2.028 2.025 2.023 1.999 1.996 1.994
f1 (ppm)
BnO OBn
BnO
O
S
4n
1.96 13.69 2.80 2.00 1.96
1.00 1.01 0.06 1.05 2.06 2.07 0.99
1.08 1.04 1.00 0.99
1.03 3.15
1.07
f1 (ppm)

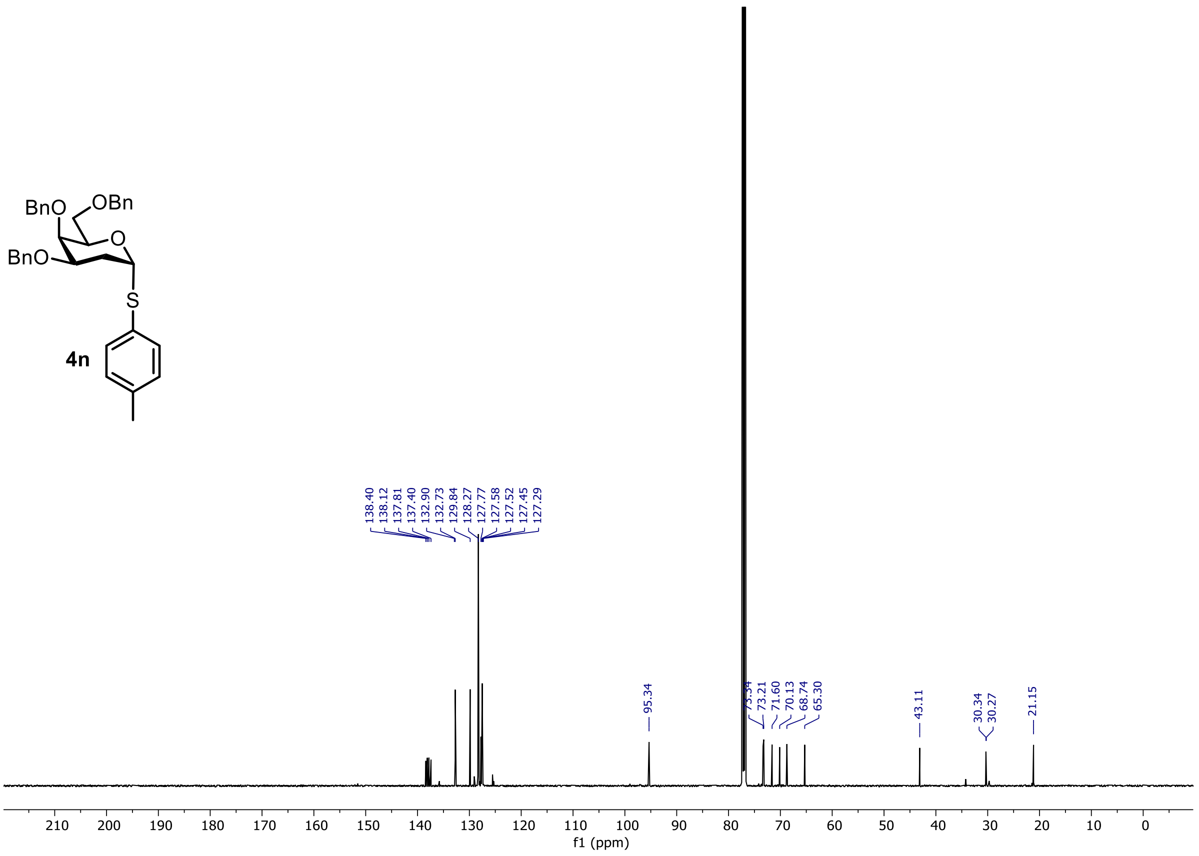
BnO OBn
BnO
O
S
4n
138.40 138.12 137.81 137.40 132.90 132.73 129.84 128.27 127.77 127.58 127.52 127.45 127.29
95.34
73.34 73.21 71.60 70.13 68.74 65.30
43.11
30.34 30.27
21.15
f1 (ppm)

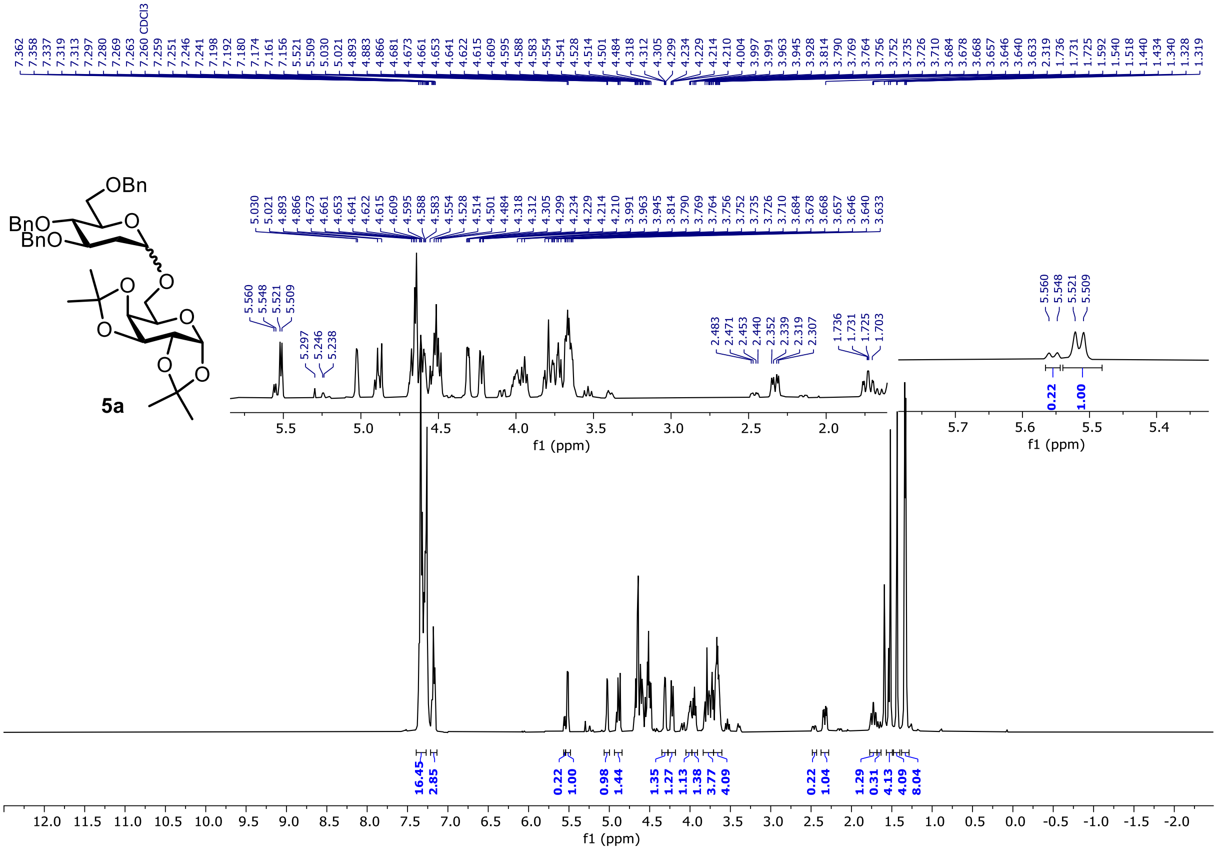
OBn
BnO
BnO
O
5a
CDCl3
f1 (ppm)
f1 (ppm)
f1 (ppm)

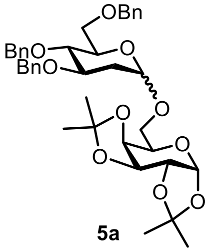
OBn
BnO
BnO
O
O
O
O
O
O
O

5a

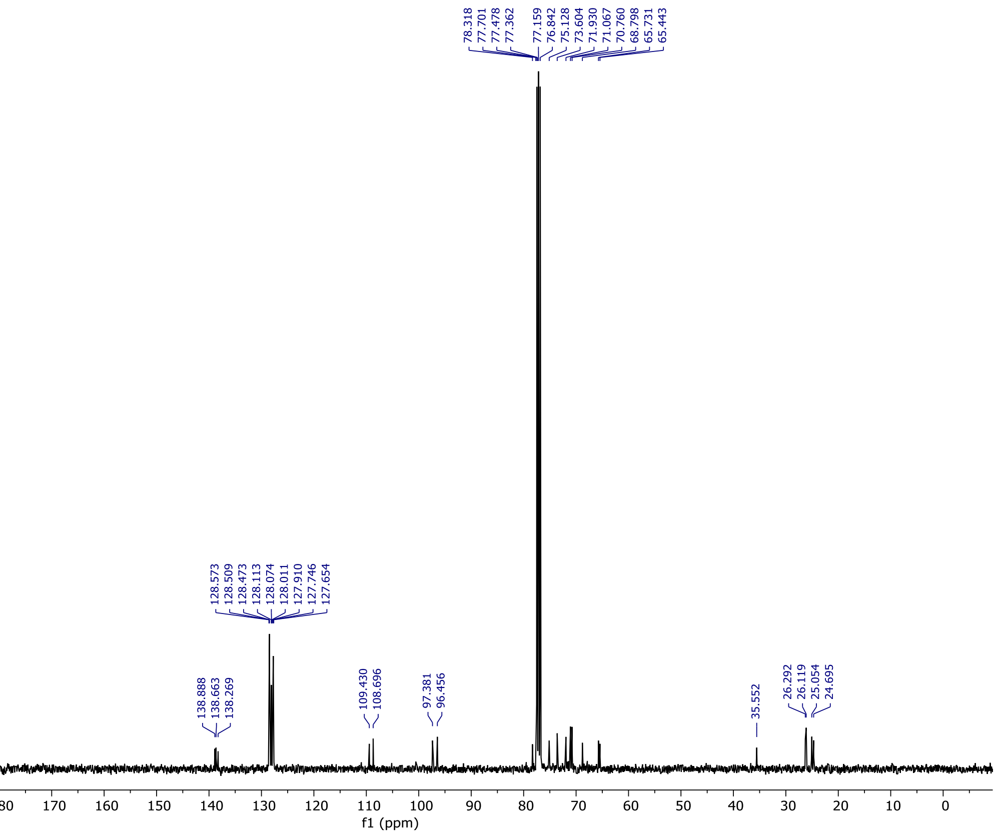
78.318
77.701
77.478
77.362
77.159
76.842
75.128
73.604
71.930
71.067
70.760
68.798
65.731
65.443
128.573
128.509
128.473
128.113
128.074
128.011
127.910
127.746
127.654
138.888
138.663
138.269
109.430
108.696
97.381
96.456
35.552
26.292
26.119
25.054
24.695
220 210 200 190 180 170 160 150 140 130 120 110 100 90 80 70 60 50 40 30 20 10 0
f1 (ppm)

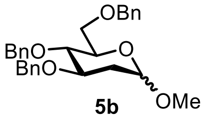
OBn
BnO
BnO
O
OMe

5b

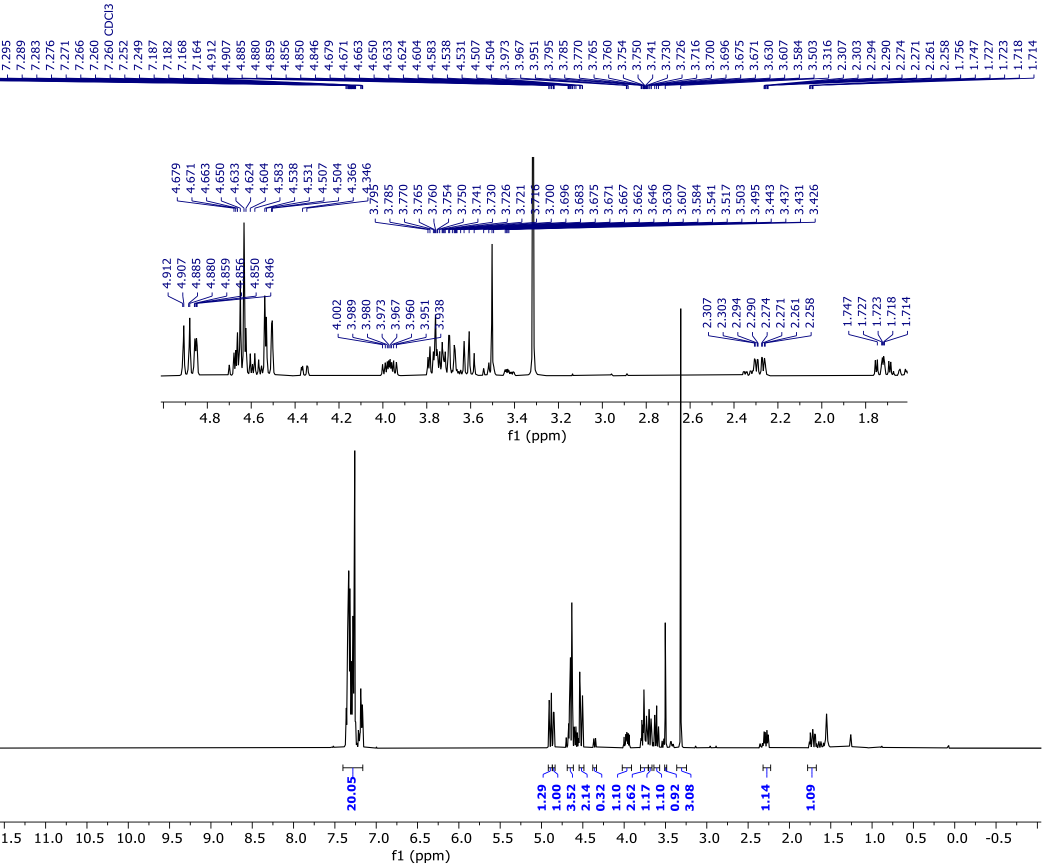
7.366
7.361
7.345
7.340
7.334
7.332
7.327
7.324
7.317
7.315
7.309
7.306
7.301
7.298
7.295
7.289
7.283
7.276
7.271
7.266
7.260
7.260 CDCl3
7.252
7.249
7.187
7.182
7.168
7.164
4.912
4.907
4.885
4.880
4.859
4.856
4.850
4.846
4.679
4.671
4.663
4.650
4.633
4.624
4.604
4.583
4.538
4.531
4.507
4.504
3.973
3.967
3.951
3.795
3.785
3.770
3.765
3.760
3.754
3.750
3.741
3.730
3.726
3.716
3.700
3.696
3.675
3.671
3.630
3.607
3.584
3.503
3.316
2.307
2.303
2.294
2.290
2.274
2.271
2.261
2.258
1.756
1.747
1.727
1.723
1.718
1.714
4.679
4.671
4.663
4.650
4.633
4.624
4.604
4.583
4.538
4.531
4.507
4.504
4.366
4.346
3.795
3.785
3.770
3.765
3.760
3.754
3.750
3.741
3.730
3.726
3.721
3.716
3.700
3.696
3.683
3.675
3.671
3.667
3.662
3.646
3.630
3.607
3.584
3.541
3.517
3.503
3.495
3.443
3.437
3.431
3.426
4.912
4.907
4.885
4.880
4.859
4.856
4.850
4.846
4.002
3.989
3.980
3.973
3.967
3.960
3.951
3.938
2.307
2.303
2.294
2.290
2.274
2.271
2.261
2.258
1.747
1.727
1.723
1.718
1.714
4.8 4.6 4.4 4.2 4.0 3.8 3.6 3.4 3.2 3.0 2.8 2.6 2.4 2.2 2.0 1.8
f1 (ppm)
20.05
1.29
1.00
3.52
2.14
0.32
1.10
2.62
1.17
1.10
0.92
3.08
1.14
1.09
13.5 13.0 12.5 12.0 11.5 11.0 10.5 10.0 9.5 9.0 8.5 8.0 7.5 7.0 6.5 6.0 5.5 5.0 4.5 4.0 3.5 3.0 2.5 2.0 1.5 1.0 0.5 0.0 -0.5
f1 (ppm)

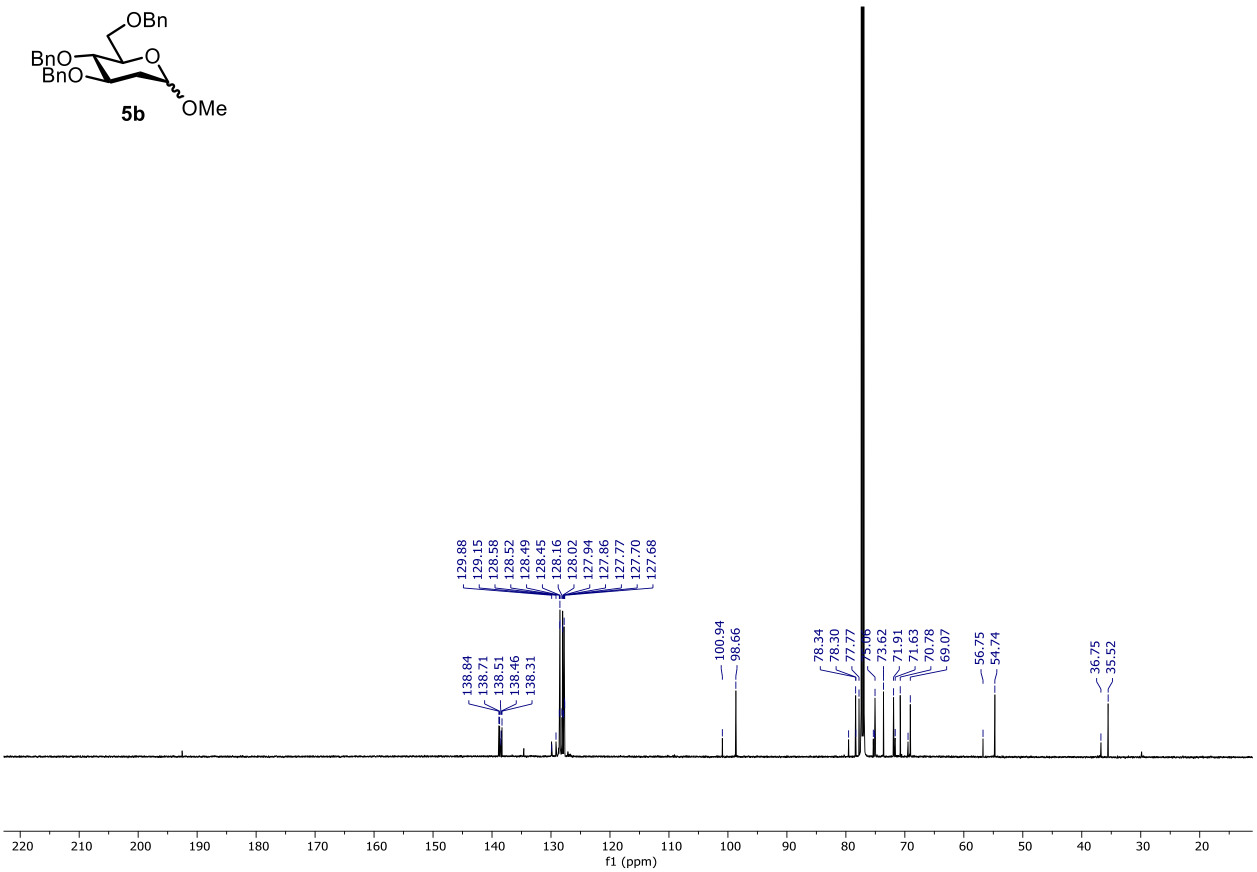

OBn
BnO
BnO
O
OMe
5b
f1 (ppm)

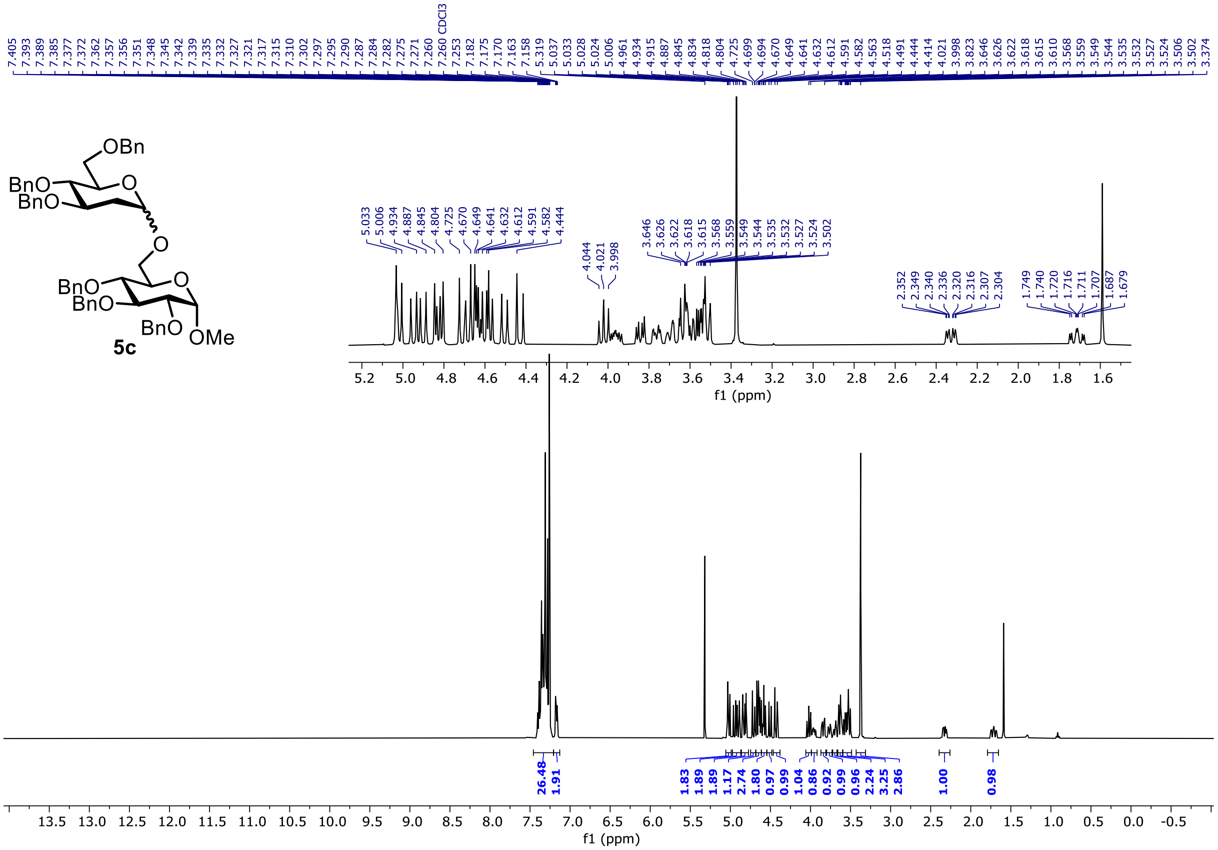

CDCl3
OBn
BnO
BnO
O
O
BnO
BnO
BnO
O
OMe
5c
f1 (ppm)

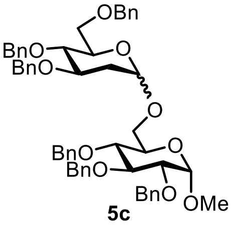
OBn
BnO
BnO
O
O
BnO
BnO
BnO
O
OMe

5c

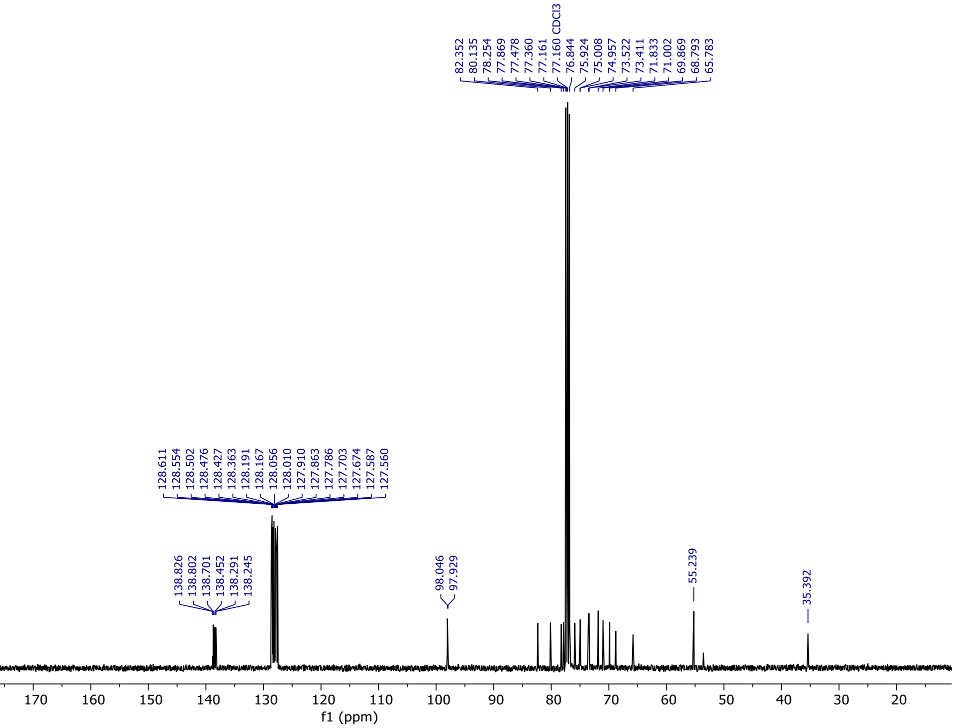
82.352
80.135
78.254
77.869
77.478
77.360
77.161
77.160 CDCl3
76.844
75.924
75.008
74.957
73.522
73.411
71.833
71.002
69.869
68.793
65.783
128.611
128.554
128.502
128.476
128.427
128.363
128.191
128.167
128.056
128.010
127.910
127.863
127.786
127.703
127.674
127.587
127.560
138.826
138.802
138.701
138.452
138.291
138.245
98.046
97.929
55.239
35.392
210 200 190 180 170 160 150 140 130 120 110 100 90 80 70 60 50 40 30 20
f1 (ppm)

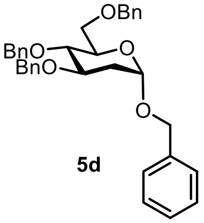
OBn
BnO
BnO
O
O

5d

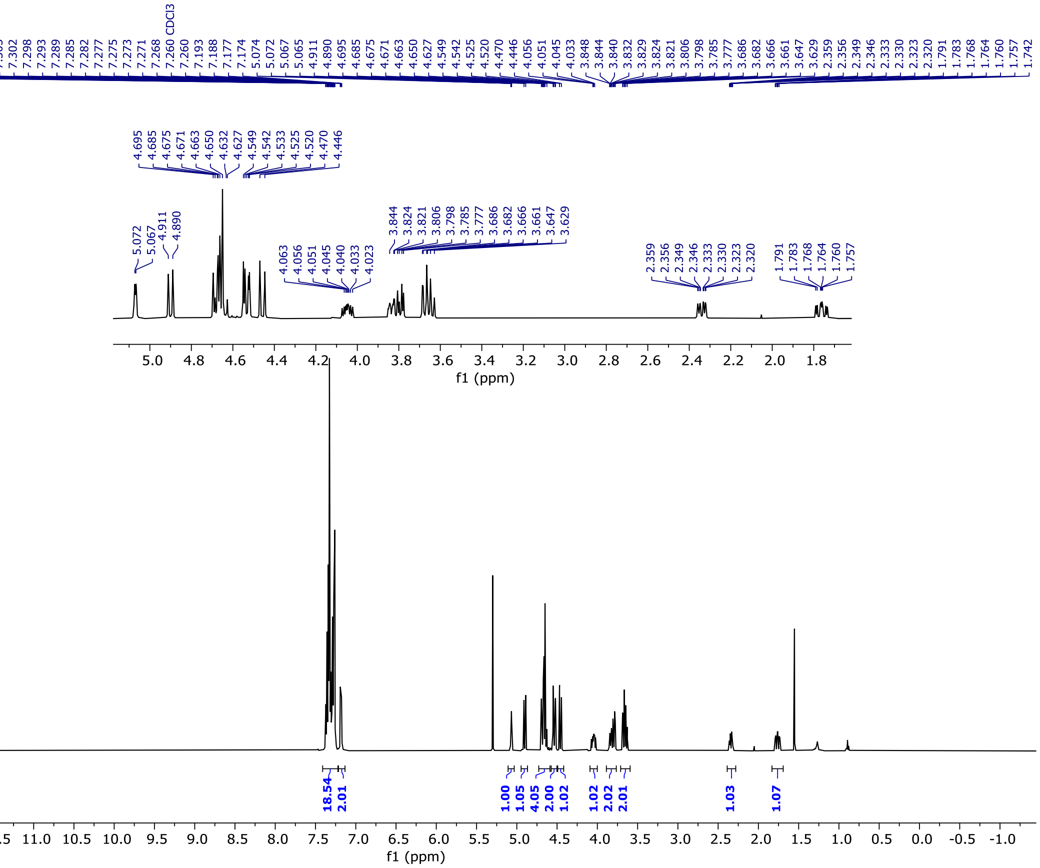
7.375
7.371
7.358
7.355
7.351
7.342
7.340
7.338
7.332
7.327
7.314
7.312
7.310
7.308
7.305
7.302
7.298
7.293
7.289
7.285
7.282
7.277
7.275
7.273
7.271
7.268
7.260 CDCl3
7.260
7.193
7.188
7.177
7.174
5.074
5.072
5.067
5.065
4.911
4.890
4.695
4.685
4.675
4.671
4.663
4.650
4.627
4.549
4.542
4.525
4.520
4.470
4.446
4.056
4.051
4.045
4.033
3.848
3.844
3.840
3.832
3.829
3.824
3.821
3.806
3.798
3.785
3.777
3.686
3.682
3.666
3.661
3.647
3.629
2.359
2.356
2.349
2.346
2.333
2.330
2.323
2.320
1.791
1.783
1.768
1.764
1.760
1.757
1.742
4.695
4.685
4.675
4.671
4.663
4.650
4.632
4.627
4.549
4.542
4.533
4.525
4.520
4.470
4.446
5.072
5.067
4.911
4.890
4.063
4.056
4.051
4.045
4.040
4.033
4.023
3.844
3.821
3.806
3.798
3.785
3.777
3.686
3.682
3.666
3.661
3.647
3.629
2.359
2.356
2.349
2.346
2.333
2.330
2.323
2.320
1.791
1.783
1.768
1.764
1.760
1.757
5.0 4.8 4.6 4.4 4.2 4.0 3.8 3.6 3.4 3.2 3.0 2.8 2.6 2.4 2.2 2.0 1.8
f1 (ppm)
18.54
2.01
1.00
1.05
4.05
2.00
1.02
1.02
2.02
2.01
1.03
1.07
13.5 13.0 12.5 12.0 11.5 11.0 10.5 10.0 9.5 9.0 8.5 8.0 7.5 7.0 6.5 6.0 5.5 5.0 4.5 4.0 3.5 3.0 2.5 2.0 1.5 1.0 0.5 0.0 -0.5 -1.0
f1 (ppm)

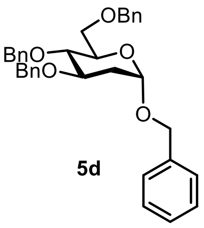
OBn
BnO
BnO
O

5d

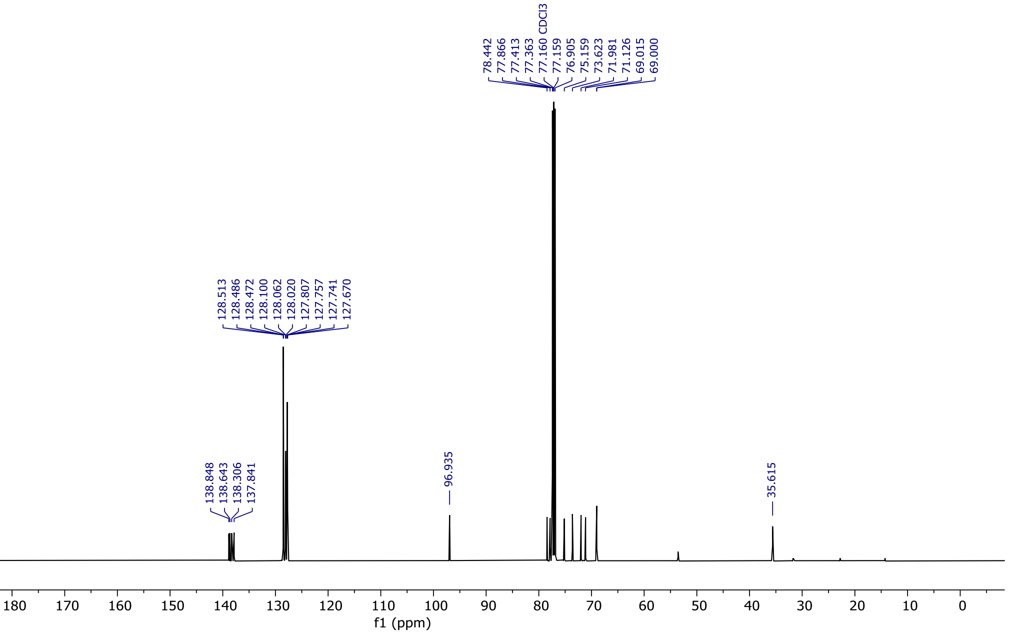
CDCl3
f1 (ppm)

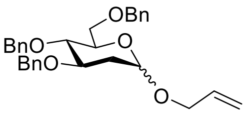
OBn
BnO
BnO
O

5e

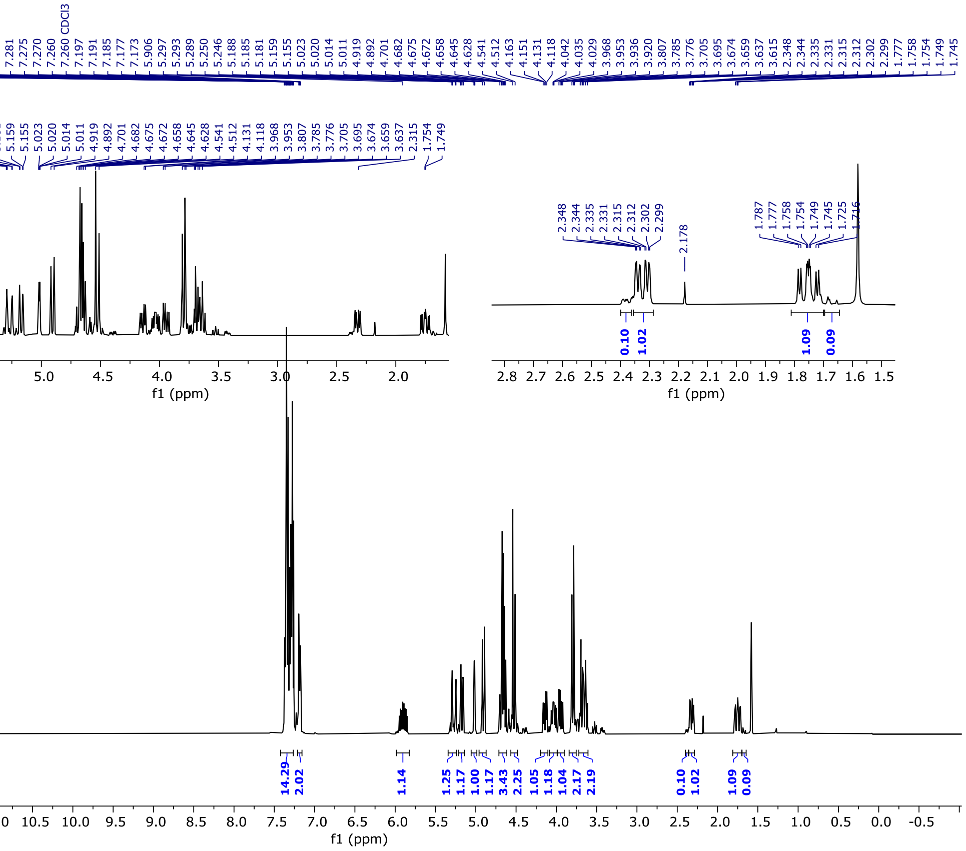
CDCl3
f1 (ppm)
f1 (ppm)
f1 (ppm)

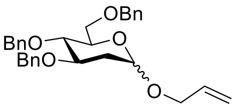
OBn
BnO
BnO
O
O

5e

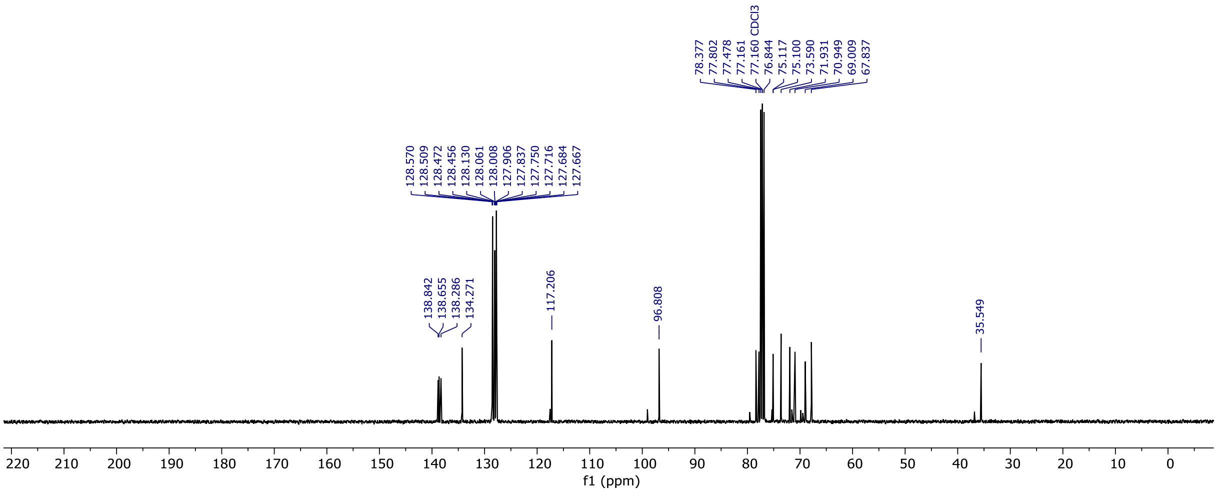
78.377
77.802
77.478
77.161
77.160 CDCl3
76.844
75.117
75.100
73.590
71.931
70.949
69.009
67.837
128.570
128.509
128.472
128.456
128.130
128.061
128.008
127.906
127.837
127.750
127.716
127.684
127.667
138.842
138.655
138.286
134.271
117.206
96.808
35.549
220 210 200 190 180 170 160 150 140 130 120 110 100 90 80 70 60 50 40 30 20 10 0
f1 (ppm)

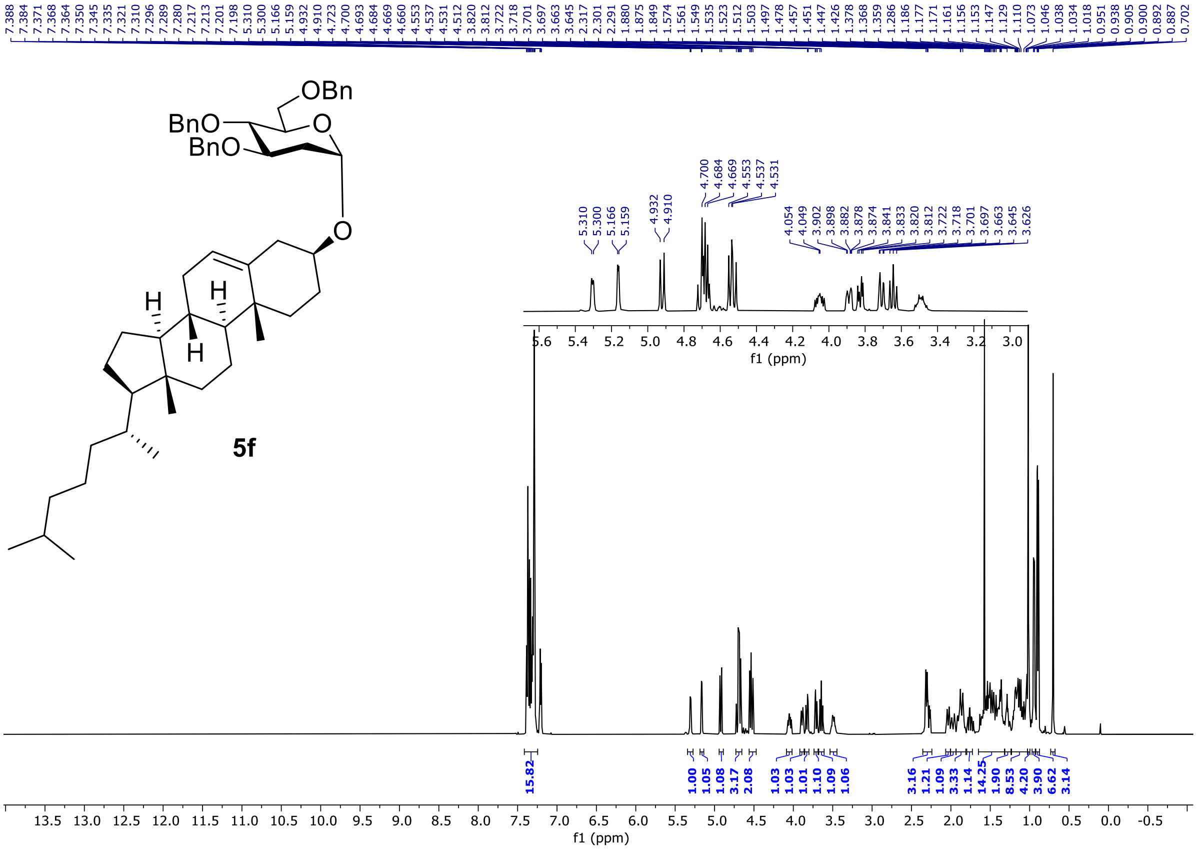
7.388 7.384 7.371 7.368 7.364 7.350 7.345 7.335 7.321 7.310 7.296 7.289 7.280 7.217 7.213 7.201 7.198 5.310 5.300 5.166 5.159 4.932 4.910 4.723 4.700 4.693 4.684 4.669 4.660 4.553 4.537 4.531 4.512 3.820 3.812 3.722 3.718 3.701 3.697 3.663 3.645 2.317 2.301 2.291 1.880 1.875 1.849 1.574 1.561 1.549 1.535 1.523 1.512 1.503 1.497 1.478 1.457 1.451 1.447 1.426 1.378 1.368 1.359 1.286 1.186 1.177 1.171 1.161 1.156 1.153 1.147 1.129 1.110 1.073 1.046 1.038 1.034 1.018 0.951 0.938 0.905 0.900 0.892 0.887 0.702
BnO
BnO
O
OBn
O
H
H
H
5.310 5.300 5.166 5.159 4.932 4.910 4.700 4.684 4.669 4.553 4.537 4.531
4.054 4.049 3.902 3.898 3.882 3.878 3.874 3.841 3.833 3.820 3.812 3.722 3.718 3.701 3.697 3.663 3.645 3.626
f1 (ppm)
15.82 1.00 1.05 1.08 3.17 2.08 1.03 1.03 1.01 1.10 1.09 1.06 3.16 1.21 1.09 3.33 1.14 14.25 1.90 8.53 4.20 3.90 6.62 3.14
f1 (ppm)

5f

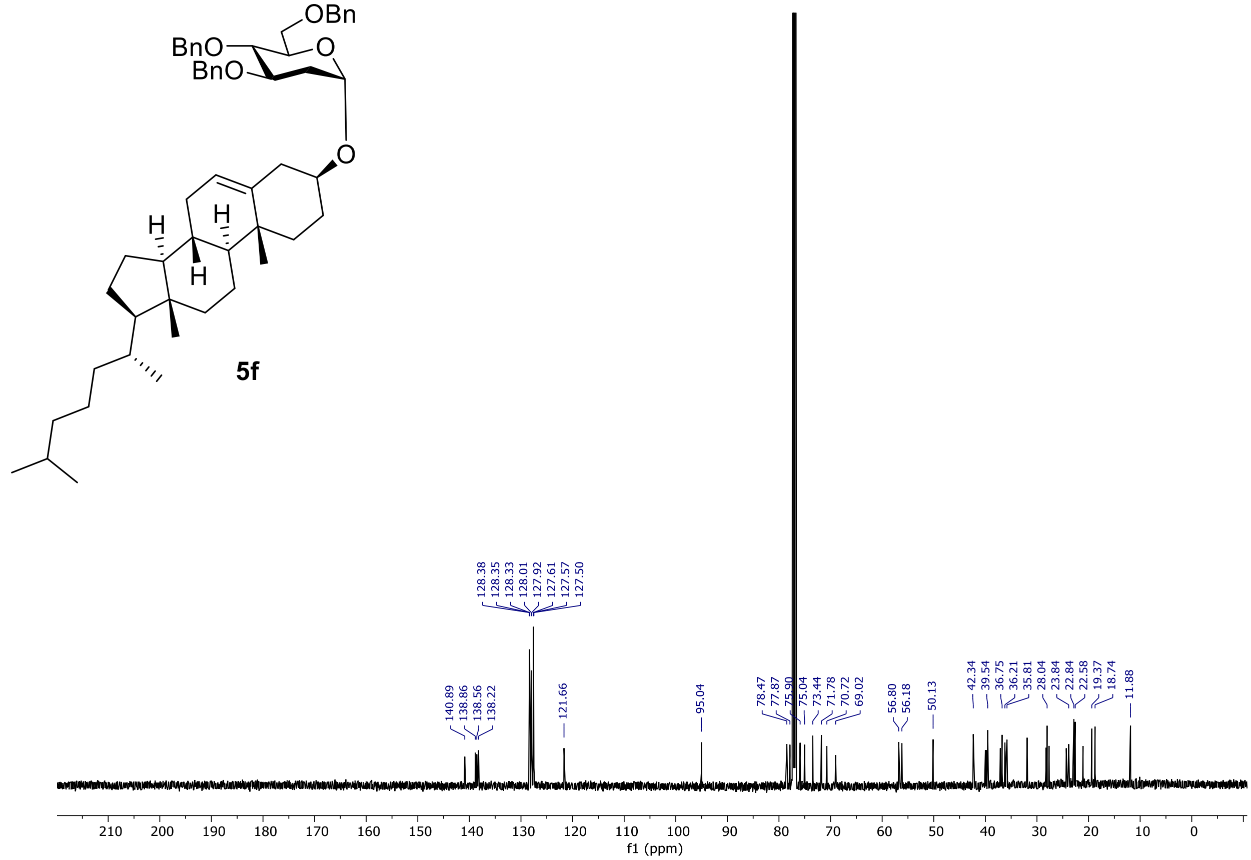
BnO
BnO
O
OBn
O
H
H
H
128.38 128.35 128.33 128.01 127.92 127.61 127.57 127.50
140.89 138.86 138.56 138.22 121.66 95.04 78.47 77.87 75.90 75.04 73.44 71.78 70.72 69.02 56.80 56.18 50.13 42.34 39.54 36.75 36.21 35.81 28.04 23.84 22.84 22.58 19.37 18.74 11.88
f1 (ppm)

5f

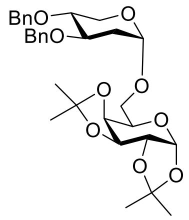
BnO
BnO

6a

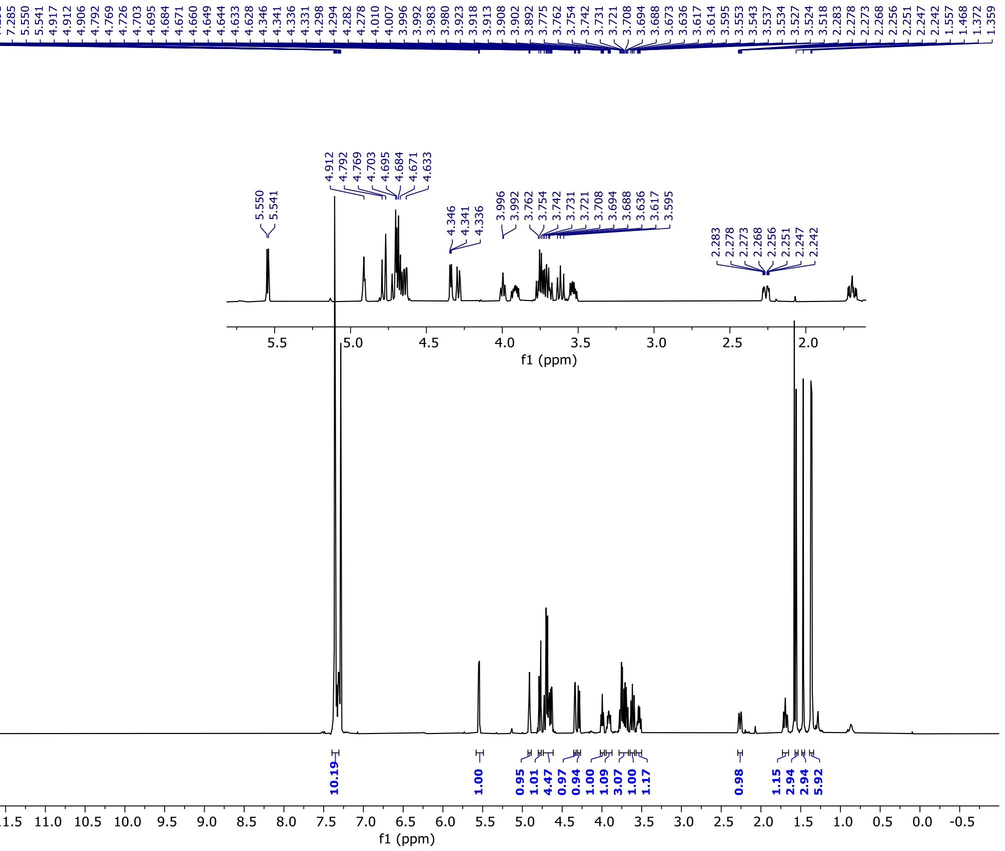
7.372 7.362 7.355 7.351 7.345 7.339 7.336 7.334 7.321 7.315 7.310 7.303 7.297 7.291 7.285 5.550 5.541 4.917 4.912 4.906 4.792 4.769 4.726 4.703 4.695 4.684 4.671 4.660 4.649 4.644 4.633 4.628 4.346 4.341 4.336 4.331 4.298 4.294 4.282 4.278 4.010 4.007 3.996 3.992 3.983 3.980 3.923 3.918 3.913 3.908 3.902 3.892 3.775 3.762 3.754 3.742 3.731 3.721 3.708 3.694 3.688 3.673 3.636 3.617 3.614 3.595 3.553 3.543 3.537 3.534 3.527 3.524 3.518 2.283 2.278 2.273 2.268 2.256 2.251 2.247 2.242 1.557 1.468 1.372 1.359
5.550 5.541
4.912 4.792 4.769 4.703 4.695 4.684 4.671 4.633
4.346 4.341 4.336
3.996 3.992 3.762 3.754 3.742 3.731 3.721 3.708 3.694 3.688 3.636 3.617 3.595
2.283 2.278 2.273 2.268 2.256 2.251 2.247 2.242
5.5 5.0 4.5 4.0 3.5 3.0 2.5 2.0
f1 (ppm)
10.19 1.00 0.95 1.01 4.47 0.97 0.94 1.00 1.09 3.07 1.00 1.17 0.98 1.15 2.94 2.94 5.92
13.5 13.0 12.5 12.0 11.5 11.0 10.5 10.0 9.5 9.0 8.5 8.0 7.5 7.0 6.5 6.0 5.5 5.0 4.5 4.0 3.5 3.0 2.5 2.0 1.5 1.0 0.5 0.0 -0.5
f1 (ppm)

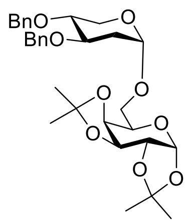
BnO
BnO
O
O
O
O
O
O
O

6a

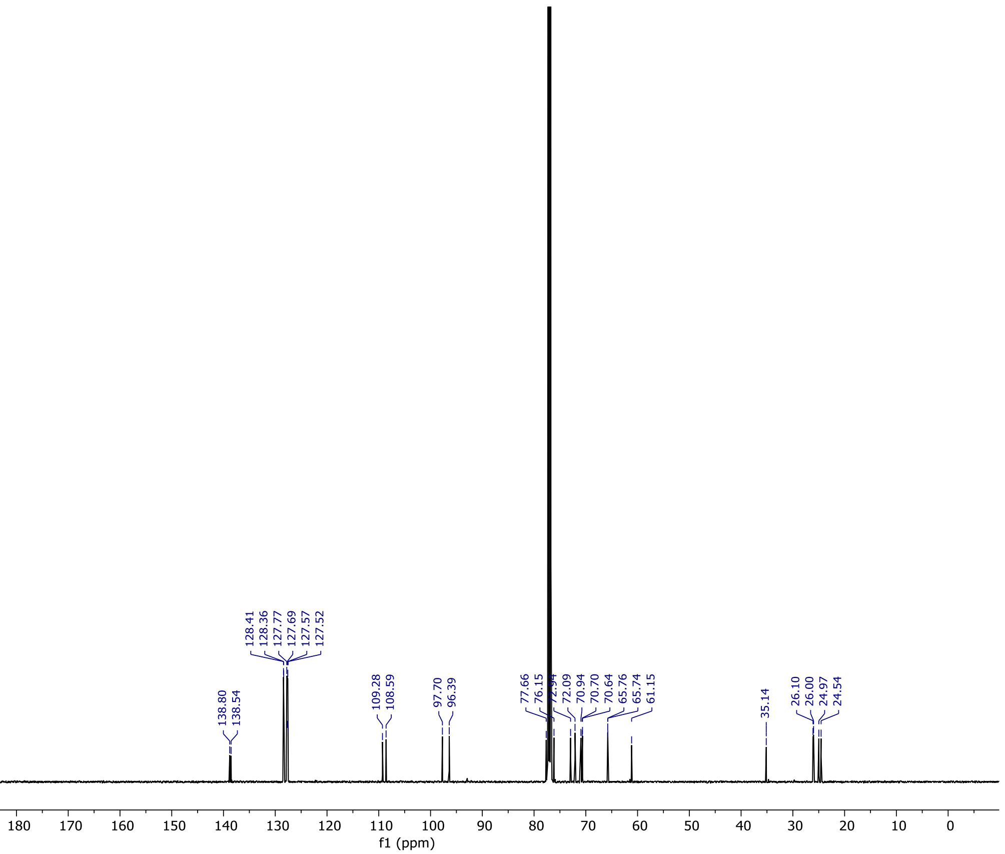
128.41
128.36
127.77
127.69
127.57
127.52
138.80
138.54
109.28
108.59
97.70
96.39
77.66
76.15
72.94
72.09
70.94
70.70
70.64
65.76
65.74
61.15
35.14
26.10
26.00
24.97
24.54
210 200 190 180 170 160 150 140 130 120 110 100 90 80 70 60 50 40 30 20 10 0
f1 (ppm)

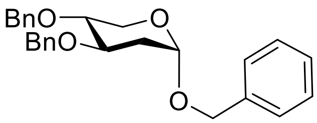
BnO
BnO
O
O

6b

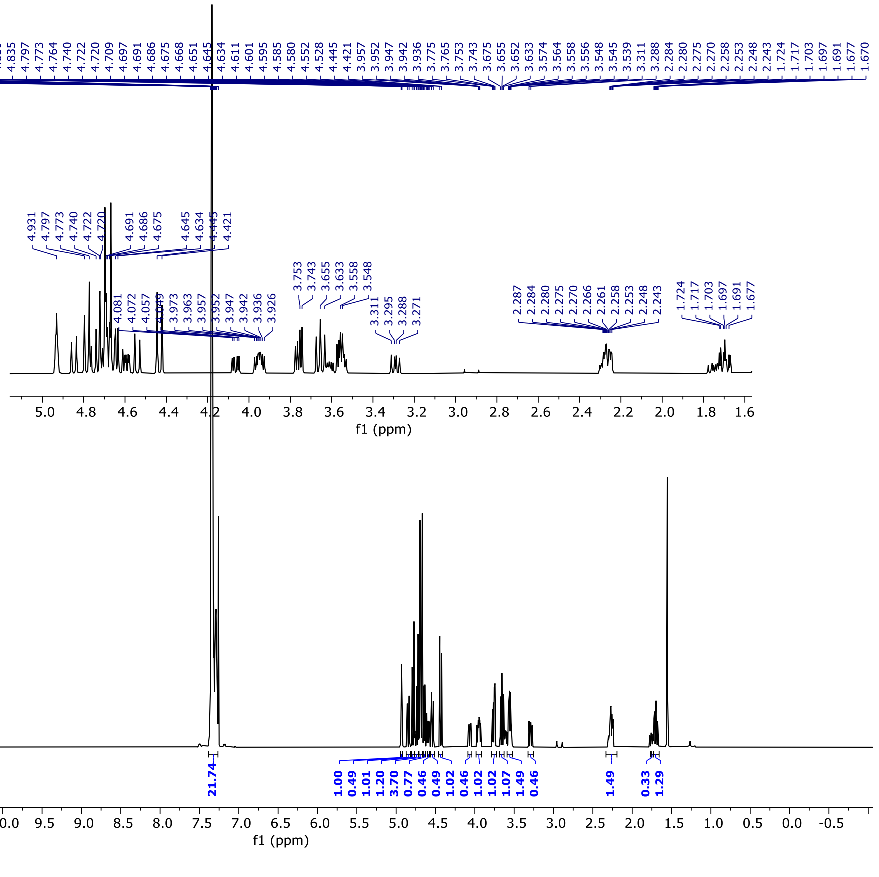
7.360
7.356
7.355
7.347
7.342
7.335
7.331
7.319
7.314
7.307
7.304
7.300
7.296
7.294
7.289
7.284
7.279
CDCl3
7.260
4.937
4.931
4.925
4.859
4.835
4.797
4.773
4.764
4.740
4.722
4.720
4.709
4.697
4.691
4.686
4.675
4.668
4.651
4.645
4.634
4.611
4.601
4.595
4.585
4.580
4.552
4.528
4.445
4.421
3.957
3.952
3.947
3.942
3.936
3.775
3.765
3.753
3.743
3.675
3.655
3.652
3.633
3.574
3.564
3.558
3.556
3.548
3.545
3.539
3.311
3.288
2.284
2.280
2.275
2.270
2.258
2.253
2.248
2.243
1.724
1.717
1.703
1.697
1.691
1.691
1.677
1.670
4.931
4.797
4.773
4.740
4.722
4.720
4.691
4.686
4.675
4.645
4.634
4.445
4.421
4.081
4.072
4.057
4.049
3.973
3.963
3.957
3.952
3.947
3.942
3.936
3.926
3.753
3.743
3.655
3.633
3.558
3.548
3.311
3.295
3.288
3.271
2.287
2.284
2.280
2.275
2.270
2.266
2.261
2.258
2.253
2.248
2.243
1.724
1.717
1.703
1.697
1.691
1.677
5.0 4.8 4.6 4.4 4.2 4.0 3.8 3.6 3.4 3.2 3.0 2.8 2.6 2.4 2.2 2.0 1.8 1.6
f1 (ppm)
21.74
1.00
0.49
1.01
1.20
3.70
0.77
0.46
0.49
1.02
0.46
1.02
1.02
1.07
1.49
0.46
1.49
0.33
1.29
13.5 13.0 12.5 12.0 11.5 11.0 10.5 10.0 9.5 9.0 8.5 8.0 7.5 7.0 6.5 6.0 5.5 5.0 4.5 4.0 3.5 3.0 2.5 2.0 1.5 1.0 0.5 0.0 -0.5
f1 (ppm)

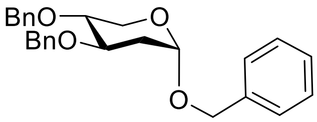
BnO
BnO
O
O

6b

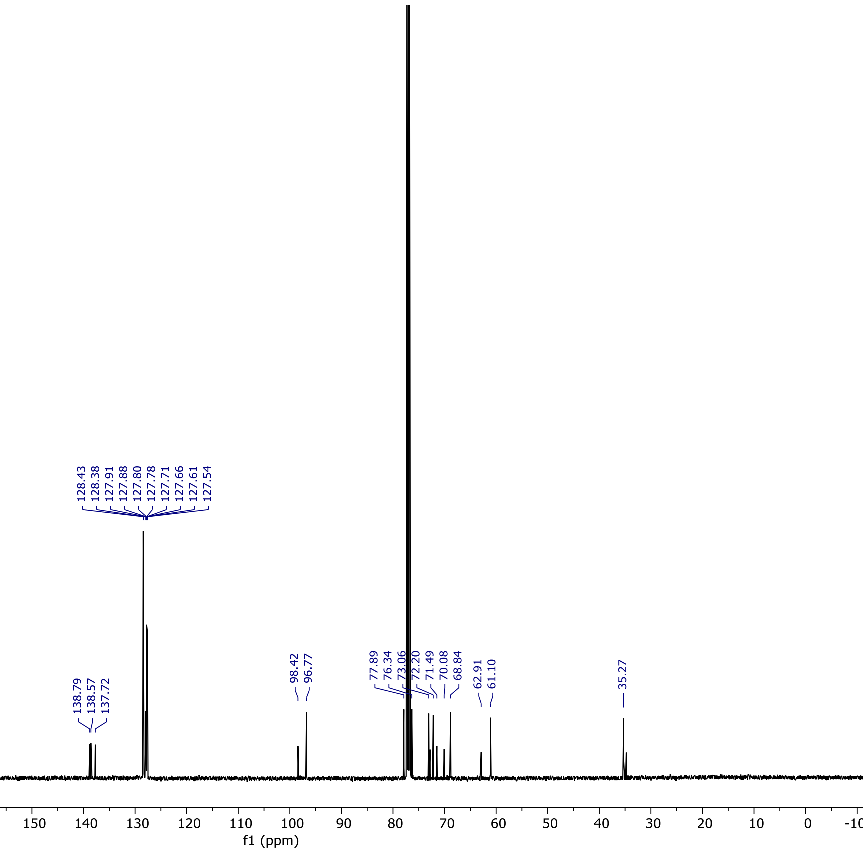
128.43
128.38
127.91
127.88
127.80
127.78
127.71
127.66
127.61
127.54
138.79
138.57
137.72
98.42
96.77
77.89
76.34
73.06
72.20
71.49
70.08
68.84
62.91
61.10
35.27
210 200 190 180 170 160 150 140 130 120 110 100 90 80 70 60 50 40 30 20 10 0 -10
f1 (ppm)

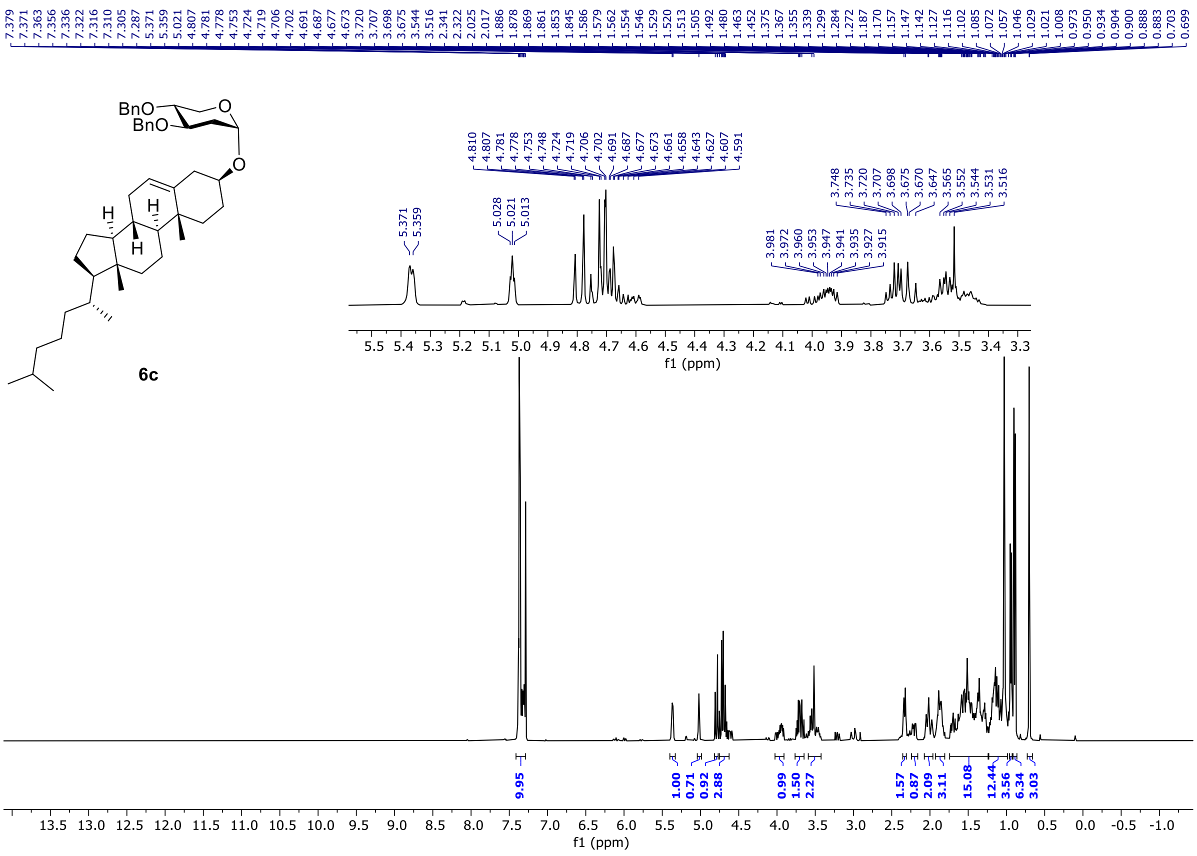
BnO
BnO
O
H
H
H
6c
f1 (ppm)

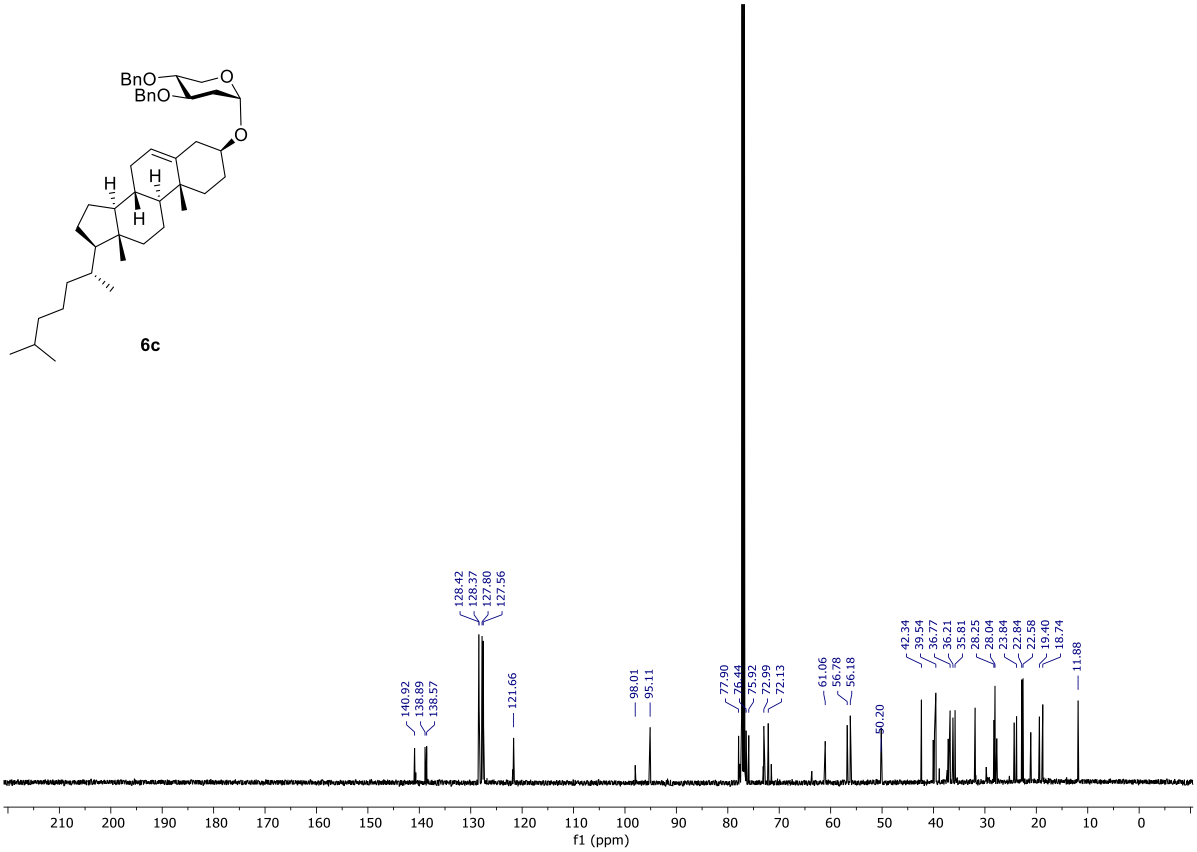
BnO
BnO
O
H
H
H
6c
f1 (ppm)

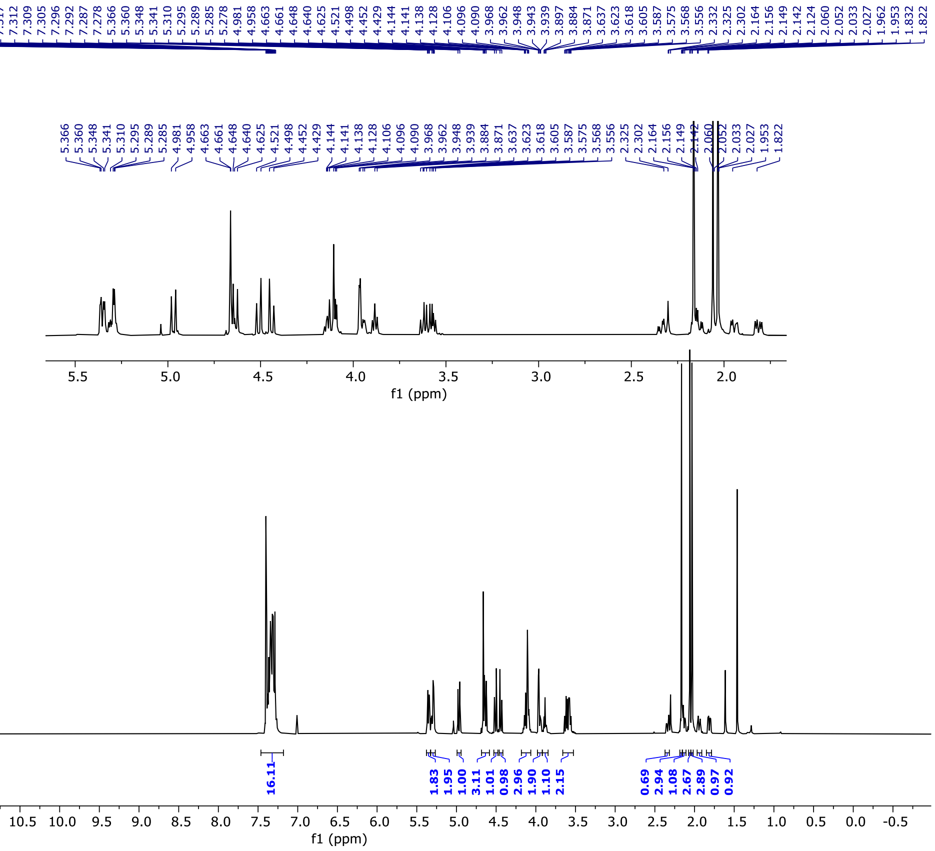

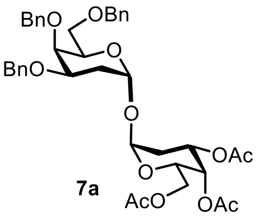

7a

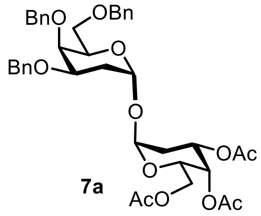
BnO
OBn
BnO
O
O
OAc
O
AcO
OAc

7a

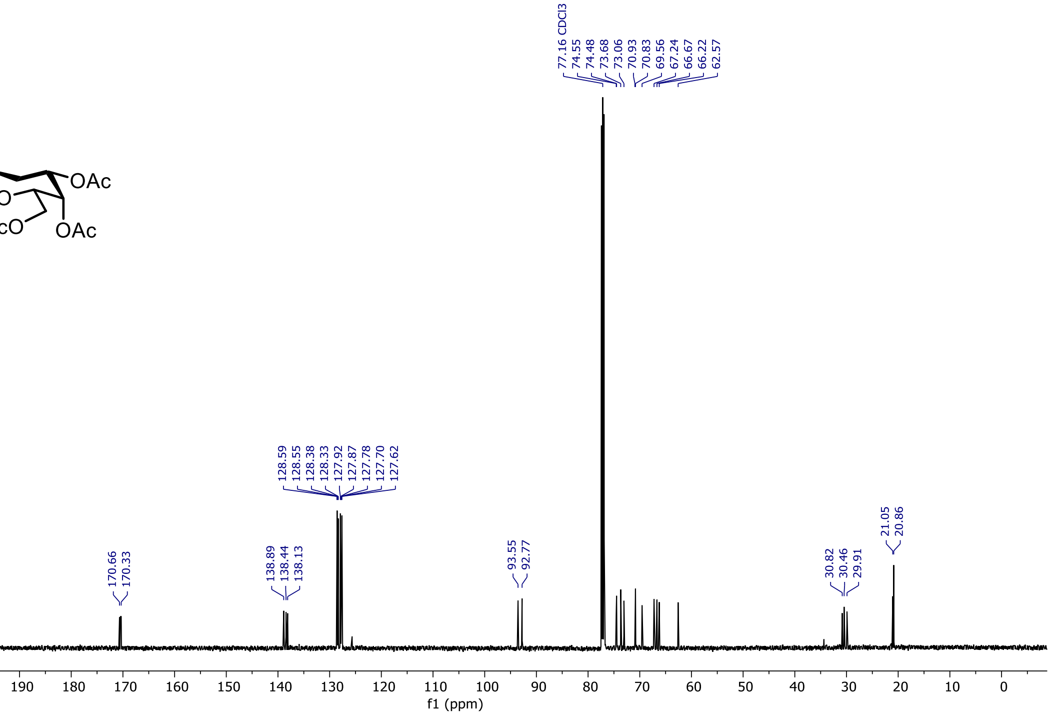
77.16 CDCl3
74.55
74.48
73.68
73.06
70.93
70.83
69.56
67.24
66.67
66.22
62.57
128.59
128.55
128.38
128.33
127.92
127.87
127.78
127.70
127.62
170.66
170.33
138.89
138.44
138.13
93.55
92.77
30.82
30.46
29.91
21.05
20.86
f1 (ppm)

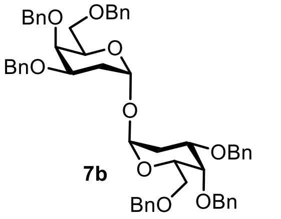
BnO
OBn
BnO
O
O
OBn
O
BnO
OBn

7b

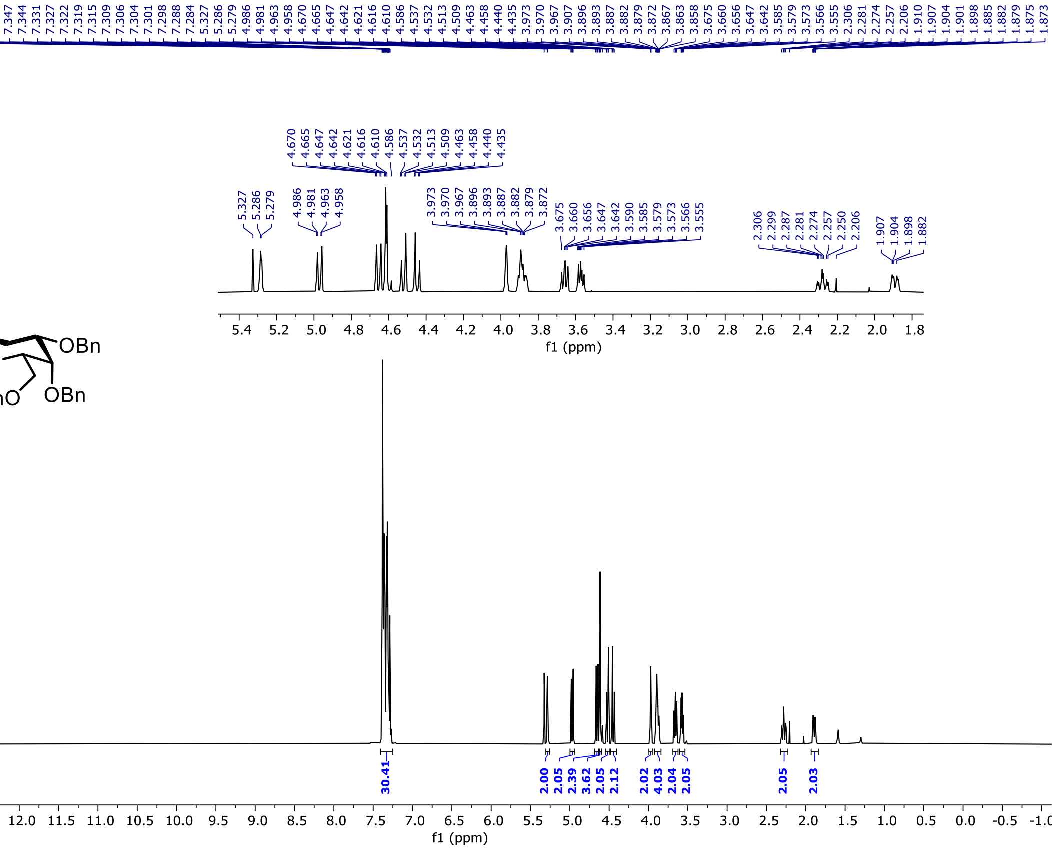
5.327
5.286
5.279
4.986
4.981
4.963
4.958
4.670
4.665
4.647
4.642
4.621
4.616
4.610
4.586
4.537
4.532
4.513
4.509
4.463
4.458
4.440
4.435
3.973
3.970
3.967
3.896
3.893
3.887
3.882
3.879
3.872
3.675
3.660
3.656
3.647
3.642
3.590
3.585
3.579
3.573
3.566
3.555
2.306
2.299
2.287
2.281
2.274
2.257
2.250
2.206
1.907
1.904
1.898
1.882
f1 (ppm)
30.41
2.00
2.05
2.39
3.62
2.05
2.12
2.02
4.03
2.04
2.05
2.05
2.03
f1 (ppm)

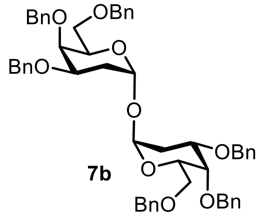

7b

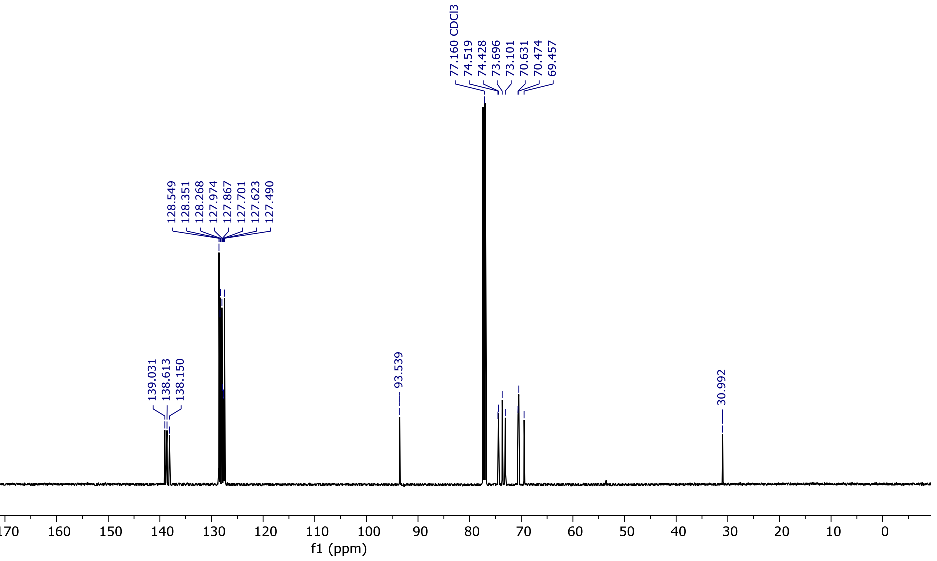
139.031
138.613
138.150
128.549
128.351
128.268
127.974
127.867
127.701
127.623
127.490
93.539
77.160 CDCl3
74.519
74.428
73.696
73.101
70.631
70.474
69.457
30.992
f1 (ppm)

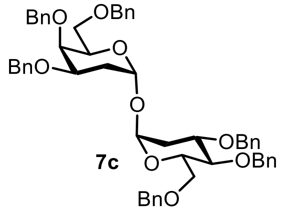

7c

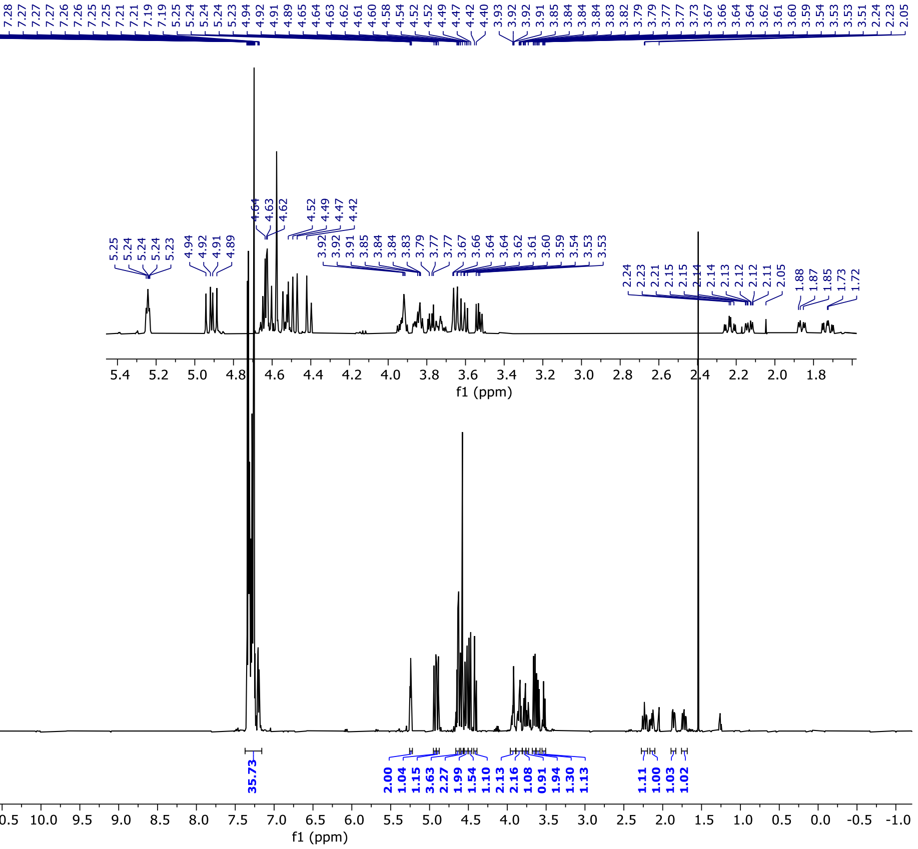
7.35 7.34 7.34 7.33 7.33 7.32 7.32 7.32 7.31 7.31 7.31 7.31 7.30 7.29 7.29 7.29 7.28 7.28 7.27 7.27 7.27 7.26 7.26 7.25 7.25 7.21 7.21 7.19 7.19 5.25 5.24 5.24 5.24 5.23 4.94 4.92 4.91 4.89 4.65 4.64 4.63 4.62 4.61 4.60 4.58 4.54 4.52 4.52 4.52 4.49 4.47 4.42 4.40 3.93 3.92 3.92 3.91 3.85 3.84 3.84 3.83 3.82 3.79 3.79 3.77 3.77 3.73 3.67 3.66 3.64 3.64 3.62 3.61 3.60 3.59 3.54 3.53 3.53 3.51 2.24 2.23 2.05
35.73
2.00 1.04 1.15 3.63 2.27 1.99 1.54 1.10 2.13 2.16 1.08 0.91 1.94 1.30 1.13
1.11 1.00 1.03 1.02
f1 (ppm)

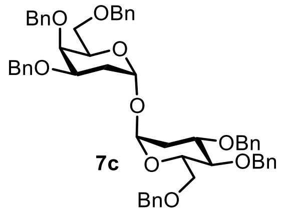

BnO
OBn
O
BnO
O
O
OBn
OBn
BnO
7c

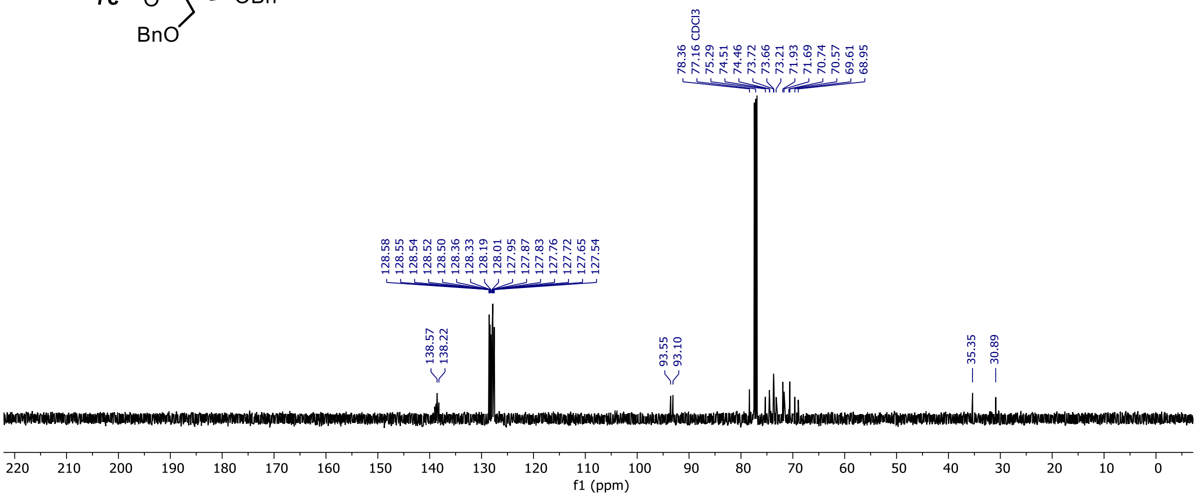

138.57
138.22
128.58
128.55
128.54
128.52
128.50
128.36
128.33
128.19
128.01
127.95
127.87
127.83
127.76
127.72
127.65
127.54
93.55
93.10
78.36
77.16 CDCl3
75.29
74.51
74.46
73.72
73.66
73.21
71.93
71.69
70.74
70.57
69.61
68.95
35.35
30.89
220 210 200 190 180 170 160 150 140 130 120 110 100 90 80 70 60 50 40 30 20 10 0
f1 (ppm)

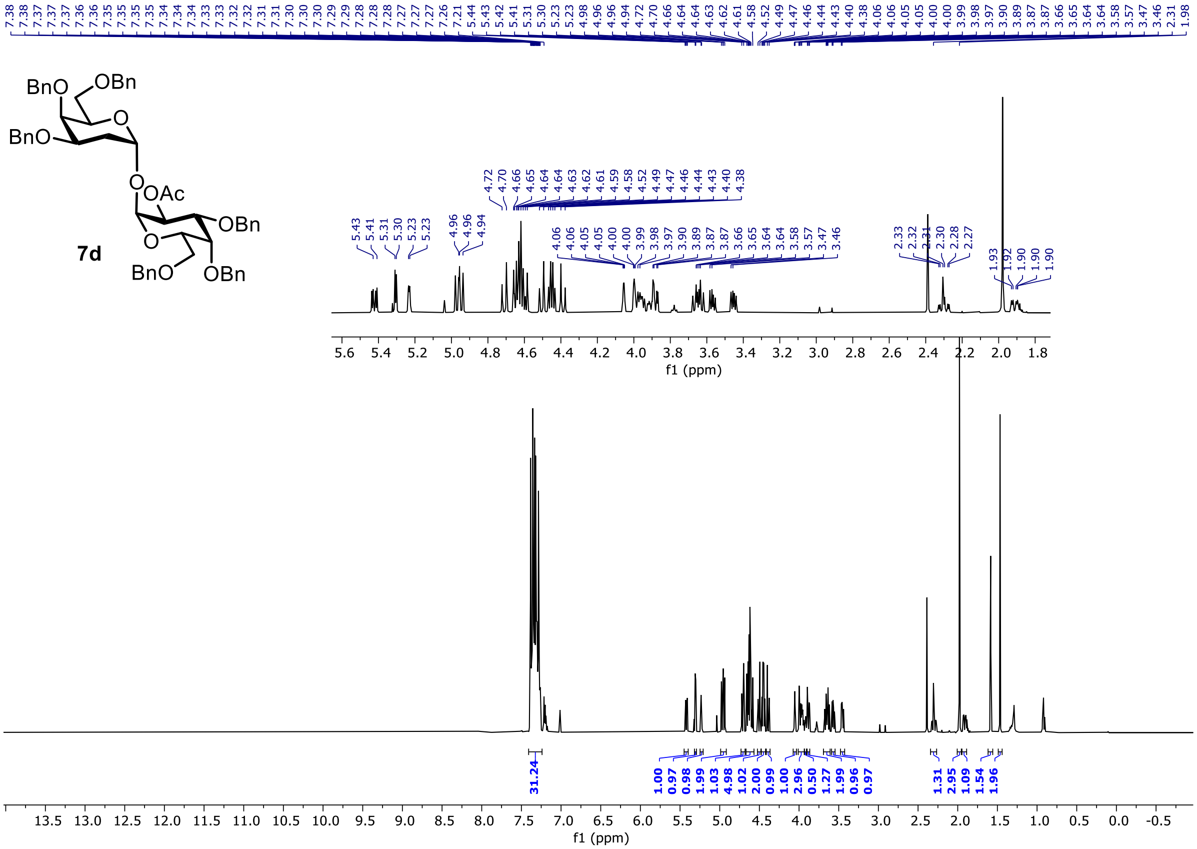

7d

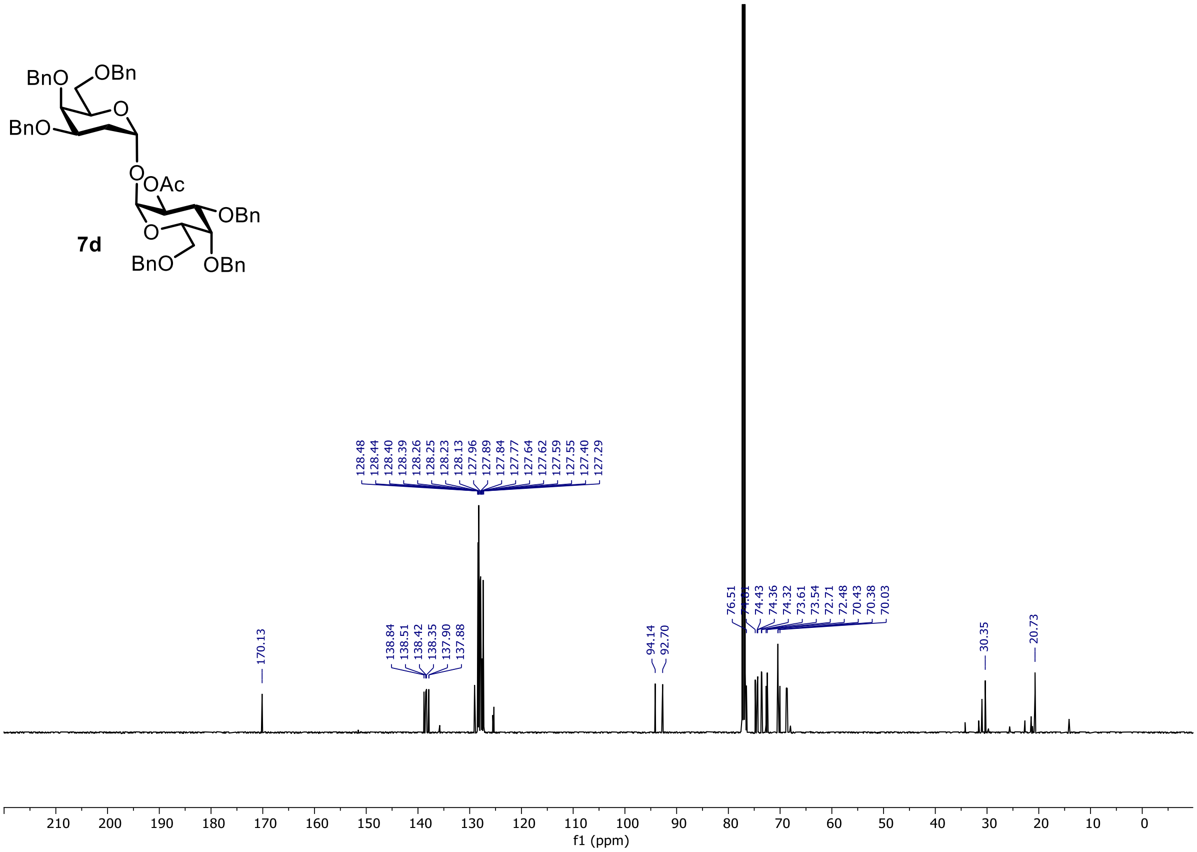

7d

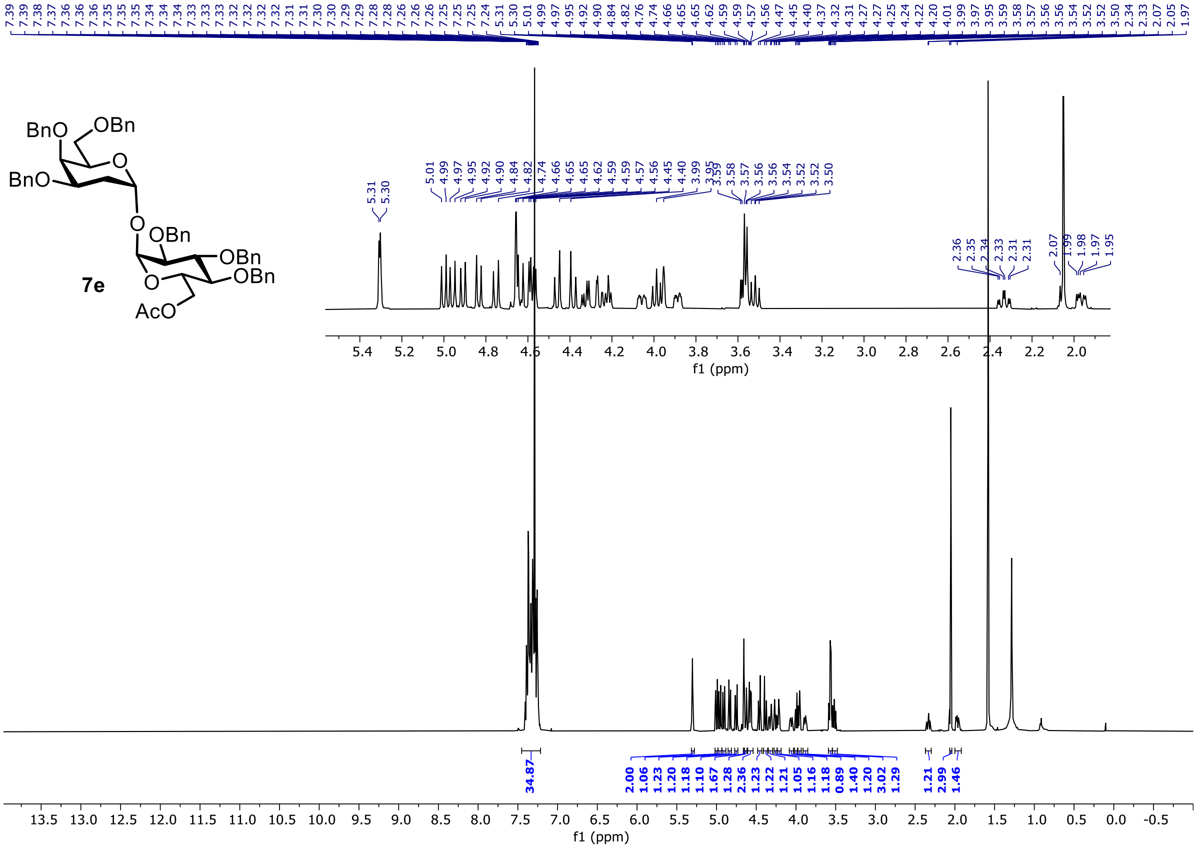

7e
f1 (ppm)

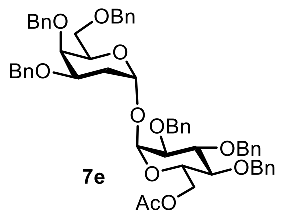
BnO
OBn
O
BnO
O
OBn
OBn
OBn
O
AcO

7e

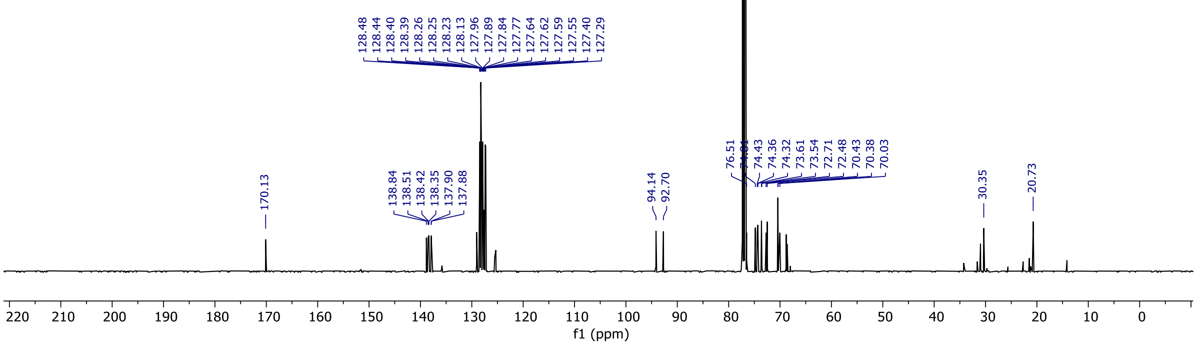
128.48
128.44
128.40
128.39
128.26
128.25
128.23
128.13
127.96
127.89
127.84
127.77
127.64
127.62
127.59
127.55
127.40
127.29
170.13
138.84
138.51
138.42
138.35
137.90
137.88
94.14
92.70
76.51
74.43
74.36
74.32
73.61
73.54
72.71
72.48
70.43
70.38
70.03
30.35
20.73
f1 (ppm)

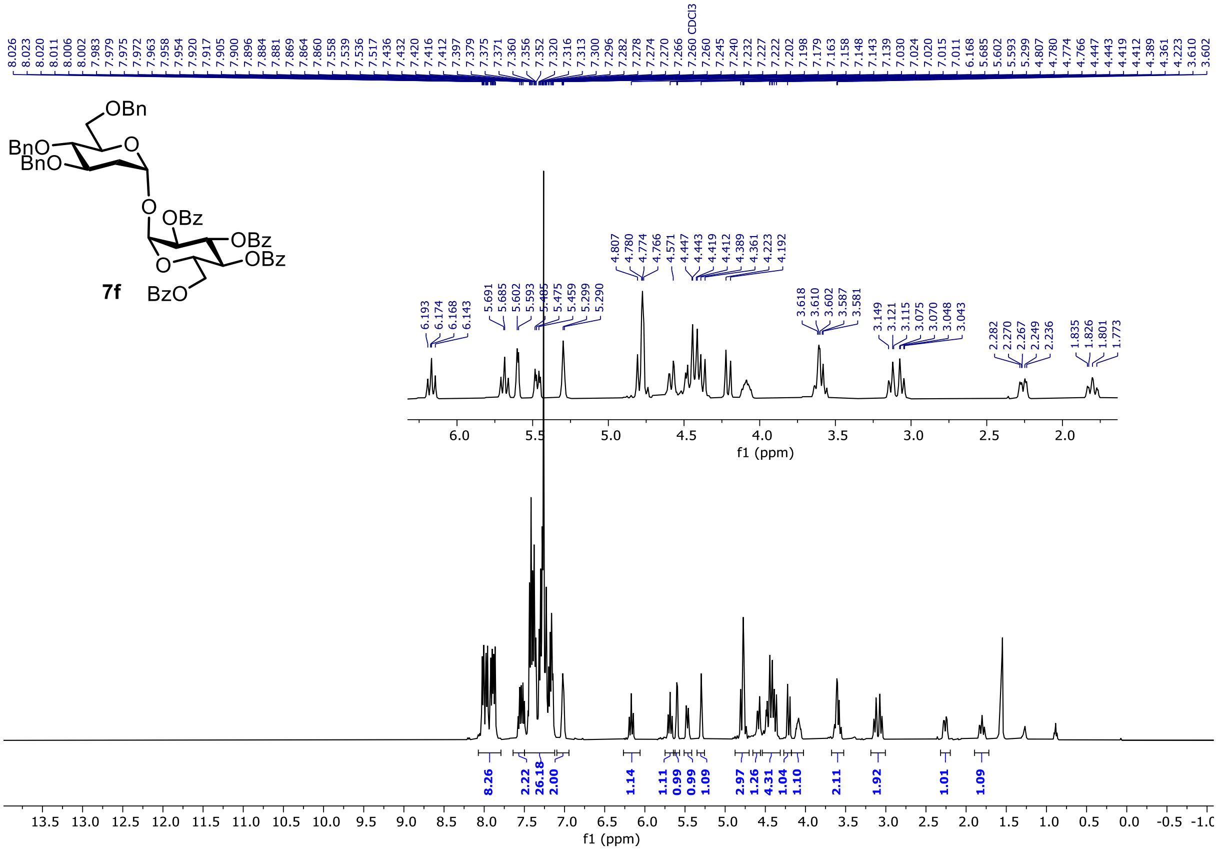

8.026 8.023 8.020 8.011 8.006 8.002 7.983 7.979 7.975 7.972 7.963 7.958 7.954 7.920 7.917 7.905 7.900 7.896 7.884 7.881 7.869 7.864 7.860 7.558 7.539 7.536 7.517 7.436 7.432 7.420 7.416 7.412 7.397 7.379 7.375 7.371 7.360 7.356 7.352 7.320 7.316 7.313 7.300 7.296 7.282 7.278 7.274 7.270 7.266 7.260 CDCl3 7.260 7.245 7.240 7.232 7.227 7.222 7.202 7.198 7.179 7.163 7.158 7.148 7.143 7.139 7.030 7.024 7.020 7.015 7.011 6.168 5.685 5.602 5.593 5.299 4.807 4.780 4.774 4.766 4.447 4.443 4.419 4.412 4.389 4.361 4.223 3.610 3.602
OBn
BnO
BnO
O
O
OBz
OBz
OBz
O
BzO
7f
6.193 6.174 6.168 6.143
5.691 5.685 5.602 5.593 5.485 5.475 5.459 5.299 5.290
4.807 4.780 4.774 4.766 4.571 4.447 4.443 4.419 4.412 4.389 4.361 4.223 4.192
3.618 3.610 3.602 3.587 3.581
3.149 3.121 3.115 3.075 3.070 3.048 3.043
2.282 2.270 2.267 2.249 2.236
1.835 1.826 1.801 1.773
6.0 5.5 5.0 4.5 4.0 3.5 3.0 2.5 2.0
f1 (ppm)
8.26 2.22 26.18 2.00 1.14 1.11 0.99 0.99 1.09 2.97 1.26 4.31 1.04 1.10 2.11 1.92 1.01 1.09
13.5 13.0 12.5 12.0 11.5 11.0 10.5 10.0 9.5 9.0 8.5 8.0 7.5 7.0 6.5 6.0 5.5 5.0 4.5 4.0 3.5 3.0 2.5 2.0 1.5 1.0 0.5 0.0 -0.5 -1.0
f1 (ppm)

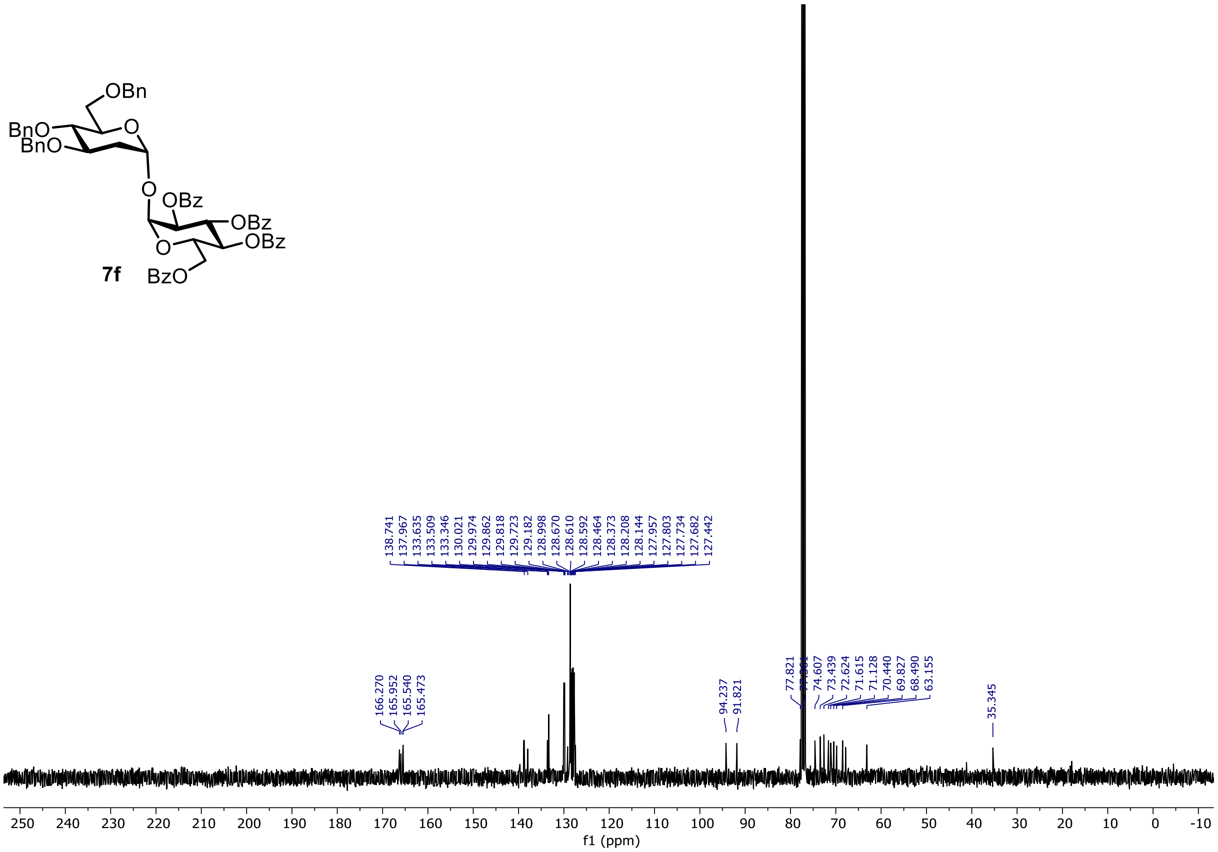

OBn
BnO
BnO
O
O
OBz
OBz
OBz
O
BzO
7f
138.741 137.967 133.635 133.509 133.346 130.021 129.974 129.862 129.818 129.723 129.182 128.998 128.670 128.610 128.592 128.464 128.373 128.208 128.144 127.957 127.803 127.734 127.682 127.442
166.270 165.952 165.540 165.473
94.237 91.821
77.821 74.607 73.439 72.624 71.615 71.128 70.440 69.827 68.490 63.155
35.345
250 240 230 220 210 200 190 180 170 160 150 140 130 120 110 100 90 80 70 60 50 40 30 20 10 0 -10
f1 (ppm)

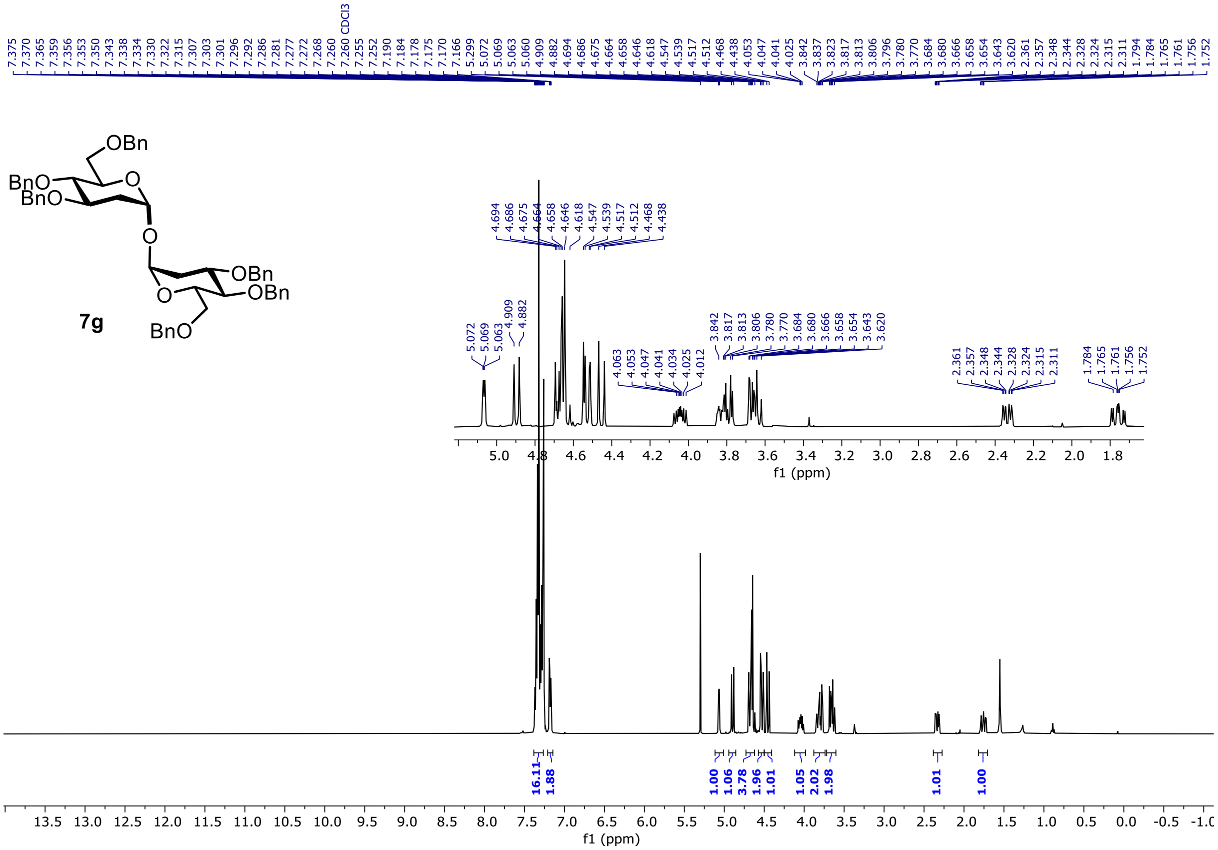
7.375 7.370 7.365 7.359 7.356 7.353 7.350 7.343 7.338 7.334 7.330 7.322 7.315 7.307 7.303 7.301 7.296 7.292 7.286 7.281 7.277 7.272 7.268 7.260 7.260 CDCl3 7.255 7.252 7.190 7.184 7.178 7.175 7.170 7.166 5.299 5.072 5.069 5.063 5.060 4.909 4.882 4.694 4.686 4.675 4.664 4.658 4.646 4.618 4.547 4.539 4.517 4.512 4.468 4.438 4.063 4.053 4.047 4.041 4.025 3.842 3.837 3.823 3.817 3.813 3.806 3.796 3.780 3.770 3.684 3.680 3.666 3.658 3.654 3.643 3.620 2.361 2.357 2.348 2.344 2.328 2.324 2.315 2.311 1.794 1.784 1.765 1.761 1.756 1.752
BnO
BnO
OBn
O
O
OBn
OBn
O
BnO
7g
16.11
1.88
1.00
1.06
3.78
1.96
1.01
1.05
2.02
1.98
1.01
1.00
f1 (ppm)

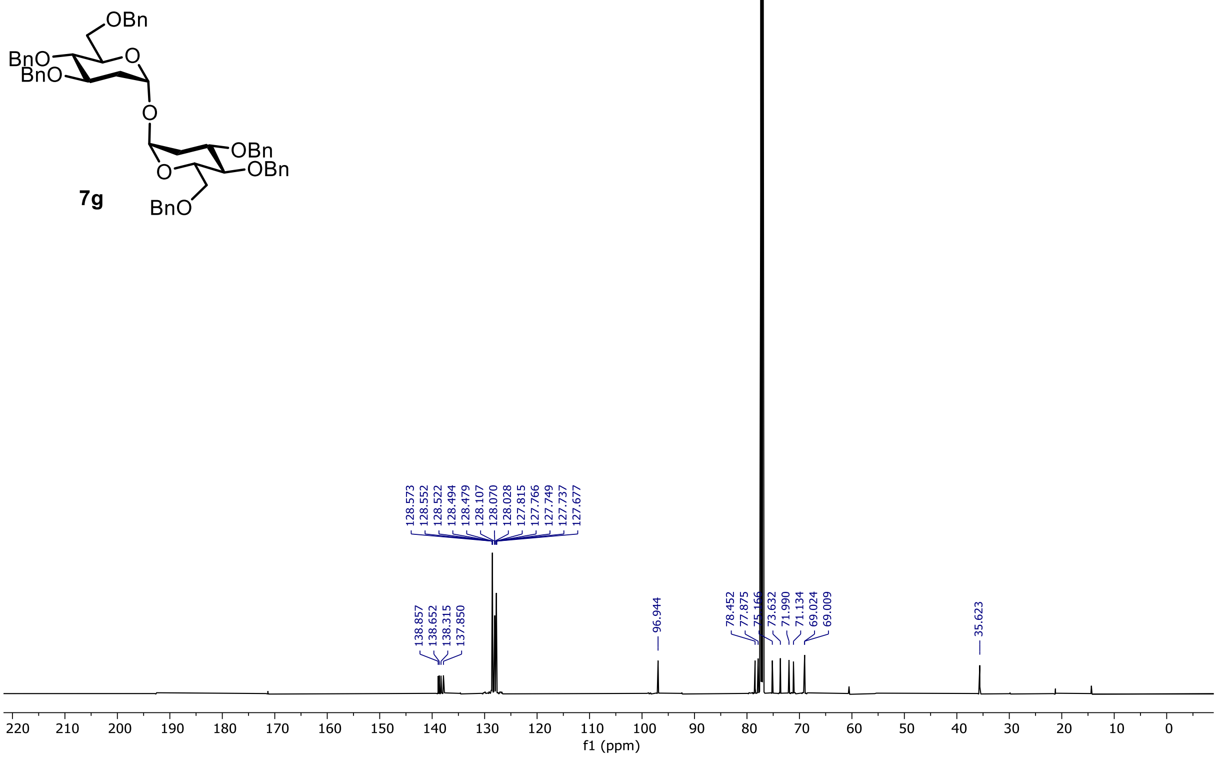
OBn
BnO
BnO
O
O
OBn
OBn
O
BnO
7g
128.573 128.552 128.522 128.494 128.479 128.107 128.070 128.028 127.815 127.766 127.749 127.737 127.677
138.857 138.652 138.315 137.850
96.944
78.452 77.875 75.166 73.632 71.990 71.134 69.024 69.009
35.623
f1 (ppm)

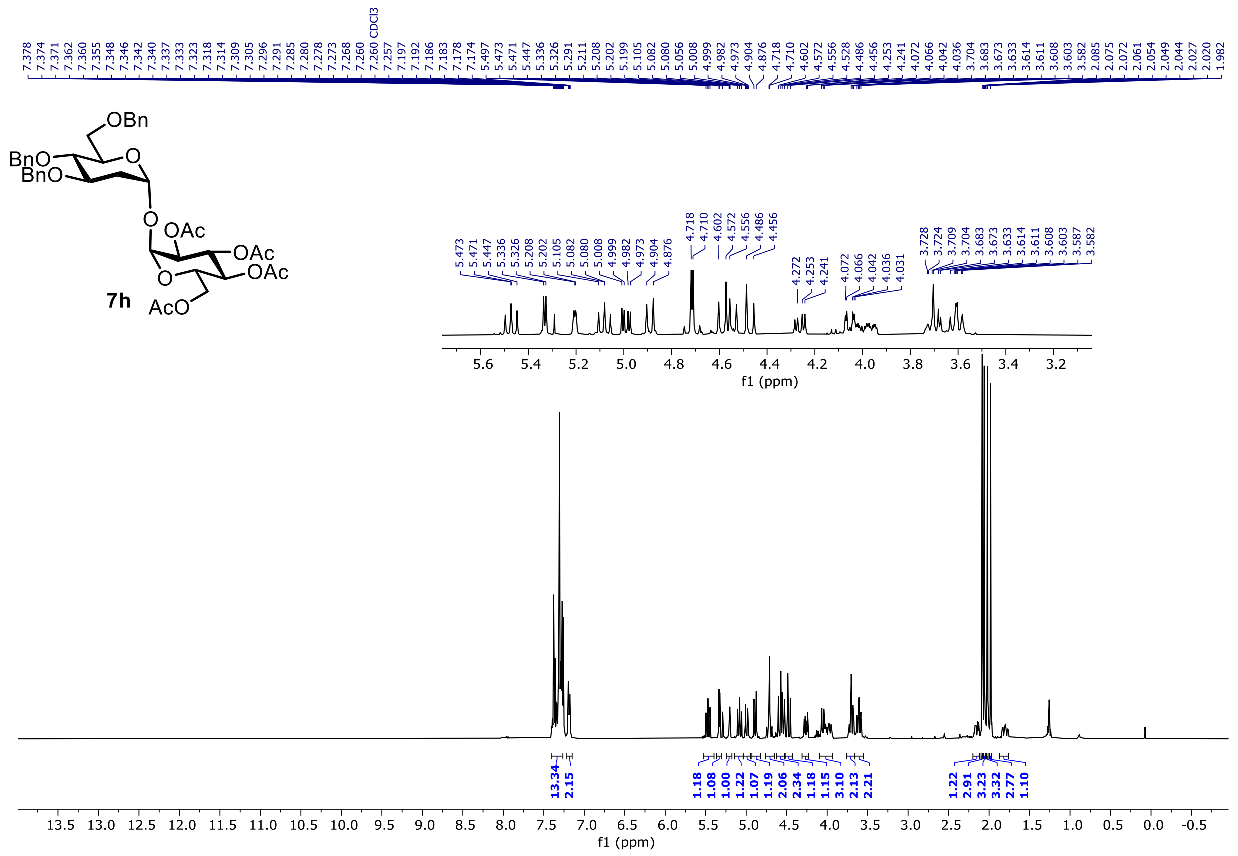

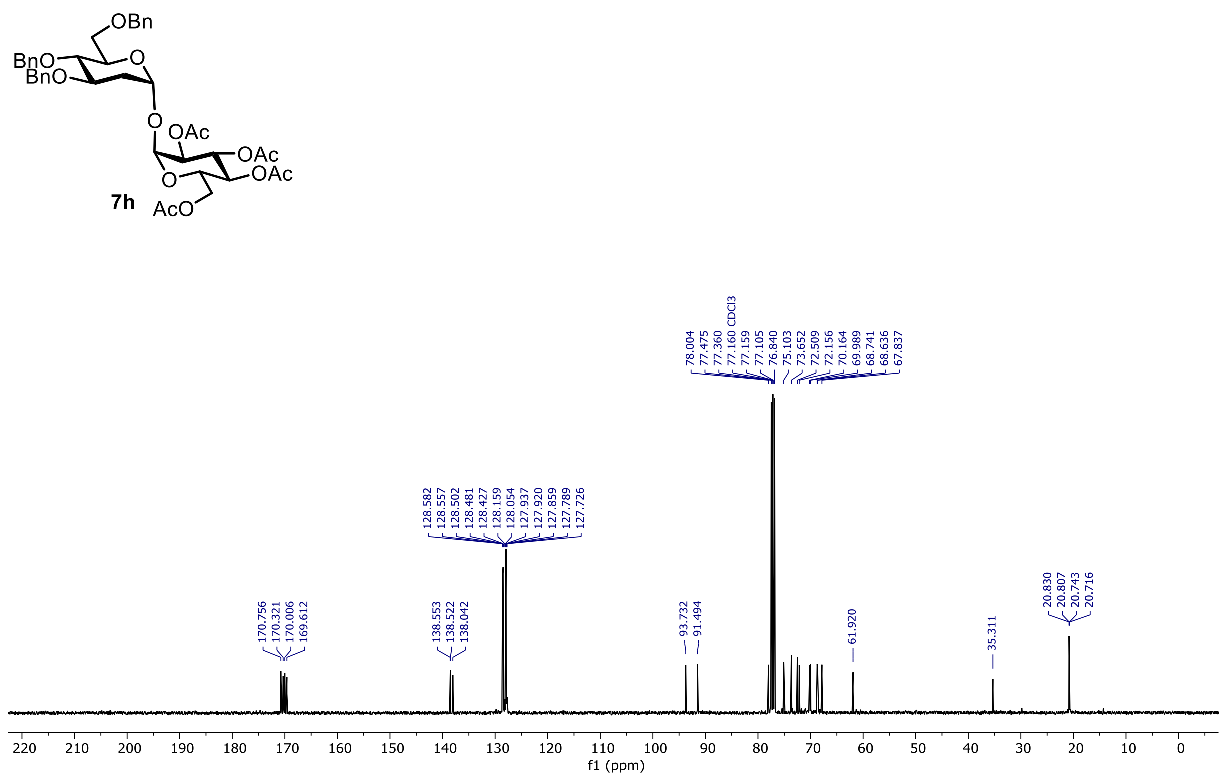

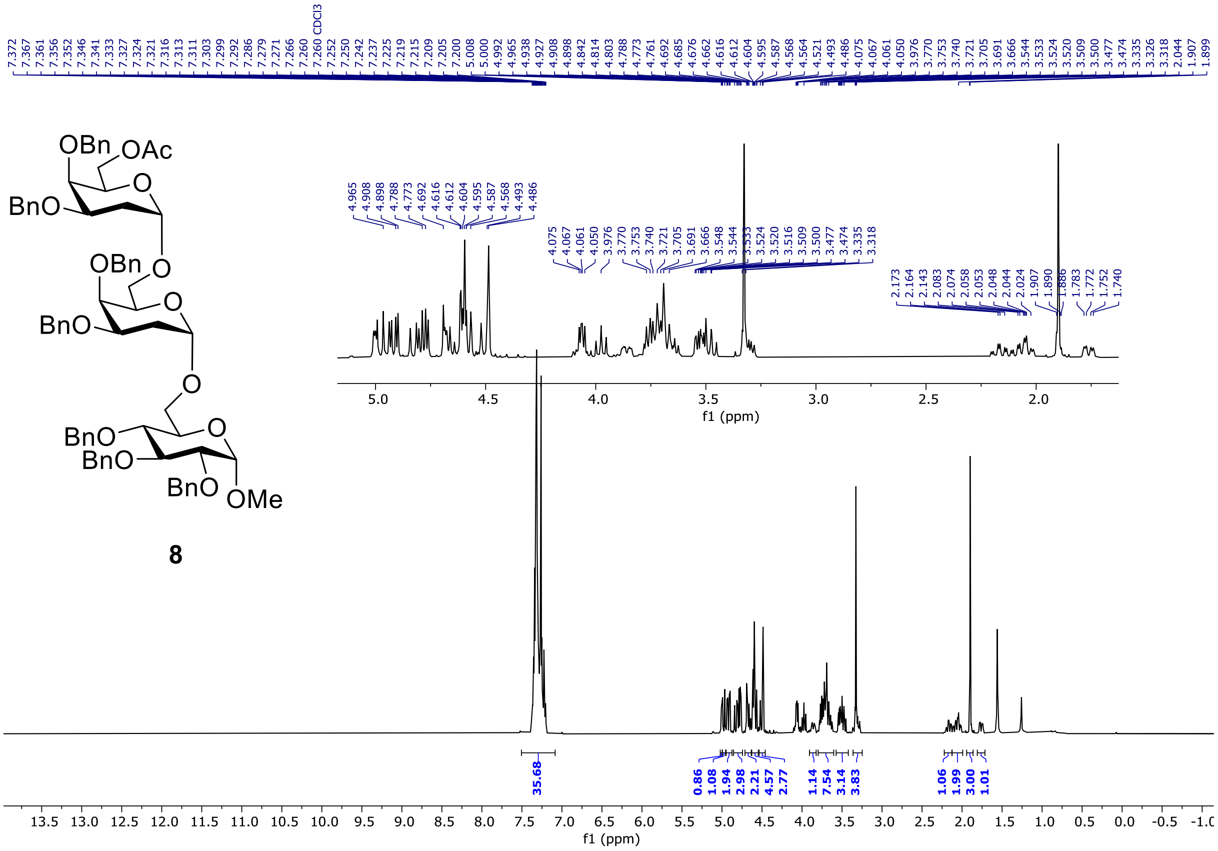

8

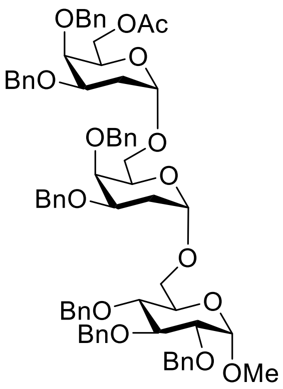
OBn
OAc
O
BnO
OBn
O
O
BnO
O
BnO
O
BnO
BnO
OMe

8

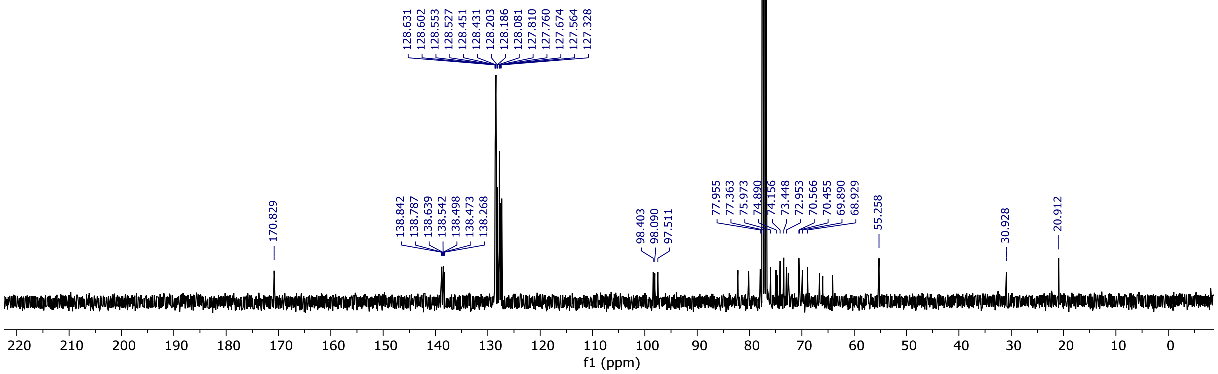
128.631
128.602
128.553
128.527
128.451
128.431
128.203
128.186
128.081
127.810
127.760
127.674
127.564
127.328
170.829
138.842
138.787
138.639
138.542
138.498
138.473
138.268
98.403
98.090
97.511
77.955
77.363
75.973
74.890
74.156
73.448
72.953
70.566
70.455
69.890
68.929
55.258
30.928
20.912
220 210 200 190 180 170 160 150 140 130 120 110 100 90 80 70 60 50 40 30 20 10 0
f1 (ppm)

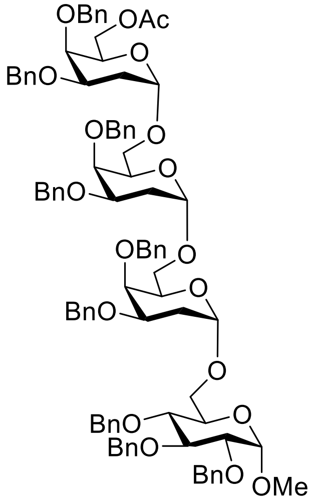
OBn
OAc
O
BnO
OBn
O
O
BnO
OBn
O
O
BnO
O
BnO
O
BnO
BnO
OMe

9

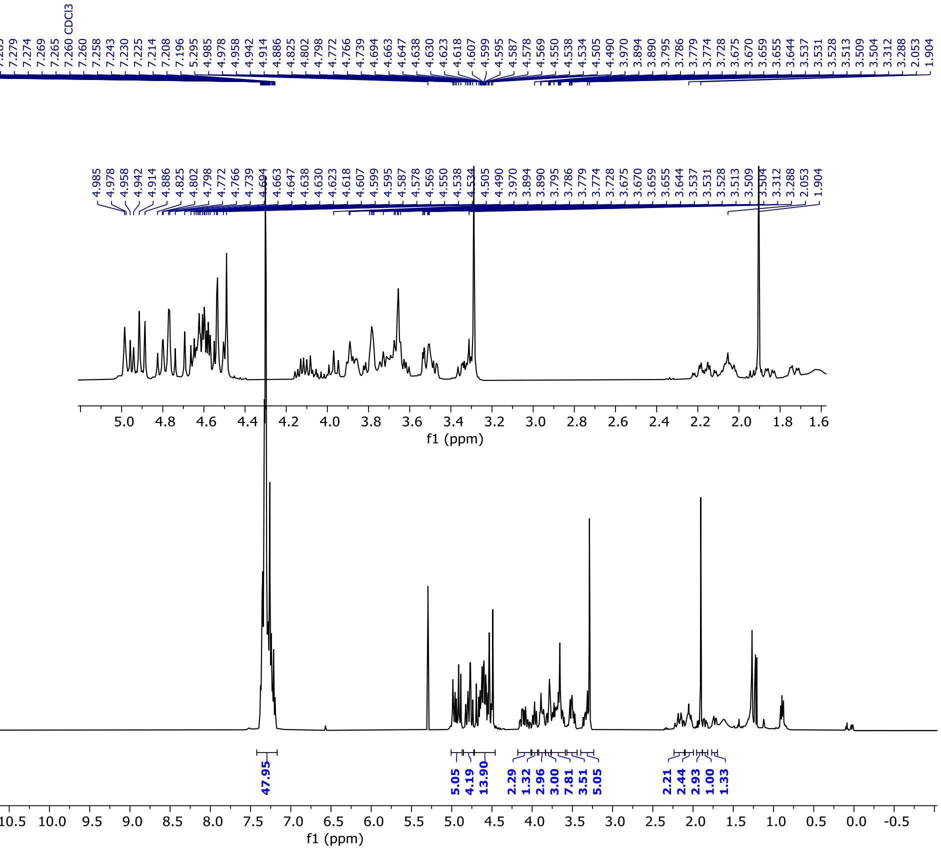
7.377
7.372
7.365
7.361
7.356
7.350
7.347
7.342
7.339
7.331
7.326
7.323
7.317
7.312
7.306
7.302
7.297
7.291
7.287
7.283
7.279
7.274
7.269
7.265
7.260 CDCl3
7.260
7.258
7.243
7.230
7.225
7.214
7.208
7.196
5.295
4.985
4.978
4.958
4.942
4.914
4.886
4.825
4.802
4.798
4.772
4.766
4.739
4.694
4.663
4.647
4.638
4.630
4.623
4.618
4.607
4.599
4.595
4.587
4.578
4.569
4.550
4.538
4.534
4.505
4.490
3.970
3.894
3.890
3.795
3.786
3.779
3.774
3.728
3.675
3.670
3.659
3.655
3.644
3.537
3.531
3.528
3.513
3.509
3.504
3.312
3.288
2.053
1.904
5.0 4.8 4.6 4.4 4.2 4.0 3.8 3.6 3.4 3.2 3.0 2.8 2.6 2.4 2.2 2.0 1.8 1.6
f1 (ppm)
47.95
5.05
4.19
13.90
2.29
1.32
2.96
3.00
7.81
3.51
5.05
2.21
2.44
2.93
1.00
1.33
13.5 13.0 12.5 12.0 11.5 11.0 10.5 10.0 9.5 9.0 8.5 8.0 7.5 7.0 6.5 6.0 5.5 5.0 4.5 4.0 3.5 3.0 2.5 2.0 1.5 1.0 0.5 0.0 -0.5
f1 (ppm)

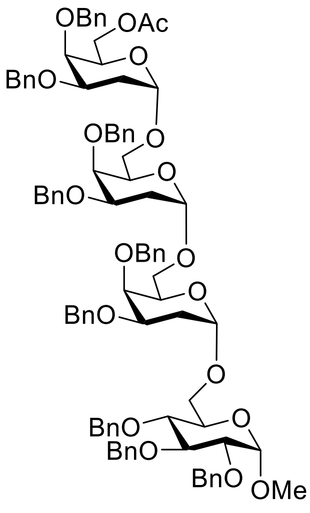

9

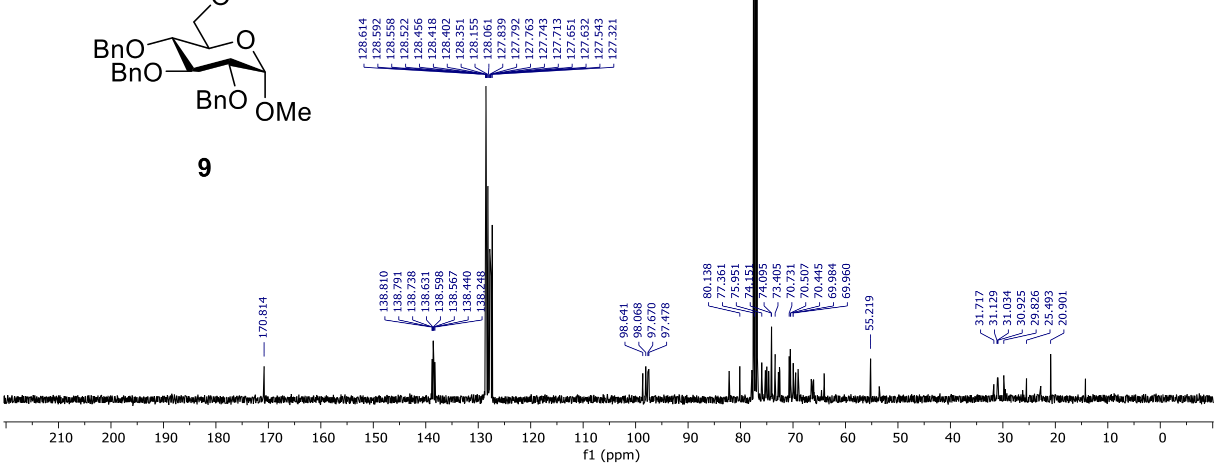

References

(1) Zhang, Q.; Chan, Y.-Y.; Zhang, M.; Yeung, Y.-Y.; Ke, Z. Hypervalent Chalcogenonium···π Bonding Catalysis. *Angew Chem Int Ed* **2022**, *61* (35), e202208009. DOI: 10.1002/anie.202208009.

(2) Balmond, E. I.; Benito‐Alifonso, D.; Coe, D. M.; Alder, R. W.; McGarrigle, E. M.; Galan, M. C. A 3, 4‐trans‐Fused Cyclic Protecting Group Facilitates α‐Selective Catalytic Synthesis of 2‐Deoxyglycosides. *Angew Chem Int Ed* **2014**, *126* (31), 8329-8333. Khan, A. T.; Musawwer Khan, M. A simple and convenient synthetic protocol for O-isopropylidenation of sugars using bromodimethylsulfonium bromide (BDMS) as a catalyst. *Carbohydr Res* **2010**, *345* (1), 154-159. DOI: 10.1016/j.carres.2009.09.017.

(3) Palo-Nieto, C.; Sau, A.; Williams, R.; Galan, M. C. Cooperative Bronsted Acid-Type Organocatalysis for the Stereoselective Synthesis of Deoxyglycosides. *J Org Chem* **2017**, *82* (1), 407-414. DOI: 10.1021/acs.joc.6b02498.

(4) Szechner, B.; Urbańczyk-Lipkowska, Z.; Chmielewski, M. Glycosyl hydroperoxides derived from 2-deoxysugars. *Carbohyd Res* **2010**, *345* (17), 2464-2468. DOI: 10.1016/j.carres.2010.09.005.

(5) Schene, H.; Waldmann, H. Synthesis of deoxy glycosides under neutral conditions in LiClO4/solvent mixtures. *Synthesis* **1999**, *1999* (S 01), 1411-1422.

(6) Kumar, M.; Gurawa, A.; Kumar, N.; Kashyap, S. Bismuth-Catalyzed Stereoselective 2-Deoxyglycosylation of Disarmed/Armed Glycal Donors. *Org Lett* **2022**, *24* (2), 575-580. DOI: 10.1021/acs.orglett.1c04008.

(7) Palo-Nieto, C.; Sau, A.; Galan, M. C. Gold(I)-Catalyzed Direct Stereoselective Synthesis of Deoxyglycosides from Glycals. *J Am Chem Soc* **2017**, *139* (40), 14041-14044. DOI: 10.1021/jacs.7b08898.

(8) Mishra, B.; Yuan, Y.; Yu, H.; Kang, H.; Gao, J.; Daniels, R.; Chen, X. Synthetic Sialosides Terminated with 8-N-Substituted Sialic Acid as Selective Substrates for Sialidases from Bacteria and Influenza Viruses. *Angew Chem Int Ed* **2024**, *63* (29), e202403133. DOI: 10.1002/anie.202403133.

(9) te Velde, G.; Bickelhaupt, F. M.; Baerends, E. J.; Fonseca Guerra, C.; van Gisbergen, S. J. A.; Snijders, J. G.; Ziegler, T. Chemistry with ADF. *J Comp Chem* **2001**, *22* (9), 931-967. DOI: 10.1002/jcc.1056. Fonseca Guerra, C.; Snijders, J. G.; te Velde, G.; Baerends, E. J. Towards an order-N DFT method. *Theo Chem Acc* **1998**, *99* (6), 391-403. DOI: 10.1007/s002140050353.

(10) AMS2023.101, SCM Theoretical Chemistry; Vrije Universiteit, Amsterdam, The Netherlands, http://www.scm.com
(11) a) Becke, A. D. Density-functional exchange-energy approximation with correct asymptotic behavior. *Phys Rev A* **1988**, *38* (6), 3098-3100. DOI: 10.1103/PhysRevA.38.3098; b) Russo, T. V.; Martin, R. L.; Hay, P. J. Density functional calculations on first - row transition metals. *J Chem Phys* **1994**, *101* (9), 7729-7737. DOI: 10.1063/1.468265; c) Lee, C.; Yang, W.; Parr, R. G. Development of the Colle-Salvetti correlation-energy formula into a functional of the electron density. *Physical Review B* **1988**, *37* (2), 785-789. DOI: 10.1103/PhysRevB.37.785; d) Johnson, B. G.; Gill, P. M. W.; Pople, J. A. The performance of a family of density functional methods. *J Chem Phys* **1993**, *98* (7), 5612-5626. DOI: 10.1063/1.464906.

(12) Grimme, S.; Ehrlich, S.; Goerigk, L. Effect of the damping function in dispersion corrected density functional theory. *J Comp Chem* **2011**, *32* (7), 1456-1465. DOI: 10.1002/jcc.21759.

(13) van Lenthe, E.; Baerends, E. J.; Snijders, J. G. Relativistic total energy using regular approximations. *J Chem Phys* **1994**, *101* (11), 9783-9792. DOI: 10.1063/1.467943.

(14) Van Lenthe, E.; Baerends, E. J. Optimized Slater-type basis sets for the elements 1–118. *J Comp Chem* **2003**, *24* (9), 1142-1156. DOI: 10.1002/jcc.10255.

(15) a) Franchini, M.; Philipsen, P. H. T.; van Lenthe, E.; Visscher, L. Accurate Coulomb Potentials for Periodic and Molecular Systems through Density Fitting. *J Chem Theor Comp* **2014**, *10* (5), 1994-2004. DOI: 10.1021/ct500172n. b) Franchini, M.; Philipsen, P. H. T.; Visscher, L. The Becke Fuzzy Cells Integration Scheme in the Amsterdam Density Functional Program Suite. *J Comp Chem* **2013**, *34* (21), 1819-1827. DOI: DOI:10.1002/jcc.23323.

(16) a) Klamt, A.; Schüürmann, G. COSMO: a new approach to dielectric screening in solvents with explicit expressions for the screening energy and its gradient. *J Chem Soc Perkin Trans 2* **1993**, (5), 799-805. DOI: 10.1039/P29930000799. b) Klamt, A. Conductor-like Screening Model for Real Solvents: A New Approach to the Quantitative Calculation of Solvation Phenomena. *J Phys Chem* **1995**, *99* (7), 2224-2235. DOI: 10.1021/j100007a062. c) Klamt, A.; Jonas, V. Treatment of the outlying charge in continuum solvation models. *The J Chem Phys* **1996**, *105* (22), 9972-9981. DOI: 10.1063/1.472829. d) Pye, C. C.; Ziegler, T. An implementation of the conductor-like screening model of solvation within the Amsterdam density functional package. *Theor Chem Acc* **1999**, *101* (6), 396-408. DOI: 10.1007/s002140050457.

(17) Zhao, Y.; Truhlar, D. G. The M06 suite of density functionals for main group thermochemistry, thermochemical kinetics, noncovalent interactions, excited states, and transition elements: two new functionals and systematic testing of four M06-class functionals and 12 other functionals. *Theor Chem Acc* **2008**, *120* (1), 215-241. DOI: 10.1007/s00214-007-0310-x.

(18) RDKit: Open-source cheminformatics. https://www.rdkit.org. DOI: 10.5281/zenodo.591637.
(19) a) Bérces, A.; Dickson, R. M.; Fan, L.; Jacobsen, H.; Swerhone, D.; Ziegler, T. An implementation of the coupled perturbed Kohn-Sham equations: perturbation due to nuclear displacements. *Comp Phys Commun* **1997**, *100* (3), 247-262. DOI:10.1016/S0010-4655(96)00120-8. b) Jacobsen, H.; Bérces, A.; Swerhone, D. P.; Ziegler, T. Analytic second derivatives of molecular energies: a density functional implementation. *Comp Phys Commun* **1997**, *100* (3), 263-276. DOI:10.1016/S0010-4655(96)00119-1. Wolff, S. K. Analytical second derivatives in the Amsterdam density functional package. *Int J Quant Chem* **2005**, *104* (5), 645-659. DOI: DOI:10.1002/qua.20653.

(20) C. Y. Legault, CYLview, 1.0b; Université de Sherbrooke, Canada, Sherbrooke, QC, 2009, http://www.cylview.org.

(21) a) Martin, R. L.; Hay, P. J.; Pratt, L. R. Hydrolysis of Ferric Ion in Water and Conformational Equilibrium. *J Phys Chem A* **1998**, *102* (20), 3565-3573. DOI: 10.1021/jp980229p. Ariai, J.; Gellrich, U. The entropic penalty for associative reactions and their physical treatment during routine computations. *Physical Chemistry Chemical Physics* **2023**, *25* (20), 14005-14015. DOI: 10.1039/D3CP00970J. b) González-Fabra, J.; Castro-Gómez, F.; Sameera, W. M. C.; Nyman, G.; Kleij, A. W.; Bo, C. Entropic corrections for the evaluation of the catalytic activity in the Al(iii) catalysed formation of cyclic carbonates from CO2 and epoxides. *Cat Sci Tech* **2019**, *9* (19), 5433-5440. DOI: 10.1039/C9CY01285K.
